# Supplementary material for: Synthetic prodrug design enables biocatalytic activation in mice to elicit tumor growth suppression
Source: Nat Commun. 2022 Jan 10;13:39. doi: 10.1038/s41467-021-27804-5 (PMC8748823; doi:10.1038/s41467-021-27804-5)
Supplement: Supplementary file 1 — Supplementary information [file 41467_2021_27804_MOESM1_ESM.pdf]

Supplementary information

Synthetic prodrug design enables biocatalytic activation in living mice to elicit tumour growth suppression

Igor Nasibullin,[a] Ivan Smirnov,[b] Peni Ahmadi,[a] Kenward Vong,[c] Almira Kurbangalieva,[b] Katsunori Tanaka*[a,b,d,e]

[a] Biofunctional Synthetic Chemistry Laboratory, RIKEN Cluster for Pioneering Research, 2-1 Hirosawa, Wako-shi, Saitama, 351-0198, Japan,
[b] Biofunctional Chemistry Laboratory, A. Butlerov Institute of Chemistry, Kazan Federal University, 18 Kremlyovskaya street, Kazan, 420008, Russia
[c] Department of Chemistry, The Hong Kong University of Science and Technology, Clear Water Bay, Kowloon, Hong Kong, China
[d] GlycoTargeting Research Laboratory, RIKEN Baton Zone Program, 2-1 Hirosawa, Wako-shi, Saitama, 351-0198, Japan
[e] Department of Chemical Science and Engineering, School of Materials and Chemical Technology, Tokyo Institute of Technology, 2-12-1 O-okayama, Meguro-ku, Tokyo, 152-8552, Japan

E-mail: tanaka.k.dg@m.titech.ac.jp

Table of contents

1. SUPPLEMENTARY METHODS. 2
1.1. Preparation and characterization of ArM 2
1.2. HPLC Analysis 4
1.2.1. HPLC standard curves and calibration 5
1.2.2. Example HPLC traces 11
1.3. Modeling Studies 28
1.4. Activity studies 29
1.4.1. Substrate scope investigation 29
1.4.2. Michaelis–Menten kinetic experiments 30
1.5. Biological experiments 33
1.6. Synthesis of substrates 38
2. Supplementary figures (^{1}H and ^{13}C NMR spectra) 55
3. Supplementary References 92

1. SUPPLEMENTARY METHODS.

1.1. Preparation and characterization of ArM

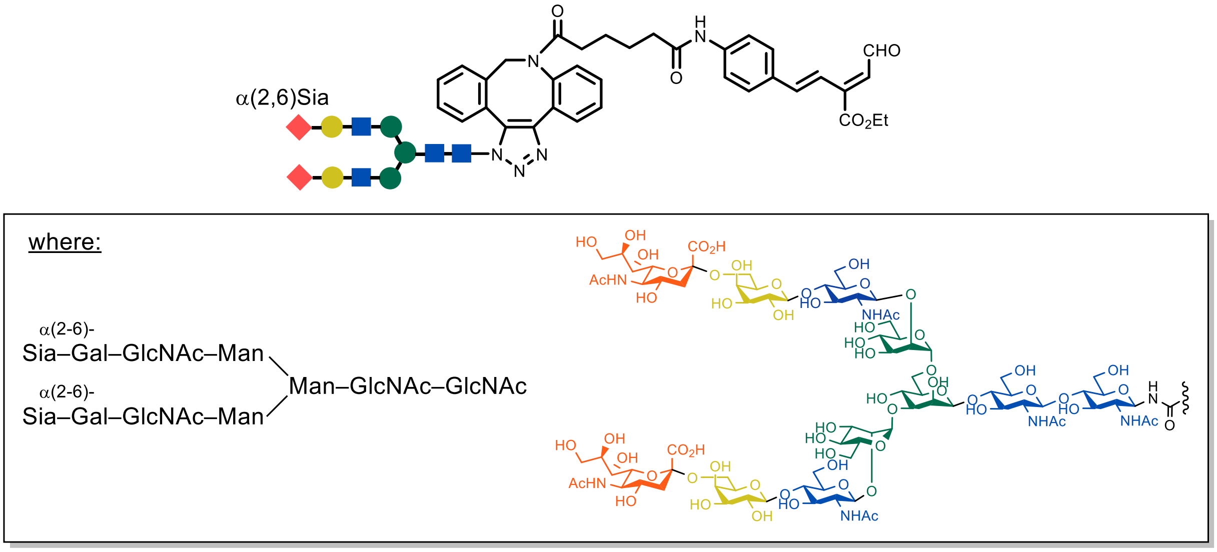

Supplementary Figure 1. Structure of the α(2,6)-Sia terminated glycan-aldehyde probe **21**

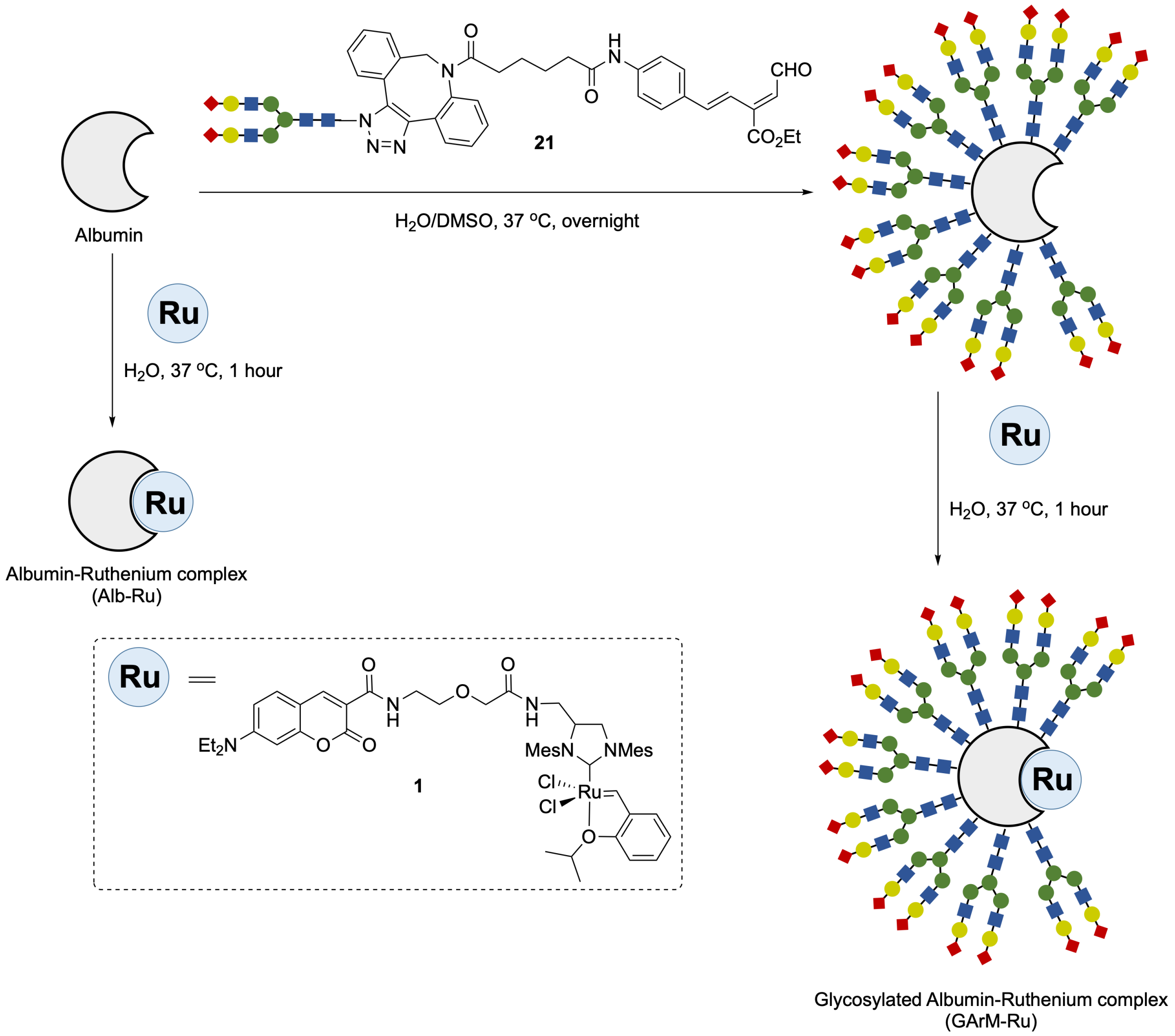

Supplementary Figure 2. Preparation of the protein complexes Alb-Ru, GlycoAlb, GArM-Ru.

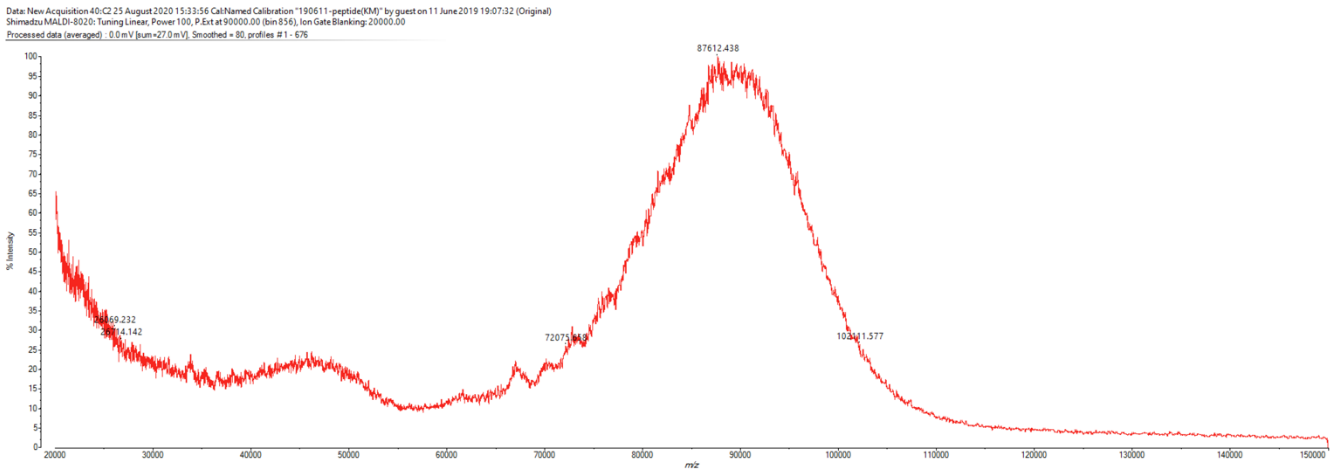

Supplementary Figure 3. MALDI-TOF-MS spectra of GlycoAlb

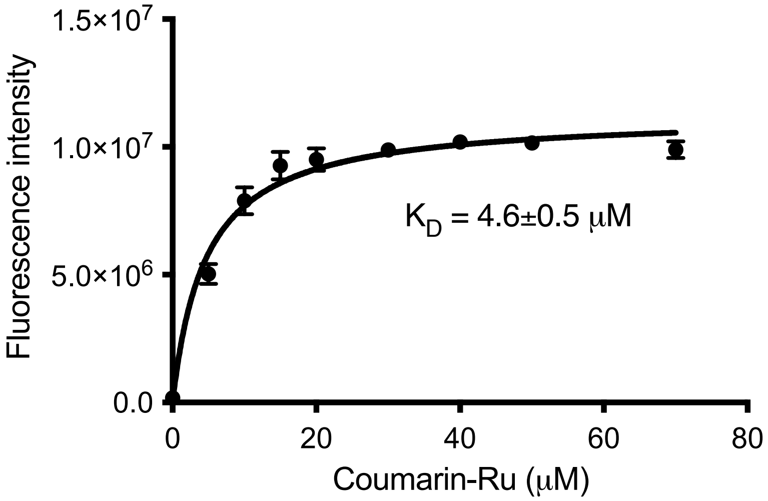

Supplementary Figure 4. Saturation binding curves based on the fluorescence emitted from Coumarin-Ru, when bound to GlycoAlb. Experiment was conducted at GlycoAlb concentration of 10 μM. The measured equilibrium dissociation constant (K_D) was determined by non-linear regression. Incubation was performed at 37 °C for 1 hour in 10% 1,4-dioxane/PBS mixture and monitored at λ_{EX}=425 nm/λ_{EM}=460 nm. Error bars represent the standard deviation of three replicate measurements. Fluorescence was read through a SpectraMax iD3 multi-mode microplate reader (Molecular Devices), data was collected using SoftMax Pro version 7.0.3 software

1.2. HPLC Analysis

Supplementary Table 1. Gradient profiles for HPLC analysis

	Flow rate (ml/min)	Time (min)	%A (H_2O with or w/o 0.1% TFA)	%A (CH_3CN with or w/o 0.1% TFA)
Method 1	1.0	0	50	50
		20	10	90
		30	10	90
		31	50	50
		38	50	50
Method 2	1.0	0	70	30
		20	10	90
		22	10	90
		22.5	30	70
		30	70	30
Method 3	1.0	0	80	20
		30	10	90
		35	80	20
Method 4	1.0	0	50	50
		30	0	100
		35	50	50

1.2.1. HPLC standard curves and calibration

HPLC Method		Calibration Curve
Method 1		
Slope	94188	
Intercept	154688	
R^2	0.99991	

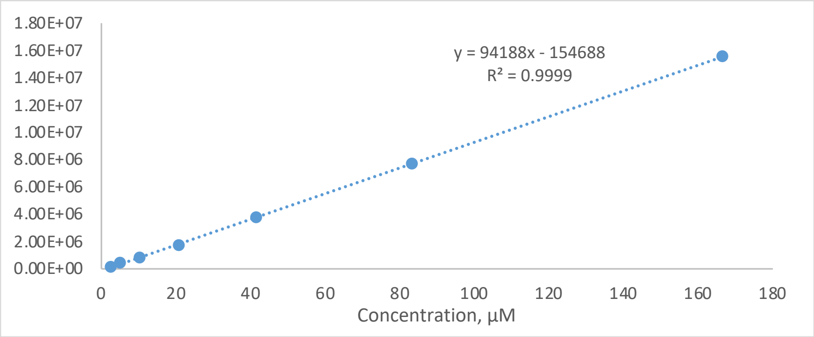

HPLC trace:

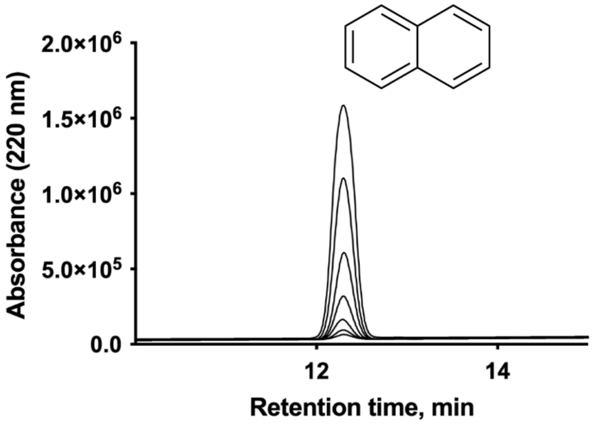

Supplementary Figure 5. HPLC calibration curve of product **13**

HPLC Method		Calibration Curve
Method 1		
Slope	33069	
Intercept	116819	
R^2	0.9994	

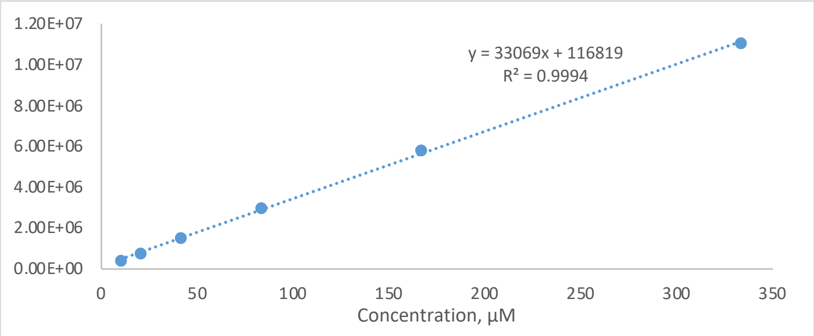

HPLC trace:

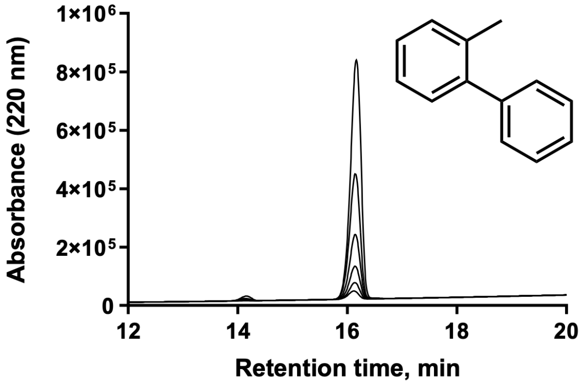

Supplementary Figure 6. HPLC calibration curve of product **3b**

HPLC Method	
Method 1	
Slope	102962
Intercept	62661.7
R^2	0.99991

Calibration Curve

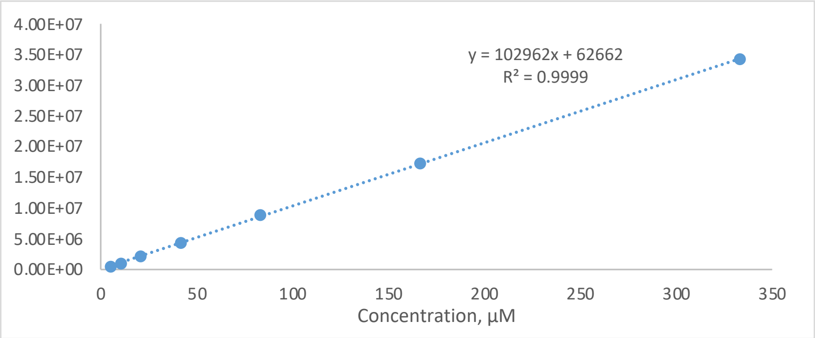

HPLC trace:

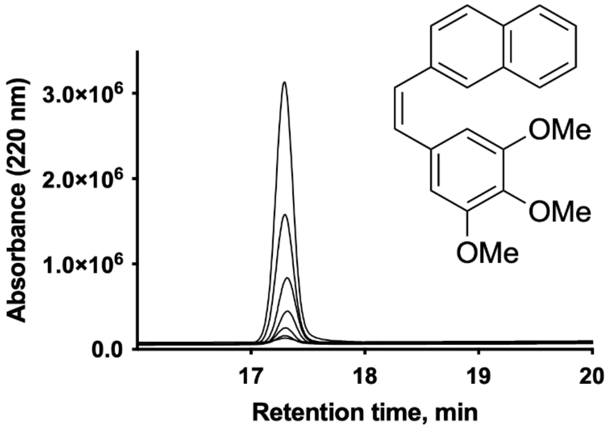

Supplementary Figure 7. HPLC calibration curve of product **20**

HPLC Method	
Method 1	
Slope	248212
Intercept	46128
R^2	0.99991

Calibration Curve

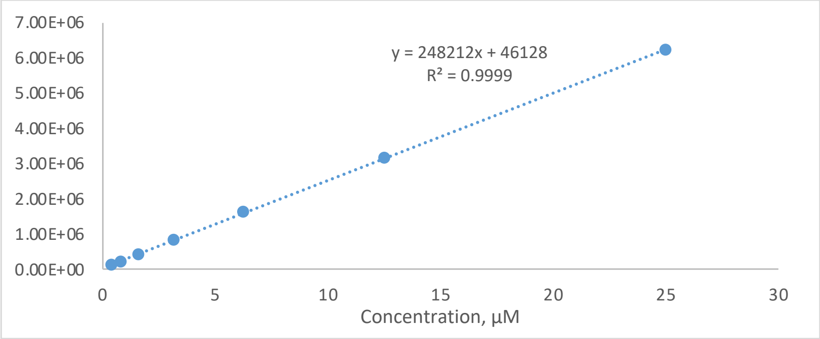

HPLC trace:

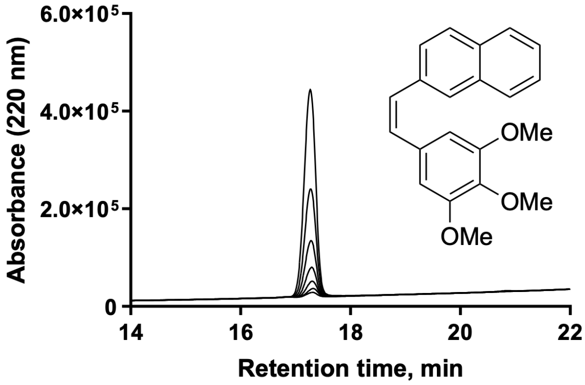

Supplementary Figure 8. HPLC calibration curve of product **20** for low concentrations (100 µM injection)

HPLC Method		Calibration Curve
Method 1		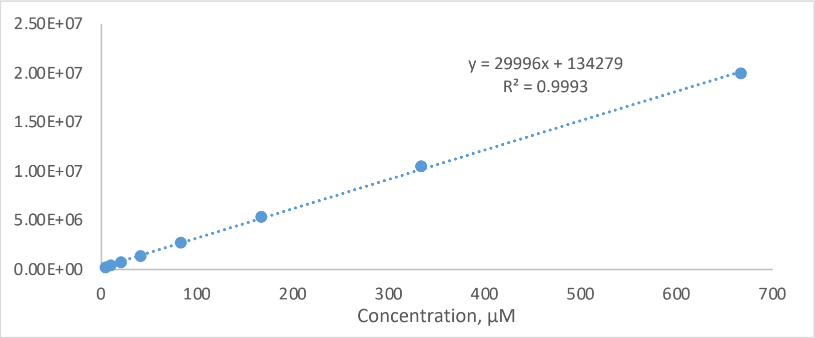
Slope	29996.16	
Intercept	134278.9	
R^2	0.999322	

HPLC trace:

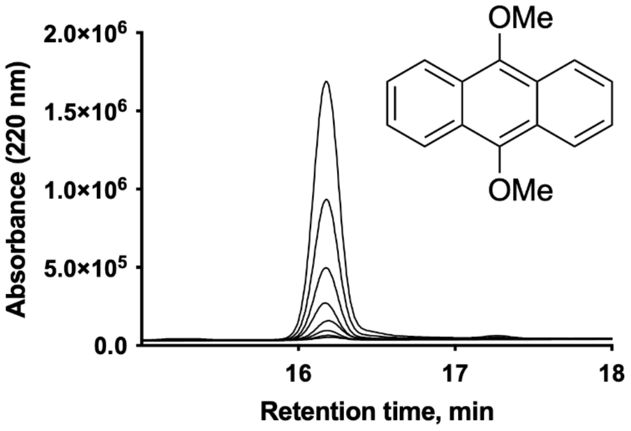

Supplementary Figure 9. HPLC calibration curve of product **4b**

HPLC Method		Calibration Curve
Method 4		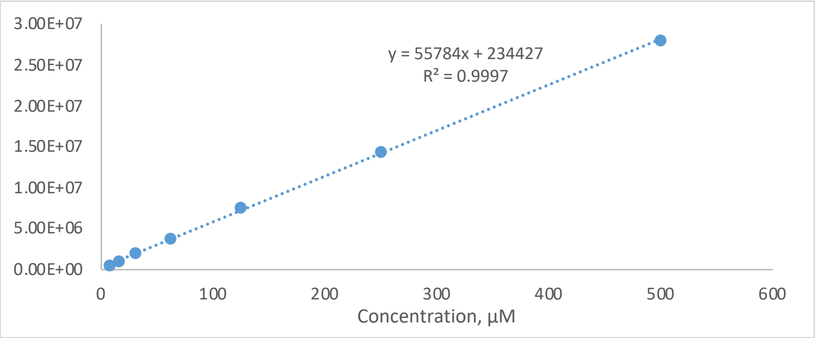
Slope	55784	
Intercept	234427	
R^2	0.9997	

HPLC trace:

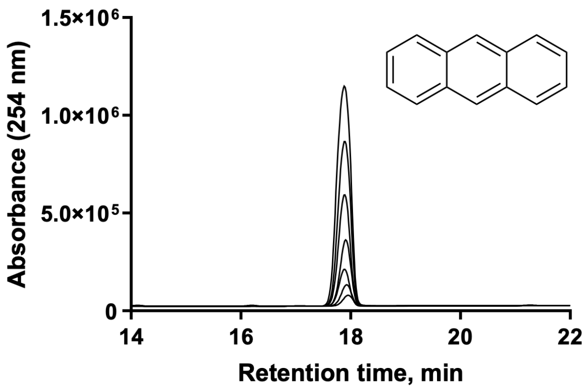

Supplementary Figure 10. HPLC calibration curve of product **5b**

HPLC Method		Calibration Curve
Method 4		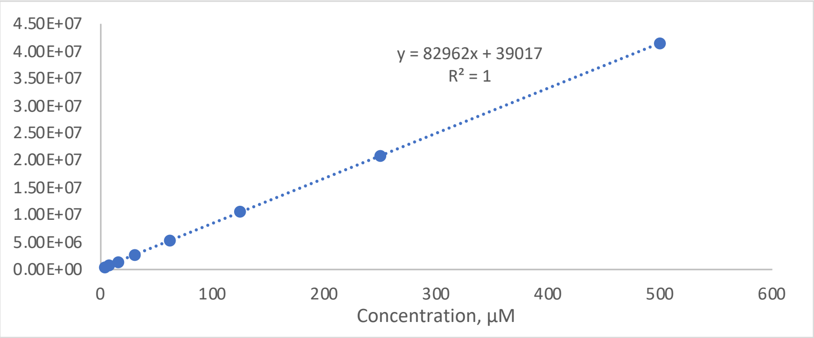
Slope	82962	
Intercept	39017	
R^2	1	

HPLC trace:

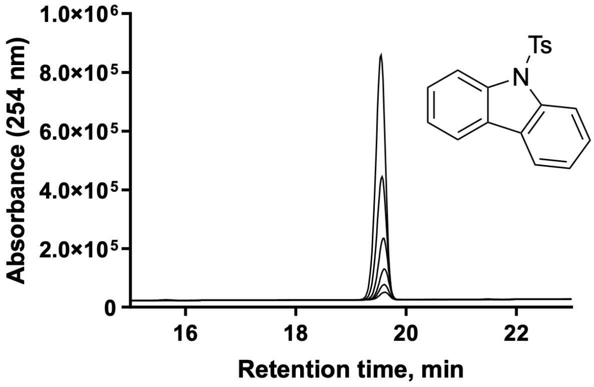

Supplementary Figure 11. HPLC calibration curve of product **6b**

HPLC Method		Calibration Curve
Method 2		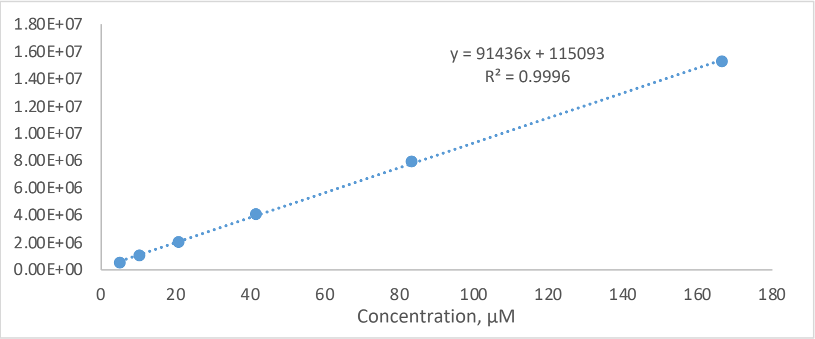
Slope	91436	
Intercept	115093	
R^2	0.9996	

HPLC trace:

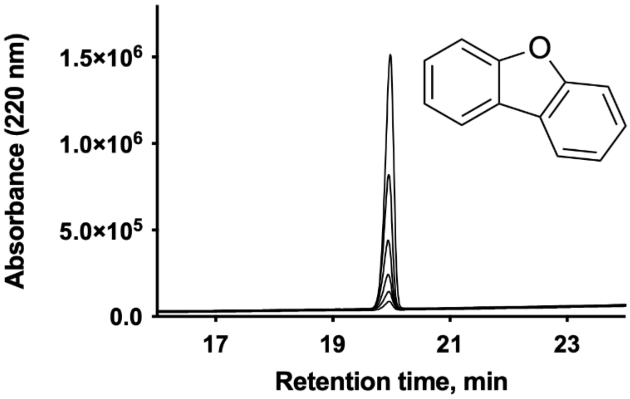

Supplementary Figure 12. HPLC calibration curve of product **7b**

HPLC Method	
Method 3	
Slope	32972
Intercept	144176
R^2	0.9998

Calibration Curve

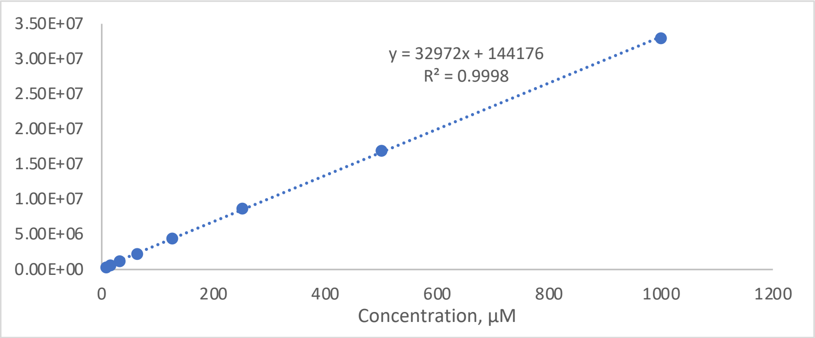

HPLC trace:

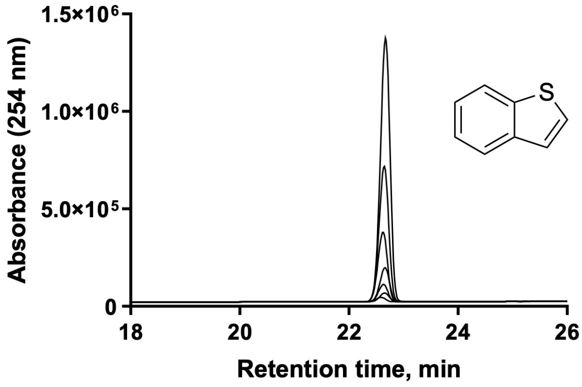

Supplementary Figure 13. HPLC calibration curve of product **8b**

HPLC Method	
Method 4	
Slope	302361
Intercept	41393
R^2	0.9997

Calibration Curve

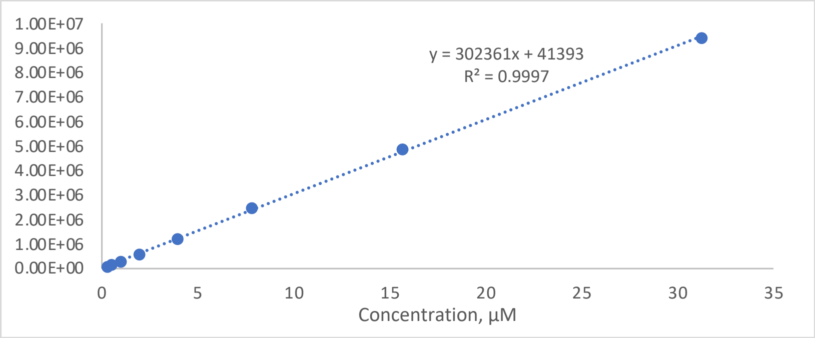

HPLC trace:

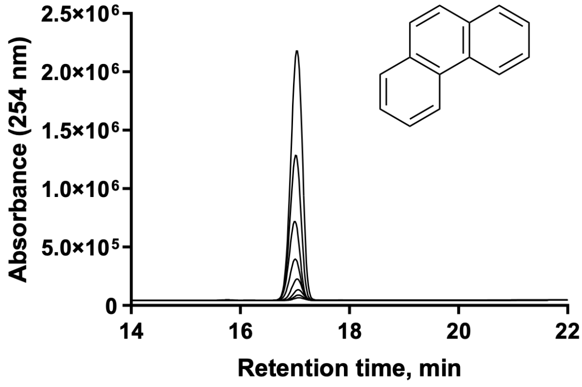

Supplementary Figure 14. HPLC calibration curve of product **9a**

HPLC Method		Calibration Curve
Method 4		
Slope	44232	
Intercept	924908	
R^2	0.9941	

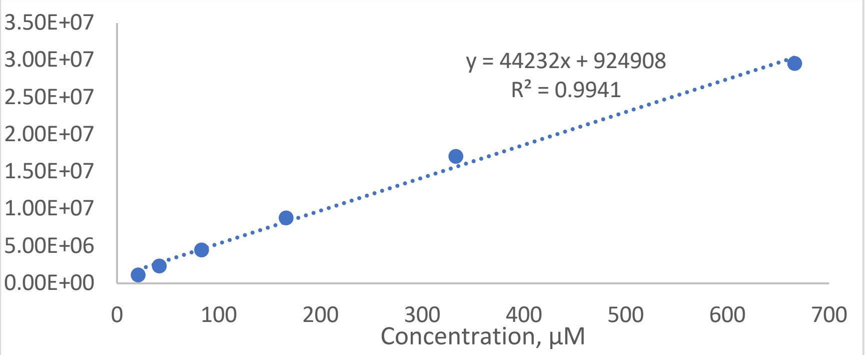

HPLC trace:

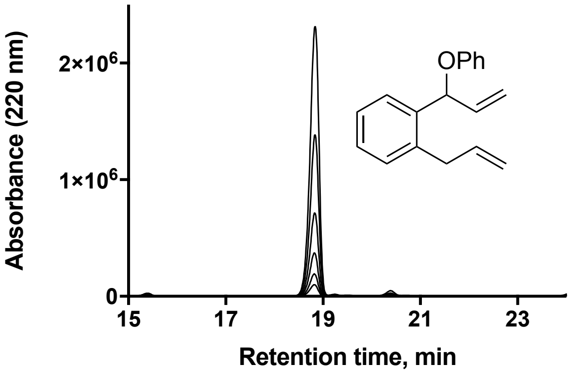

Supplementary Figure 15. HPLC calibration curve of substrate **11**

1.2.2. Example HPLC traces

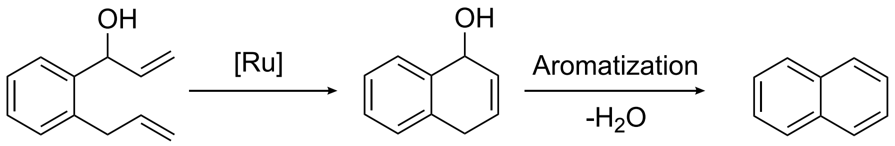

2 **2a** **13**

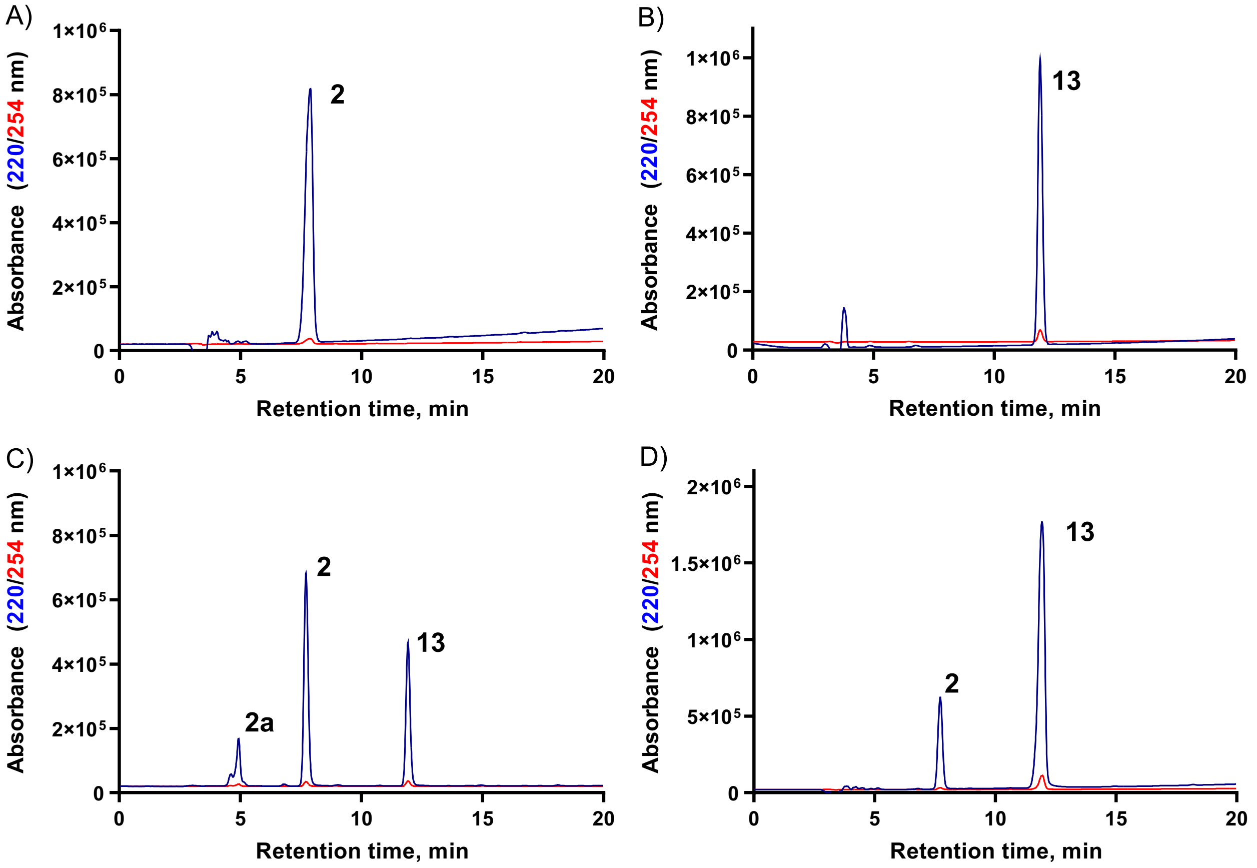

Supplementary Figure 16. Example HPLC traces of A) substrate **2**, B) product **13**, C) reaction analysis was run with neutral solvent system (CH_3CN/H_2O), D) reaction analysis was run with acidic solvent system (0.1% TFA in CH_3CN/H_2O).

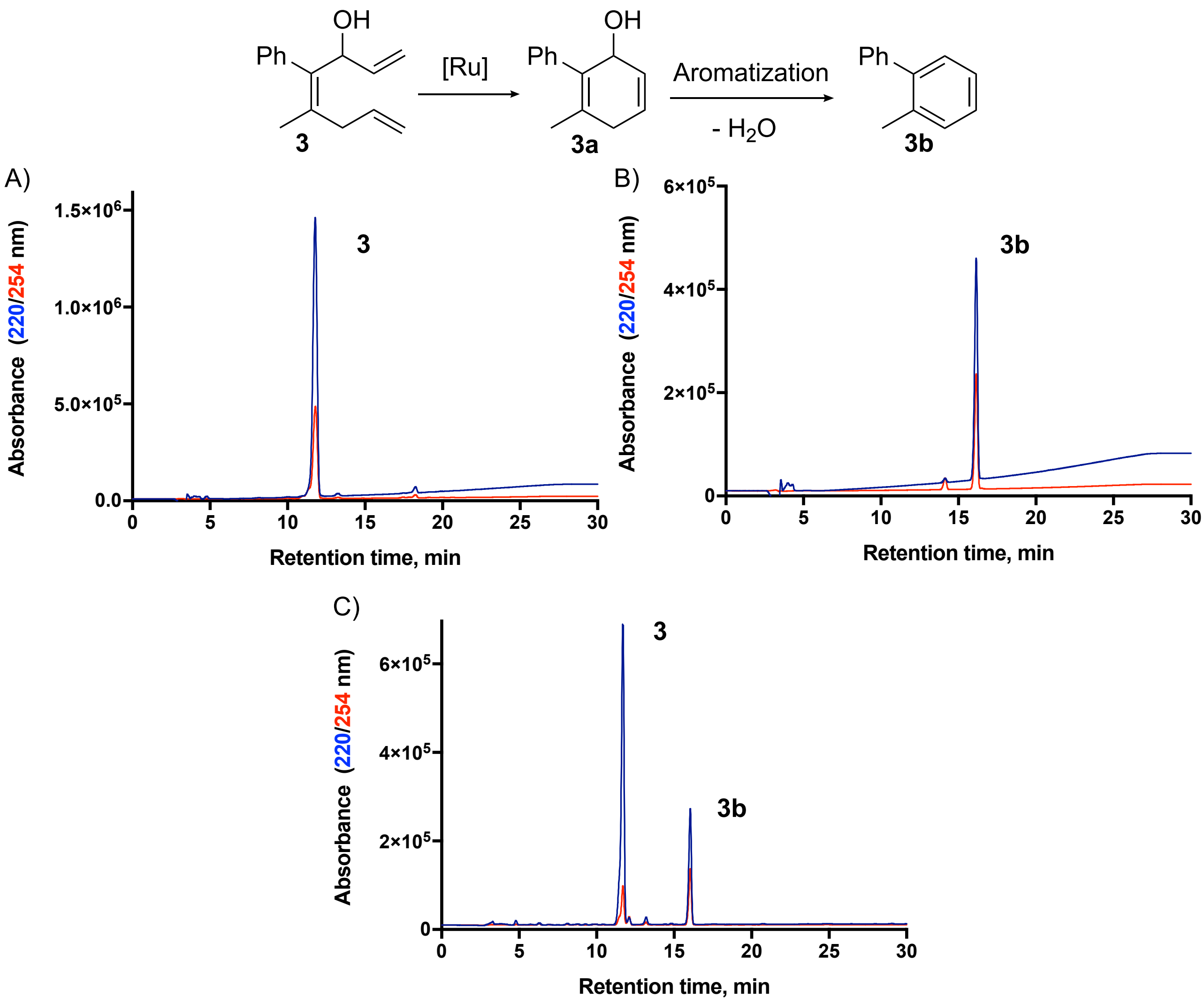

Supplementary Figure 17. Example HPLC traces of A) substrate **3**, B) product **3b**, C) reaction analysis was run with neutral solvent system (CH_3CN/H_2O),

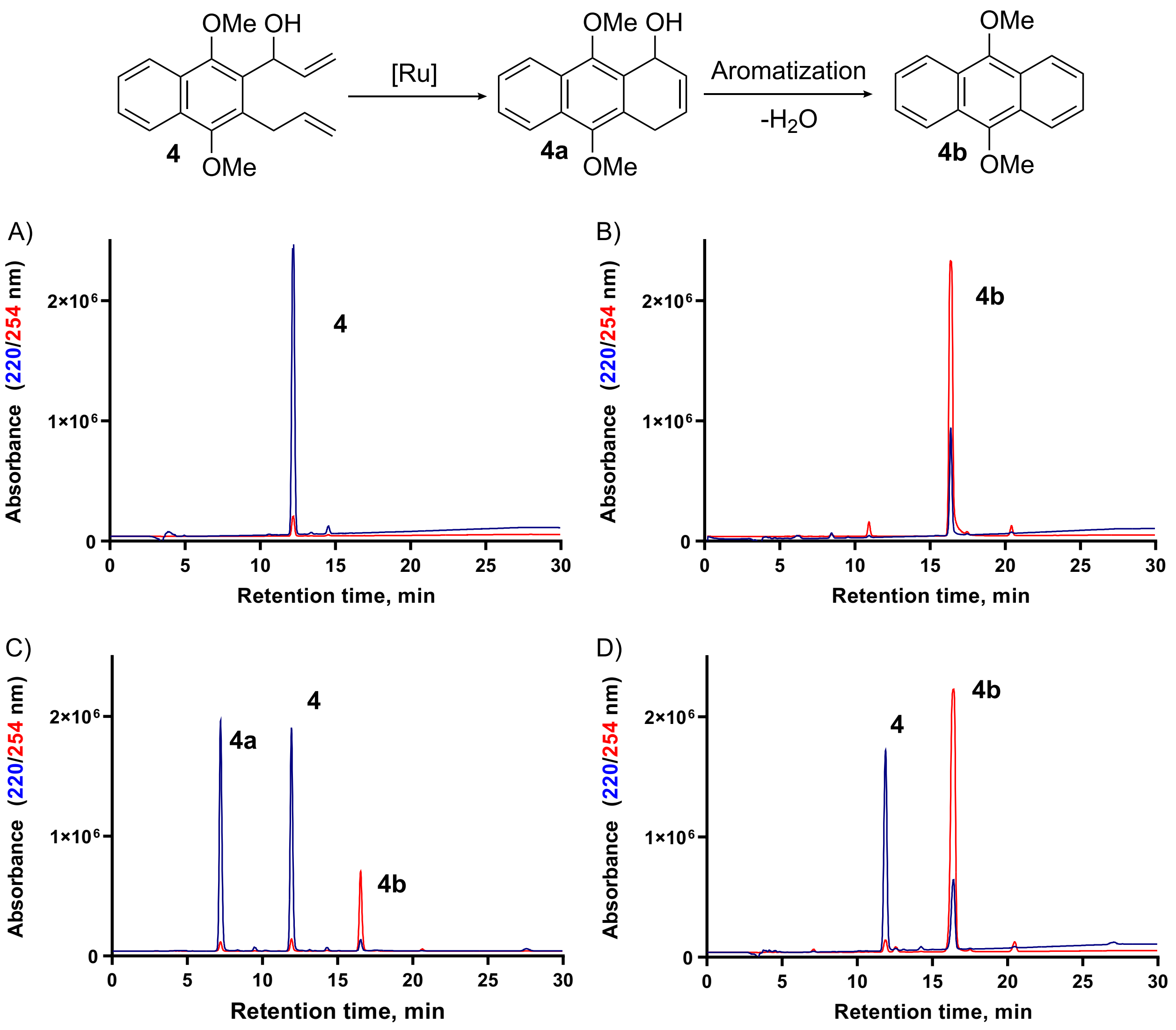

Supplementary Figure 18. Example HPLC traces of A) substrate **4**, B) product **4b**, C) reaction analysis was run with neutral solvent system (CH_3CN/H_2O), D) reaction analysis was run with acidic solvent system (0.1% TFA in CH_3CN/H_2O).

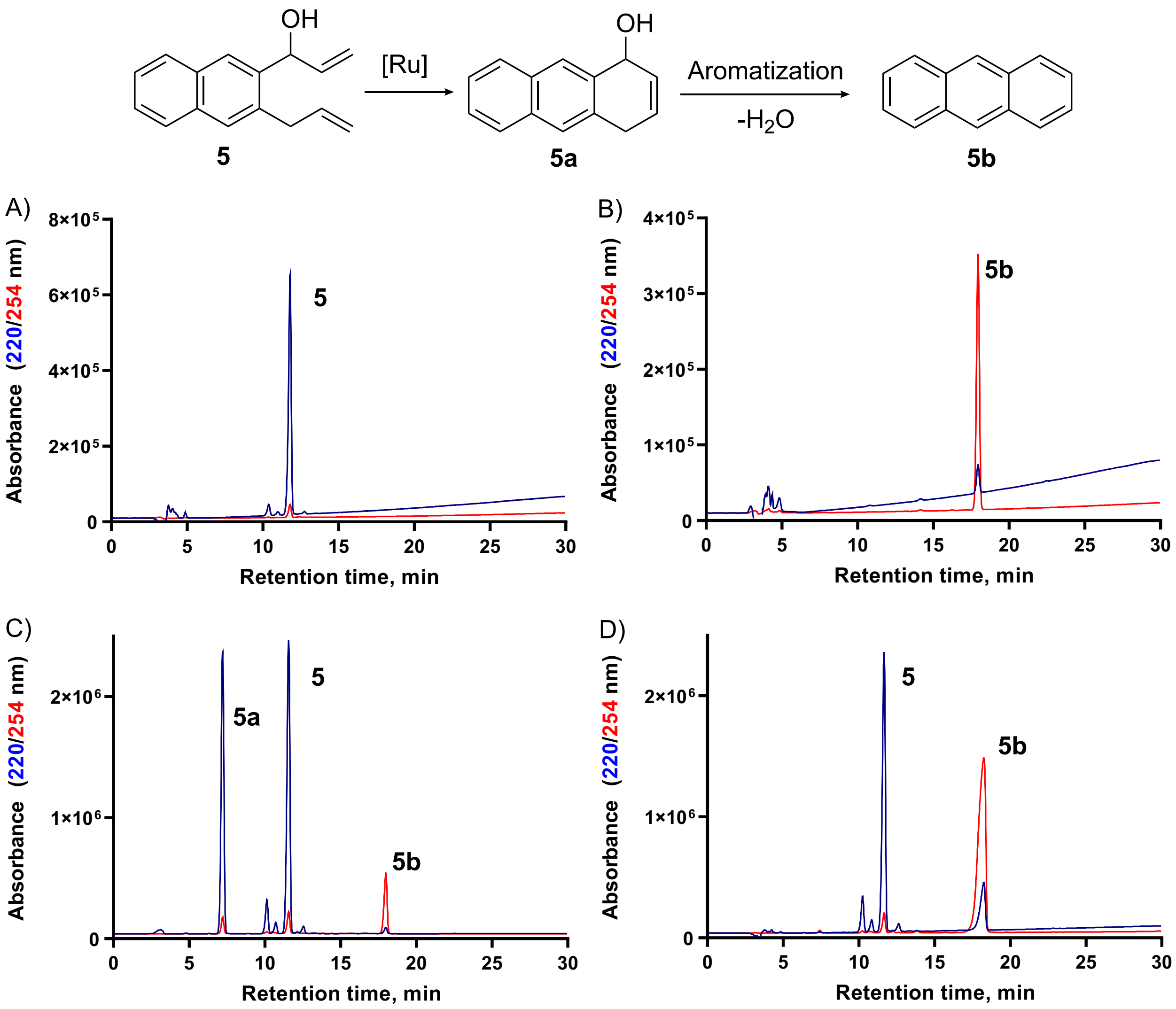

Supplementary Figure 19. Example HPLC traces of A) substrate **5**, B) product **5b**, C) reaction analysis was run with neutral solvent system (CH_3CN/H_2O), D) reaction analysis was run with acidic solvent system (0.1% TFA in CH_3CN/H_2O).

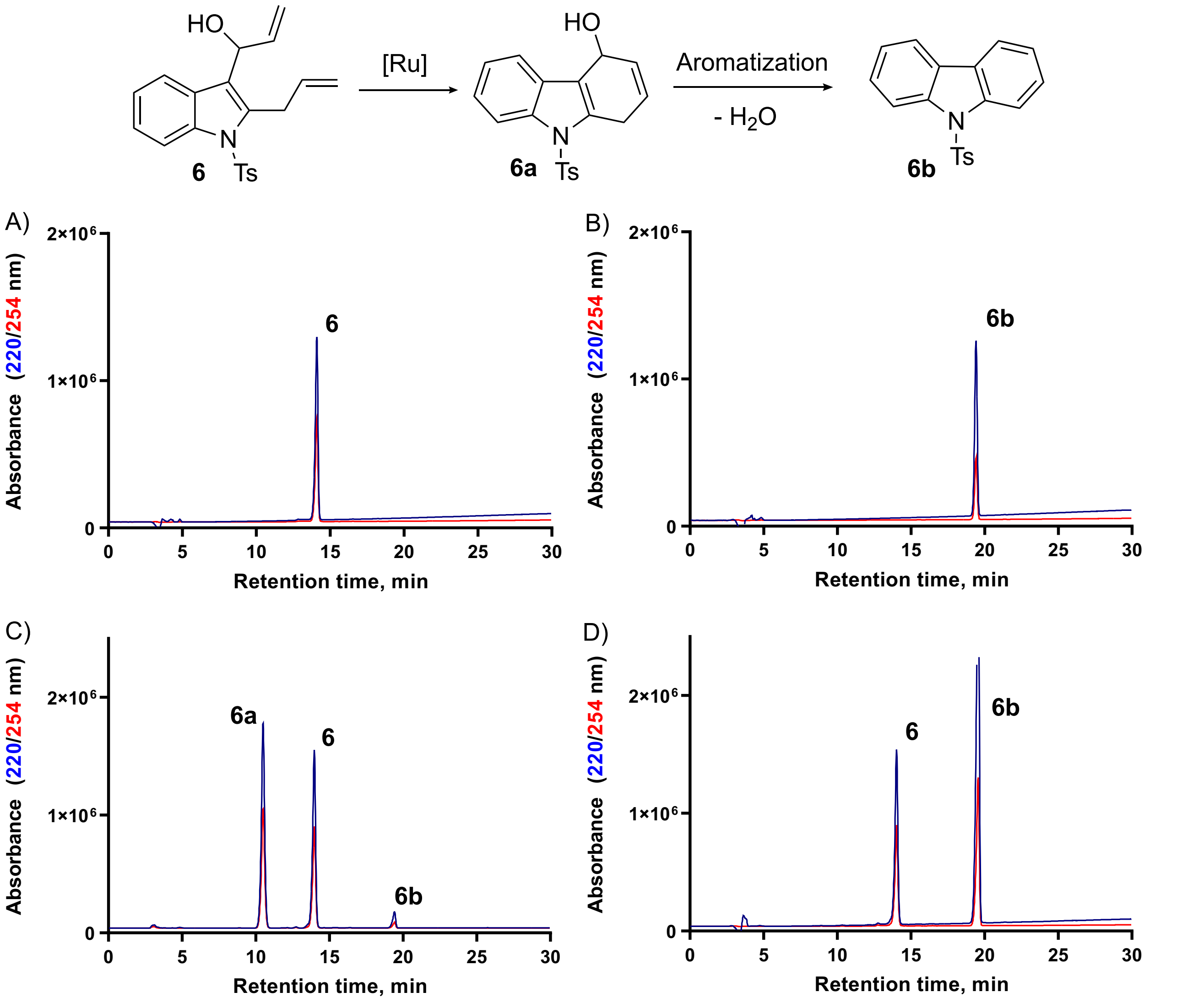

Supplementary Figure 20. Example HPLC traces of A) substrate **6**, B) product **6b**, C) reaction analysis was run with neutral solvent system (CH_3CN/H_2O), D) reaction analysis was run with acidic solvent system (0.1% TFA in CH_3CN/H_2O).

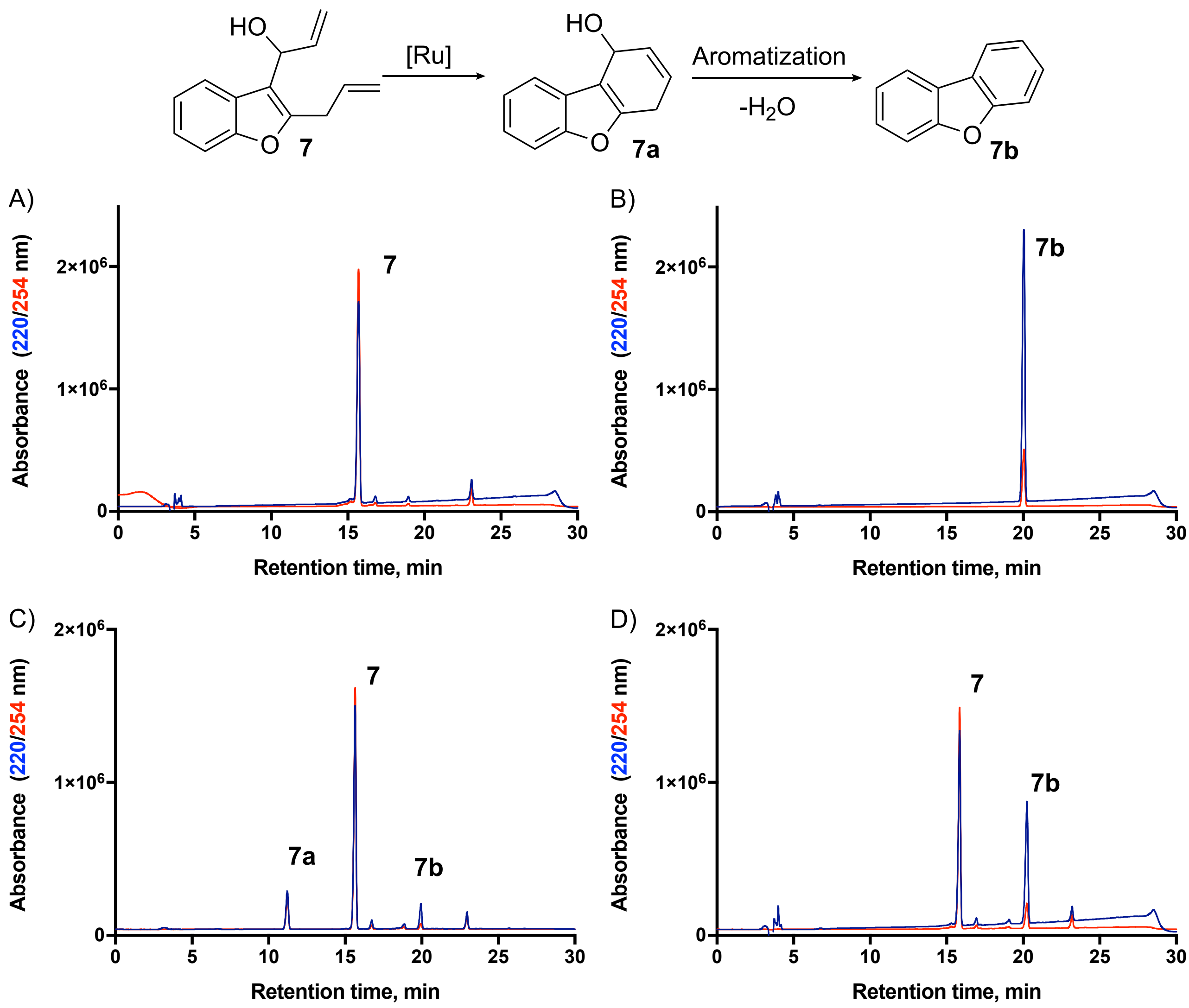

Supplementary Figure 21. Example HPLC traces of A) substrate **7**, B) product **7b**, C) reaction analysis was run with neutral solvent system (CH_3CN/H_2O), D) reaction analysis was run with acidic solvent system (0.1% TFA in CH_3CN/H_2O).

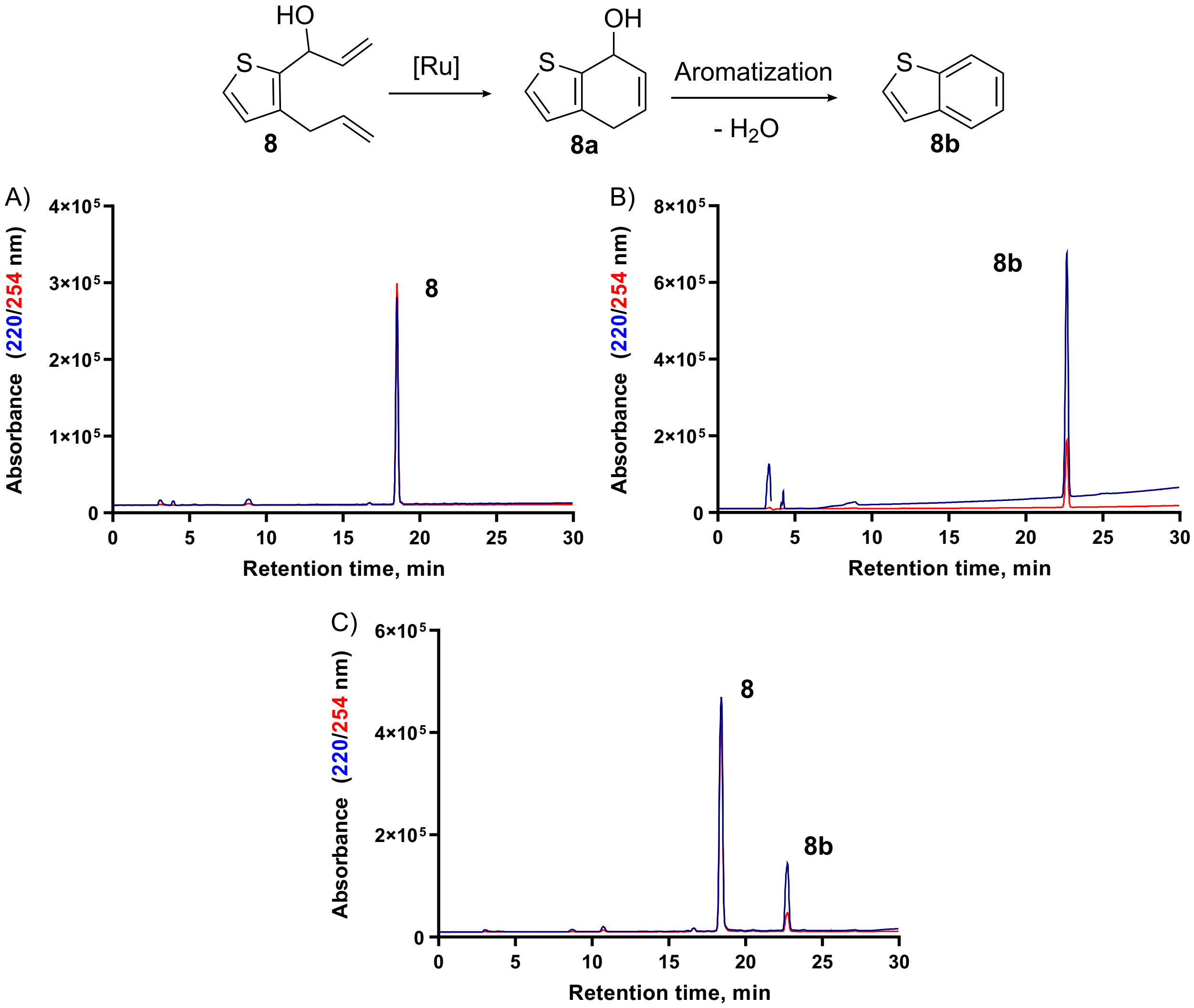

Supplementary Figure 22. Example HPLC traces of A) substrate **8**, B) product **8b**, C) reaction analysis was run with neutral solvent system (CH_3CN/H_2O),

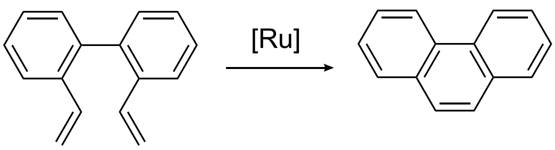

9 **9a**

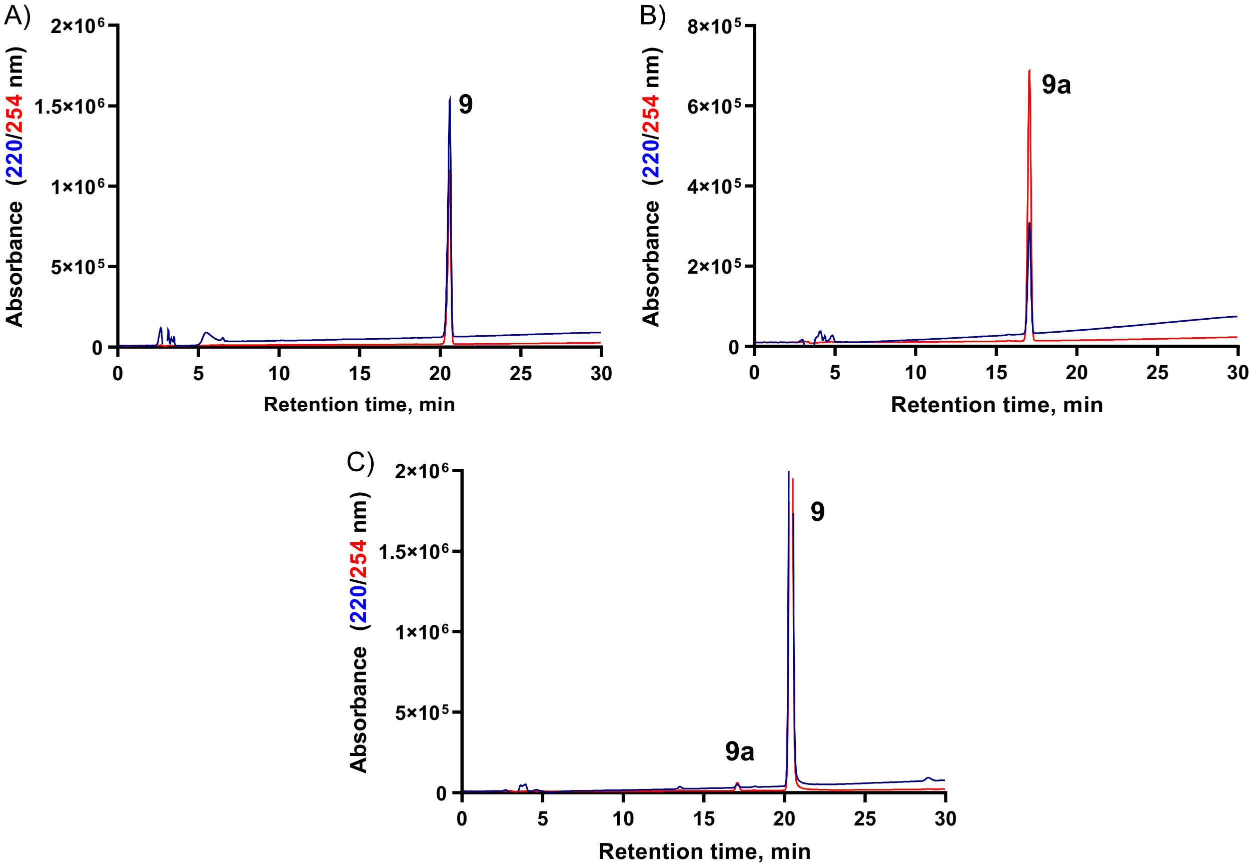

Supplementary Figure 23. Example HPLC traces of A) substrate **9**, B) product **9a**, C) reaction analysis was run with neutral solvent system (CH_3CN/H_2O).

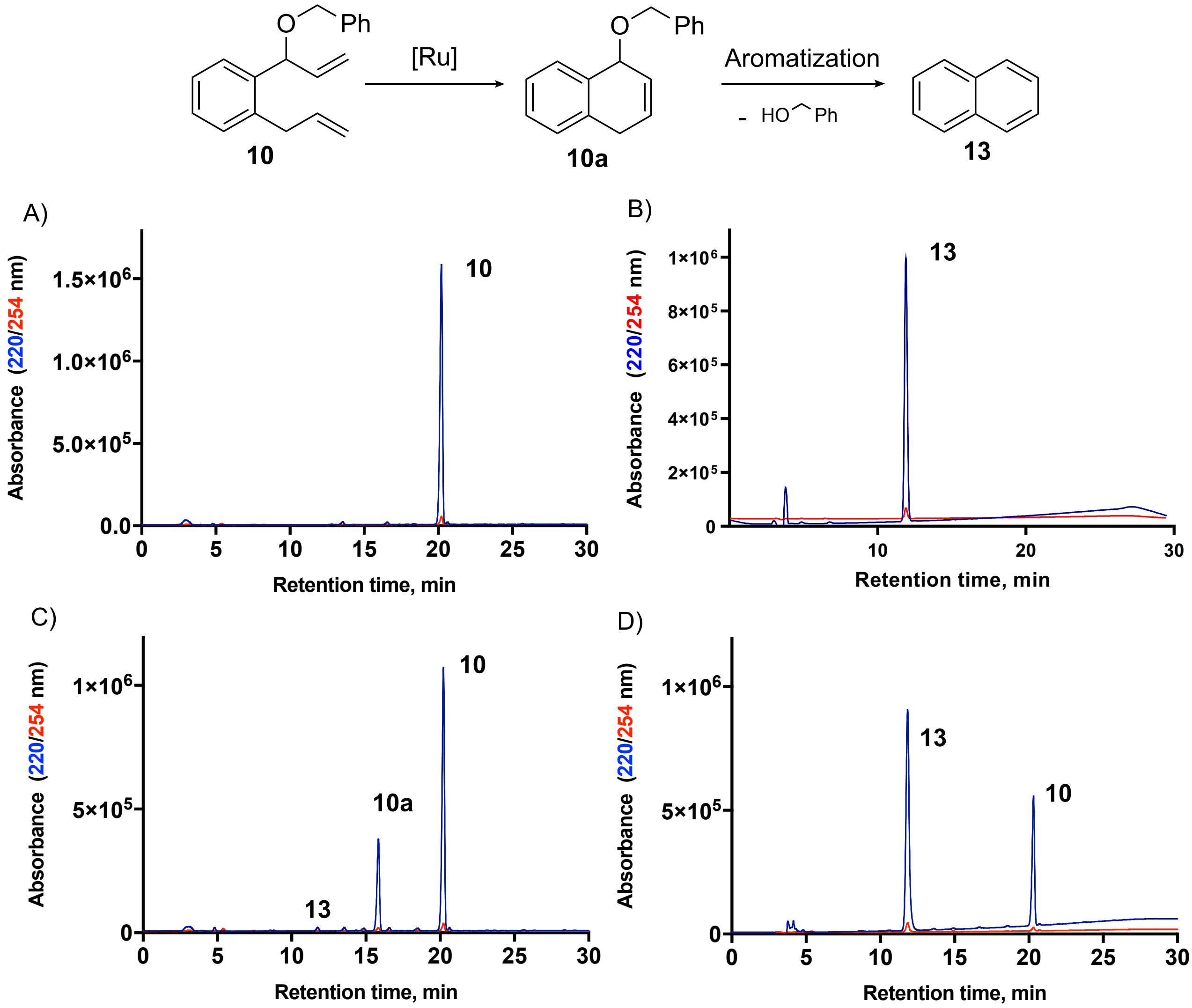

Supplementary Figure 24. Example HPLC traces of A) substrate **10**, B) product **13**, C) reaction analysis was run with neutral solvent system (CH_3CN/H_2O), D) reaction analysis was run with acidic solvent system (0.1% TFA in CH_3CN/H_2O).

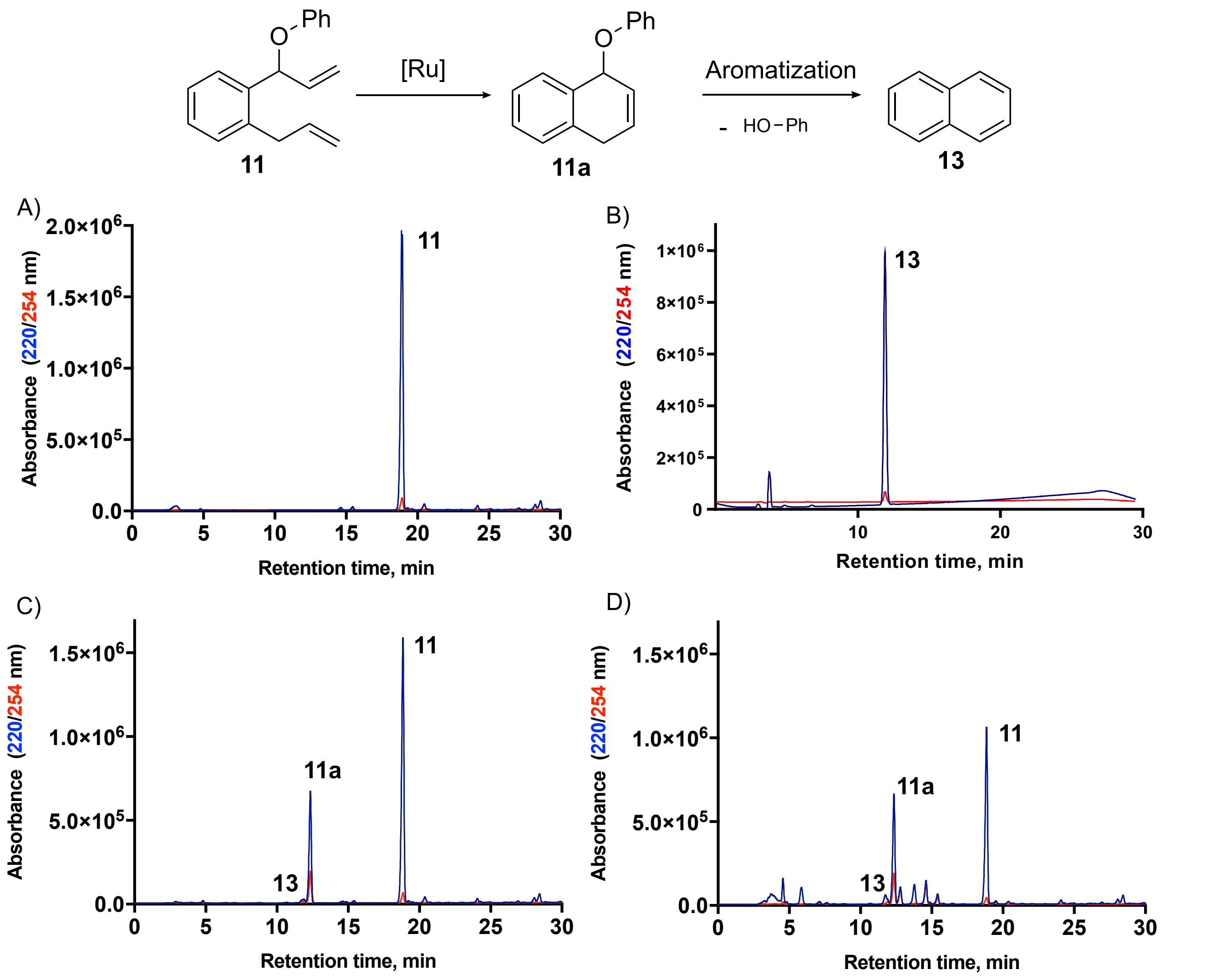

Supplementary Figure 25. Example HPLC traces of A) substrate **11**, B) product **13**, C) reaction analysis was run with neutral solvent system (CH_3CN/H_2O), D) reaction analysis was run with acidic solvent system (0.1% TFA in CH_3CN/H_2O).

Comment: reaction analysis with TFA leads to substrate **11** decomposition, meanwhile signal intensity of intermediate **11a** doesn't change. Thus, conversion of substrate **11** analyzed with neutral solvent system was used for TON_{RCM} calculation, instead of product **13** signal integration.

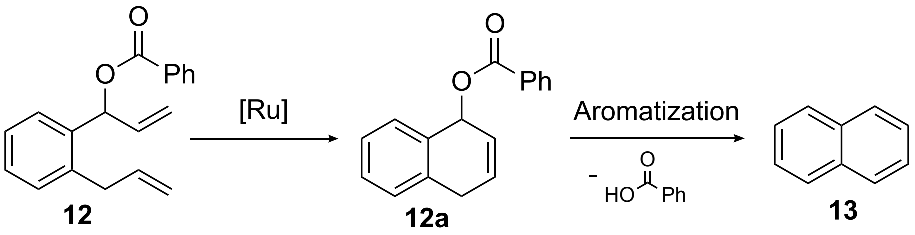

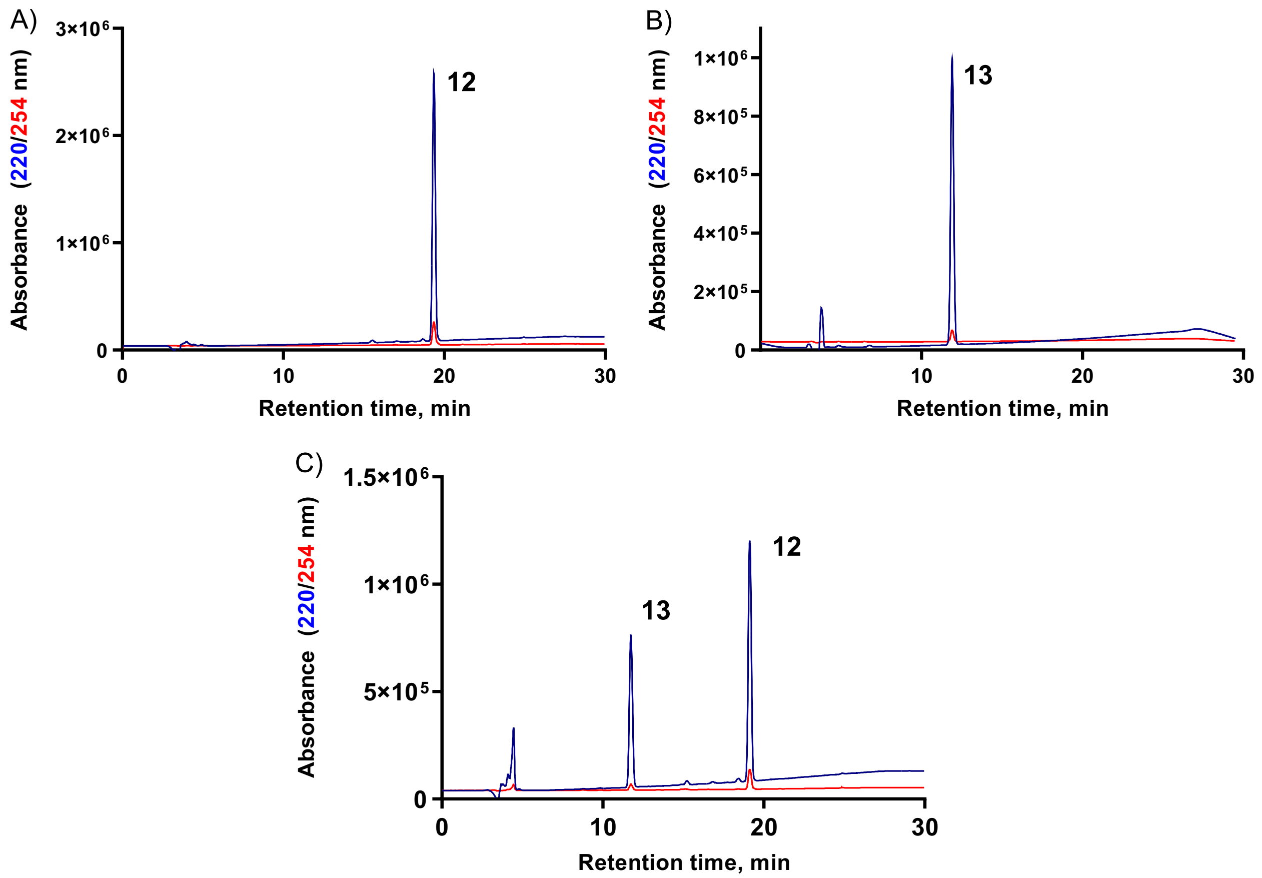

Supplementary Figure 26. Example HPLC traces of A) substrate **12**, B) product **13**, C) reaction analysis was run with neutral solvent system (CH_3CN/H_2O).

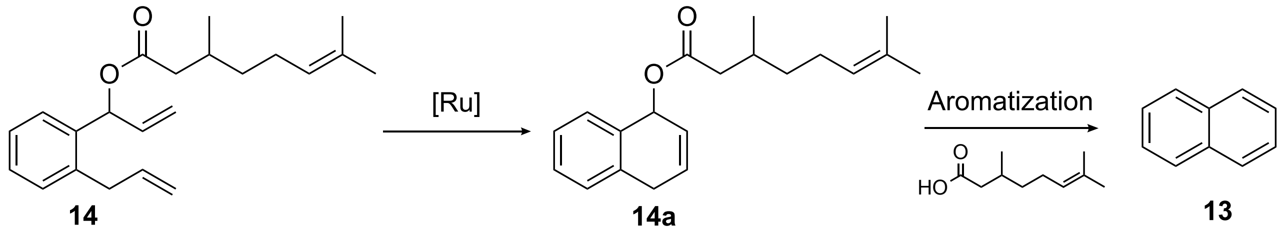

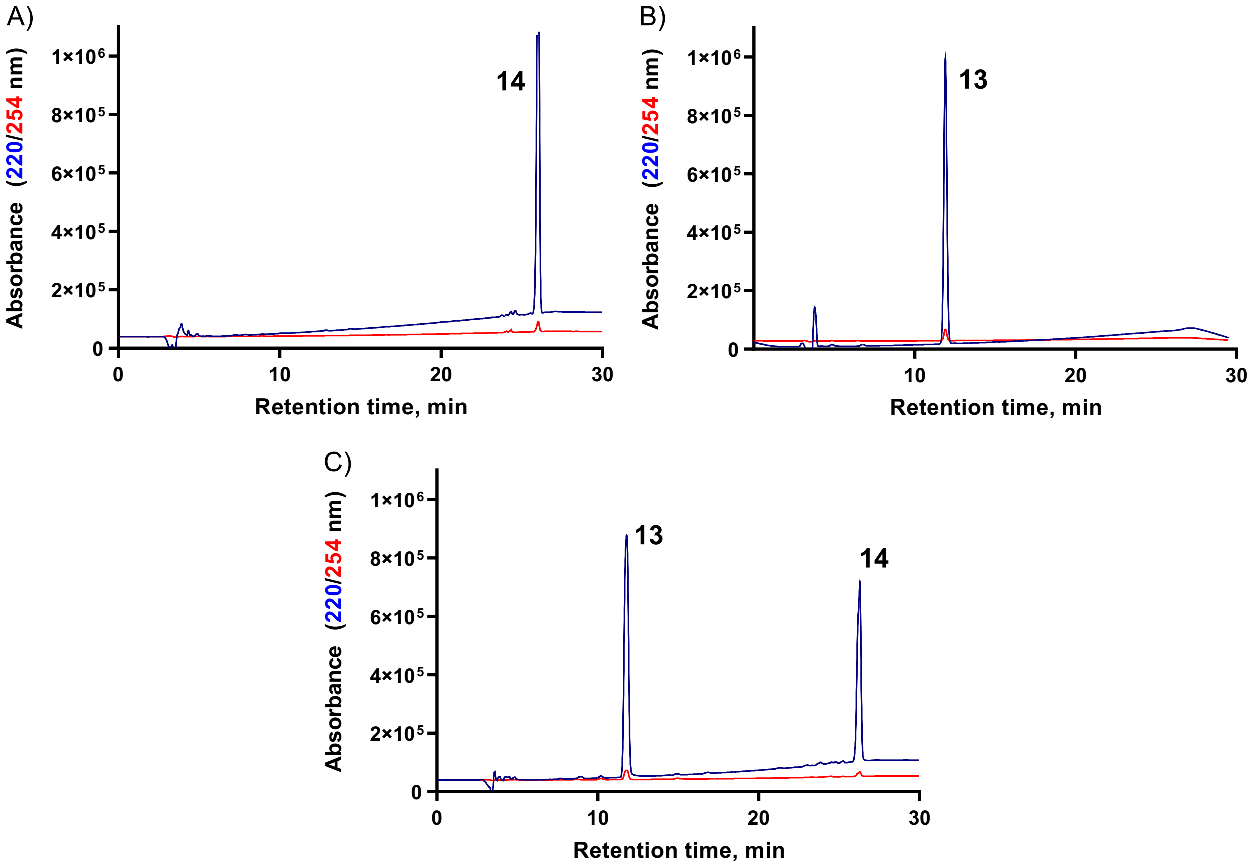

Supplementary Figure 27. Example HPLC traces of A) substrate **14**, B) product **13**, C) reaction analysis was run with neutral solvent system (CH_3CN/H_2O).

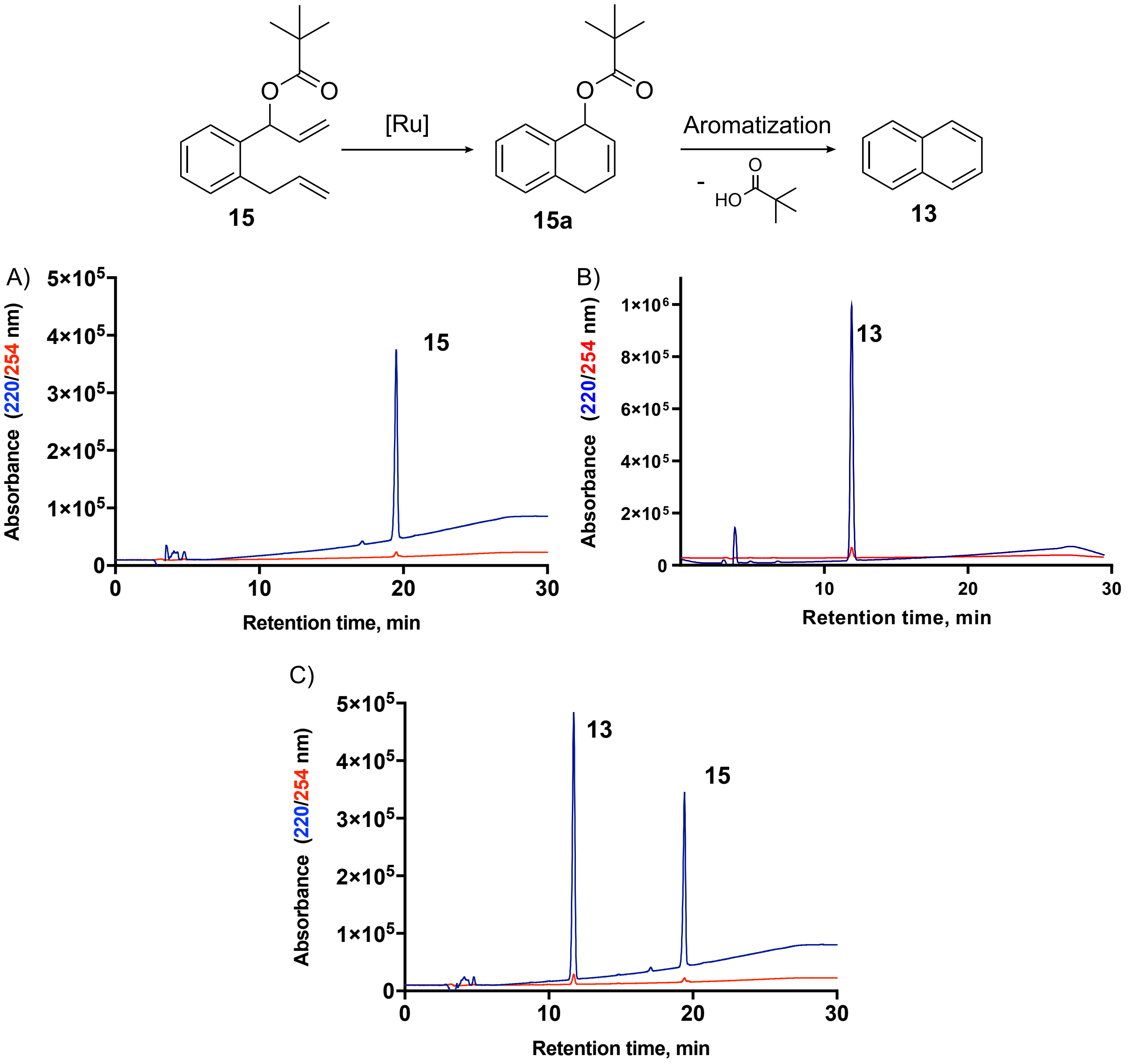

Supplementary Figure 28. Example HPLC traces of A) substrate **15**, B) product **13**, C) reaction analysis was run with neutral solvent system (CH_3CN/H_2O).

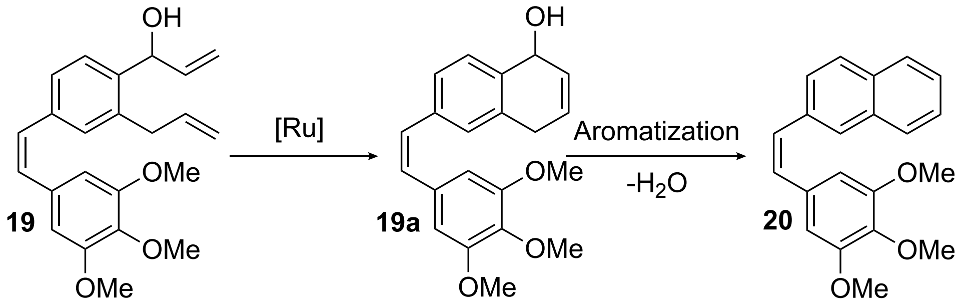

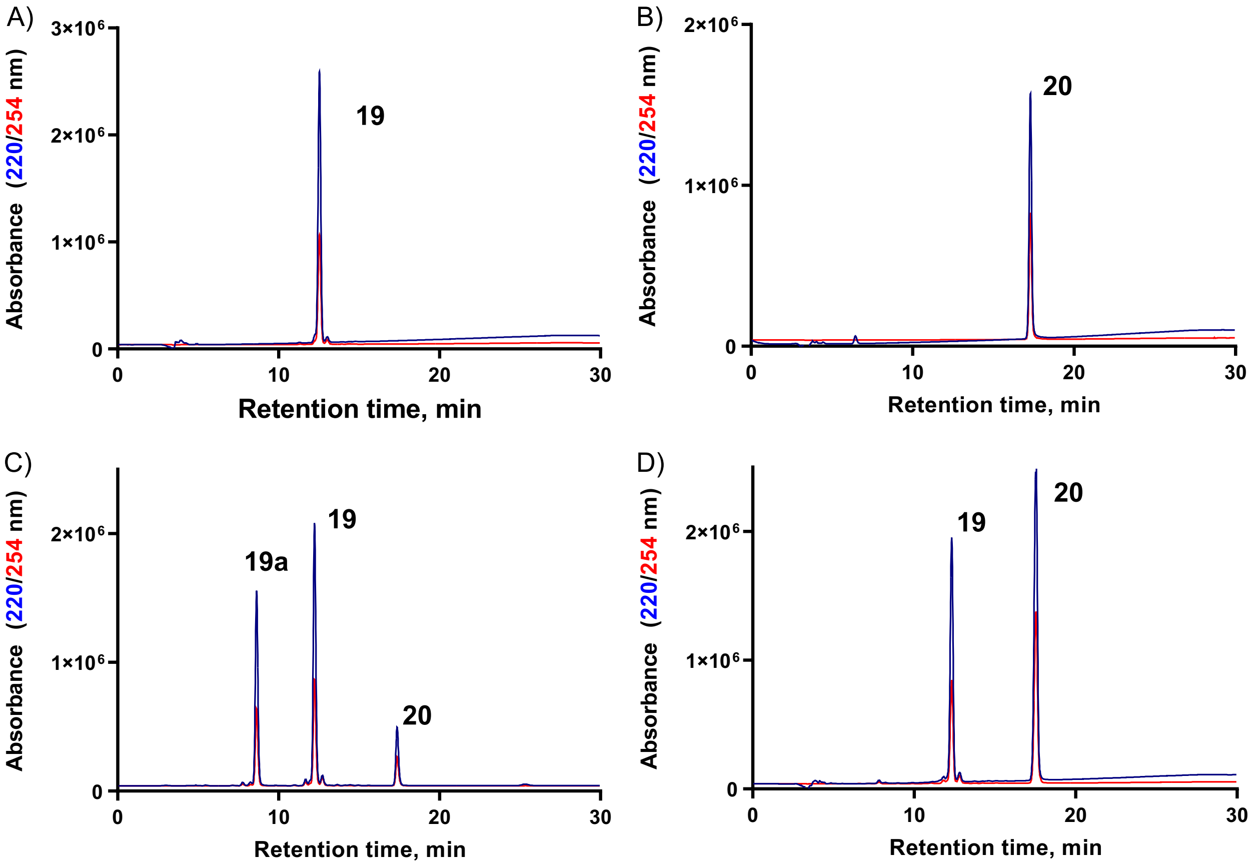

Supplementary Figure 29. Example HPLC traces of A) substrate **19**, B) product **20**, C) reaction analysis was run with neutral solvent system (CH_3CN/H_2O), D) reaction analysis was run with acidic solvent system (0.1% TFA in CH_3CN/H_2O).

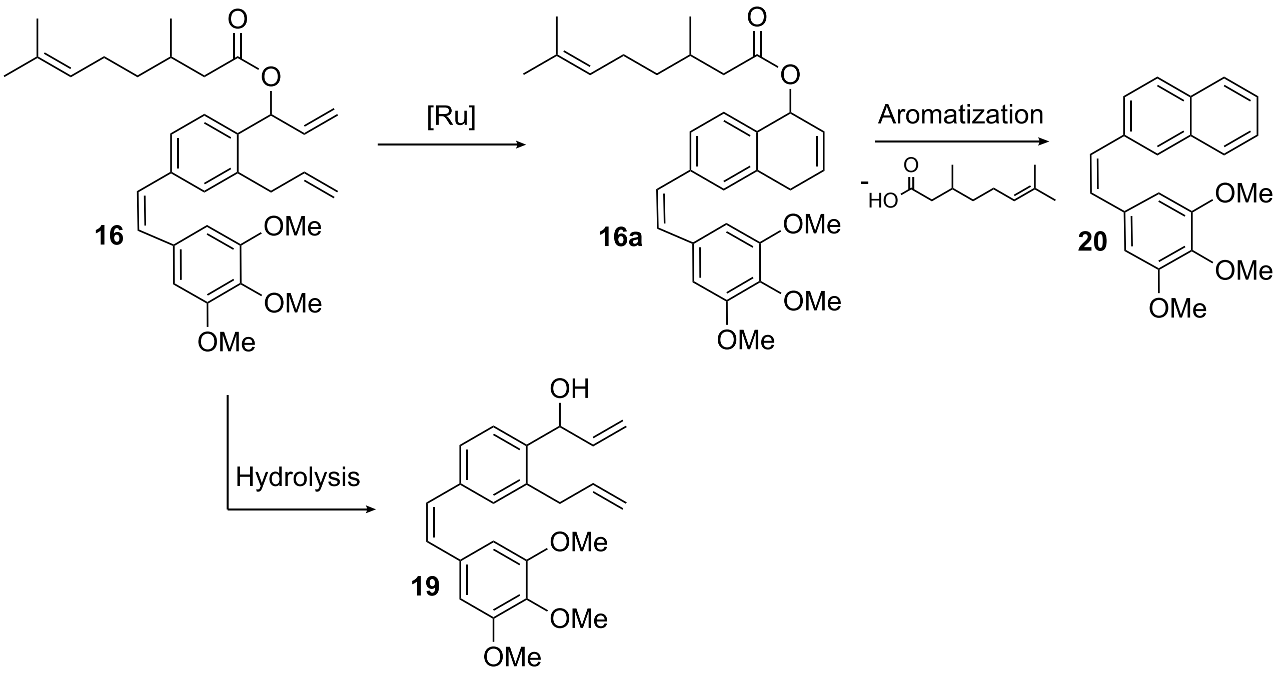

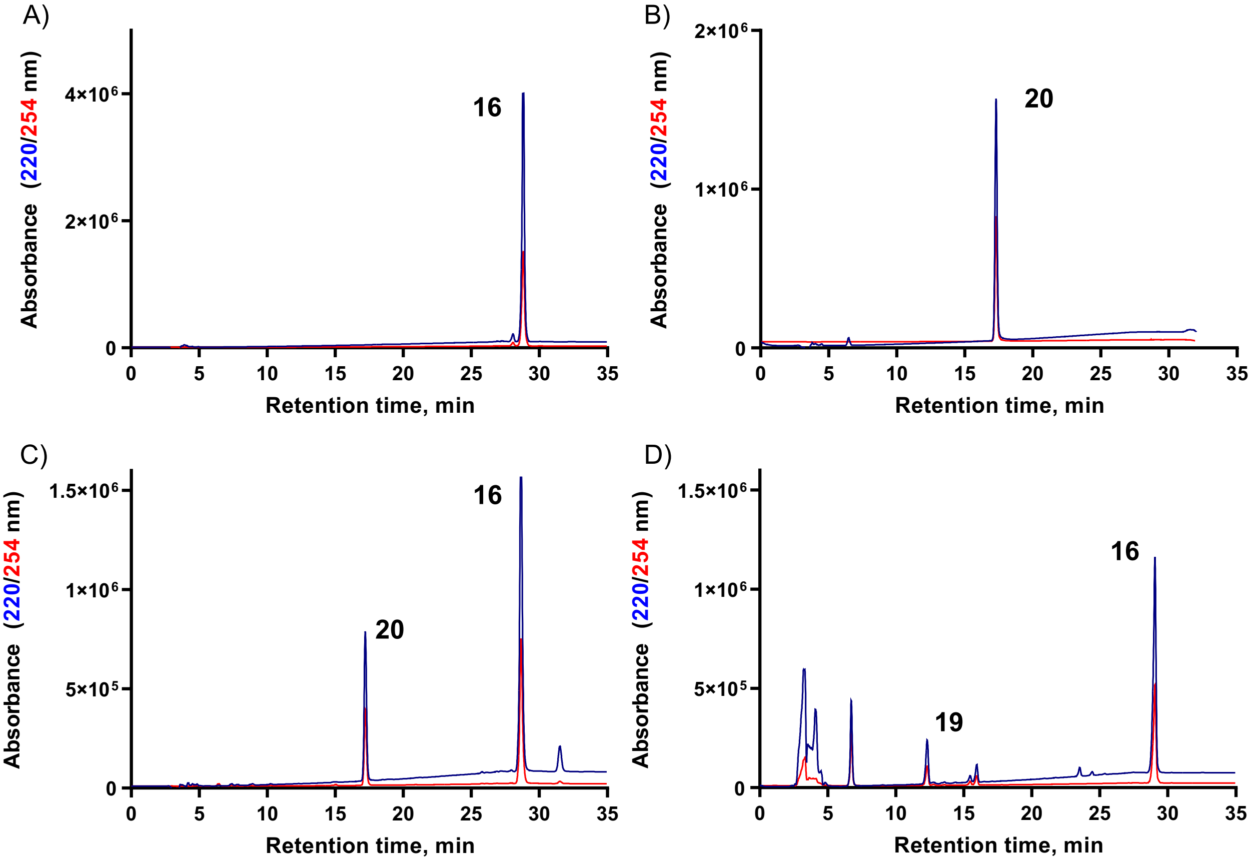

Supplementary Figure 30. Example HPLC traces of A) substrate **16**, B) product **20**, C) reaction analysis was run with neutral solvent system (CH_3CN/H_2O); D) substrate **16** stability test in blood/PBS/1,4-dioxane solution (5:4:1)

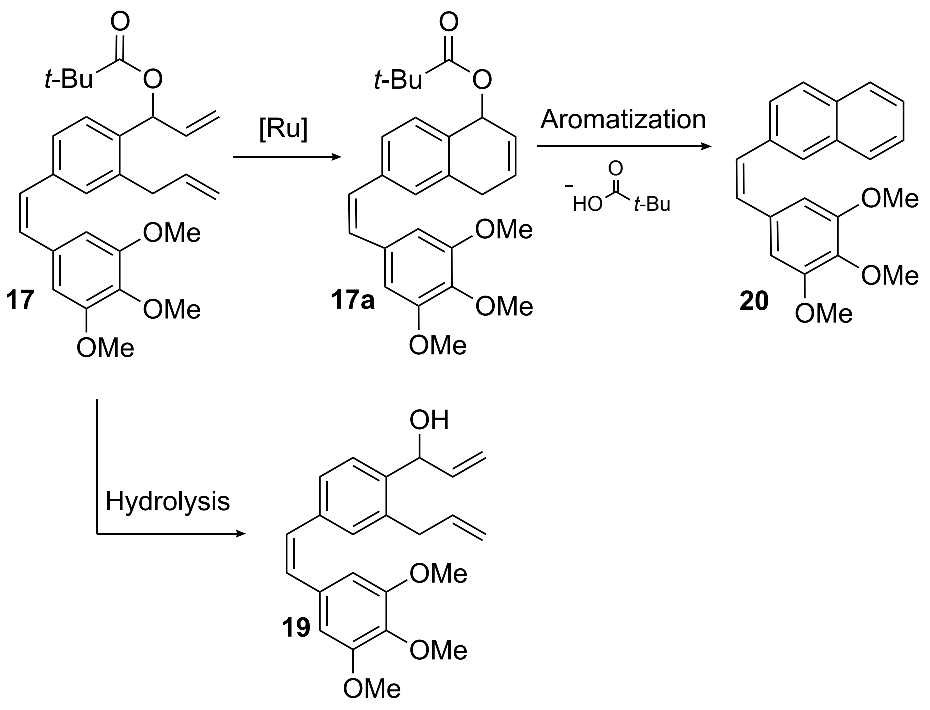

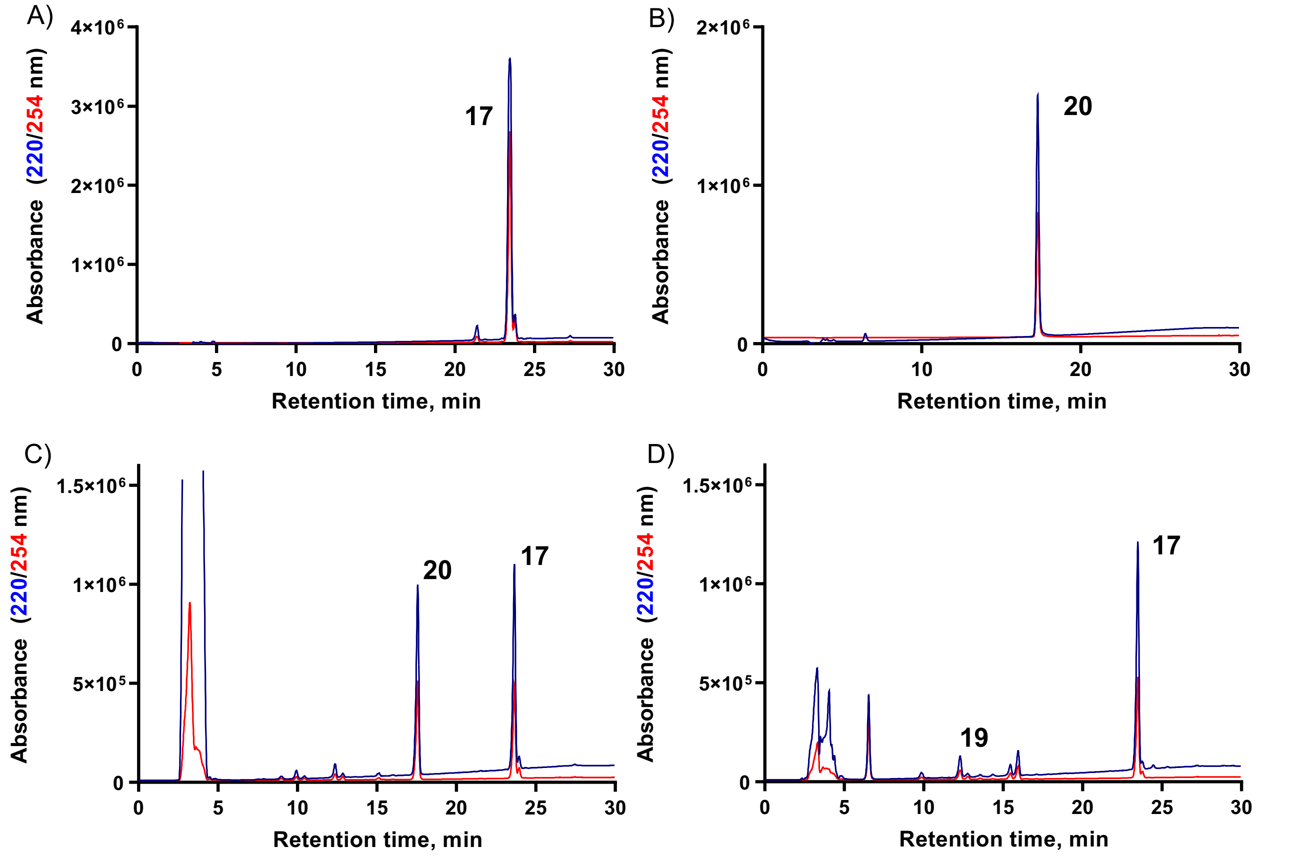

Supplementary Figure 31. Example HPLC traces of A) substrate **17**, B) product **20**, C) reaction analysis was run with neutral solvent system (CH_3CN/H_2O); D) substrate **17** stability test in blood/PBS/1,4-dioxane solution (5:4:1)

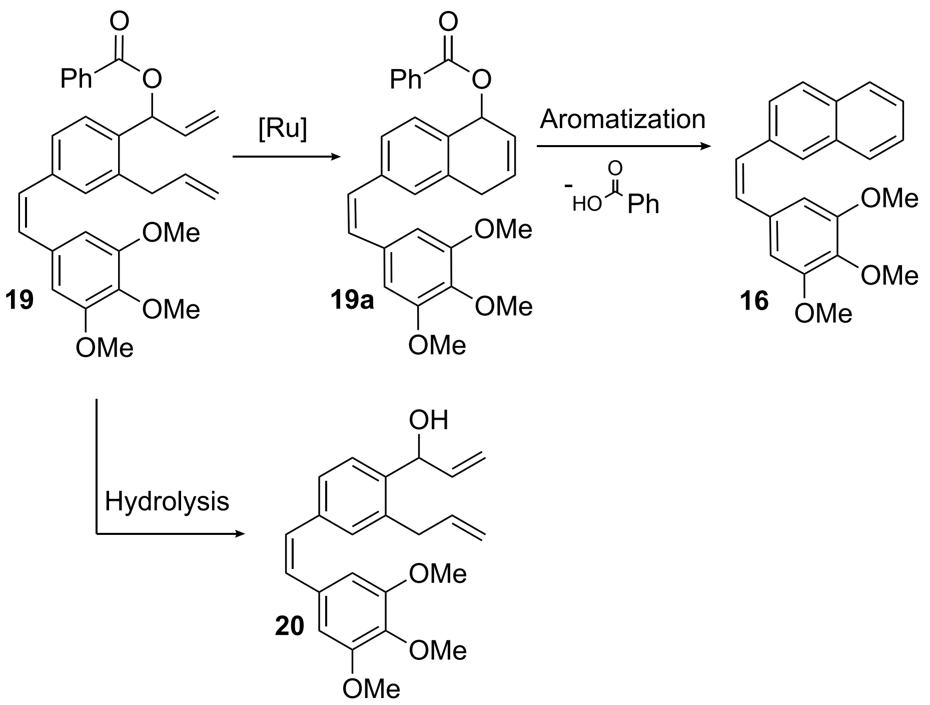

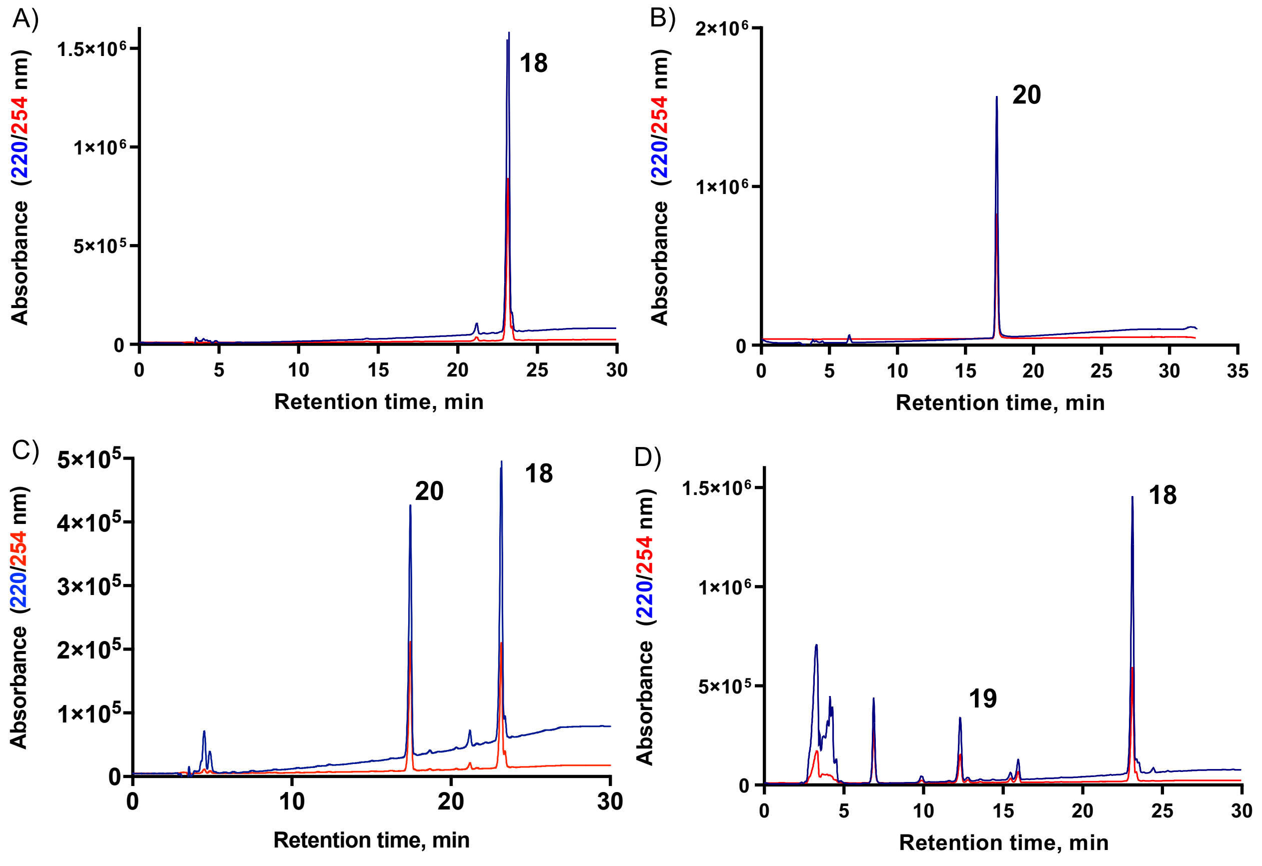

Supplementary Figure 32. Example HPLC traces of A) substrate **18**, B) product **20**, C) reaction analysis was run with neutral solvent system (CH_3CN/H_2O); D) substrate **18** stability test in blood/PBS/1,4-dioxane solution (5:4:1)

1.3. Modeling Studies

Supplementary Table 2: Calculated binding energies (kcal/mol) of various ligands to tubulin

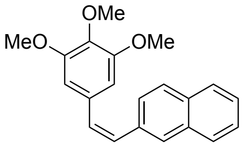

20

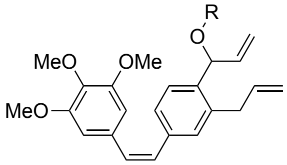

R:

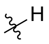

19

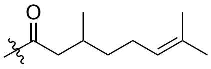

16

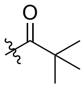

17

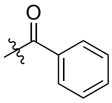

18

Docking Run	**20**	**19** (R)	**19** (S)	**18** (R)	**18** (S)	**17** (R)	**17** (S)	**16** (R)	**16** (S)
1	-9.24	-7.56	-5.56	-6.68	-5.94	-7.57	-7.36	-4.55	23.96
2	-9.23	-9.78	-9.52	-5.76	-6.32	-7.99	-7.57	0.26	3.52
3	-9.25	-8.75	-9.31	-6.20	-6.35	-8.15	-3.49	-2.59	-0.76
4	-9.27	-10.38	-8.65	-8.51	-7.69	-2.43	-6.23	-7.42	-8.62
5	-9.20	-6.86	-6.77	-3.25	-5.34	-7.06	-7.86	-9.04	-7.93
6	-9.19	-10.19	-8.97	-7.66	-8.41	-4.71	-6.36	-6.43	-4.35
7	-9.26	-10.07	-6.26	-3.70	-8.26	-5.11	-6.75	1.06	-2.26
8	-9.27	-10.04	-9.52	-6.68	-7.01	-4.60	-3.18	2.38	14.05
9	-9.26	-8.13	-6.94	-8.52	-7.88	-7.85	-6.61	1.75	-4.32
10	-9.26	-10.39	-7.01	-8.02	-7.93	-5.4	-6.94	-5.57	-6.12
avg. binding energy of each isomer	**-9.24**	**-9.22**	**-7.85**	**-6.50**	**-7.11**	**-6.09**	**-6.24**	**-3.02**	**0.72**
# of discreet conform.	1	3	6	7	7	7	5	8	8
avg. binding energy	**-9.24**	**-8.53**		**-6.81**		**-6.16**		**-1.15**	

1.4. Activity studies

1.4.1. Substrate scope investigation

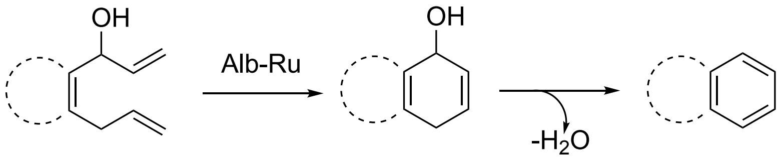

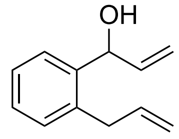

2, TON_{Arom}: 6.1±0.2
$TON_{RCM:}$ 38.6±0.1)

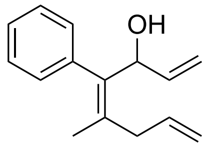

3, TON_{Arom}: 12.7±0.8
TON_{RCM}: 12.7±0.8

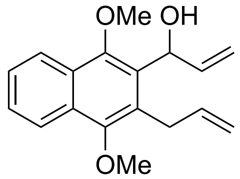

4, TON_{Arom}: 5.0±0.1
TON_{RCM}: 49.9±4.5

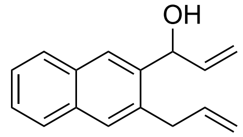

5, TON_{Arom}: 1.6±0.3
TON_{RCM}: 24.4±0.2

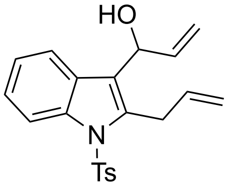

6, TON_{Arom}: 2.2±0.2
TON_{RCM}: 58.0±5.6

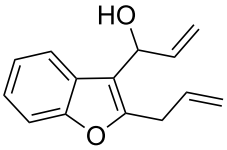

7, TON_{Arom}: 2.5±0.1
TON_{RCM}: 15.5±0.5

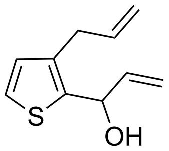

8, TON_{Arom}: 4.0±0.3
TON_{RCM}: 4.0±0.3

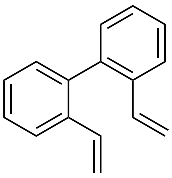

9, TON: 2.0±0.2

Supplementary Figure 33. All reactions were carried out under the standardized conditions: 4 mM of substrate was incubated with 1 mol% of Alb-Ru in PBS/1,4-dioxane (9:1) for 2 hours at 37°C. TON values were determined from product yields obtained by HPLC analysis; where TON_{Arom} refers to final product and TON_{RCM} refers to the combination of cyclohexadien-1-ol intermediate/final product. Reactions were performed in triplicate.

1.4.2. Michaelis–Menten kinetic experiments

Supplementary Table 3. Michaelis-Menten parameters for substrates **2, 12, 14, 15**. Summarized values are the maximum velocity (V_{max}), substrate affinity (K_M), turnover frequency (k_{cat}), catalytic efficiency (k_{cat}/K_M), and coefficient of determination (R^2).

	V_{max} (μM/s)	K_M, (mM)	k_{cat} (s^{-1})	k_{cat}/K_M ($M^{-1}s^{-1}$)	R^2
2	N/A	N/A	N/A	N/A	N/A
12	2.759±0.13	0.199±0.035	0.276±0.01	1383.6	0.935
14	0.935±0.06	0.053±0.026	0.093±0.01	1763.9	0.489
15	3.123±0.20	0.442±0.079	0.300±0.02	786.9	0.932

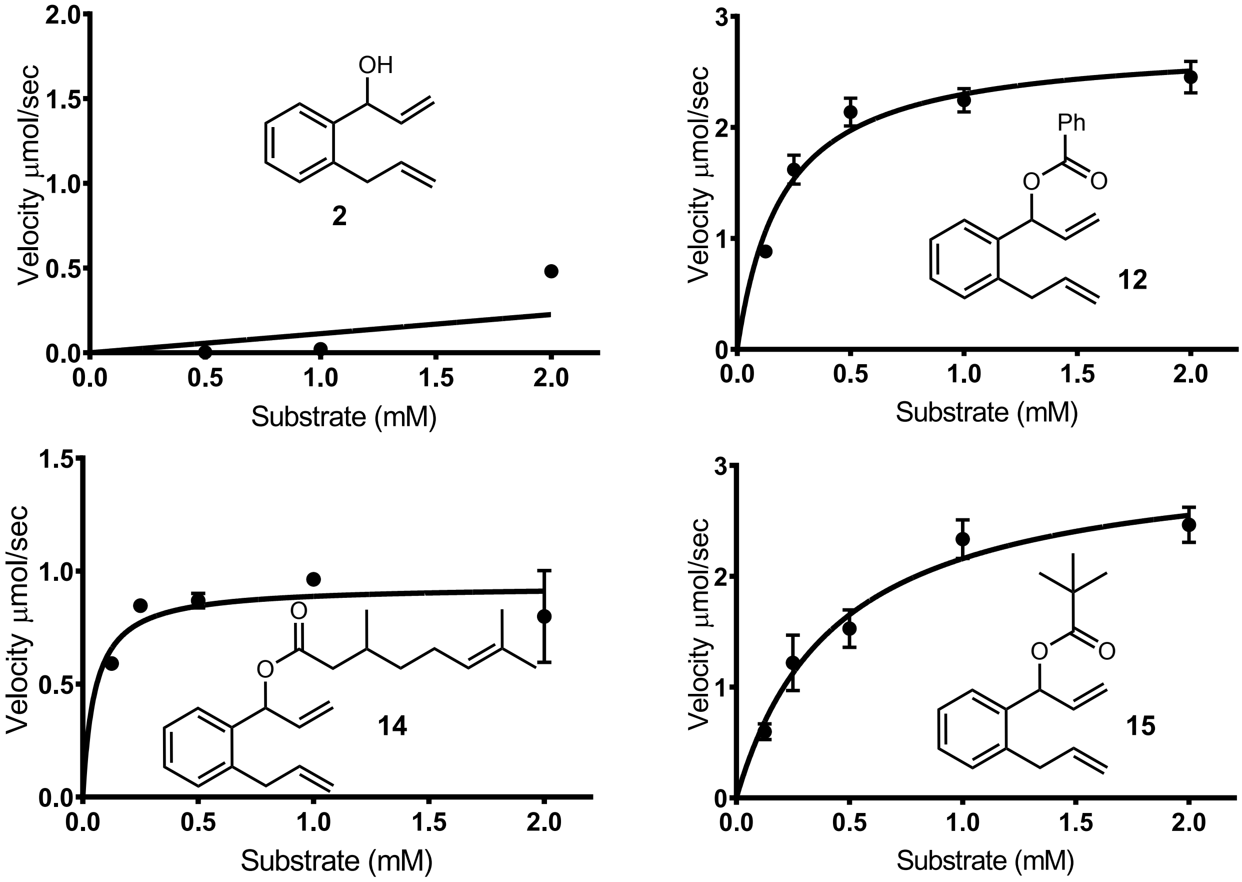

Supplementary Figure 34. Michaelis-Menten plots for substrates **2, 12, 14, 15**. Reactions were carried out in accordance with general procedure for kinetic experiments. Data are represented as mean value ± SD, *n* = 3 independent experiments.

Supplementary Table 4. Michaelis-Menten parameters for substrates **16-18**. Summarized values are the maximum velocity (V_{max}), substrate affinity (K_M), turnover frequency (k_{cat}), catalytic efficiency (k_{cat}/K_M), and coefficient of determination (R^2).

	V_{max} (μM/s)	K_M, (mM)	k_{cat} (s^{-1})	k_{cat}/K_M ($M^{-1}s^{-1}$)	R^2
16	0.112±0.007	0.134±0.032	0.011±0.001	84.1	0.810
17	0.684±0.051	0.150±0.047	0.068±0.005	457.9	0.785
18	0.046±0.006	0.043±0.033	0.0046±0.001	108.7	0.463

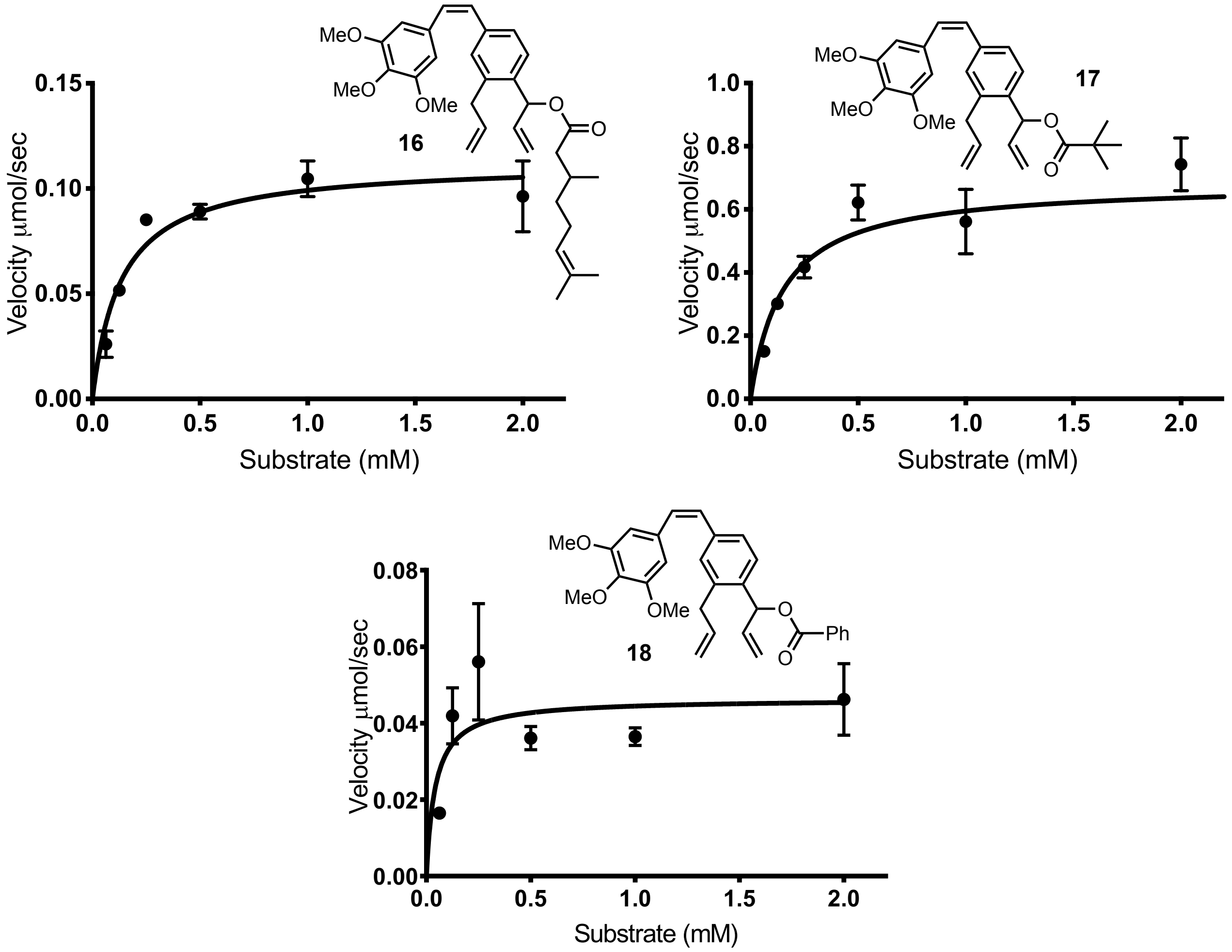

Supplementary Figure 35. Michaelis-Menten plots for substrates **16-18**. Reactions were carried out in accordance with general procedure for kinetic experiments. Data are represented as mean value ± SD, *n* = 3 independent experiments.

Supplementary Table 5. Ruthenium catalyst **1** activities using biological relevant concentrations of prodrugs **16-19.** Product yield was determined by HPLC analysis. Reactions were performed in triplicate.

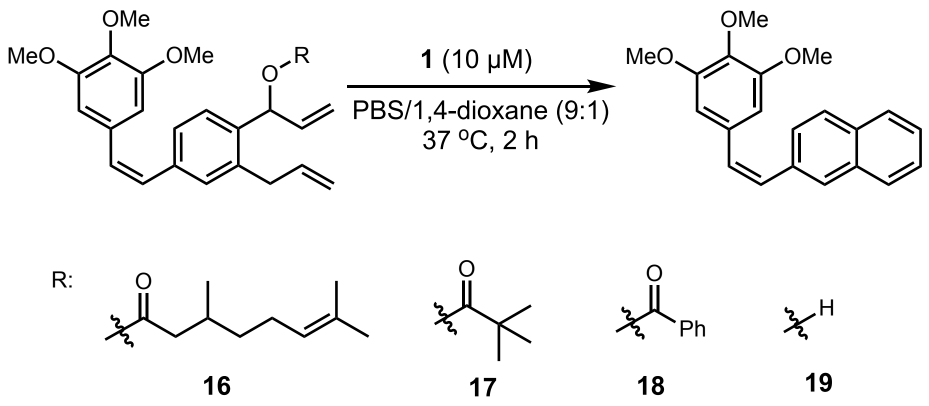

Concentration, μM	Product yield (%)			
	**19**	**16**	**17**	**18**
5	30.5±1.2	32.9±0.4	45.3±0.8	30.8±7.8
10	47.8±2.2	38.9±0.8	64.9±4.1	39.3±4.0
50	61.6±1.4	54.4±0.1	77.5±1.1	52.4±3.2
100	66.2±1.3	62.8±0.1	84.3±2.1	54.9±2.7
250	63.8±1.3	48.1±2.6	80.9±1.6	53.2±1.2

1.5. Biological experiments

Supplementary Figure 36. Dose response curves of napthylcombretastatin **20** drug (red line) and prodrug **17** (black line) for various cancer cell lines. Data are represented as mean value ± SD, *n* = 3 biologically independent samples.

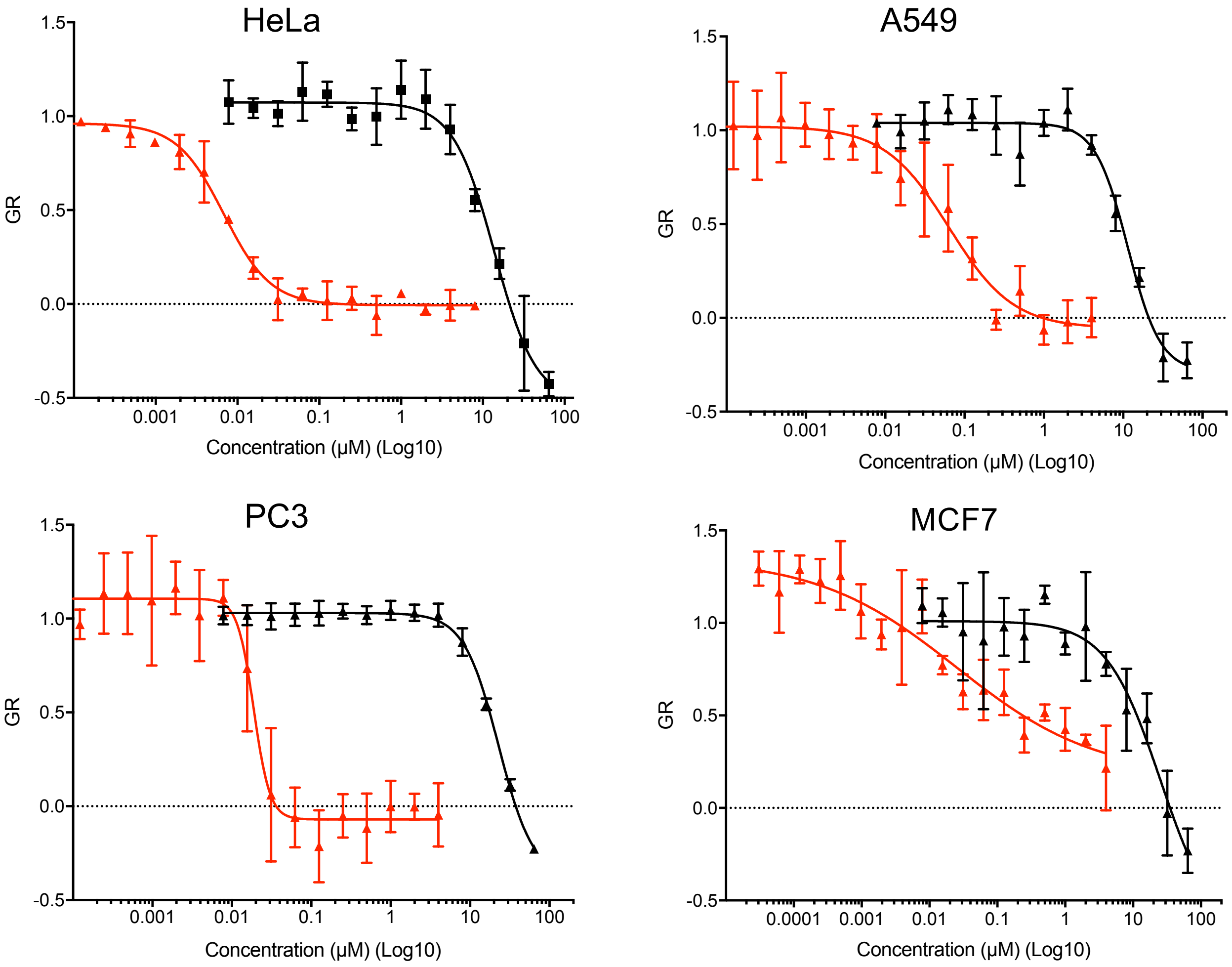

Supplementary Figure 37. Dose response curves of prodrugs **16, 17, 19** for HeLa cancer cell lines. Data are represented as mean value ± SD, *n* = 3 biologically independent samples.

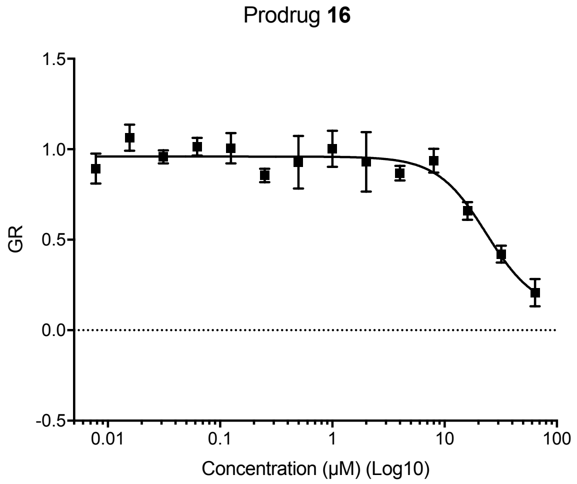

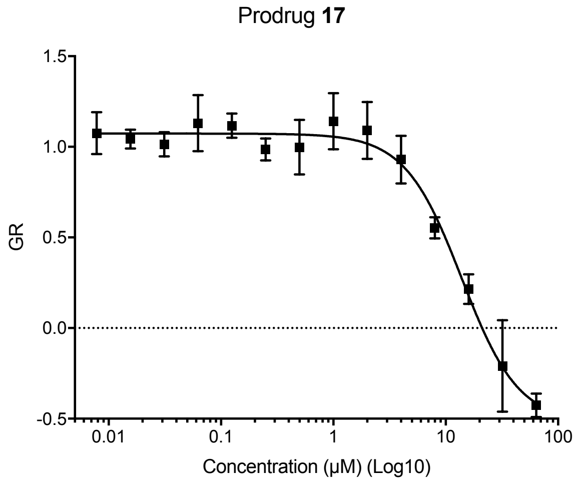

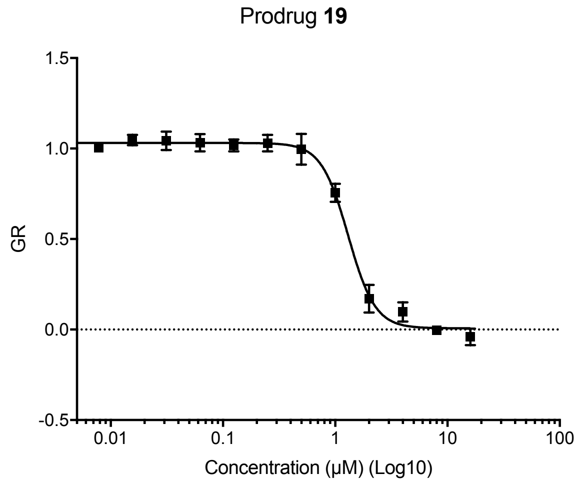

Supplementary Table 6: Calculated GR_{50} values represent concentrations that gives half maximal growth rate inhibition.

Substrate	**16**	**17**	**19**	**20**
GR_{50}, μM	25.0	10.1	1.34	0.003

Supplementary Figure 38. Dose response curves of prodrug **17** (blue line) and reaction mixture of **17** with 0.25 or 0.5 µM Alb-Ru (red line) for various cancer cell lines. Data are represented as mean value ± SD, *n* = 3 biologically independent samples.

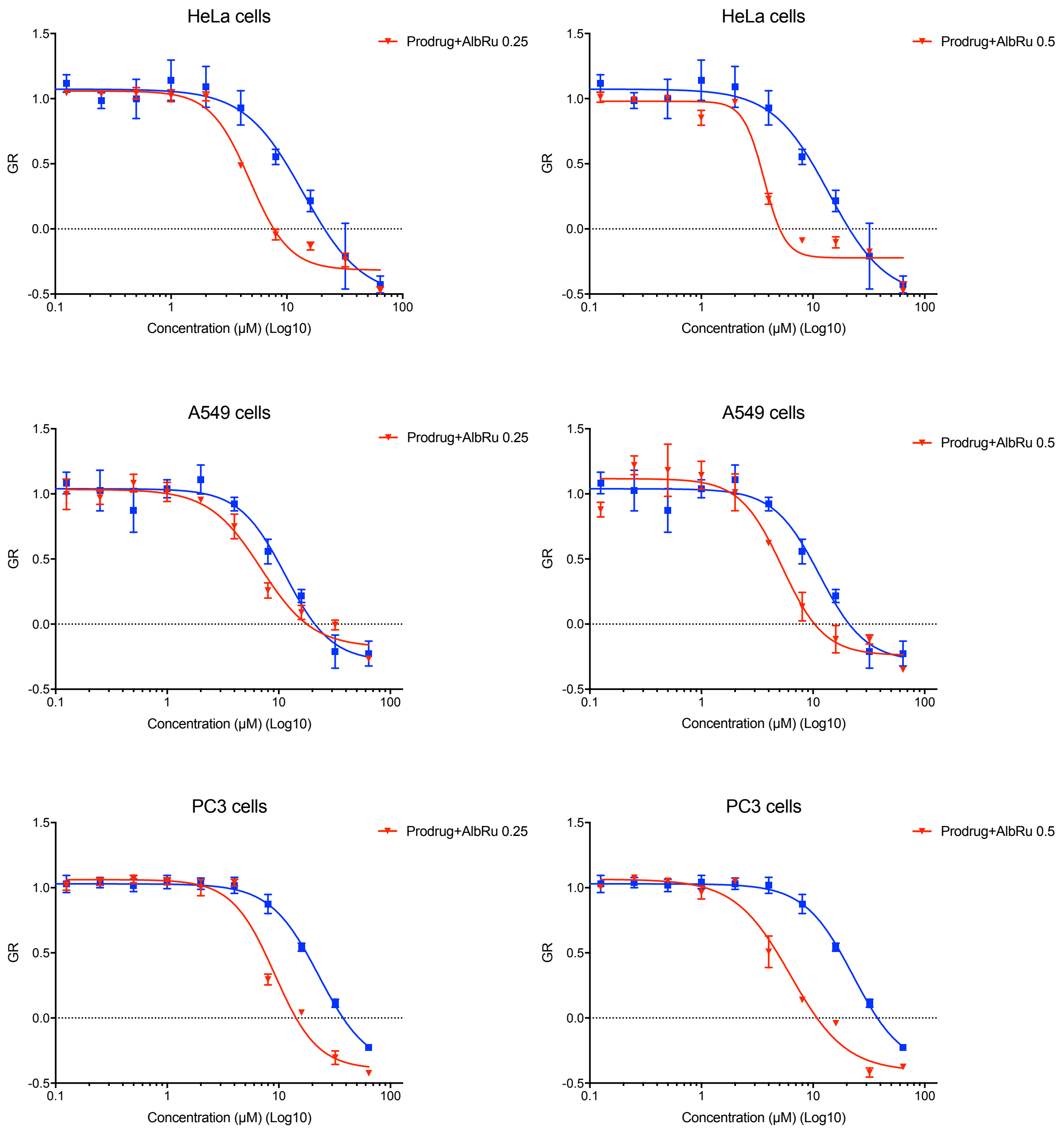

Supplementary Figure 39. Effects on cell growth following incubations with mixtures of Alb-Ru and prodrug **17**. The prodrug was kept with constant 4 μM concentration, with varying concentrations of Alb-Ru. Data are represented as mean value ± SD, *n* = 3 biologically independent samples.

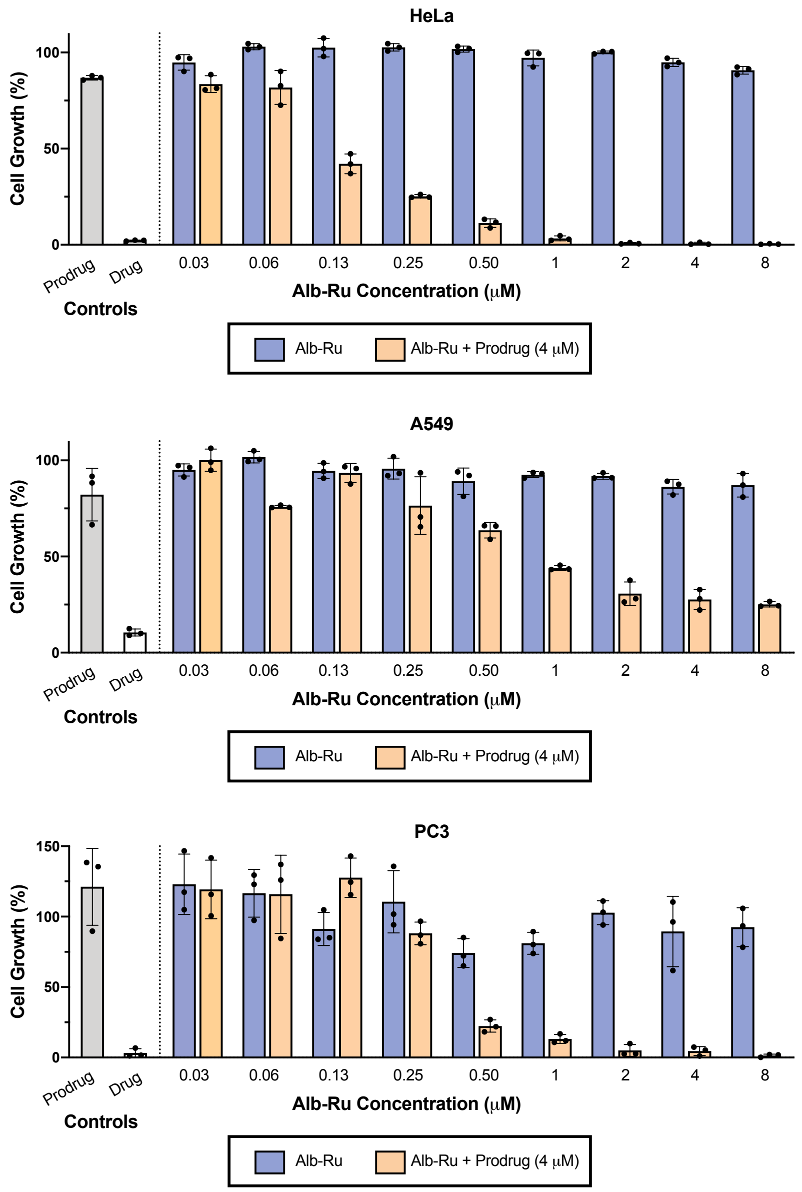

Supplementary Figure 40. Body weight (n = 5) change of various treatments group mice. Data are represented as mean value ± SD, *n* = 5 biologically independent samples.

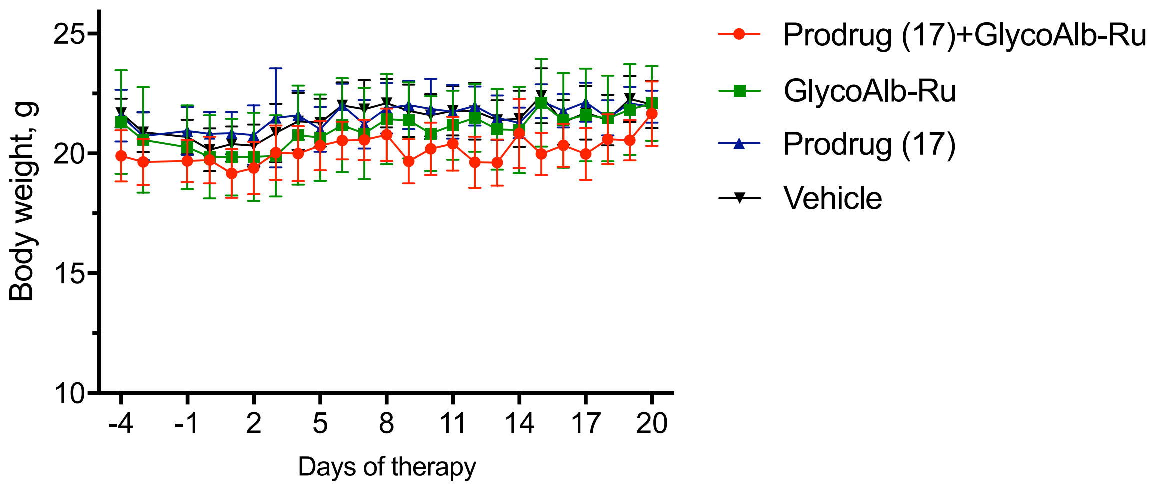

1.6. Synthesis of substrates

General method A. (Dimethoxyacetal protection).

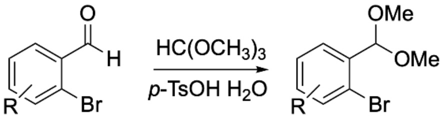

2-bromobenzaldehyde (1 eq.) was placed into the round bottom flask and suspended in 20 ml of anhydrous methanol, under N_2 atmosphere. Then, trimethyl orthoformate (10 eq.) and catalytic amount of *p*-TsOH·H_2O (1 mol%) were added, solution became clear and stirred for 4 hours at room temperature. After that time, reaction was quenched with $NaHCO_3$ sat. aq. solution by followed evaporation of methanol. The residue was redissolved in water/ethyl acetate mixture, aqueous layer was extracted three times with ethyl acetate. The combined organic extracts were washed with brine, dried over sodium sulfate, filtered and the filtrate evaporated to give the desired product. Product was used in next step without additional purification.

General method B. (Allylation).

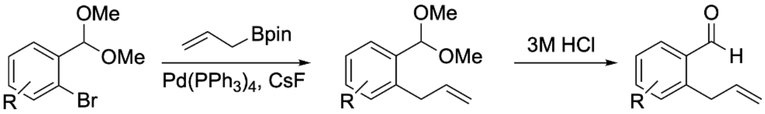

1) 1-bromo-2-(dimethoxymethyl)-benzene (1.0 eq), $Pd(PPh_3)_4$ (10 mol%), cesium fluoride (4.0 eq) and allylboronic acid pinacol ester (2.0 eq) were placed into 100 ml flask and suspended in 40 ml of anhydrous 1,4-dioxane (under N_2 atmosphere). The obtained solution was stirred at room temperature for 30 min and then heated up to reflux on oil bath for 20 hours. After cooling reaction mixture was diluted with ethyl acetate and filtered through a silica gel short pad. The filtrate solution was evaporated to dryness and purified by silica gel column chromatography (eluent Hexane/EtOAc – 9:1) to give allylated product as yellowish oil. 2) For dimethyl acetal deprotection, 1-allyl-2-dimethoxymethyl-benzene was dissolved in 9 ml of THF and treated with 0.2 ml of 3M HCl. After 30 min stirring at room temperature, reaction mixture was quenched with saturated aq. $NaHCO_3$, extracted three times with ethyl acetate. The combined organic extracts were washed with brine, dried over sodium sulfate, filtered and the filtrate evaporated to give 2-allylbenzaldehyde as yellowish oil.

General method C. (Grignard reaction).

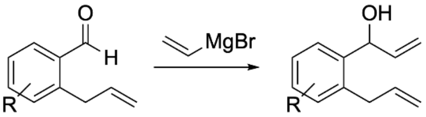

To a round bottom flask benzaldehyde was placed (1.0 eq.) and dissolved in anhydrous THF (10 ml), by followed cooling on ice bath. Then 1M THF vinyl magnesium bromide (1.5 eq.) solution was added dropwise and stirred for 2 hours. After that time, reaction was quenched with saturated aq. NH_4Cl solution and extracted three times with ethyl acetate. The combined organic extracts were washed with brine, dried over sodium sulfate, filtered and the filtrate evaporated. Crude product was purified by silica gel column chromatography (eluent hexane/EtOAc = 9:1 – 8:2 – 7:3).

General method D. (Esterification)

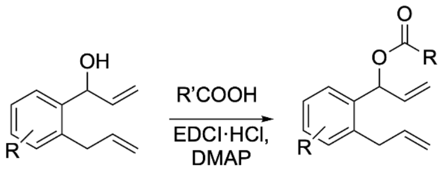

To the stirred solution of EDCI (2.0 eq.), DMAP (0.2 eq.) and carboxylic acid (1.3 eq.) in anhydrous CH_2Cl_2 (10 mL) was added dropwise solution of alcohol (1.0 eq.) in CH_2Cl_2 (2 mL). Solution was stirred at room temperature for 15 h, reaction progress was monitored by TLC. Then, reaction was quenched by water and extracted with CH_2Cl_2 (3x10 mL). The organic layers were combined, washed with brine, dried over Na_2SO_4 and evaporated to dryness. The crude product was purified by silica gel column chromatography.

The following compounds were synthesized according to the literature procedure: Ru-catalyst (**1**)[1], 1-(2-allylphenyl)prop-2-en-1-ol (**2**)[2], (Z)-5-methyl-4-phenylocta-1,4,7-trien-3-ol (**3**)[3], 3-bromo-4-formylbenzoic acid[4], 2-bromobenzofuran-3-carbaldehyde[5], 3-bromo-2-naphthaldehyde[6], triphenyl(3,4,5-trimethoxybenzyl)phosphonium bromide[7], methyl 1,4-dihydroxy-2-naphthoate[8], 2-allyl-1-tosyl-1*H*-indole-3-carbaldehyde[9], 1-(2-allylphenyl)-3-(trimethylsilyl)prop-2-yn-1-ol[10].

Methyl 3-bromo-4-(dimethoxymethyl)benzoate

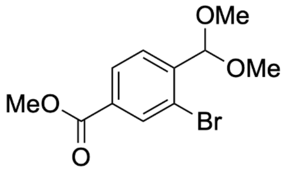

1) 3-bromo-4-formylbenzoic acid[4] (1429 mg, 6.24 mmol, 1.0 eq.) was suspended in 30 mL of anhydrous MeOH and cooled on ice bath. Acetyl chloride (2.7 mL, 37.4 mmol, 6.0 eq.) was added dropwise, after ice bath was removed. Obtained solution was stirred at room temperature for 20 hours, then quenched with $NaHCO_3$ sat. aq. solution by followed evaporation of methanol. The residue was redissolved in water/ethyl acetate mixture, aqueous layer was extracted two times with ethyl acetate. The combined organic extracts were washed with brine, dried over sodium sulfate, filtered and the filtrate evaporated. Crude product was purified by silica gel column chromatography (eluent Hexane/EtOAc – 8:2) to give inseparable mixture of aldehyde and dimethoxy acetal (1:4 ratio, 1.3 g). 2) Obtained mixture was placed into the round bottom flask and suspended in 20 mL of anhydrous methanol, under N_2 atmosphere. Then, trimethyl orthoformate (600 µl, 5.36 mmol, 4 eq.) and catalytic amount of *p*-TsOH·H_2O (13.3 mg, 0.07 mmol, 5 mol%) were added, solution became clear and stirred for 4 hours at room temperature. After that time, reaction was quenched with $NaHCO_3$ sat. aq. solution by followed evaporation of methanol. The residue was redissolved in water/ethyl acetate mixture, aqueous layer was extracted two times with ethyl acetate. The organic extracts were mixed and washed with brine, dried over sodium sulfate, filtered and the filtrate evaporated to give the desired product (1280 mg, 71 % over two steps). Product was used in next step without additional purification. ^{1}H-NMR (400 MHz, $CDCl_3$) δ (ppm) 8.23 (d, 1H, J = 1.6 Hz), 7.99 (dd, 1H, J_1 = 8.1 Hz, J_2 = 1.6 Hz), 7.69 (d, 1H, J = 8.1 Hz), 5.58 (s, 1H), 3.93 (s, 3H), 3.39 (s, 6H); ^{13}C (100 MHz, $CDCl_3$, δ) 165.6, 141.5, 134.1, 131.9, 128.5, 128.3, 122.9, 102.5, 54.0 (2C), 52.5;

2-Bromo-3-(dimethoxymethyl)-1-benzofuran

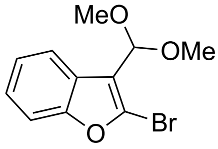

2-Bromo-3-(dimethoxymethyl)-1-benzofuran was synthesized according to the general procedure **A** using 2-bromobenzofuran-3-carbaldehyde[5] (934.5 mg, 4.15 mmol), trimethylorthoformate (4.54 mL, 41.53 mmol), *p*-toluenesulfonic acid monohydrate (7.9 mg, 41.5 µmol) in MeOH (40 mL) under reflux. The reaction mixture was purified by column flash chromatography on silica gel (EtOAc / hexane, gradient from 0 % to 7 % EtOAc) to give a protected aldehyde as a light-yellow oil (1.026 g, 91 %). ^{1}H-NMR (400 MHz, $CDCl_3$): δ (ppm) 7.83 – 7.79 (m, 1H), 7.45 – 7.41, 7.30 – 7.21 (m, 3H), 5.50 (s, 1H), 3.43 (s, 6H). ^{13}C-NMR (100 MHz, $CDCl_3$): δ (ppm) 155.5, 128.0, 127.0, 117.2, 124.7, 123.7, 121.3, 111.0, 100.4, 54. HRMS (ESI+): m/z 292.9791 (292.9784 calculated for $C_{11}H_{11}BrNaO_3$, $[M+Na]^+$)

2-Bromo-3-(dimethoxymethyl)naphthalene

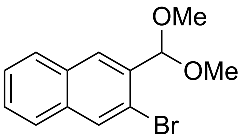

2-Bromo-3-(dimethoxymethyl)naphthalene was synthesized according to the general procedure **A** using 3-bromo-2-naphthaldehyde[6] (122.0 mg, 0.52 mmol), trimethylorthoformate (570 µL, 551 mg, 5.2 mmol), *p*-toluenesulfonic

acid monohydrate (1.0 mg, 5.2 μmol) in MeOH (30 mL) under reflux. The reaction mixture was purified by flash chromatography on silica gel (EtOAc / hexane, gradient from 0 % to 7 % EtOAc) to give the desired product as a colorless oil (136.8 mg, 94 %). ^{1}H-NMR (400 MHz, $CDCl_3$): δ (ppm) 8.10 (s, 2H), 7.89 – 7.83 (m, 1H), 7.78 – 7.71 (m, 1H), 7.54 – 7.48 (m, 2H), 5.71 (s, 1H), 3.43 (s, 6H). ^{13}C-NMR (100 MHz, $CDCl_3$): δ (ppm) 134.4, 133.6, 131.9, 128.6, 127.4, 126.8, 126.7, 131.8, 128.0, 120.2, 102.8, 53.8. HRMS (ESI+): m/z 302.9991 (302.9991 calculated for $C_{13}H_{13}BrNaO_2$, $[M+Na]^+$).

Methyl 3-allyl-4-(dimethoxymethyl)benzoate

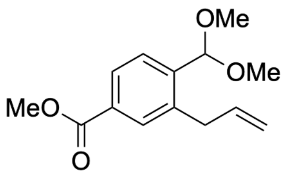

Methyl 3-allyl-4-(dimethoxymethyl)benzoate was prepared according to **general method B** (except deprotection step), by taking methyl 3-bromo-4-(dimethoxymethyl)benzoate (410 mg, 1.42 mmol), $Pd(PPh_3)_4$ (164 mg, 0.14 mmol), cesium fluoride (863 mg, 5.68 mmol) and allylboronic acid pinacol ester (477 mg, 2.84 mmol). Yield 298 mg (84%). ^{1}H-NMR (400 MHz, $CDCl_3$) δ (ppm) 7.90 (dd, 1H, J_1 = 8.0 Hz, J_2 = 1.7 Hz), 7.88 (d, 1H, J = 1.6 Hz), 7.66 (d, 1H, J = 8.1 Hz), 5.98 (ddt, 1H, J_1 = 16.5 Hz, J_2 = 10.1 Hz, J_3 = 6.3 Hz), 5.53 (s, 1H), 5.10 (dq, 1H, J_1 = 10.1 Hz, J_2 = 1.5 Hz), 5.03 (dq, 1H, J_1 = 17.0 Hz, J_2 = 1.7 Hz), 3.91 (s, 3H), 3.54 (br.d, 2H, J = 6.3 Hz), 3.31 (s, 6H); ^{13}C NMR (100 MHz, $CDCl_3$) δ (ppm) 167.0, 140.5, 138.5, 136.6, 131.2, 130.4, 127.3, 127.1, 116.4, 100.9, 53.2 (2C), 52.2, 36.3; HRMS (ESI) m/z 273.1090 (273.1097 calculated for $C_{14}H_{18}NaO_4$, $[M+Na]^+$).

2-Allylbenzofuran-3-carbaldehyde

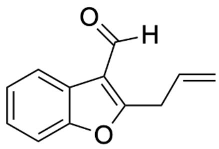

2-Allylbenzofuran-3-carbaldehyde was prepared according to **general method B** (except deprotection step), by taking 2-bromo-3-(dimethoxymethyl)-1-benzofuran (161 mg, 0.59 mmol), $Pd(PPh_3)_4$ (102 mg, 0.09 mmol), cesium fluoride (356 mg, 2.36 mmol) and allylboronic acid pinacol ester (198 mg, 1.18 mmol). For dimethyl acetal deprotection, to a 3-(dimethoxymethyl)-2-(prop-2-en-1-yl)-1-benzofuran (100 mg, 0.43 mmol) solution in CH_3CN (5 mL) Amberlyst 15 (15 mg) was added and stirred for 1 hour at room temperature. Then, resin was filtered out and evaporated to dryness. Product was purified by silica gel column chromatography (eluent Hexane/EtOAc=9:1) to give 37 mg (34%) of 2-(prop-2-en-1-yl)-1-benzofuran-3-carbaldehyde as a yellow oil. Note: deprotection with HCl results in double bond migration, isomers cannot be separated. ^{1}H-NMR (400 MHz, $CDCl_3$) δ (ppm) 10.26 (s, 1H), 8.17 – 8.11 (m, 1H), 7.51 – 7.44 (m, 1H), 7.37 – 7.31 (m, 2H), 6.03 (ddt, 1H, J_1 = 16.5 Hz, J_2 = 10.0 Hz, J_3 = 6.4 Hz), 5.37 – 5.22 (m, 2H), 3.88 (dt, 2H, J_1 = 6.4 Hz, J_2 = 1.4 Hz); ^{13}C-NMR (100 MHz, $CDCl_3$) δ (ppm) 185.0, 167.3, 154.2, 131.7 (2C), 125.5, 124.7, 122.0, 119.0, 117.9, 111.1, 31.8; HRMS (ESI) m/z 187.0757 (187.0754 calculated for $C_{12}H_{11}O_2$, $[M+H]^+$).

3-allyl-2-naphthaldehyde

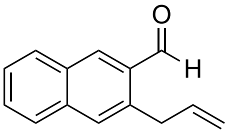

3-allyl-2-naphthaldehyde was prepared according to **general method B,** by taking 2-bromo-3-(dimethoxymethyl)naphthalene (116.3 mg, 0.41 mmol), CsF (251.6 mg, 1.6 mmol), $Pd(PPh_3)_4$ (47.8 mg, 41.4 µmol), and allylboronic acid pinacol ester (156 µL, 139 mg, 0.83 mmol). Yield (54 mg, 67 %). ^{1}H-NMR (400 MHz, $CDCl_3$): δ (ppm) 10.31 (s, 1H), 8.36 (s, 1H), 7.97 (d, 1H, J = 7.8 Hz), 7.84 (d, 1H, J = 8.1 Hz), 7.70 (s, 1H), 7.62 (ddd, 1H, J_1 = 8.2 Hz, J_2 = 6.9 Hz, J_3 = 1.3 Hz), 7.53 (ddd, 1H, J_1 = 8.2 Hz, J_2 = 6.9 Hz, J_3 = 1.2 Hz), 6.13 (ddt, 1H, J_1 = 17.1 Hz, J_2 = 10.2 Hz, J_3 = 6.2 Hz), 5.12 (dq, 1H, J_1 = 10.2 Hz, J_2 = 1.5 Hz), 5.03 (dq, 1H, J_1 = 17.1 Hz, J_2 = 1.7 Hz), 3.97 (d, 2H, J = 6.2 Hz). ^{13}C-NMR (100 MHz, $CDCl_3$): δ (ppm) 192.9, 137.3, 137.1, 136.3, 136.0, 132.6, 131.6, 129.7, 129.4, 127.5, 126.7, 116.5, 31.2. HRMS (ESI) m/z 197.0955 (197.0961 calculated for $C_{14}H_{13}O$, $[M+H]^+$).

2-(3-allylthiophen-2-yl)-1,3-dioxolane

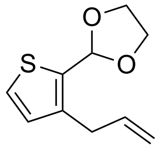

To a flask with 2-(3-bromothiophen-2-yl)-1,3-dioxolane (118.3 mg, 0.50 mmol) in anhydrous Et_2O (5 mL) was dropwise added *n*-BuLi solution (1.57 M, 0.5 mL, 0.76 mmol) at –78 °C and stirred for 1 h. Then allyl bromide solution (87 µL, 0.96 mmol) in Et_2O (5 mL) was added and the reaction mixture was stirred for 3 h at –78 °C. After that, the reaction mixture was warmed to r.t. and quenched with saturated aqueous NH_4Cl solution (10 mL). The aqueous layer was extracted 3 times with Et_2O, combined organic layers were washed once with brine, dried over sodium sulfate, concentrated under reduced pressure, and purified by silica gel column (EtOAc / hexane, 1:8) to give the desired product as a volatile colorless oil (23.1 mg, 23 %). Performing the allylation reaction via general procedure **B** gave the desired product as a mixture of protected and deprotected forms with 75 % yield. ^{1}H-NMR (400 MHz, $CDCl_3$): δ (ppm) 7.23 (d, 1H, J = 5.1 Hz), 6.85 (d, 1H, J = 5.1 Hz), 6.13 (s, 1H), 5.95 (ddt, 1H, J_1 = 17.4 Hz, J_2 = 9.6 Hz, J_3 = 6.3 Hz), 5.10 – 4.01 (m, 2H), 4.21 – 4.09 (m, 2H), 4.07 – 3.95 (m, 2H), 3.44 (dt, 2H, J_1 = 6.3 Hz, J_2 =1.5 Hz). ^{13}C-NMR (100 MHz, $CDCl_3$): δ (ppm) 138.8, 136.5, 135.4, 129.5, 125.2, 116.0, 99.2, 65.4, 32.8.

3-allylthiophene-2-carbaldehyde

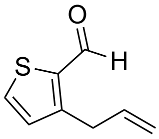

2-(3-Allylthiophen-2-yl)-1,3-dioxolane (39.6 mg, 0.20 mmol), $FeCl_3 \cdot 6H_2O$ (190.9 mg, 0.71 mmol) were mixed in anhydrous CH_2Cl_2 (5 mL) and stirred at r.t. for 40 min. Filtration via short silica gel plug gave desired aldehyde as a light-yellow oil (26.3 mg, 86 %). ^{1}H-NMR (400 MHz, $CDCl_3$): δ (ppm) 10.04 (s, 1H), 7.66 (d, 1H, J = 4.9 Hz), 7.01 (d, 1H, J = 4.9 Hz), 6.04 – 5.92 (m, 1H), 5.15 – 5.05 (m, 2H), 3.73 (dt, 2H, J_1 = 6.4 Hz, J_2 = 1.5 Hz). ^{13}C-NMR (100 MHz, $CDCl_3$): δ (ppm) 182.4, 149.3, 138.0, 135.7, 134.6, 131.1, 117.1, 32.8. GC–MS (EI+): m/z 152.0 (152.029 calculated for C_8H_8OS, $[M]^+$). Data match with previously reported[11].

(3-allyl-4-(dimethoxymethyl)phenyl)methanol

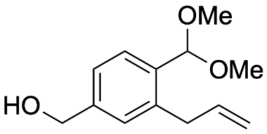

Lithium aluminium hydride (50 mg, 1.32 mmol, 1.5 eq.) was suspended in 10 ml of anhydrous THF, solution was cooled on ice bath, then solution of methyl 3-allyl-4-(dimethoxymethyl)benzoate in THF (5 ml) was added dropwise. Reaction mixture was stirred on ice bath for 30 min, then at room temperature for 2 hr. Excess of $LiAlH_4$ was carefully quenched by water, stirred until solution became white and filtered through short celite pad. The filtrate was washed with brine, dried over sodium sulfate and evaporated to give (3-allyl-4-(dimethoxymethyl)phenyl)methanol 166 mg (85%). Product was used in next step without additional purification. ^{1}H-NMR (300 MHz, $CDCl_3$) δ (ppm) 7.55 (d, 1H, J = 7.8 Hz), 7.22 (d, 1H, J = 9.1 Hz), 7.20 (s, 1H), 5.97 (ddt, 1H, J_1 = 16.7 Hz, J_2 = 10.1 Hz, J_3 = 6.4 Hz), 5.48 (s, 1H), 5.09 – 4.99 (m, 2H), 4.66 (s, 2H), 3.50 (br.d, 2H, J = 6.3 Hz), 3.31 (s, 6H), 1.84 (s, 1H); ^{13}C-NMR (100 MHz, $CDCl_3$) δ (ppm) 141.5, 138.6, 137.3, 135.2, 128.7, 127.4, 124.7, 116.1, 101.6, 65.2, 53.2 (2C), 36.4; HRMS (ESI) m/z 245.1151 (245.1148 calcd for $C_{13}H_{18}NaO_3$, $[M+Na]^+$).

3-allyl-4-(dimethoxymethyl)benzaldehyde

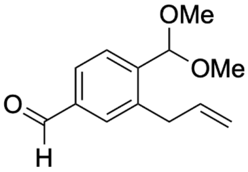

The (3-allyl-4-(dimethoxymethyl)phenyl)methanol (156 mg, 0.7 mmol, 1.0 eq.) was dissolved in 10 ml of CH_2Cl_2 by followed addition of manganese dioxide (1.2 g, 14 mmol, 20 eq.). Reaction progress was monitored by TLC. After s.m. consumption (approximately 1hr), reaction mixture was filtered through celite pad for manganese dioxide removing. The filtrate was evaporated to dryness; the crude product was purified by silica gel column chromatography (eluent Hexane/EtOAc=8:2) to give 147 mg (95%) of 3-allyl-4-(dimethoxymethyl)benzaldehyde. ^{1}H-NMR (400 MHz, $CDCl_3$) δ (ppm) 10.01 (s, 1H), 7.76 – 7.72 (m, 3H), 5.98 (ddt, 1H, J_1 = 16.6 Hz, J_2 = 10.1 Hz, J_3 = 6.4 Hz), 5.54 (s, 1H), 5.13 (dq, 1H, J_1 = 10.1 Hz, J_2 = 1.5 Hz), 5.05 (dq, 1H, J_1 = 17.1 Hz, J_2 = 1.5 Hz), 3.57 (dt, 2H, J_1 = 6.3 Hz, J_2 = 1.3 Hz), 3.33 (s, 6H); ^{13}C-NMR (100 MHz, $CDCl_3$, δ) 192.3, 142.2, 139.3, 136.6, 136.3, 131.2, 127.8, 127.6, 116.8, 100.9, 53.3 (2C), 36.2; HRMS (ESI) m/z 243.0979 (243.0992 calcd for $C_{13}H_{16}NaO_3$, $[M+H]^+$)

(*E*/*Z*)-2-allyl-4-(3,4,5-trimethoxystyryl)benzaldehyde

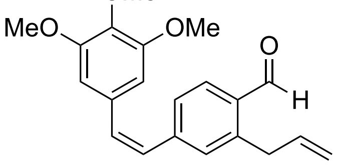

To a round bottom flask, 60% dispersion of sodium hydride (45 mg, 1.14 mmol, 1.7 eq.) was placed, suspended in anhydrous toluene (10 ml) and cooled to 0°C. Then, triphenyl(3,4,5-trimethoxybenzyl)phosphonium bromide[7] (419 mg, 0.8 mmol, 1.2 eq.) was added by one portion and stirred for 30 min at 0°C. After that time solution of 3-allyl-4-(dimethoxymethyl)benzaldehyde (147 mg, 0.67 mmol, 1.0 eq.) in toluene (4 ml) was added dropwise and stirred at 0°C for 20 min, then gradually warmed up to room temperature, stirred for 20 hr. Reaction was quenched by methanol, evaporated to dryness and purified by silica gel column chromatography (hexane/EtOAc = 9:1 – 8:2) to

give 238 mg (92%) of *E/Z*-isomers mixture. Obtained mixture was dissolved in THF (10 ml) and treated with 500 μl of 6M HCl aq. solution for dimethylacetal deprotection. After 1 hr stirring at room temperature, reaction mixture was quenched with saturated aq. $NaHCO_3$, extracted three times with ethyl acetate. The combined organic extracts were washed with brine, dried over sodium sulfate, filtered and the filtrate was evaporated. Obtained *E/Z*-isomers benzaldehyde form were separated by silica gel column chromatography (hexane/EtOAc = 8:2), *Z*-isomer 110 mg (48% over two steps), *E*-isomer 102 mg (42% over two steps). *E*-isomer: ^{1}H-NMR (400 MHz, $CDCl_3$) δ (ppm) 10.21 (s, 1H), 7.84 (d, 1H, *J* = 8.0 Hz), 7.53 (dd, 1H, *J* = 8.0 Hz, J_2 = 1.4 Hz), 7.40 (d, *J* = 1.1 Hz, 1H), 7.18 (d, 1H, *J* = 16.2 Hz), 7.02 (d, 1H, *J* = 16.2 Hz), 6.77 (s, 2H), 6.07 (ddt, 1H, J_1 = 16.4 Hz, J_2 = 10.1 Hz, J_3 = 6.2 Hz), 5.13 (dd, 1H, J_1 = 10.4 Hz, J_2 = 1.5 Hz), 5.04 (dq, 1H, J_1 = 17.1 Hz, J_2 = 1.6 Hz), 3.93 (s, 6H), 3.89 (s, 3H), 3.84 (d, 2H, *J* = 6.2 Hz); ^{13}C-NMR (100 MHz, $CDCl_3$) δ (ppm) 191.7, 153.6(2C), 142.8, 142.8, 138.8, 137.0, 132.9, 132.4, 132.3, 132.1, 129.2, 126.9, 124.7, 116.7, 104.1(2C), 61.1, 56.3(2C), 36.8; *Z*-isomer: ^{1}H-NMR (400 MHz, $CDCl_3$) δ (ppm) 10.18 (s, 1H), 7.73 (d, 1H, *J* = 8.0 Hz), 7.32 (dd, 1H, J_1 = 7.9 Hz, J_2 = 1.4 Hz), 7.21 (s, 1H), 6.66 (d, 1H, *J* = 12.2 Hz), 6.55 (d, 1H, *J* = 12.2 Hz), 6.45 (s, 2H), 5.93 (ddt, 1H, J_1 = 16.5 Hz, J_2 = 10.1 Hz, J_3 = 6.3 Hz), 5.04 (dq, 1H, J_1 = 10.1 Hz, J_2 = 1.4 Hz), 4.93 (dq, 1H, J_1 = 17.2 Hz, J_2 = 1.5 Hz), 3.84 (s, 3H), 3.72 (d, 2H, *J* = 6.3 Hz), 3.66 (s, 6H); ^{13}C-NMR (100 MHz, $CDCl_3$) δ (ppm) 191.8, 153.2(2C), 143.4, 142.3, 137.8, 136.7, 132.8, 132.5, 132.0, 131.8, 131.7, 128.7, 127.6, 116.6, 106.2(2C), 61.0, 56.0(2C), 36.6; HRMS (ESI) m/z 339.1594 (339.1591 calcd for $C_{21}H_{23}O_4$, $[M+H]^+$)

Methyl 1-hydroxy-4-((tetrahydro-2H-pyran-2-yl)oxy)-2-naphthoate

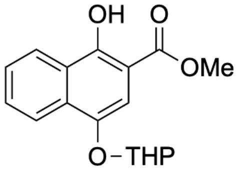

To a suspension of methyl 1,4-dihydroxy-2-naphthoate[8] (375 mg, 1.72 mmol, 1.0 eq.) and dihydropyran (250 μL, 2.75 mmol, 1.6 eq.) in CH_2Cl_2 (15 mL) was added pyridinium p-toluenesulfonate (43 mg, 0.17 mmol, 0.1 eq.). The reaction mixture was stirred at room temperature for 6 h. To workup, the reaction was quenched by the addition of sat. aq. $NaHCO_3$. The product was then extracted with CH_2Cl_2, dried over Na_2SO_4, and concentrated in vacuo. The crude product was purified by silica gel column chromatography (Hexane/EtOAc = 8/2) to give methyl 1-hydroxy-4-((tetrahydro-2H-pyran-2-yl)oxy)-2-naphthoate as a white solid (370 mg, 71%). ^{1}H-NMR (400 MHz, $CDCl_3$) δ (ppm) 11.68 (s, 1H), 8.39 (d, 1H, *J* = 7.9 Hz), 8.22 (d, 1H, *J* = 8.1 Hz), 7.63 (ddd, 1H, J_1 = 8.3 Hz, J_2 = 6.9 Hz, J_3 = 1.3 Hz), 7.55 (ddd, 1H, J_1 = 8.2 Hz, J_2 = 6.9 Hz, J_3 = 1.2 Hz), 7.39 (s, 1H), 5.57 (t, 1H, *J* = 3.0 Hz), 3.96 (s, 3H), 3.99 – 3.90 (m, 1H), 3.66 (dtd, 1H, J_1 = 5.3 Hz, J_2 = 3.8 Hz, J_3 = 0.9 Hz), 2.23 – 1.93 (m, 3H), 1.86 – 1.60 (m, 3H); ^{13}C-NMR (100 MHz, $CDCl_3$) δ (ppm) 171.5, 156.2, 144.7, 130.6, 129.1, 126.3, 125.6, 124.0, 122.0, 105.8, 104.7, 96.8, 62.0, 52.4, 30.7, 25.5, 18.9; HRMS (ESI) m/z 325.1045 (325.1046 calcd for $C_{17}H_{18}NaO_5$, $[M+H]^+$)

Methyl 1-methoxy-4-((tetrahydro-2H-pyran-2-yl)oxy)-2-naphthoate

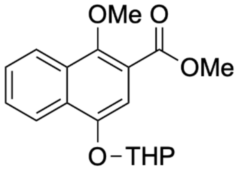

To a solution of methyl 1-hydroxy-4-((tetrahydro-2H-pyran-2-yl)oxy)-2-naphthoate (350 mg, 1.16 mmol, 1.0 eq.) in anhydrous acetone (20 mL) was added K_2CO_3 (401mg, 2.9 mmol, 2.5 eq.). The obtained solution was stirred for 15 min at room temperature. After methyl iodide (145 μL, 2.32 mmol, 2.0 eq.) was added dropwise. The reaction

mixture was refluxed for 15 hours, then cooled down to room temperature and diluted with EtOAc (20 mL), washed with water, brine, dried over Na_2SO_4 and concentrated in vacuo. The crude product was purified by silica gel column chromatography (Hexane/EtOAc = 8/2) to give methyl 1-methoxy-4-((tetrahydro-2H-pyran-2-yl)oxy)-2-naphthoate as a colorless oil (320 mg, 87%). ^{1}H-NMR (400 MHz, $CDCl_3$) δ (ppm) 8.31 – 8.26 (m, 1H), 8.24 – 8.20 (m, 1H), 7.62 – 7.54 (m, 2H), 7.48 (s, 1H), 5.67 (t, 1H, *J* = 3.0 Hz), 4.01 (s, 3H), 3.96 (s, 3H), 3.96 – 3.88 (m, 1H), 3.69 – 3.62 (m, 1H), 2.20 – 2.08 (m, 1H), 2.07 – 1.94 (m, 2H), 1.81 – 1.69 (m, 2H), 1.67 – 1.59 (m, 1H); ^{13}C-NMR (100 MHz, $CDCl_3$) δ (ppm) 166.7, 152.6, 148.5, 129.4, 129.4, 127.9, 127.0, 123.7, 122.4, 119.2, 108.2, 96.6, 63.5, 62.2, 52.4, 30.6, 25.4, 18.9; HRMS (ESI) m/z 317.1386 (317.1384 calcd for $C_{18}H_{21}O_5$, $[M+H]^+$).

Methyl 4-hydroxy-1-methoxy-2-naphthoate

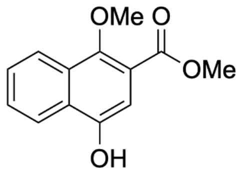

To a solution of methyl 1-methoxy-4-((tetrahydro-2H-pyran-2-yl)oxy)-2-naphthoate (279 mg, 0.88 mmol, 1.0 equiv) in a mixture of MeOH–THF (20 mL, 1:1, v/v) was added p-toluenesulfonic acid monohydrate (17 mg, 88 μmol, 10 mol%) and then the solution was stirred at room temperature for 2 h. To workup, the mixture was quenched with sat. aq. NH_4Cl and extracted with EtOAc. The combined organic layers were washed with water and brine, dried over Na_2SO_4, filtered, and concentrated in vacuo. The crude product was then purified by silica gel column chromatography (hexane/EtOAc = 7:3) to give compound methyl 4-hydroxy-1-methoxy-2-naphthoate as a yellowish solid (173 mg, 85 %). Characterization matched a previous report of this known compound ^{1}H-NMR (400 MHz, $CDCl_3$) δ (ppm) 8.26 – 8.19 (m, 2H), 7.63 – 7.56 (m, 2H), 7.34 (s, 1H), 6.31 (s, 1H), 4.00 (s, 3H), 3.98 (s, 3H); ^{13}C-NMR (100 MHz, $CDCl_3$, δ) 167.3, 152.2, 148.0, 129.6, 128.1, 127.9, 127.2, 123.8, 122.5, 118.7, 108.3, 63.6, 52.7; HRMS (ESI) m/z 233.0806 (233.0808 calcd for $C_{13}H_{13}O_4$, $[M+H]^+$).

Methyl 4-(allyloxy)-1-methoxy-2-naphthoate

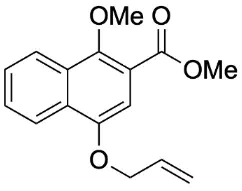

To a solution of 4-hydroxy-1-methoxy-2-naphthoate (173 mg, 0.75 mmol, 1.0 eq.) in anhydrous DMF (10 mL) was added K_2CO_3 (257 mg, 1.86 mmol, 2.5 eq.). The obtained solution was stirred for 15 min at room temperature. After allyl bromide (130 μL, 1.5 mmol, 2.0 eq.) was added dropwise. The reaction mixture was heated up to 85°C and stirred for 15 hours, then cooled down to room temperature and diluted with EtOAc (20 mL), washed with water, brine, dried over Na_2SO_4 and concentrated in vacuo. The crude product was purified by silica gel column chromatography (Hexane/EtOAc = 8/2) to give methyl 4-(allyloxy)-1-methoxy-2-naphthoate as a yellowish oil (175 mg, 86%). ^{1}H-NMR (400 MHz, $CDCl_3$) δ (ppm) 8.33 – 8.27 (m, 1H), 8.25 – 8.19 (m, 1H), 7.62 – 7.53 (m, 2H), 7.17 (s, 1H), 6.17 (ddt, 1H, J_1 = 17.2 Hz, J_2 = 10.5 Hz, J_3 = 5.2 Hz), 5.53 (dq, 1H, J_1 = 17.2 Hz, J_2 = 1.5 Hz), 5.35 (dq, 1H, J_1 = 10.5 Hz, J_2 = 1.3 Hz), 4.73 (dt, 2H, J_1 = 5.0 Hz, J_2 = 1.4 Hz), 4.00 (s, 3H), 3.98 (s, 3H); ^{13}C-NMR (100 MHz, $CDCl_3$) δ (ppm) 166.9, 152.3, 150.4, 133.2, 129.4, 129.1, 127.9, 127.2, 123.6, 122.5, 118.7, 117.8, 105.0, 69.4, 63.5, 52.4; LRMS (ESI) m/z 273.11 (273.11 calcd for $C_{16}H_{17}O_4$, $[M+H]^+$).

Methyl 3-allyl-4-hydroxy-1-methoxy-2-naphthoate

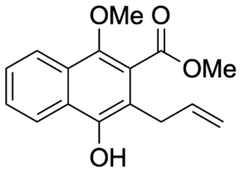

Methyl 4-(allyloxy)-1-methoxy-2-naphthoate (175 mg, 0.64 mmol) with magnetic stirrer bar was placed into 5 mL flask, filled with nitrogen and heated up to 190ºC for 20 min (compound color changed to dark brown). Next, flask was cooled down to room temperature and purified by silica gel column chromatography (hexane/EtOAc = 7/3) to give methyl 3-allyl-4-hydroxy-1-methoxy-2-naphthoate as a yellowish solid (153 mg, 88 %). ^{1}H-NMR (400 MHz, $CDCl_3$) δ (ppm) 8.22 – 8.14 (m, 1H), 8.09 – 8.03 (m, 1H), 7.57 – 7.49 (m, 2H), 6.03 (ddt, 1H, J_1 = 16.2 Hz, J_2 = 10.0 Hz, J_3 = 6.2 Hz), 5.52 (s, 1H), 5.26 (dq, 1H, J_1 = 17.2 Hz, J_2 = 1.7 Hz), 5.23 (dq, 1H, J_1 = 9.8 Hz, J_2 = 1.4 Hz), 3.97 (s, 3H), 3.97 (s, 3H), 3.48 (dt, 2H, J_1 = 6.2 Hz, J_2 = 1.6 Hz); ^{13}C-NMR (100 MHz, $CDCl_3$) δ (ppm) 168.6, 147.5, 146.8, 135.6, 127.4, 126.9, 126.7, 126.6, 125.1, 122.6, 122.2, 117.3, 114.8, 63.7, 52.6, 33.0; HRMS (ESI) m/z 295.0944 (295.0941 calcd for $C_{16}H_{16}NaO_4$, $[M+Na]^+$).

Methyl 3-allyl-1,4-dimethoxy-2-naphthoate

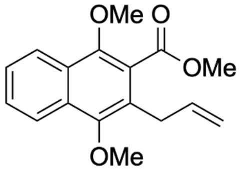

To a solution of 3-allyl-4-hydroxy-1-methoxy-2-naphthoate (153 mg, 0.56 mmol, 1.0 eq.) in anhydrous DMF (10 mL) was added K_2CO_3 (193 mg, 1.4 mmol, 2.5 eq.). The obtained solution was stirred for 15 min at room temperature. After methyl iodide (70 µL, 1.12 mmol, 2.0 eq.) was added dropwise. The reaction mixture was heated up to 70ºC and stirred for 15 hours, then cooled down to room temperature and diluted with EtOAc (20 mL), washed with water, brine, dried over Na_2SO_4 and concentrated in vacuo. The crude product was purified by silica gel column chromatography (Hexane/EtOAc = 8/2) to give methyl 3-allyl-1,4-dimethoxy-2-naphthoate as a colorless oil (151 mg, 94%). ^{1}H-NMR (400 MHz, $CDCl_3$) δ (ppm) 8.10 (dd, 1H, J_1 = 5.1 Hz, J_2 = 1.5 Hz), 8.08 (dd, 1H, J_1 = 5.1 Hz, J_2 = 1.6 Hz), 7.59 – 7.49 (m, 2H), 5.95 (ddt, 1H, J_1 = 17.6 Hz, J_2 = 9.5 Hz, J_3 = 6.2 Hz), 5.08 – 5.02 (m, 2H), 3.98 (s, 3H), 3.93 (s, 3H), 3.90 (s, 3H), 3.63 (dt, 2H, J_1 = 6.2 Hz, J_2 = 1.6 Hz); ^{13}C-NMR (100 MHz, $CDCl_3$) δ (ppm) 168.3, 150.5, 150.4, 136.5, 129.6, 127.7, 127.5, 126.4, 125.6, 125.3, 123.1, 122.7, 116.0, 63.7, 62.6, 52.4, 31.6; HRMS (ESI) m/z 287.1276 (287.1278 calcd for $C_{17}H_{19}O_4$, $[M+H]^+$)

(3-allyl-1,4-dimethoxynaphthalen-2-yl)methanol

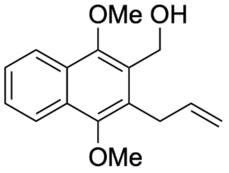

$LiAlH_4$ (50 mg, 1.32 mmol, 1.5 eq.) was suspended in 10 ml of anhydrous Et_2O, solution was cooled on ice bath, then solution of methyl 3-allyl-1,4-dimethoxy-2-naphthoate (100 mg, 0.35 mmol, 1.0 eq.) in Et_2O (5 ml) added dropwise. Reaction mixture was stirred on ice bath for 30 min, then at room temperature for 2 hr. Excess of $LiAlH_4$ was carefully quenched by water, stirred until solution became white and filtered through short celite pad. The filtrate was washed with brine, dried over sodium sulfate and evaporated to give (3-allyl-1,4-dimethoxynaphthalen-2-yl)methanol 73 mg (81%). Product was used in next step without additional purification. ^{1}H-NMR (400 MHz,

$CDCl_3$) δ (ppm) 8.11 – 8.04 (m, 2H), 7.56 – 7.46 (m, 2H), 6.23 – 6.07 (m, 1H), 5.08 (dq, 1H, J_1 = 10.2 Hz, J_2 = 1.7 Hz), 4.93 (dq, 1H, J_1 = 17.1 Hz, J_2 = 1.8 Hz), 4.87 (d, 2H, J = 5.9 Hz), 3.99 (s, 3H), 3.90 (s, 3H), 3.76 (dt, 2H, J_1 = 5.5 Hz, J_2 = 1.8 Hz), 2.25 (t, 1H, J = 6.0 Hz); ^{13}C-NMR (100 MHz, $CDCl_3$) δ (ppm) 151.8, 150.8, 138.4, 129.4, 128.9, 128.0, 127.8, 126.6, 126.0, 122.8, 122.7, 115.6, 63.4, 62.5, 57.7, 30.6; HRMS (ESI) m/z 281.1150 (281.1148 calcd for $C_{16}H_{18}NaO_3$, $[M+Na]^+$).

3-allyl-1,4-dimethoxy-2-naphthaldehyde

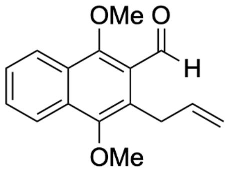

The (3-allyl-1,4-dimethoxynaphthalen-2-yl)methanol (73 mg, 0.28 mmol, 1.0 eq.) was dissolved in 10 ml of CH_2Cl_2 by followed addition of manganese dioxide (487 mg, 5.6 mmol, 20 eq.). Reaction progress was monitored by TLC. After s.m. consumption (approximately 1hr), reaction mixture was filtered through celite pad for manganese dioxide removing. The filtrate was evaporated to dryness; the crude product was purified by silica gel column chromatography (eluent Hexane/EtOAc=8:2) to give 65 mg (91%) of 3-allyl-1,4-dimethoxy-2-naphthaldehyde. ^{1}H-NMR (400 MHz, $CDCl_3$) δ (ppm) 10.67 (s, 1H), 8.21 (d, 1H, J = 8.5 Hz), 8.10 (d, 1H, J = 8.3 Hz), 7.66 (ddd, 1H, J_1 = 8.1 Hz, J_2 = 7.0 Hz, J_3 = 1.2 Hz), 7.56 (ddd, 1H, J_1 = 8.0 Hz, J_2 = 6.9 Hz, J_3 = 1.0 Hz), 6.08 (ddt, 1H, J_1 = 15.9 Hz, J_2 = 10.4 Hz, J_3 = 5.7 Hz), 4.99 (dq, 1H, J_1 = 10.3 Hz, J_2 = 1.7 Hz), 4.93 (dq, 1H, J_1 = 17.2 Hz, J_2 = 1.7 Hz), 4.06 (s, 3H), 3.98 (dt, 2H, J_1 = 5.8 Hz, J_2 = 1.6 Hz), 3.90 (s, 3H); ^{13}C-NMR (100 MHz, $CDCl_3$) δ (ppm) 191.9, 160.3, 151.0, 137.8, 132.1, 129.5, 127.8, 127.8, 126.7, 124.5, 123.4, 123.0, 115.1, 65.5, 62.6, 30.0; HRMS (ESI) m/z 257.1172 (257.1172 calcd for $C_{16}H_{17}O_3$, $[M+H]^+$).

(*Z*)-1-(2-allyl-4-(3,4,5-trimethoxystyryl)phenyl)prop-2-en-1-ol (20)

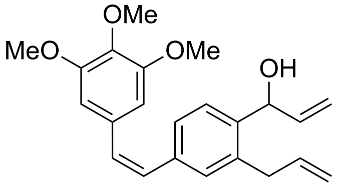

Compound **2** was prepared according to **general method C**, by taking (*Z*)-2-allyl-4-(3,4,5-trimethoxystyryl)benzaldehyde (61 mg, 0.18 mmol) and vinyl magnesium bromide 1M THF solution (220 µL). Yield 48 mg (74%) as a yellowish oil. ^{1}H-NMR (400 MHz, $CDCl_3$) δ (ppm) 7.35 (d, 1H, J = 8.0 Hz), 7.18 (dd, 1H, J_1 = 8.0 Hz, J_2 = 1.7 Hz), 7.09 (d, 1H, J = 1.6 Hz), 6.55 (d, 1H, J = 12.2 Hz), 6.50 (d, 1H, J = 12.2 Hz), 6.45 (s, 2H), 6.00 (ddd, 1H, J_1 = 17.1 Hz, J_2 = 10.4 Hz, J_3 = 5.4 Hz), 5.88 (ddt, 1H, J_1 = 16.6 Hz, J_2 = 10.2 Hz, J_3 = 6.3 Hz), 5.41 (br. s, 1H), 5.29 (dt, J_1 = 17.1 Hz, J_2 = 1.4 Hz), 5.18 (dt, 1H, J_1 = 10.2 Hz, J_2 = 1.1 Hz), 5.01 (dq, 1H, J_1 = 10.2 Hz, J_2 = 1.4 Hz), 4.93 (dq, 1H, J = 17.0 Hz, J_2 = 1.6 Hz), 3.83 (s, 3H), 3.65 (s, 6H), 3.38 (dd, 2H, J_1 = 6.2 Hz, J_2 = 1.4 Hz), 1.98 (d, 1H, J = 3.2 Hz); ^{13}C-NMR (100 MHz, $CDCl_3$, δ) 153.1(2C), 140.0, 139.6, 137.4, 137.3, 137.1, 137.1, 132.8, 130.7, 130.5, 129.8, 127.6, 126.7, 116.2, 115.2, 106.2, 71.3, 61.0, 55.9 (2C), 36.7; HRMS (ESI) m/z 367.1902 (367.1904 calcd for $C_{23}H_{27}O_4$, $[M+H]^+$).

1-(3-allyl-1,4-dimethoxynaphthalen-2-yl)prop-2-en-1-ol (4)

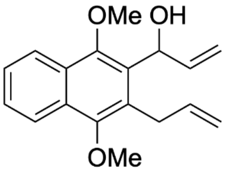

Compound **4** was prepared according to **general method C**, by taking 3-allyl-1,4-dimethoxy-2-naphthaldehyde (65 mg, 0.25 mmol) and vinyl magnesium bromide 1M THF solution (375 µL). Yield 57 mg (80%) as a colorless oil. ^{1}H-NMR (400 MHz, $CDCl_3$) δ (ppm) 8.10 – 8.06 (m, 1H), 8.05 – 7.99 (m, 1H), 7.56 – 7.46 (m, 2H), 6.28 (ddd, 1H, J_1 = 17.3 Hz, J_2 = 10.5 Hz, J_3 = 3.8 Hz), 6.09 (ddt, 1H, J_1 = 17.2 Hz, J_2 = 10.5 Hz, J_3 = 5.4 Hz), 5.60 (ddt, 1H, J_1 = 9.3 Hz, J_2 = 4.0 Hz, J_3 = 2.1 Hz), 5.29 (dt, 1H, J_1 = 17.3 Hz, J_2 = 1.8 Hz), 5.22 (dt, 1H, J_1 = 10.5 Hz, J_2 = 1.8 Hz), 5.11 (dq, 1H, J_1 = 10.1 Hz, J_2 = 1.6 Hz), 4.97 (dq, 1H, J_1 = 17.2 Hz, J_2 = 1.8 Hz), 4.16 (d, 1H, J = 9.4 Hz), 3.96 (s, 3H), 3.90 (s, 3H), 3.8 (ddt, 1H, J_1 = 16.5 Hz, J_2 = 5.4 Hz, J_3 = 1.8 Hz), 3.56 (ddt, 1H, J_1 = 16.5 Hz, J_2 = 5.2 Hz, J_3 = 1.9 Hz); ^{13}C-NMR (100 MHz, $CDCl_3$) δ (ppm) 151.1, 150.9, 141.3, 137.3, 131.3, 128.6, 127.9, 126.8, 126.4, 126.0, 122.8, 122.4, 116.1, 114.0, 71.1, 63.3, 62.6, 30.6; HRMS (ESI) m/z 307.1306 (307.1305 calcd for $C_{18}H_{20}NaO_3$, $[M+Na]^+$)

1-(3-allylnaphthalen-2-yl)prop-2-en-1-ol (5)

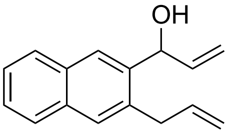

Compound **5** was prepared according to **general method C**, by taking the solution of 3-allyl-2-naphthaldehyde (30.2 mg, 0.15 mmol) in THF (10 mL), vinylmagnesium bromide solution in THF (1 M, 0.23 mL, 0.23 mmol). Yield 30.9 mg (89%) as colorless oil. ^{1}H NMR (400 MHz, $CDCl_3$): δ (ppm) 7.94 (s, 1H), 7.86 – 7.74 (m, 2H), 7.65 (s, 1H), 7.49 – 7.40 (m, 2H), 6.22 – 6.05 (m, 2H), 5.61 – 5.55 (m, 1H), 5.40 (dt, 1H, J_1 = 17.1 Hz, J_2 = 1.4 Hz), 5.29 (dt, 1H, J_1 = 10.4 Hz, J_2 = 1.5 Hz), 5.16 (dq, 1H, J_1 = 10.3 Hz, J_2 = 1.6 Hz), 5.06 (dq, 1H, J_1 = 17.3 Hz, J_2 = 1.8 Hz), 3.65 (d, 2H, J = 5.9 Hz), 2.06 (d, 1H, J = 4.1 Hz). ^{13}C{^{1}H} NMR (100 MHz, $CDCl_3$): δ (ppm) 139.9, 139.2, 137.5, 135.5, 133.2, 132.4, 128.6, 127.9, 127.2, 126.3, 126.0, 125.7, 116.5, 115.6, 71.7, 36.9. HRMS (ESI) m/z 247.1087 (247.1093 calcd for $C_{16}H_{16}NaO$, $[M+Na]^+$).

1-(2-allyl-1-tosyl-1H-indol-3-yl)prop-2-en-1-ol (6)

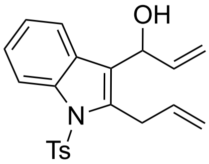

Compound **6** was synthesized according to the **general method C** using 2-allyl-1-tosyl-1*H*-indole-3-carbaldehyde8 (496 mg, 1.5 mmol), THF (10 mL), vinylmagnesium bromide solution (1 M, 5 mL, 5 mmol) at –20°C for 3 h. Yield 442 mg (82%). ^{1}H-NMR (400 MHz, $CDCl_3$): δ (ppm) 8.18 (d, 1H, J = 8.7 Hz), 7.71 (d, 1H, J = 7.8 Hz), 7.67 – 7.62 (m, 2H), 7.31 – 7.25 (m, 1H), 7.24 – 7.20 (m, 1H), 7.20 – 7.15 (m, 2H), 6.13 (ddd, 1H, J_1 = 16.9 Hz, J_2 = 10.4 Hz, J_3 = 5.0 Hz), 6.01 (ddt, 1H, J_1 = 16.8 Hz, J_2 = 10.1 Hz, J_3 = 5.9 Hz), 5.48 (m, 1H), 5.33 (dt, 1H, J_1 = 16.9 Hz, J_2 = 1.4 Hz), 5.18 (dt, 1H, J_1 = 10.4 Hz, J_2 = 1.4 Hz), 5.05 (dq, 1H, J_1 = 10.2 Hz, J_2 = 1.2 Hz), 5.00 (dq, 1H, J_1 = 16.8 Hz, J_2 = 1.3 Hz), 3.95 (ddt, 1H, J_1 = 16.4 Hz, J_2 = 5.7 Hz, J_3 = 1.6 Hz), 3.84 (ddt, 1H, J_1 = 16.8 Hz, J_2 = 5.9 Hz, J_1 = 1.7 Hz), 2.34 (s, 3H), 1.89 (d, 1H, J = 3.2 Hz). ^{13}C-NMR (100 MHz, $CDCl_3$): δ (ppm) 145.0, 138.4, 136.9, 136.2, 135.5,

135.4, 129.9, 128.2, 126.6, 124.5, 123.5, 121.8, 120.6, 116.5, 115.5, 115.2, 68.6, 30.2, 21.7. HRMS (ESI+): m/z 390.1135 (390.1134 calculated for $C_{21}H_{21}NNaO_3S$, [M + Na]$^+$).

1-(2-allylbenzofuran-3-yl)prop-2-en-1-ol (7)

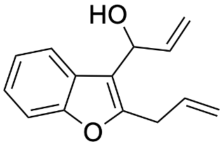

Compound **7** was prepared according to **general method C**, by taking 2-allylbenzofuran-3-carbaldehyde (37 mg, 0.18 mmol) and vinyl magnesium bromide 1M THF solution (300 μL). Yield 27 mg (63%) as a yellowish oil. ^{1}H-NMR (400 MHz, $CDCl_3$) δ (ppm) 7.67 – 7 62 (m, 1H), 7.41 (d, 1H, J = 7.6 Hz), 7.23 (td, 1H, J_1 = 7.7 Hz, J_2 = 1.8 Hz), 7.18 (td, 1H, J_1 = 7.4 Hz, J_2 = 1.2 Hz), 6.19 (ddd, 1H, J_1 = 17.1 Hz, J_2 = 10.2 Hz, J_3 = 5.3 Hz), 5.97 (ddt, 1H, J_1 = 16.5 Hz, J_2 = 10.2 Hz, J_3 = 6.3 Hz), 5.46 (ddd, 1H, J_1 = 5.2 Hz, J_2 = 3.3 Hz, J_3 = 1.6 Hz), 5.41 (dt, 1H, J_1 = 17.1 Hz, J_2 = 1.4 Hz), 5.23 (dt, 1H, J_1 = 10.4 Hz, J_2 = 1.4 Hz), 5.19 – 5.11 (m, 2H), 3.59 (d, 2H, J = 6.3 Hz), 2.00 (d, 1H, J = 3.4 Hz); ^{13}C-NMR (100 MHz, $CDCl_3$, δ) 154.4, 152.9, 138.7, 133.6, 127.3, 123.9, 122.6, 120.5, 117.3, 116.2, 115.4, 111.1, 68.1, 31.5; LRMS (ESI) m/z 215.11 (215.11 calcd for $C_{14}H_{15}O_2$, [M+H]$^+$)

1-(3-allylthiophen-2-yl)prop-2-en-1-ol (8)

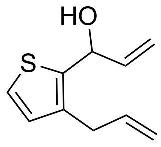

Compound **8** was synthesized according to the **general method C** using the solution of 3-allylthiophene-2-carbaldehyde (32.0 mg, 0.21 mmol) in THF (5 mL), vinylmagnesium bromide solution 1 M in THF (420 μL, 0.42 mmol) at 0°C, reaction time 3 h. Yield 28.0 mg (74 %) as light-yellow oil. ^{1}H-NMR (400 MHz, $CDCl_3$): δ (ppm) 7.20 (d, 1H, J = 5.0 Hz,), 6.85 (d, 1H, J = 5.0 Hz,), 6.10 (ddd, 1H, J_1 = 17.1 Hz, J_2 = 10.4 Hz, J_3 = 5.6 Hz), 5.95 (ddt, 1H, J_1 = 16.7 Hz, J_2 = 10.3 Hz, J_3 = 6.4 Hz), 5.53–5.48 (m, 1H), 5.40 (dt, 1H, J_1 = 17.1 Hz, J_2 = 1.4 Hz), 5.23 (dt, 1H, J_1 = 10.4 Hz, J_2 = 1.3 Hz), 5.08 – 5.00 (m, 2H), 3.40 (dt, 2H, J_1 = 6.4 Hz, J_2 = 1.5 Hz), 2.06 (d, 1H, J = 3.8 Hz). ^{13}C-NMR (100 MHz, $CDCl_3$): δ (ppm) 140.3, 139.2, 136.9, 136.5, 129.6, 124.2, 115.9, 115.6, 69.1, 32.8. GC–MS (EI+): m/z 180.1 (180.0603 calculated for $C_{10}H_{12}OS$, [M]$^+$).

1-allyl-2-(1-(benzyloxy)allyl)benzene (10)

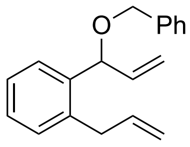

To a solution of sodium hydride (22 mg, 0.54 mmol of 60% dispersion in mineral oil) in THF (4 ml) was added 2 ml THF solution of compound **2** (53 mg, 0.3 mmol). The reaction mixture was stirred at room temperature for 30 min, then cooled on ice bath and benzyl bromide (72 μL, 0.6 mmol) was added. The reaction mixture was stirred at room temperature overnight, quenched with H_2O, then extracted with EtOAc. The organic layer was washed with H_2O and brine, dried over $MgSO_4$ and concentrated in vacuo. The residue was purified by column chromatography (Hexane/EtOAc = 10:1) to afford compound **10** as a colorless oil. Yield 58 mg (72%). ^{1}H-NMR (400 MHz, $CDCl_3$) δ (ppm) 7.51 (dd, 1H, J_1 = 7.2 Hz, J_2 = 1.9 Hz), 7.37 – 7.29 (m, 4H), 7.79 – 7.22 (m, 3H), 7.17 (dd, 1H, J_1 = 7.2 Hz, J_2 = 1.8 Hz), 6.04 – 5.95 (m, 2H), 5.26 – 5.18(m, 2H), 5.09 (d, 1H, J = 6.2 Hz), 5.02 (dq, 1H, J_1 = 10.0 Hz, J_2 = 1.5 Hz), 4.94 (dq, 1H, J_1 = 17.1 Hz, J_2 = 1.7 Hz), 4.47 (m, 2H), 3.37 (m, 2H); ^{13}C-NMR (100 MHz, $CDCl_3$) δ (ppm) 138.8,

138.6, 138.5, 137.7, 137.2, 130.0, 128.5 (2C), 127.9 (3C), 127.6, 127.4, 126.9, 116.5, 116.1, 78.4, 70.3, 36.8; LRMS (ESI) m/z 287.14 (287.14 calcd for $C_{19}H_{20}NaO$, $[M+Na]^+$)

1-allyl-2-(1-phenoxyprop-2-yn-1-yl)benzene

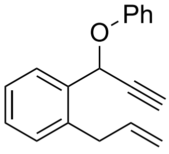

In round bottom flask, 1-(2-allylphenyl)-3-(trimethylsilyl)prop-2-yn-1-ol[10] (116 mg, 0.47 mmol), PPh_3 (185 mg, 0.71 mmol) and phenol (45 mg, 0.47 mmol) were mixed and dissolved in 5 mL of dry toluene. The solution was cooled on ice bath and then 3 mL toluene solution of DIAD (142 mg, 0.71 mmol) was added very slowly (over 30 min). The solution was warmed to room temperature and stirred for 2 hours. Then the mixture was concentrated in vacuo, crude product was purified using column chromatography (eluent Hexane/EtOAc = 10:1). Isolated product was dissolve in MeOH, treated with K_2CO_3 and stirred for 2 hours for TMS deprotection. Then, reaction mixture was quenched with water, MeOH was evaporated, residue extracted with EtOAc. Yield 71 mg (61%). ^{1}H-NMR (400 MHz, $CDCl_3$) δ (ppm) 7.80 (dd, 1H, J_1 = 7.2 Hz, J_2 = 1.8 Hz), 7.35 – 7.27 (m, 4H), 7.25 – 7.23 (m, 1H), 7.07 (d, 2H, J = 8.3 Hz), 7.00 (t, 1H, J = 7.3 Hz), 6.01 (d, 1H, J = 2.3 Hz), 6.04 – 5.93 (m, 1H), 5.07 (dq, 1H, J_1 = 10.1 Hz, J_2 = 1.5 Hz), 5.00 (dq, 1H, J_1 = 17.1 Hz, J_2 = 1.6 Hz), 3.51 (d, 2H, J = 6.3 Hz), 2.67 (d, 1H, J = 2.2 Hz); ^{13}C-NMR (100 MHz, $CDCl_3$) δ (ppm) 157.4, 137.9, 136.7, 135.4, 130.4, 129.6 (2C), 129.3, 128.1, 127.0, 121.9, 116.5, 116.0 (2C), 80.9, 76.8, 67.3, 37.0; LRMS (ESI) m/z 271.11 (271.11 calcd for $C_{18}H_{16}NaO$, $[M+Na]^+$).

1-allyl-2-(1-(benzyloxy)allyl)benzene (11)

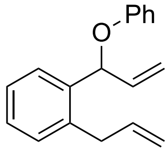

In round bottom flask, 1-allyl-2-(1-phenoxyprop-2-yn-1-yl)benzene (20 mg, 0.08 mmol) and Lindlar catalyst (2 mg) were mixed and suspended in 3 mL of EtOH/Heptene-1 (1:1 mixture). Flask was filled with hydrogen gas (1 atm), reaction mixture was stirred for 2 hours, reaction progress was monitored by TLC (Hexane/Toluene = 7:3). Then, solution was filtered through celite pad and purified by column chromatography (eluent Hexane/Toluene = 7:3) Yield 18 mg (90%). ^{1}H-NMR (400 MHz, $CDCl_3$) δ (ppm) 7.51 – 7.47 (m, 1H), 7.26 – 7.19 (m, 5H), 6.92 – 6.88 (m, 3H), 6.11 (ddd, 1H, J_1 = 17.4 Hz, J_2 = 10.1 Hz, J_3 = 5.5 Hz), 5.98 (ddt, 1H, J_1 = 16.7 Hz, J_2 = 10.1 Hz, J_3 = 6.4 Hz), 5.87 (d, 1H, J = 5.4 Hz), 5.31 – 5.26 (m, 2H), 5.11 (dq, 1H, J_1 = 10.2 Hz, J_2 = 1.5 Hz), 5.05 (dq, 1H, J_1 = 17.1 Hz, J_2 = 1.7 Hz), 3.54 – 3.43 (m, 2H); ^{13}C-NMR (100 MHz, $CDCl_3$) δ (ppm) 158.0, 137.9, 137.2, 137.1, 136.9, 130.2, 129.5 (2C), 128.1, 127.3, 127.0, 121.0, 117.1, 116.5, 116.2 (2C), 77.3, 36.9; LRMS (ESI) m/z 273.13 (273.13 calcd for $C_{18}H_{18}NaO$, $[M+Na]^+$).

1-(2-allylphenyl)allyl benzoate (12)

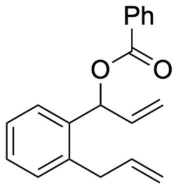

Compound **12** was prepared according to **general method D,** by taking compound **2** (28 mg, 0.16 mmol), benzoic acid (26 mg, 0.21 mmol), EDCI (61 mg, 0.32 mmol), DMAP (4 mg, 32 μmol). Yield 20 mg (45%) as a colorless oil. ^{1}H-NMR (400 MHz, $CDCl_3$) δ (ppm) 8.10 – 8.08 (m, 2H), 7.58 – 7.50 (m, 2H), 7.46 – 7.42 (m, 2H), 7.28 – 7.26 (m, 2H), 7.22 – 7.20 (m, 1H), 6.74 (dt, 1H, J_1 = 5.2 Hz, J_2 = 1.4 Hz), 6.12 (ddd, 1H, J_1 = 17.2 Hz, J_2 = 10.5 Hz, J_3 = 5.3 Hz), 6.01 (ddt, 1H, J_1 = 16.7 Hz, J_2 = 10.2 Hz, J_3 = 6.3 Hz), 5.31 (dt, 1H, J_1 = 17.1 Hz, J_2 = 1.3 Hz), 5.28 (dt, 1H, 1H, J_1 = 10.6 Hz, J_2 = 1.3 Hz), 5.10 – 5.00 (m, 2H), 3.63 (dd, 1H, J_1 = 16.0 Hz, J_2 = 6.5 Hz), 3.54 (ddt, 1H, J_1 = 15.9 Hz, J_2 = 6.0 Hz, J_3 = 1.5 Hz); ^{13}C-NMR (100 MHz, $CDCl_3$) δ (ppm) 165.6, 137.8, 137.1, 137.0, 136.3, 133.2, 130.4, 130.1, 129.9, 128.5, 128.5, 127.8, 126.9, 117.0, 116.4, 73.5, 37.1; LRMS (ESI) m/z 301.12 (301.12 calcd for $C_{19}H_{18}NaO_2$, $[M+Na]^+$).

1-(2-allylphenyl)allyl 3,7-dimethyloct-6-enoate (14)

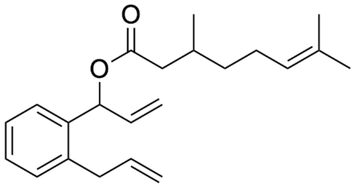

Compound **14** was prepared according to **general method D,** by taking compound **2** (49 mg, 0.28 mmol), citronellic acid (61 mg, 0.36 mmol), EDCI (107 mg, 0.56 mmol), DMAP (6.8 mg, 56 μmol). Yield 75 mg (83%) as a colorless oil. ^{1}H-NMR (400 MHz, $CDCl_3$) δ (ppm) 7.42 – 7.36 (m, 1H), 7.27 – 7.22 (m, 2H), 7.20 – 7.17 (m, 1H), 6.50 (d, 1H, J = 5.4 Hz), 6.04 – 5.92 (m, 2H (overlapped)), 5.21 – 5.17 (m, 2H (overlapped)), 5.11 – 5.05 (m, 2H (overlapped)), 5.01 (ddd, 1H, J_1 = 17.0 Hz, J_2 = 3.5 Hz, J_3 = 1.8 Hz), 3.56 (ddd, 1H, J_1 = 16.0 Hz, J_2 = 6.5 Hz, J_3 = 0.9 Hz), 3.46 (ddt, 1H, J_1 = 16.0 Hz, J_2 = 6.1 Hz, J_3 = 1.5 Hz), 2.36 (ddd, 1H, J_1 = 14.8 Hz, J_2 = 7.1 Hz, J_3 = 6.0 Hz), 2.16 (ddd, 1H, J_1 = 14.8 Hz, J_2 = 8.2 Hz, J_3 = 3.1 Hz), 2.04 – 1.86 (m, 3H), 1.67 (s, 3H), 1.58 (s, 3H), 1.33 – 1.27 (m, 1H), 1.25 – 1.14 (m, 1H), 0.92 (dd, 3H, J_1 = 6.6 Hz, J_2 = 3.8 Hz); ^{13}C-NMR (100 MHz, $CDCl_3$) δ (ppm) 172.3, 137.7, 137.2, 137.1, 136.5, 131.7, 130.0, 128.3, 127.8, 126.8, 124.4, 116.8, 116.3, 72.7, 42.1, 37.0, 36.9, 30.2, 25.8, 25.5, 19.8, 17.8; LRMS (ESI) m/z 349.21 (349.21 calcd for $C_{22}H_{30}NaO_2$, $[M+Na]^+$).

1-(2-allylphenyl)allyl pivalate (15)

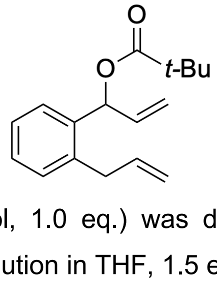

The 2-allylbenzaldehyde (138 mg, 0.95 mmol, 1.0 eq.) was dissolved in THF (5 mL) and cooled to 0 °C. Vinylmagnesium bromide (1430 μL of 1.0 M solution in THF, 1.5 eq.) was then added. This solution was stirred at 0 °C for 1.5 h. Then, pyridine (193 μL, 2.4 mmol, 2.5 eq.) and pivaloyl chloride (209 μL, 1.7 mmol, 1.8 eq.) were added. The reaction mixture was warmed to room temperature and stirred for an additional 2 h. The crude reaction mixture was quenched with aq. NH_4Cl solution. The organic layer was separated and washed with brine, then dried over Na_2SO_4, filtered, and concentrated. The crude reaction mixtures were purified by silica gel column

chromatography (eluent Hexanes:EtOAc = 95:5) to yield 92 mg (37%) of desired compound **15,** as a colorless oil.^{1}H-NMR (400 MHz, $CDCl_3$) δ (ppm) 7.44 – 7.34 (m, 1H), 7.28 – 7.21 (m, 2H), 7.21 – 7.16 (m, 1H), 6.44 (dt, 1H, J_1 = 5.1 Hz, J_2 = 1.5 Hz), 6.07 – 5.91 (m, 2H (overlapped)), 5.25 – 5.16 (m, 2H (overlapped)), 5.08 (ddd, 1H, J_1 = 10.1 Hz, J_2 = 3.1 Hz, J_3 = 1.4 Hz), 5.02 (dq, 1H, J_1 = 17.0 Hz, J_2 = 1.7 Hz), 3.56 (dd, 1H, J_1 = 16.0 Hz, J_2 = 6.6 Hz), 3.48 (ddt, 1H, J_1 = 15.9 Hz, J_2 = 6.0 Hz, J_3 = 1.5 Hz) 1.22 (s, 9H); ^{13}C-NMR (100 MHz, $CDCl_3$) δ (ppm) 177.4, 137.6, 137.4, 137.0, 136.5, 129.9, 128.2, 127.4, 126.8, 116.4, 116.3, 72.5, 39.0, 37.0, 27.3; LRMS (ESI) m/z 281.15 (281.15 calcd for $C_{17}H_{22}NaO_2$, $[M+Na]^+$).

(Z)-1-(2-allyl-4-(3,4,5-trimethoxystyryl)phenyl)allyl 3,7-dimethyloct-6-enoate (16)

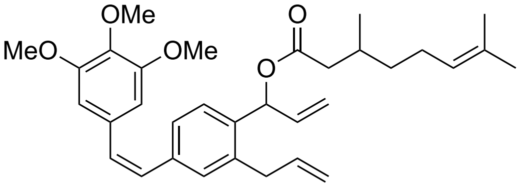

Compound **16** was prepared according to **general method D,** by taking compound **19** (17 mg, 46 μmol), citronellic acid (10 mg, 60 μmol), EDCI (18 mg, 93 μmol), DMAP (1 mg, 9 μmol). Yield 17 mg (71%) as a colorless oil. ^{1}H-NMR (400 MHz, $CDCl_3$) δ (ppm) 7.28 (d, 1H, J = 8.0 Hz), 7.16 (dd, 1H, J_1 = 8.0 Hz, J_2 = 1.5 Hz), 7.11 (d, 1H, J = 1.4 Hz), 6.55 (d, 1H, J = 12.2 Hz), 6.50 (d, 1H, J = 12.2 Hz), 6.44 (s, 2H), 6.42 (d, 1H, J = 1.4 Hz), 5.96 (ddd, 1H, J_1 = 16.8 Hz, J_2 = 10.9 Hz, J_3 = 5.4 Hz), 5.88 (ddt, 1H, J_1 = 16.6 Hz, J_2 = 10.2 Hz, J_3 = 6.3 Hz), 5.20 (d, J = 10.6 Hz), 5.18 (d, 1H, J = 17.1 Hz), 5.02 (dd, 1H, J_1 = 10.1 Hz, J_2 = 1.5 Hz), 4.95 (dq, 1H, J = 17.0 Hz, J_2 = 1.6 Hz), 3.83 (s, 3H), 3.64 (s, 6H), 3.47 (dd, 1H, J_1 = 15.9 Hz, J_2 = 6.6 Hz), 3.37 (dd, 1H, J_1 = 15.6 Hz, J_2 = 6.1 Hz), 2.39 – 2.28 (m, 1H), 2.14 (ddd, 1H, J_1 = 8.2 Hz, J_2 = 6.7 Hz), 2.04 – 1.88 (m, 3H), 1.67 (s, 3H), 1.58 (s, 3H), 1.39 – 1.27 (m, 1H), 1.25 – 1.14 (m, 1H), 0.91 (dd, 3H, J_1 = 6.6 Hz, J_2 = 4.5 Hz); ^{13}C-NMR (100 MHz, $CDCl_3$) δ (ppm) 172.1, 152.9 (2C), 137.6, 137.4, 137.3, 136.6, 136.3, 136.1, 132.5, 131.6, 130.6, 130.4, 129.6, 127.5, 127.3, 124.3, 116.8, 116.3, 106.1, 72.6, 61.0, 55.9 (2C), 42.0, 36.8, 30.1, 30.1, 25.8, 25.5, 19.7, 17.1; HRMS (ESI) m/z 519.3102 (519.3105 calcd for $C_{33}H_{43}O_5$, $[M+H]^+$).

(Z)-1-(2-allyl-4-(3,4,5-trimethoxystyryl)phenyl)allyl pivalate (17)

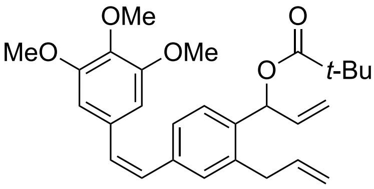

The (*Z*)-2-allyl-4-(3,4,5-trimethoxystyryl)benzaldehyde (40 mg, 0.12 mmol, 1.0 eq.) was dissolved in THF (5 mL) and cooled to 0 °C. Vinylmagnesium bromide (180 μL of 1.0 M solution in THF, 1.5 eq.) was then added. This solution was stirred at 0 °C for 1.5 h. Then, pyridine (24 μL, 0.3 mmol, 2.5 eq.) and pivaloyl chloride (27 μL, 0.22 mmol, 1.8 eq.) were added. The reaction mixture was warmed to room temperature and stirred for an additional 2 h. After that time, reaction mixture was quenched with aq. NH_4Cl solution. The organic layer was separated and washed with brine, then dried over Na_2SO_4, filtered, and concentrated. The crude reaction mixtures were purified by silica gel column chromatography (eluent Hexanes:EtOAc = 8:2 – 7:3) to yield 41 mg (76%) of desired compound **17,** as a colorless oil. ^{1}H-NMR (400 MHz, $CDCl_3$) δ (ppm) 7.27 (d, 1H, J = 8.0 Hz), 7.17 (dd, 1H, J_1 = 8.0 Hz, J_2 = 1.7 Hz), 7.11 (d, 1H, J_1 = 1.5 Hz), 6.56 (d, 1H, J = 12.1 Hz), 6.50 (d, 1H, J = 12.1 Hz), 6.44 (s, 2H), 6.38 (d, 1H, J = 5.2 Hz), 6.00 – 5.82 (m, 2H), 5.24 – 5.15 (m, 2H), 5.03 (dq, 1H, J_1 = 10.2 Hz, J_2 = 1.5 Hz), 4.97 (dq, 1H, J_1 = 17.0 Hz, J_2 = 1.6 Hz), 3.83 (s, 3H), 3.64 (s, 6H), 3.48 (dd, 1H, J_1 = 15.8 Hz, J_1 = 6.7 Hz), 3.40 (dd, 1H, J_1 = 15.9 Hz, J_1

= 6.1 Hz), 1.20 (s, 9H); ^{13}C-NMR (100 MHz, $CDCl_3$) δ (ppm) 177.3, 153.0 (2C), 137.6, 137.4, 137.3, 136.7, 136.4, 136.3, 132.6, 130.5, 130.4, 129.7, 127.3, 127.2, 116.4 (2C), 106.1 (2C), 72.4, 61.0, 55.9 (2C), 38.9, 36.8, 27.2 (3C); HRMS (ESI) m/z 493.1981 (493.1985 calcd for $C_{30}H_{30}NaO_5$, $[M+Na]^+$).

(Z)-1-(2-allyl-4-(3,4,5-trimethoxystyryl)phenyl)allyl benzoate (18)

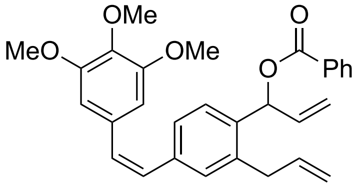

The (*Z*)-2-allyl-4-(3,4,5-trimethoxystyryl)benzaldehyde (50 mg, 0.15 mmol, 1.0 eq.) was dissolved in THF (5 mL) and cooled to 0 °C. Vinylmagnesium bromide (225 µL of 1.0 M solution in THF, 1.5 eq.) was then added. This solution was stirred at 0 °C for 1.5 h. Then, pyridine (30 µL, 0.37 mmol, 2.5 eq.) and benzoyl chloride (31 µL, 0.27 mmol, 1.8 eq.) were added. The reaction mixture was warmed to room temperature and stirred for an additional 2 h. The crude reaction mixture was quenched with aq. NH_4Cl solution. The organic layer was separated and washed with brine, then dried over Na_2SO_4, filtered, and concentrated. The crude reaction mixtures were purified by silica gel column chromatography (eluent Hexanes:EtOAc = 8:2 – 7:3) to yield 52 mg (73%) of desired compound **18**, as a colorless oil. ^{1}H-NMR (400 MHz, $CDCl_3$) δ (ppm) 8.10 – 8.02 (m, 2H), 7.60 – 7.52 (m, 1H), 7.47 – 7.38 (m, 3H), 7.19 (dd, 1H, J_1 = 8.0 Hz, J_2 = 1.7 Hz), 7.13 (d, 1H, J = 1.6 Hz), 6.67 (d, 1H, J = 5.3 Hz), 6.56 (d, 1H, J = 12.1 Hz), 6.50 (d, 1H, J = 12.1 Hz), 6.42 (s, 2H), 6.09 (ddd, 1H, J_1 = 17.2 Hz, J_2 = 10.5 Hz, J_3 = 5.3 Hz), 5.91 (ddt, 1H, J_1 = 16.7 Hz, J_2 = 10.1 Hz, J_3 = 6.4 Hz), 5.30 (dt, 1H, J_1 = 17.2 Hz, J_2 = 1.4 Hz), 5.27 (dt, 1H, J_1 = 10.6 Hz, J_2 = 1.3 Hz), 5.01 (m, 2H), 3.81 (s, 3H), 3.59 (s, 6H), 3.58 (m, 1H), 3.46 (dd, 1H, J_1 = 15.6 Hz, J_2 = 6.1 Hz); ^{13}C-NMR (100 MHz, $CDCl_3$) δ (ppm) 165.5, 152.9 (2C), 137.8, 137.7, 137.3, 136.7, 136.1, 136.1, 133.3, 132.5, 130.7, 130.5, 130.2, 129.7 (2C), 129.6, 128.6 (2C), 127.6, 127.5, 117.0, 116.5, 106.1 (2C), 73.4, 61.0, 55.9 (2C), 37.0; HRMS (ESI) m/z 451.2478 (451.2479 calcd for $C_{28}H_{35}O_5$, $[M+H]^+$).

The non-aromatized intermediates were prepared according to the following procedure: In microtube were mixed 9 μmol of substrate stock solution (450 μL, 20 mM in $CDCl_3$) and 90 μL of Hoveyda-Grubbs II generation stock solution (1 mM in $CDCl_3$). The mixture was stirred for 2 hours at room temperature, next analyzed by ^{1}H NMR, without additional purification.

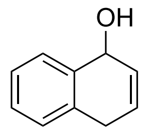	^{1}H-NMR (400 MHz, $CDCl_3$) δ (ppm) 7.62 – 7.56 (m, 1H), 7.32 – 7.23 (m, 2H), 7.18 (d, 1H, J = 7.2 Hz), 6.16 – 6.06 (m, 2H), 5.17 (br. s, 1H), 3.53 – 3.29 (m, 2H), 1.76 (d, 1H, J = 9.2 Hz).
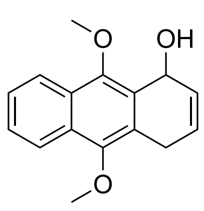	^{1}H-NMR (400 MHz, $CDCl_3$) δ (ppm) 8.12 – 8.03 (m, 2H), 7.57 – 7.44 (m, 2H), 6.25 (dtd, 1H, J_1 = 10.2 Hz, J_2 = 3.5 Hz, J_3 = 1.0 Hz), 6.16 (ddt, 1H, J_1 = 10.1 Hz, J_2 = 4.1 Hz, J_3 = 2.1 Hz), 5.69 – 5.62 (m, 1H), 4.06 (s, 3H), 3.92 (s, 3H), 3.63 (ddd, 1H, J_1 = 23.0 Hz, J_2 = 5.7 Hz, J_3 = 3.1 Hz), 3.54 (dtd, 1H, J_1 = 23.0 Hz, J_2 = 3.5 Hz, J_3 = 1.9 Hz), 3.44 (d, 1H, J = 3.6 Hz).
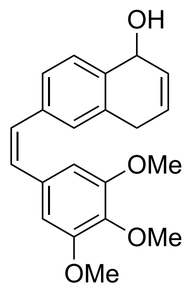	^{1}H-NMR (400 MHz, $CDCl_3$) δ (ppm) 7.46 (d, 1H, J = 8.0 Hz), 7.24 (dd, 1H, J_1 = 8.1 Hz, J_2 = 1.2 Hz), 7.13 (s, 1H), 6.56 (d, 1H, J = 12.3 Hz), 6.50 (d, 1H, J = 12.0 Hz), 6.50 (s, 2H), 6.15 – 6.03 (m, 2H), 5.13 (br.s, 1H), 3.84 (s, 3H), 3.67 (s, 3H), 3.41 – 3.21- (m, 2H), 1.73 (d, 1H, J = 9.0 Hz).
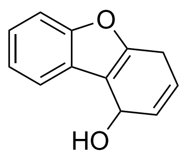	^{1}H-NMR (400 MHz, $CDCl_3$) δ (ppm) 7.74 – 7.68 (m, 1H), 7.49 – 7.43 (m, 1H), 7.32 – 7.23 (m, 2H), 6.17 – 6.11 (m, 1H), 6.06 (dtd, J_1 = 10.1 Hz, J_2 = 3.3 Hz, J_3 = 1.0 Hz), 5.55 – 5.43 (m, 1H), 3.47 (dddd, 1H, J_1 = 22.7 Hz, J_2 = 5.2 Hz, J_3 = 3.3 Hz, J_4 = 2.3 Hz), 3.42 – 3.33 (m, 1H), 1.57 (d, 1H, J = 9.8 Hz).
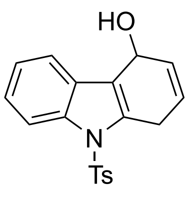	^{1}H-NMR (400 MHz, $CDCl_3$) δ (ppm) 8.17 (d, 1H, J = 8.1 Hz), 7.71 (d, 1H, J = 6.9 Hz), 7.69 – 7.65 (m, 2H), 7.33 – 7.25 (m, 2H), 7.19 (d, 2H, J = 8.1 Hz), 6.07 (br. s, 2H), 5.38 (br. s, 1H), 3.78 – 3.58 (m, 2H), 2.33 (s, 3H), 1.70 (d, 1H, J = 9.6 Hz).
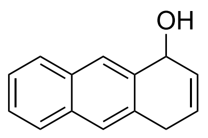	^{1}H-NMR (400 MHz, $CDCl_3$) δ (ppm) 8.1 (s, 1H), 7.87 – 7.83 (m, 1H), 7.80 – 7.75 (m, 1H), 7.68 (s, 1H), 7.48 – 7.39 (m, 2H), 6.23 – 6.15 (m, 2H), 5.38 – 5.31 (m, 1H), 3.70 – 3.50 (m, 2H), 1.92 (d, 1H, J = 8.9 Hz).

2. Supplementary figures (^{1}H and ^{13}C NMR spectra)

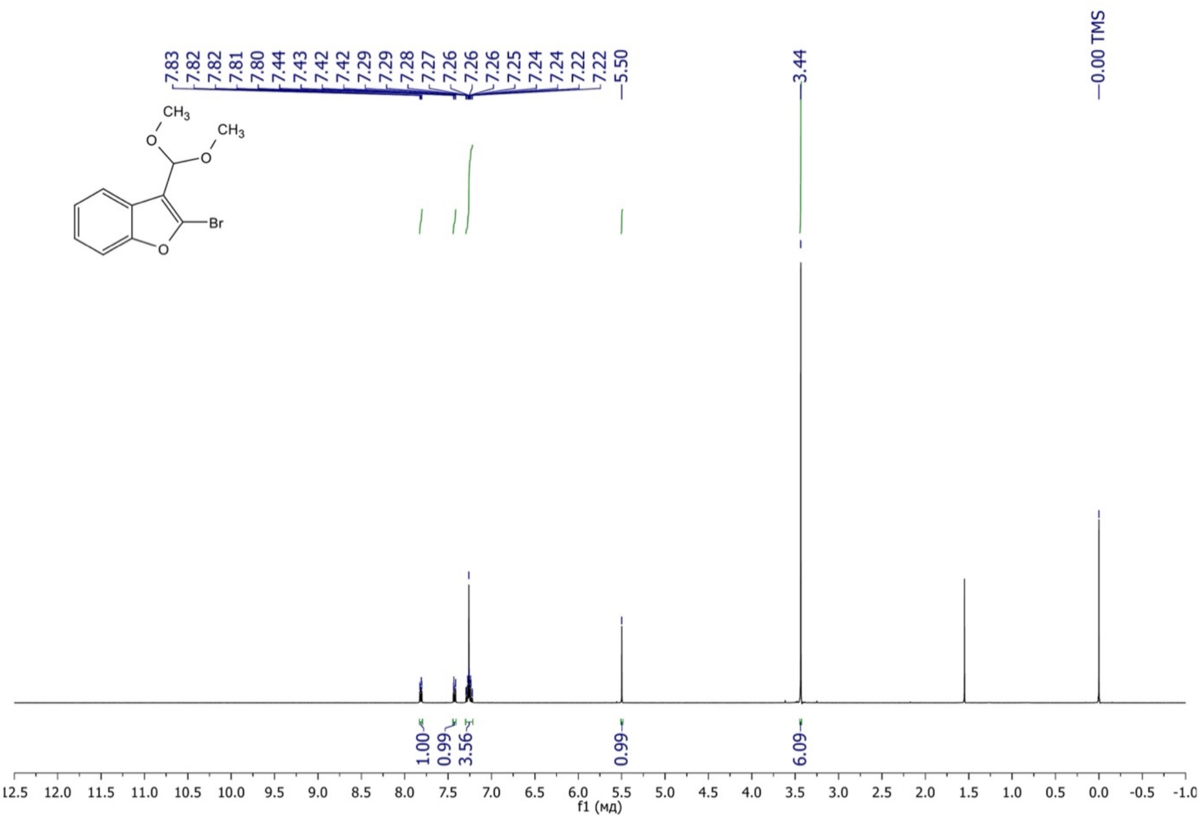

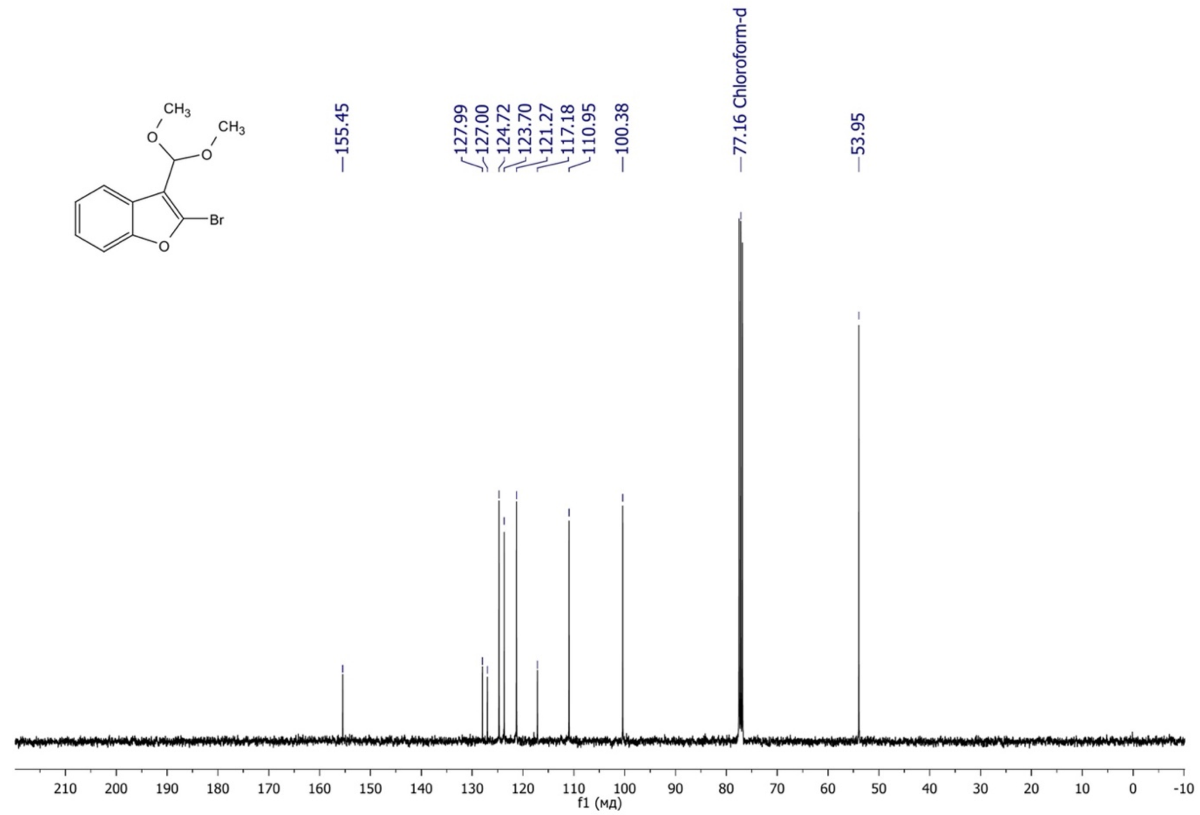

Supplementary Figure 41. ^{1}H, and ^{13}C NMR spectra of 2-Bromo-3-(dimethoxymethyl)-1-benzofuran

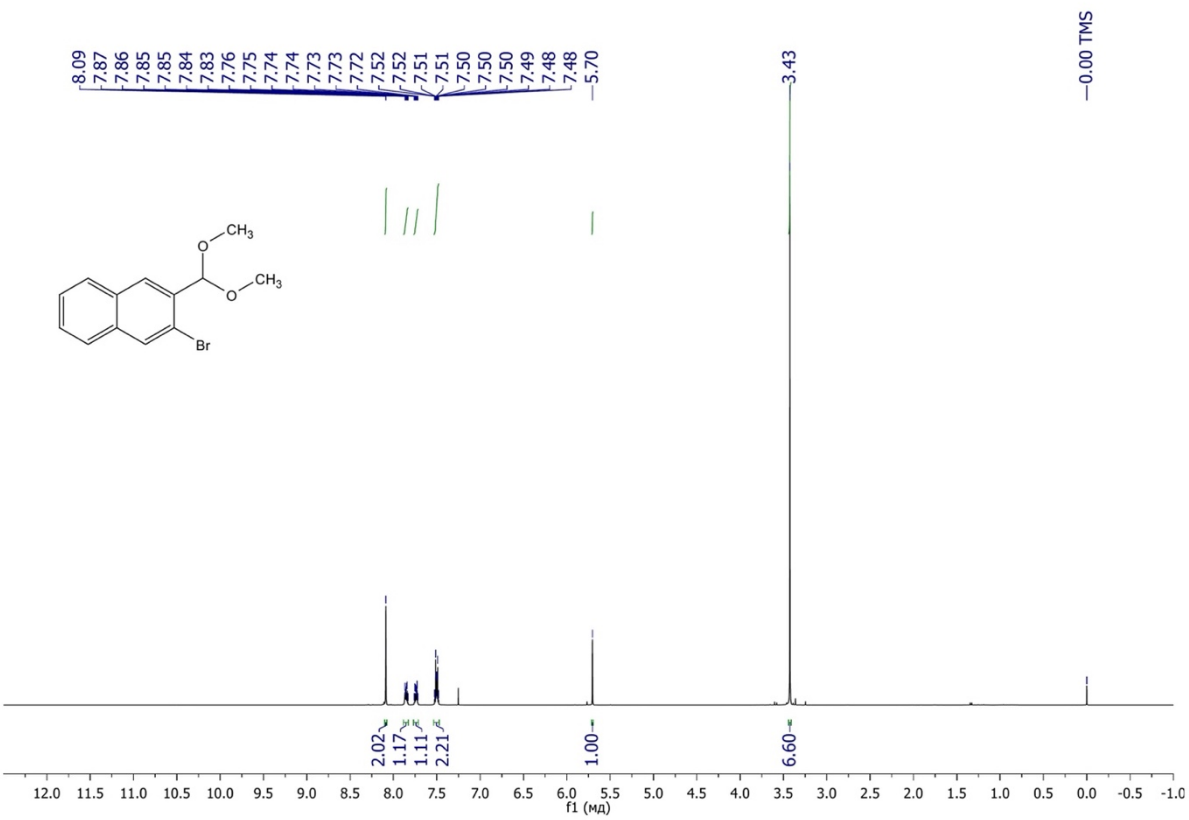

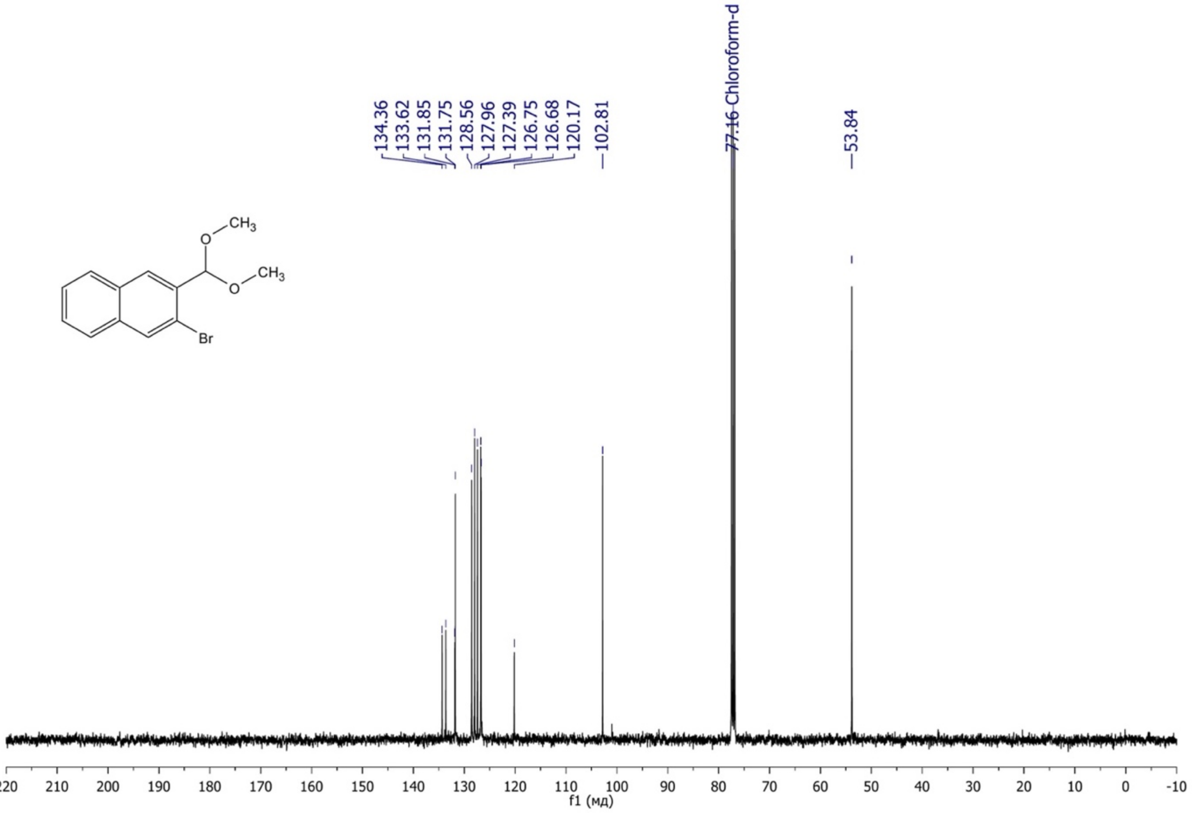

Supplementary Figure 42. ^{1}H, and ^{13}C NMR spectra of 2-Bromo-3-(dimethoxymethyl)naphthalene

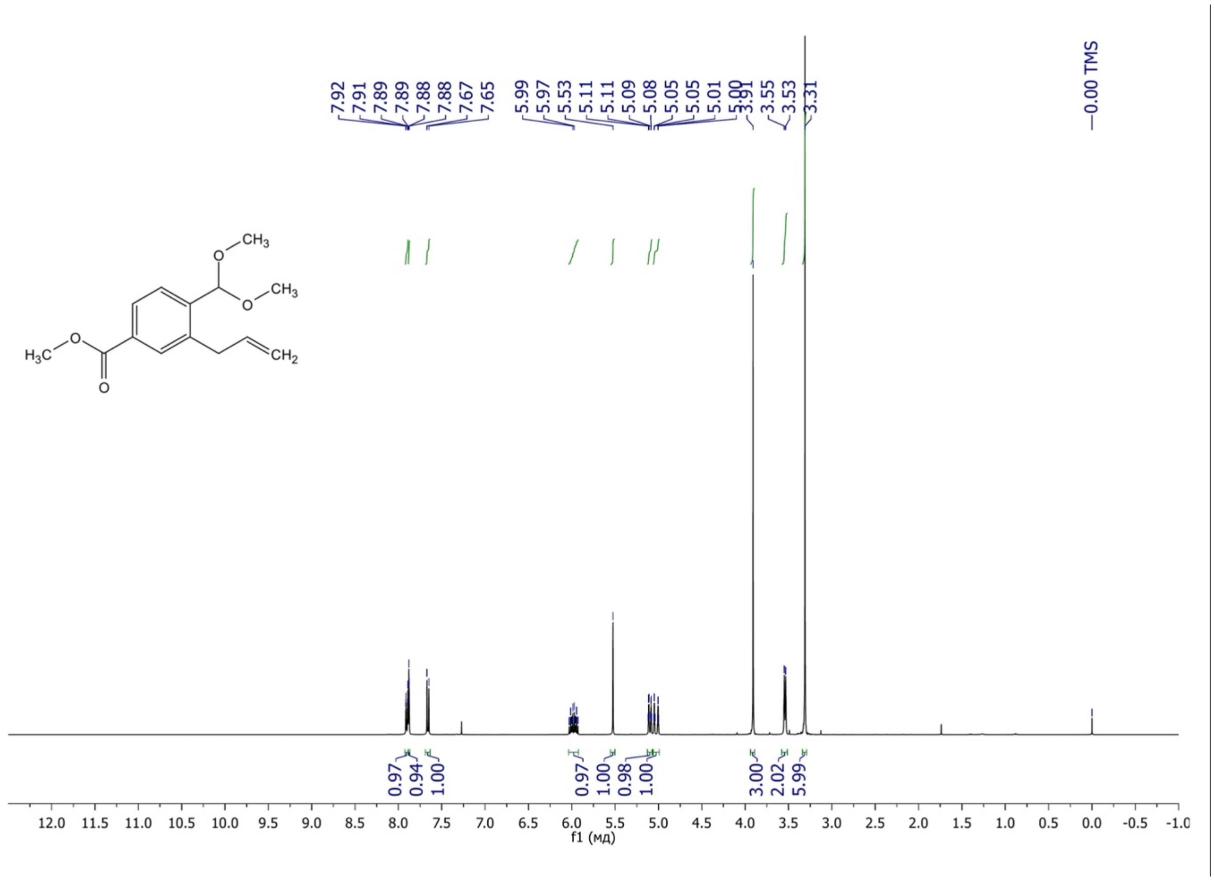

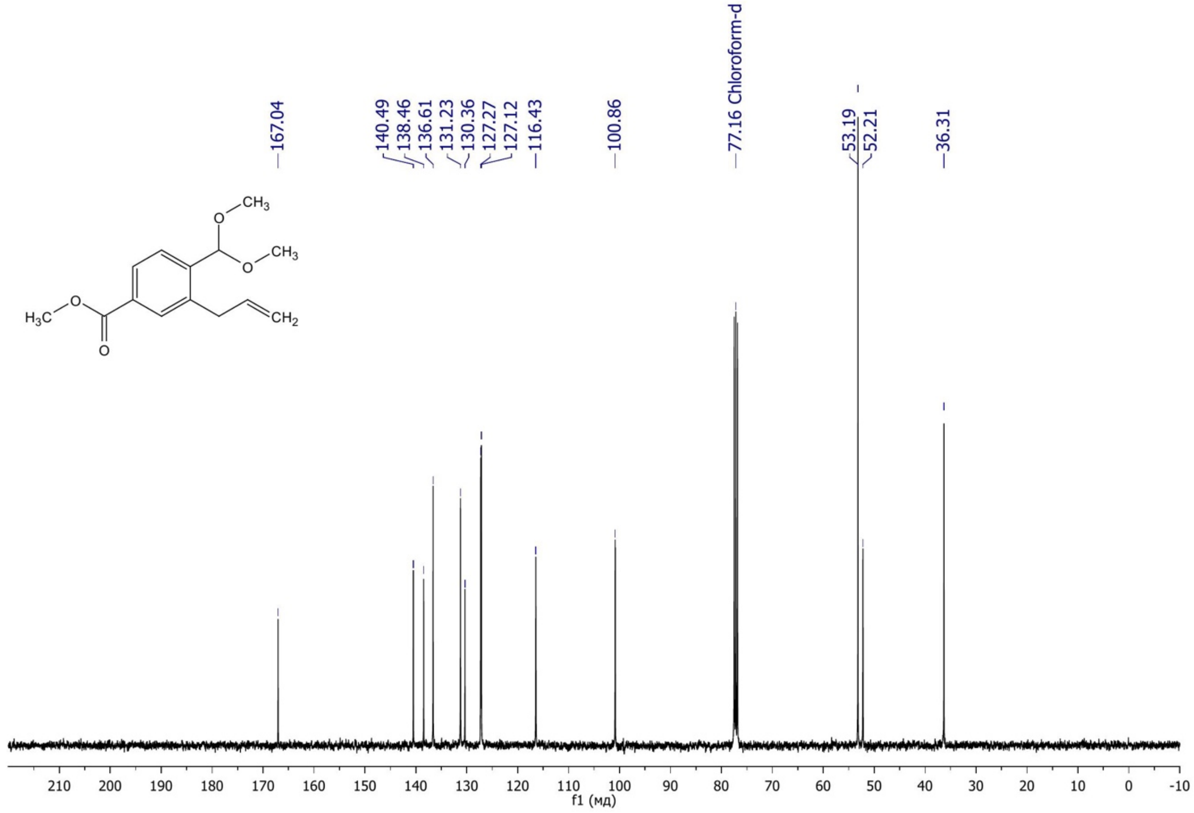

Supplementary Figure 43. ^{1}H, and ^{13}C NMR spectra of methyl 3-allyl-4-(dimethoxymethyl)benzoate

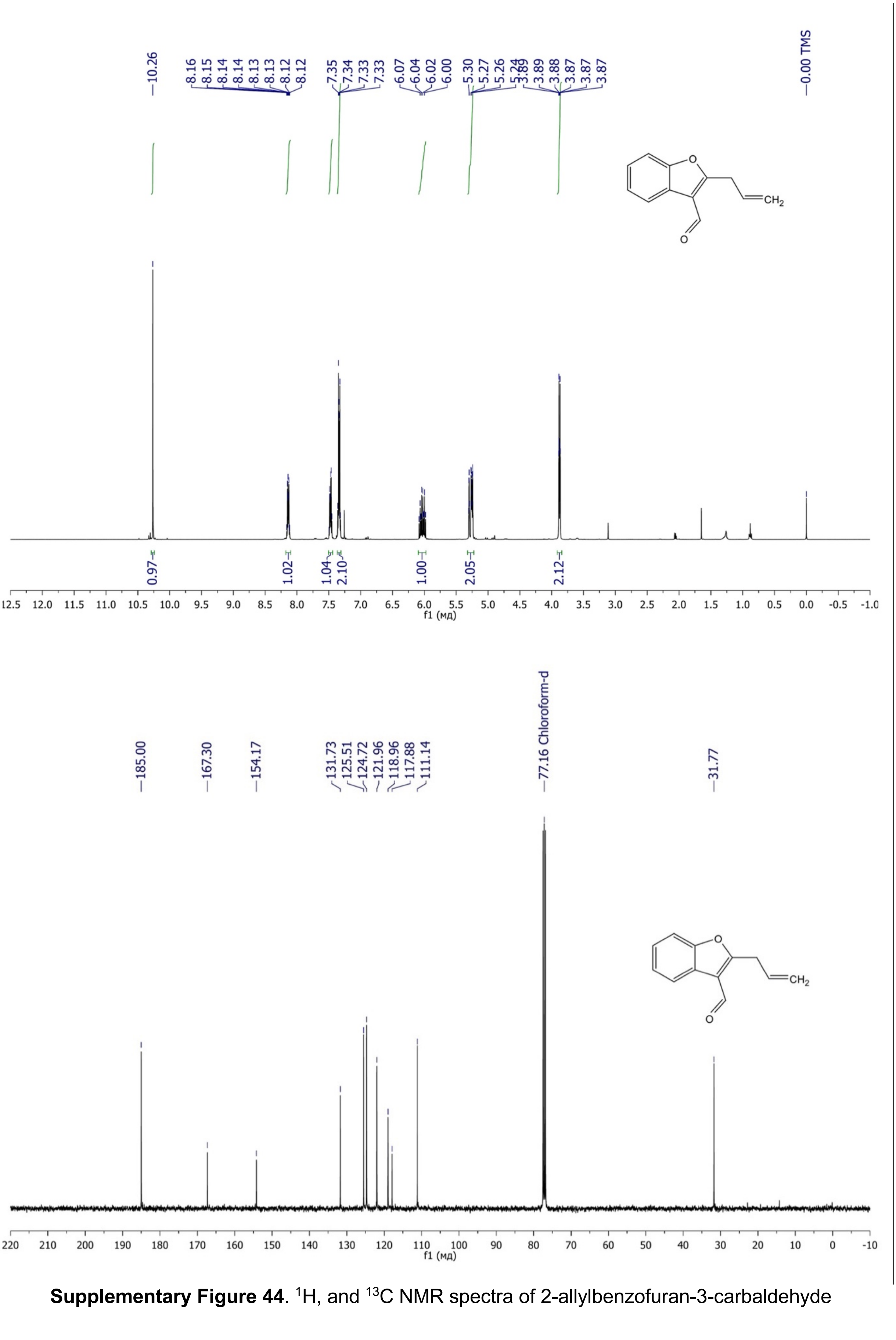

Supplementary Figure 44. ^{1}H, and ^{13}C NMR spectra of 2-allylbenzofuran-3-carbaldehyde

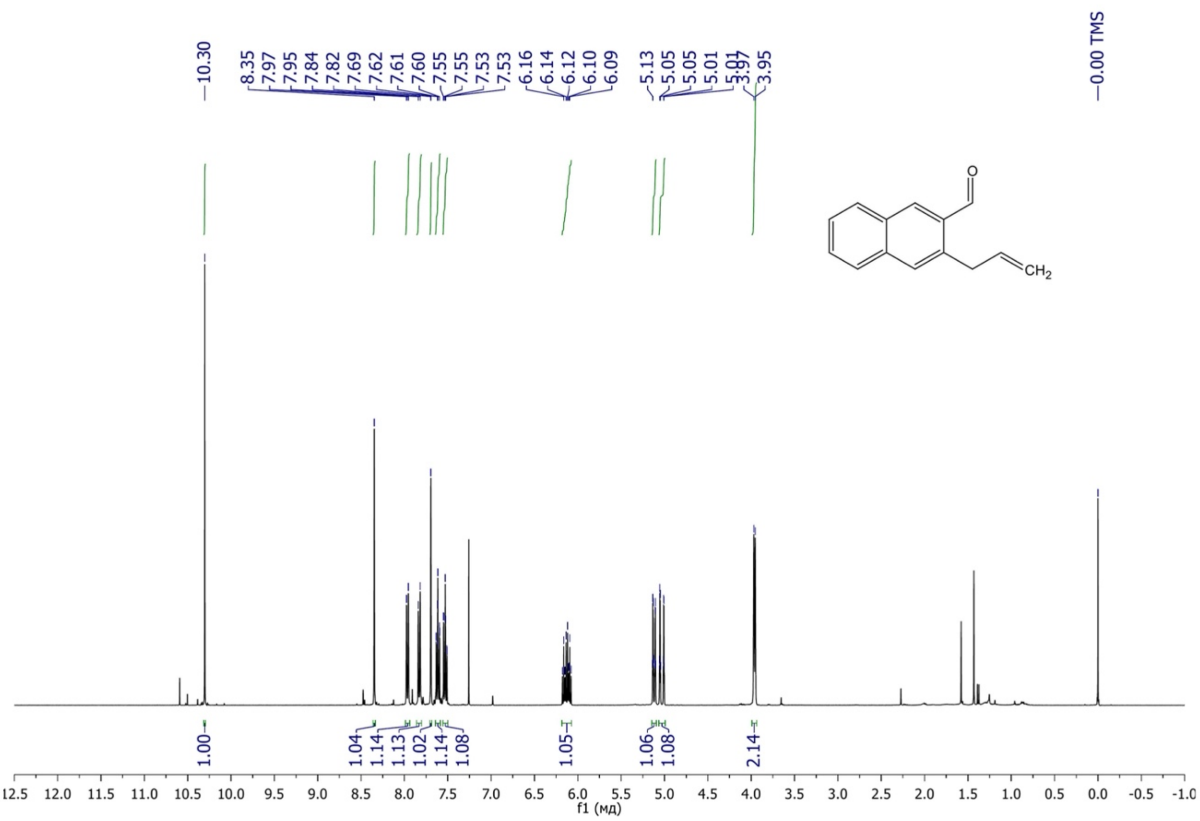

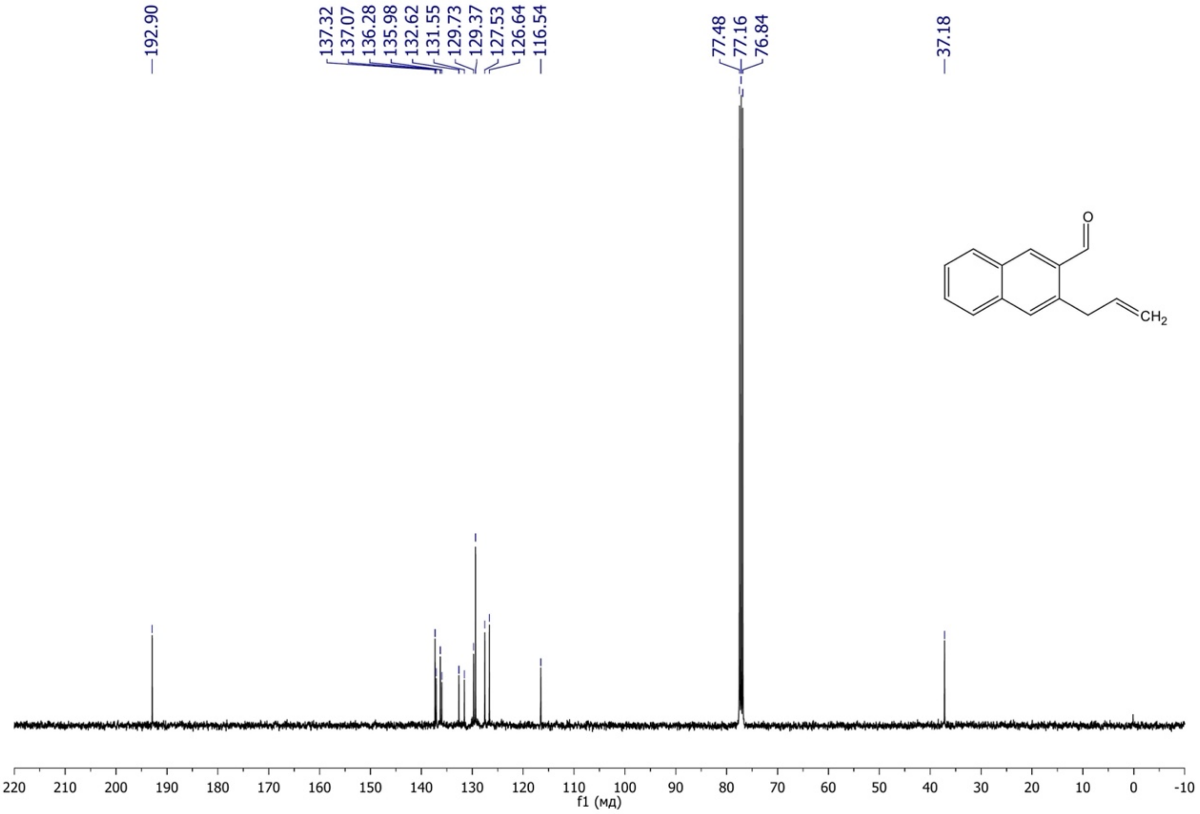

Supplementary Figure 45. ^{1}H, and ^{13}C NMR spectra of 3-allyl-2-naphthaldehyde

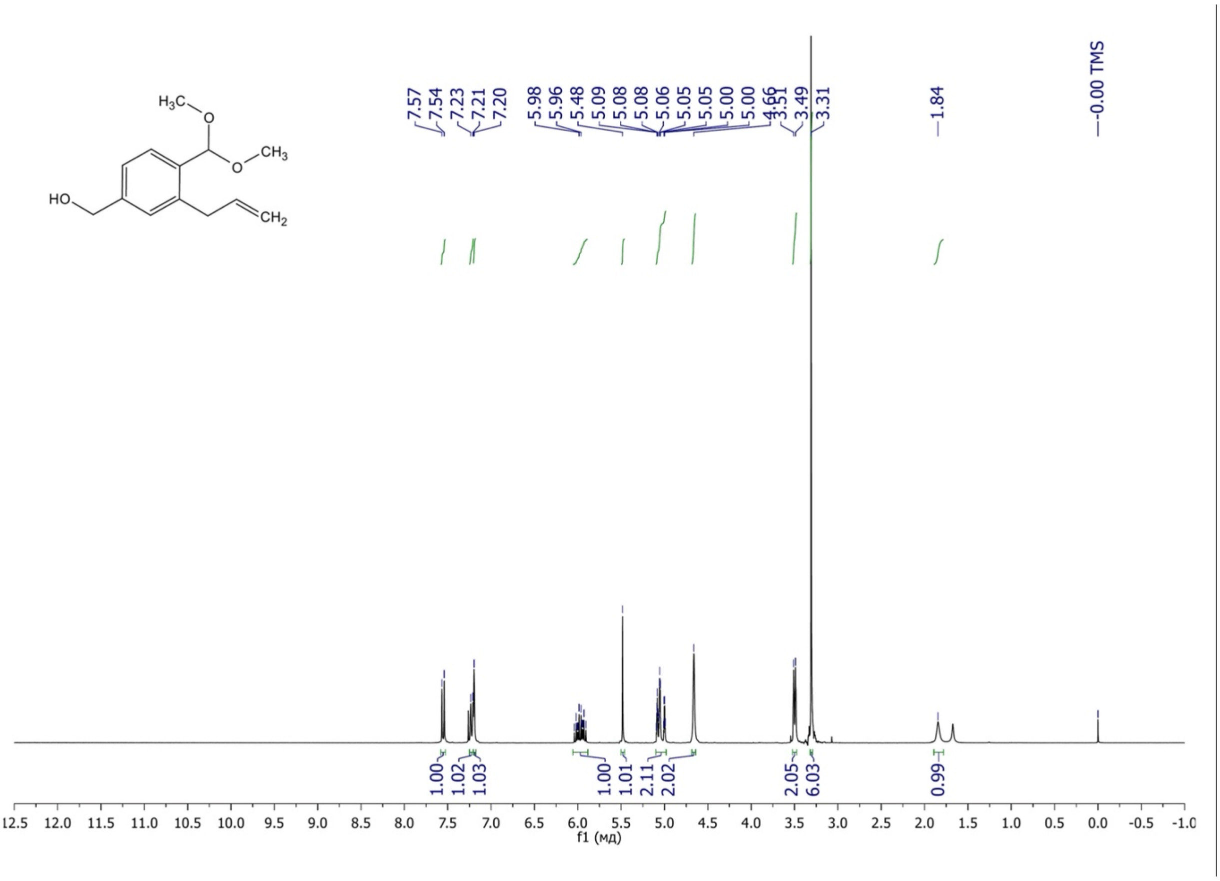

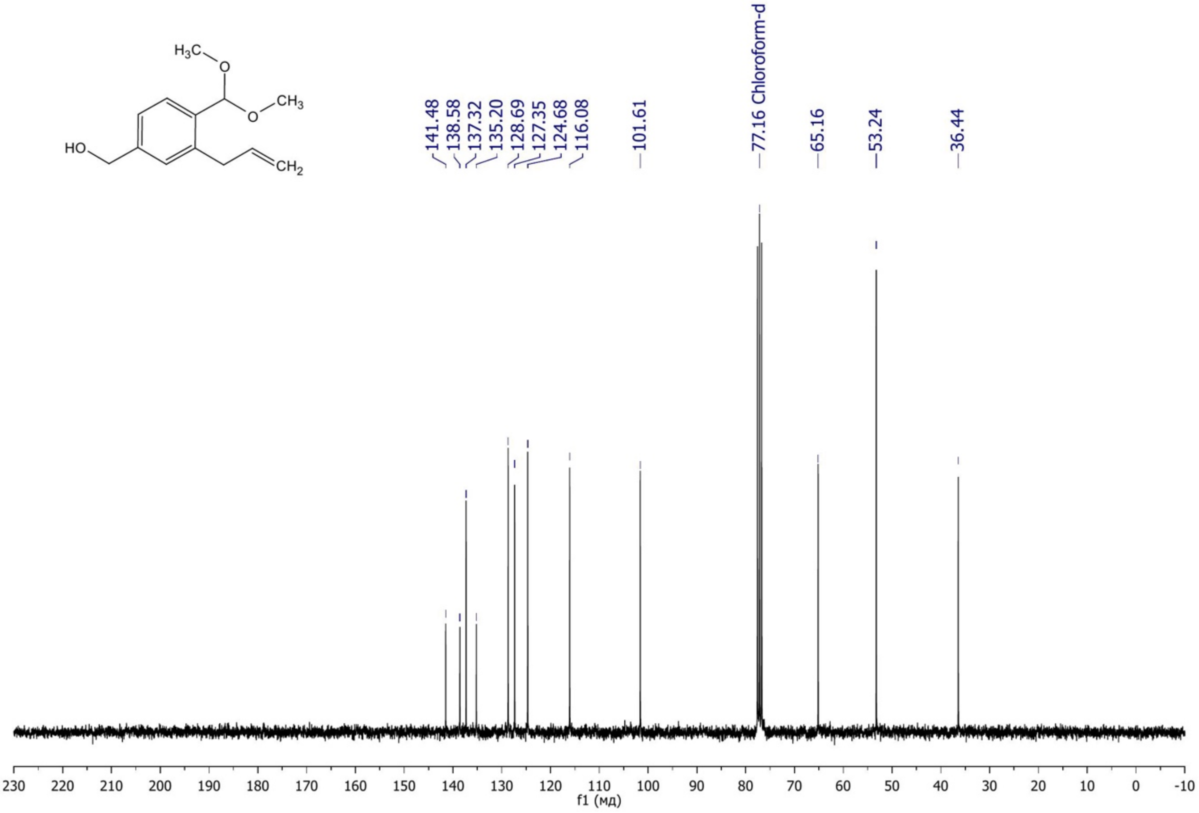

Supplementary Figure 46. ^{1}H, and ^{13}C NMR spectra of (3-allyl-4-(dimethoxymethyl)phenyl)methanol

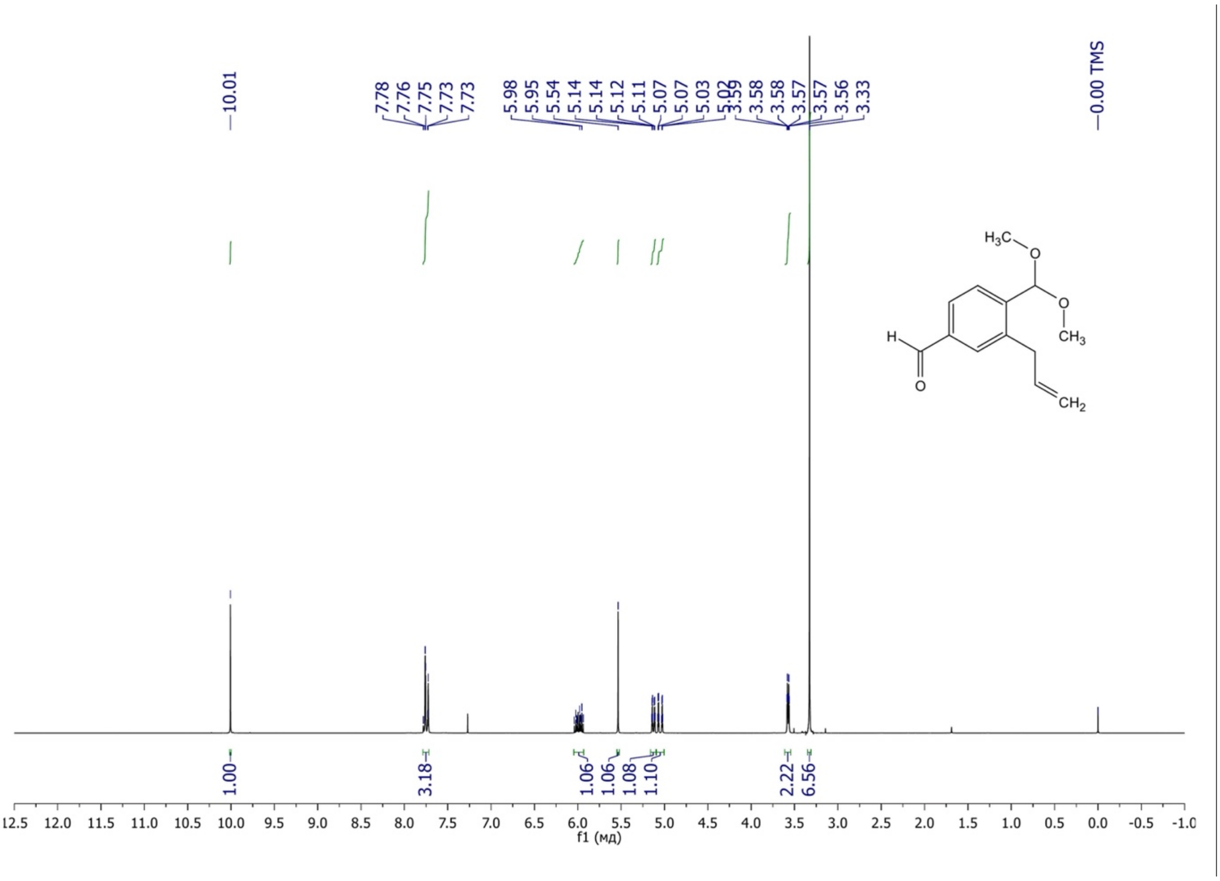

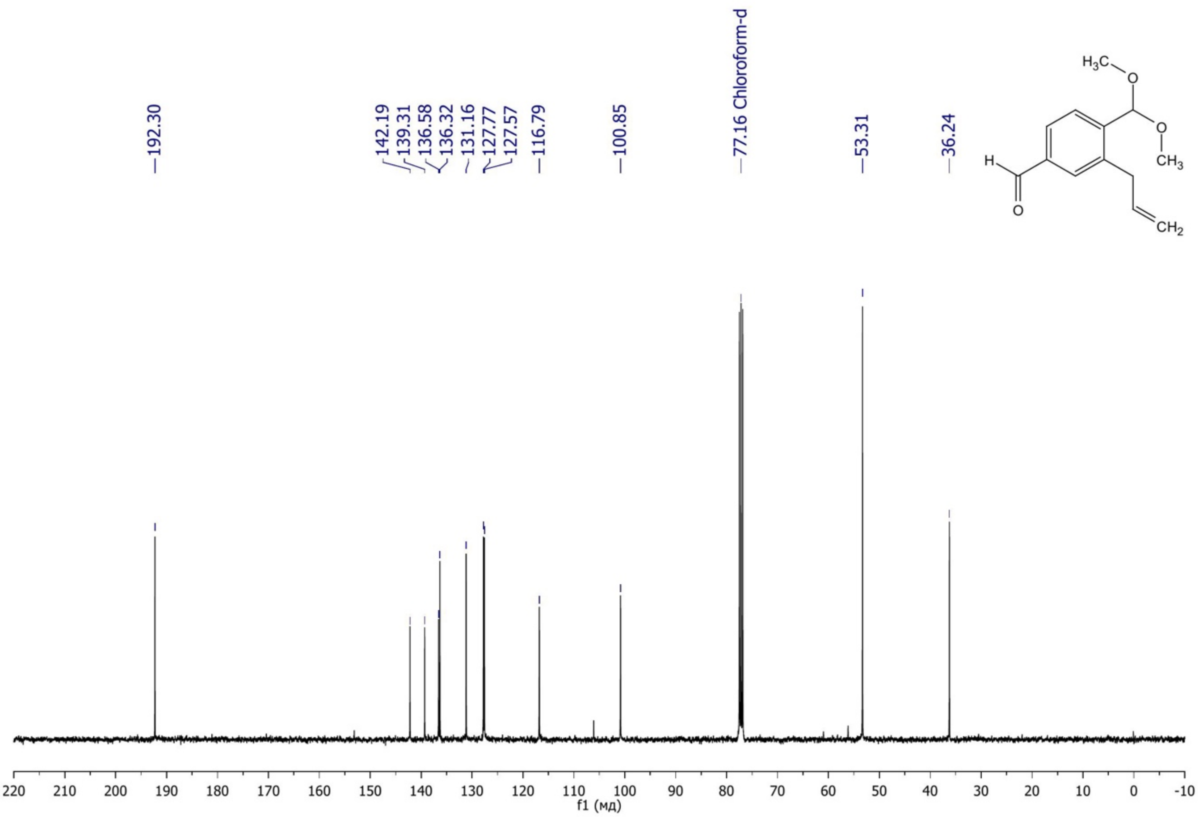

Supplementary Figure 47. ^{1}H, and ^{13}C NMR spectra of 3-allyl-4-(dimethoxymethyl)benzaldehyde

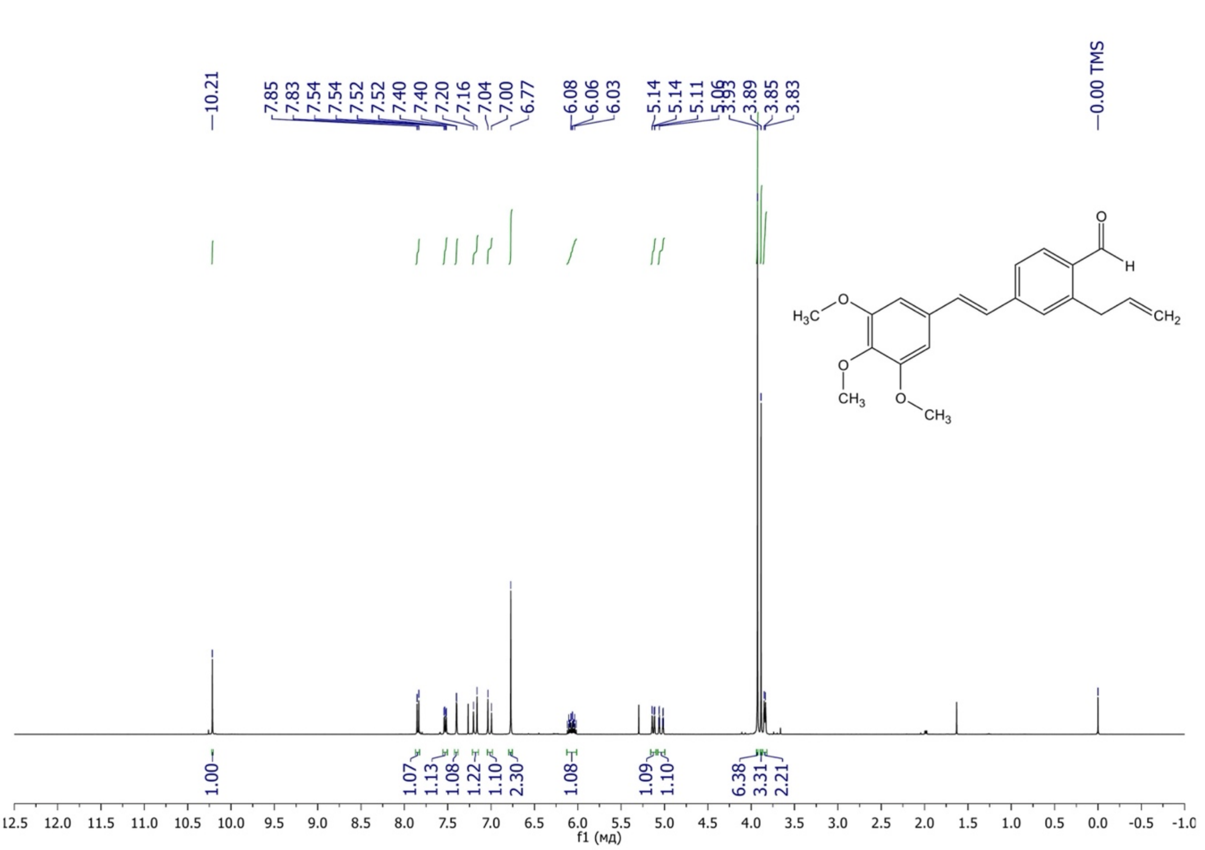

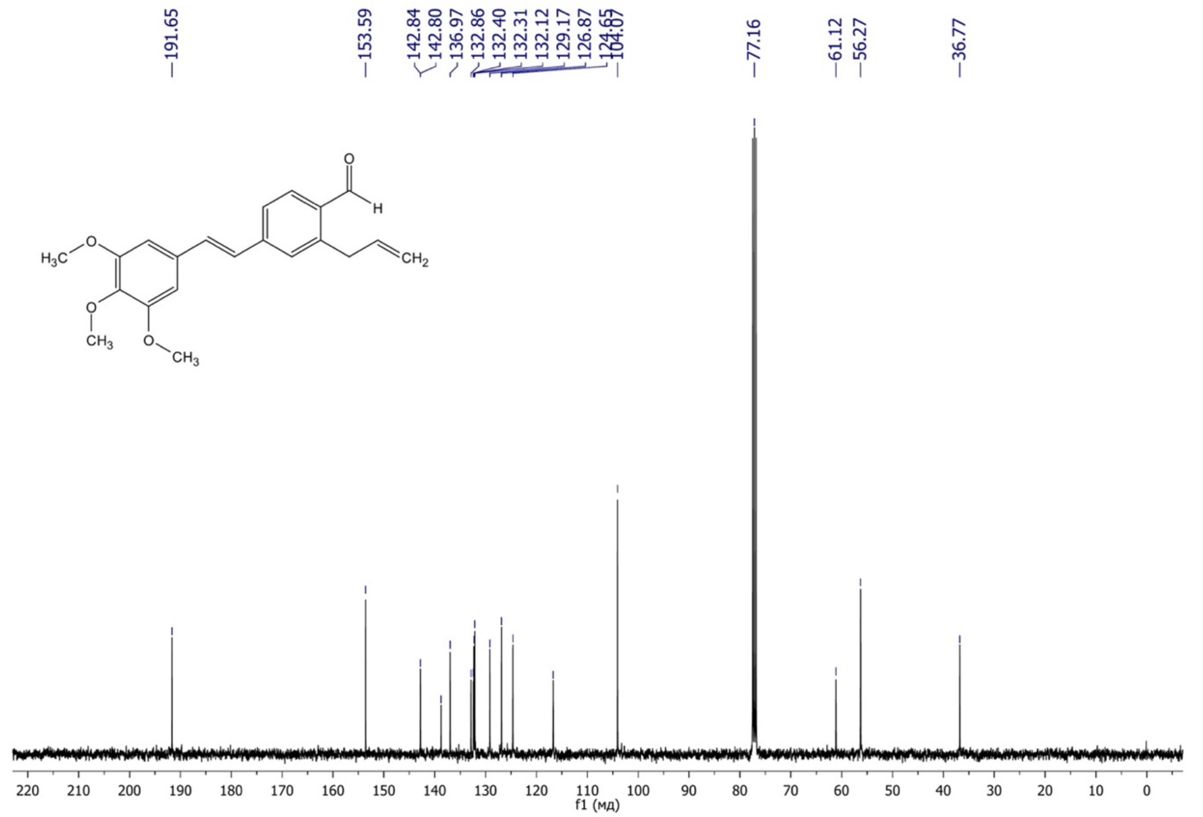

Supplementary Figure 48. ^{1}H, and ^{13}C NMR spectra of *E*-2-allyl-4-(3,4,5-trimethoxystyryl)benzaldehyde

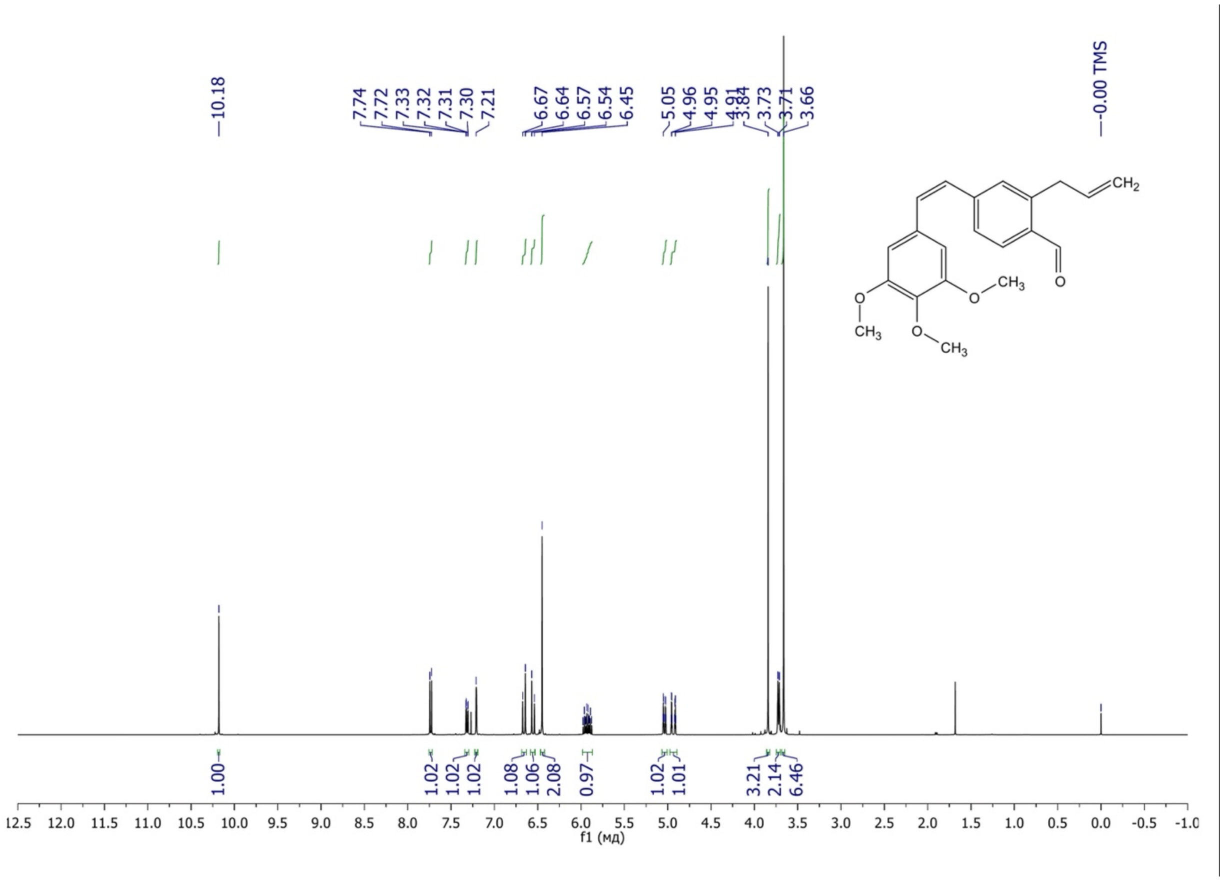

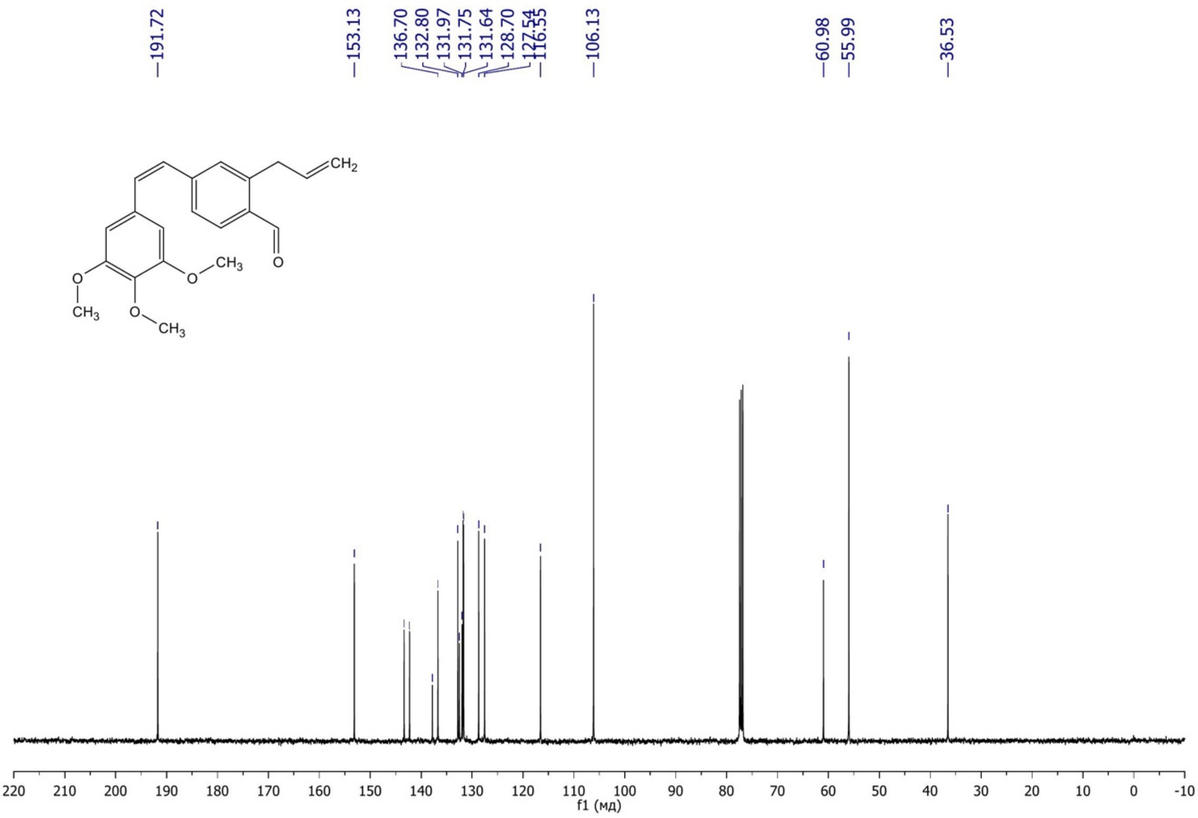

Supplementary Figure 49. ^{1}H, and ^{13}C NMR spectra of *Z*-2-allyl-4-(3,4,5-trimethoxystyryl)benzaldehyde

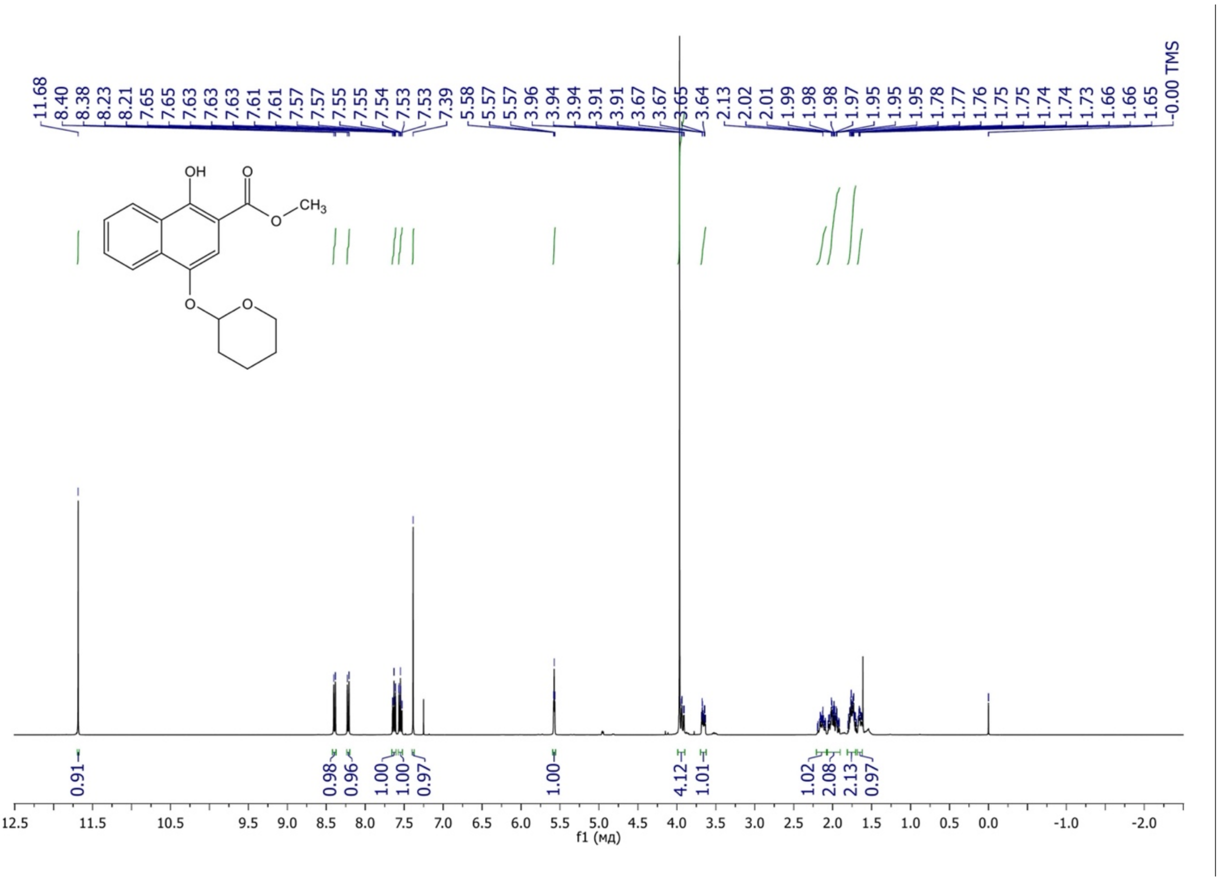

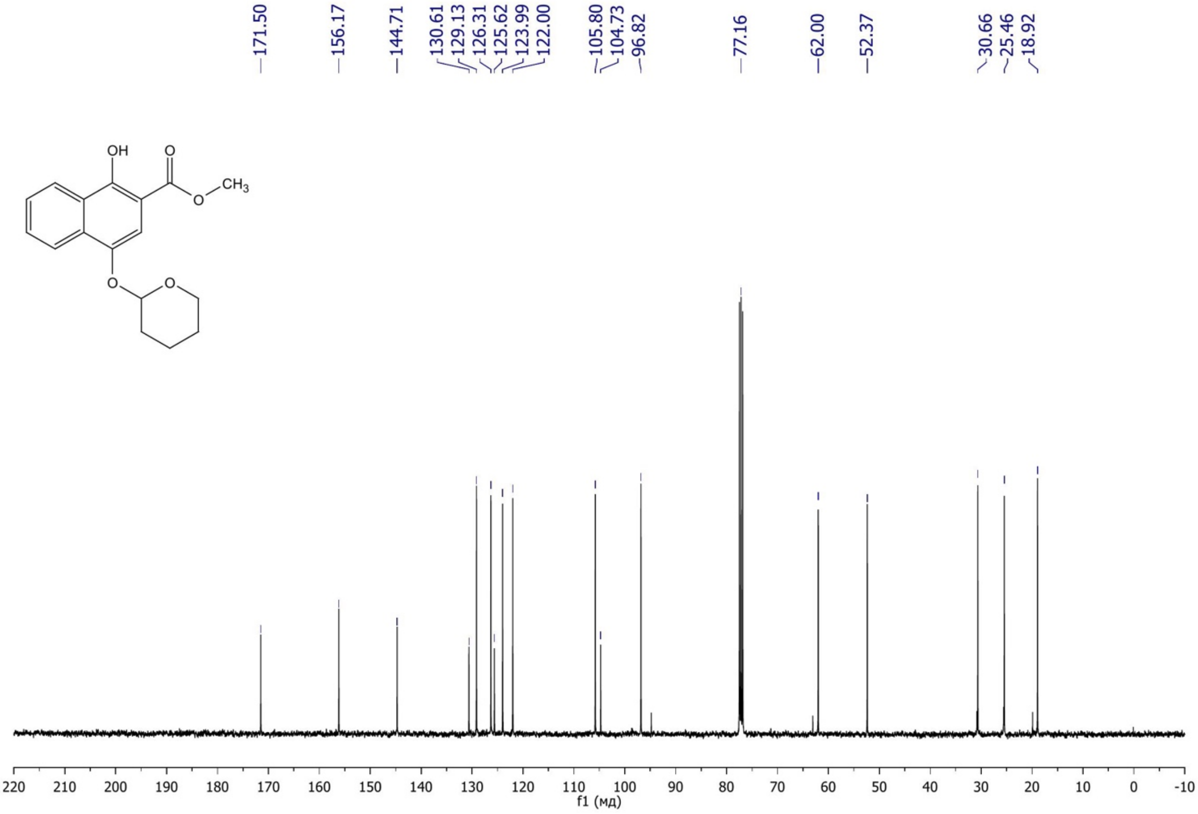

Supplementary Figure 50. ^{1}H, and ^{13}C NMR spectra of methyl 1-hydroxy-4-((tetrahydro-2H-pyran-2-yl)oxy)-2-naphthoate

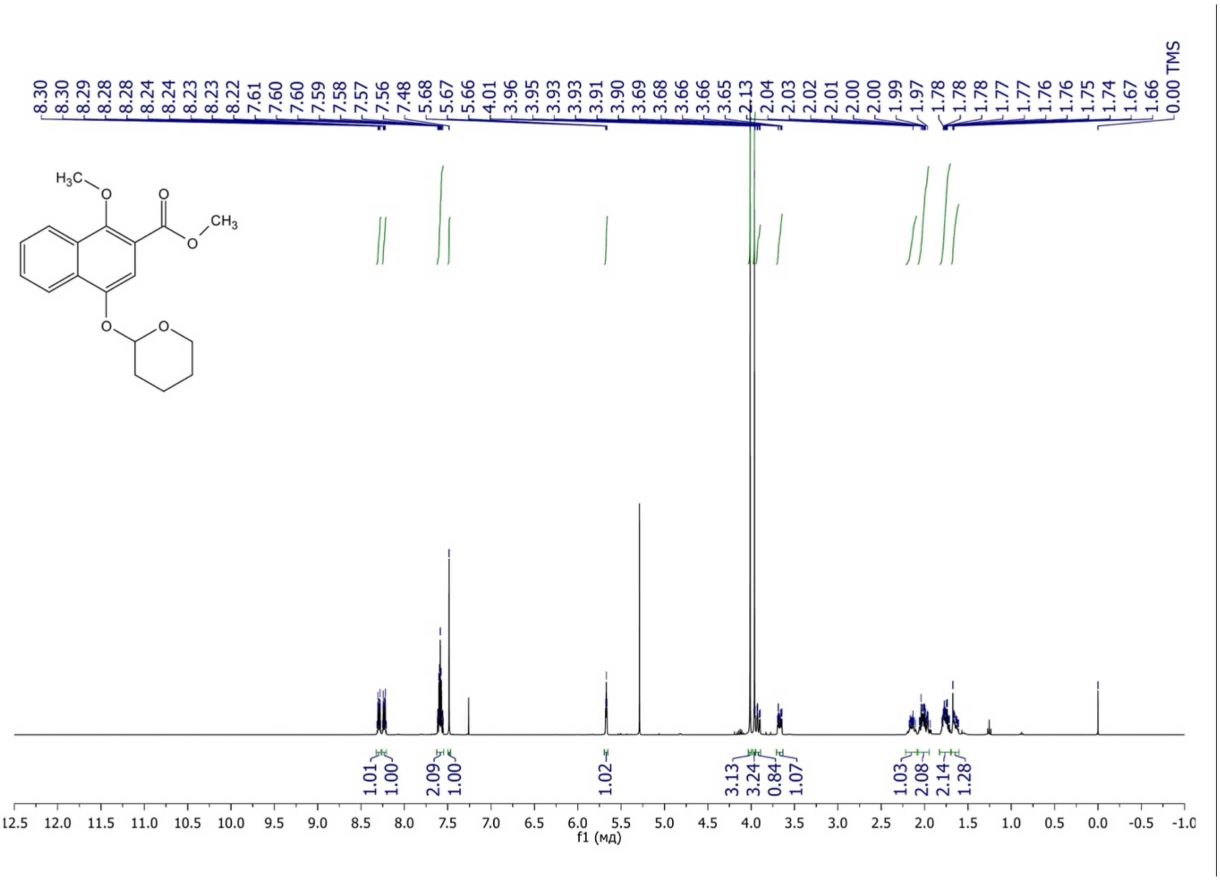

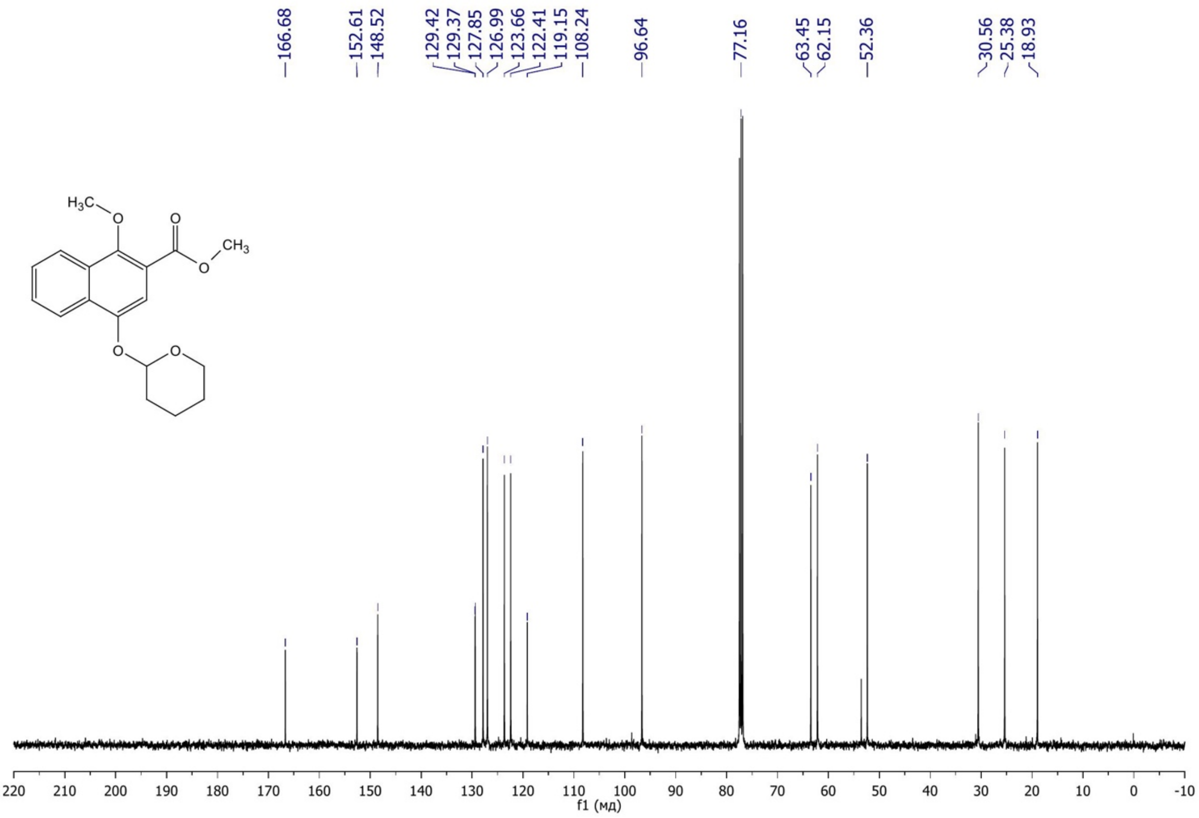

Supplementary Figure 51. ^{1}H, and ^{13}C NMR spectra of methyl 1-methoxy-4-((tetrahydro-2H-pyran-2-yl)oxy)-2-naphthoate

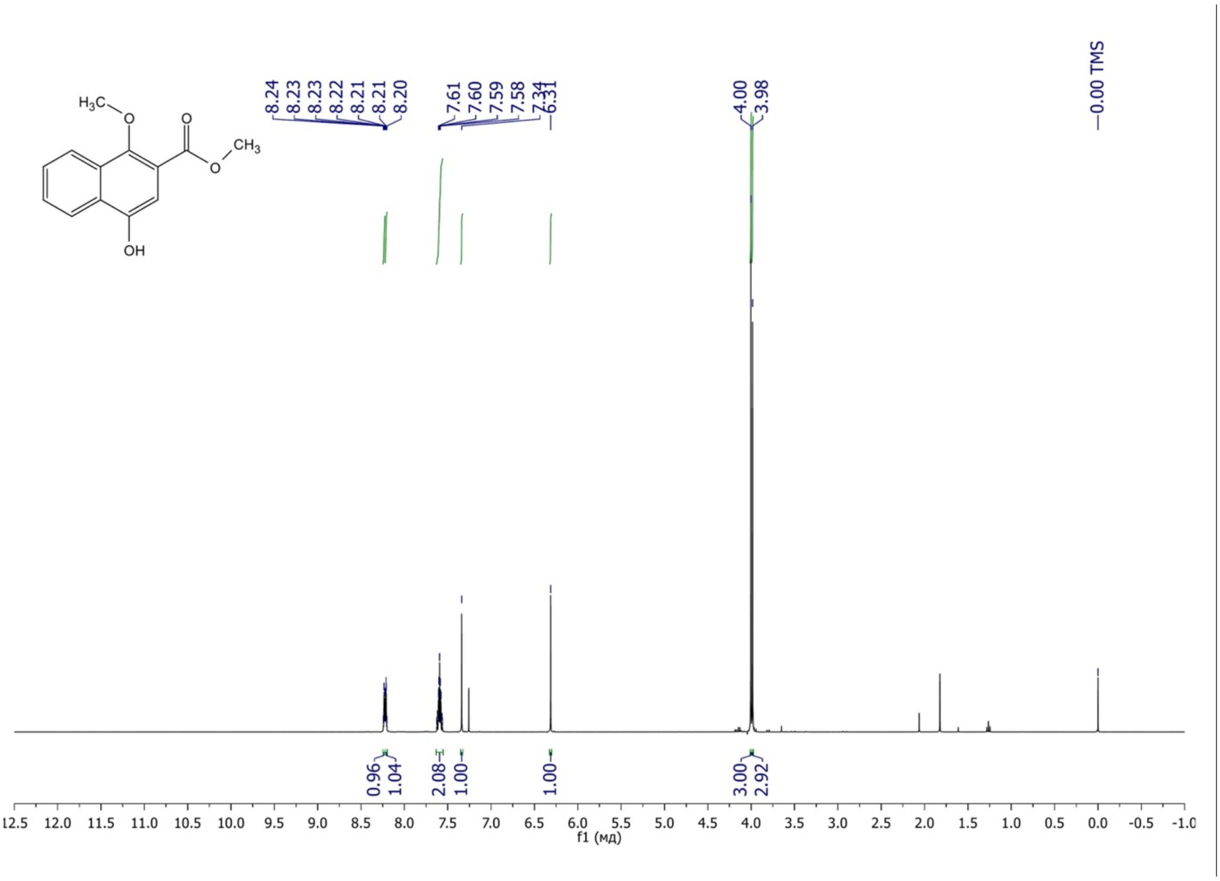

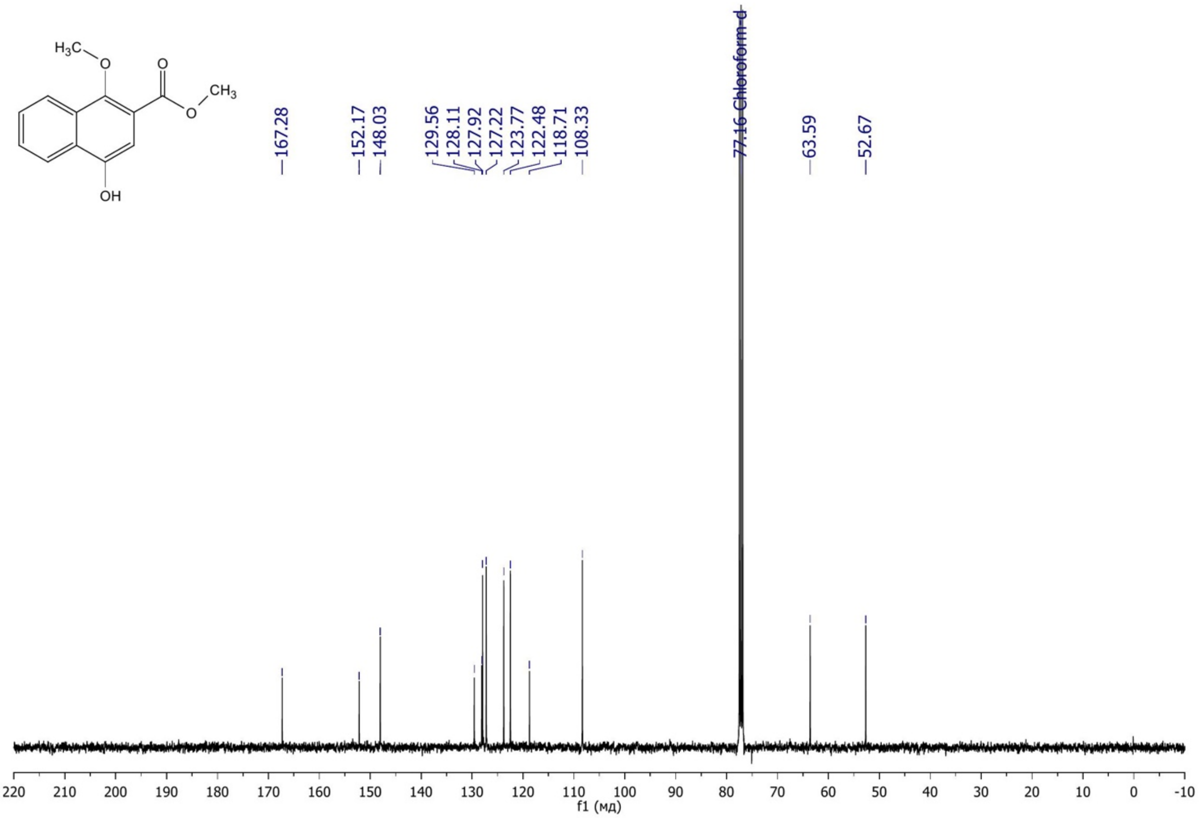

Supplementary Figure 52. ^{1}H, and ^{13}C NMR spectra of methyl 4-hydroxy-1-methoxy-2-naphthoate

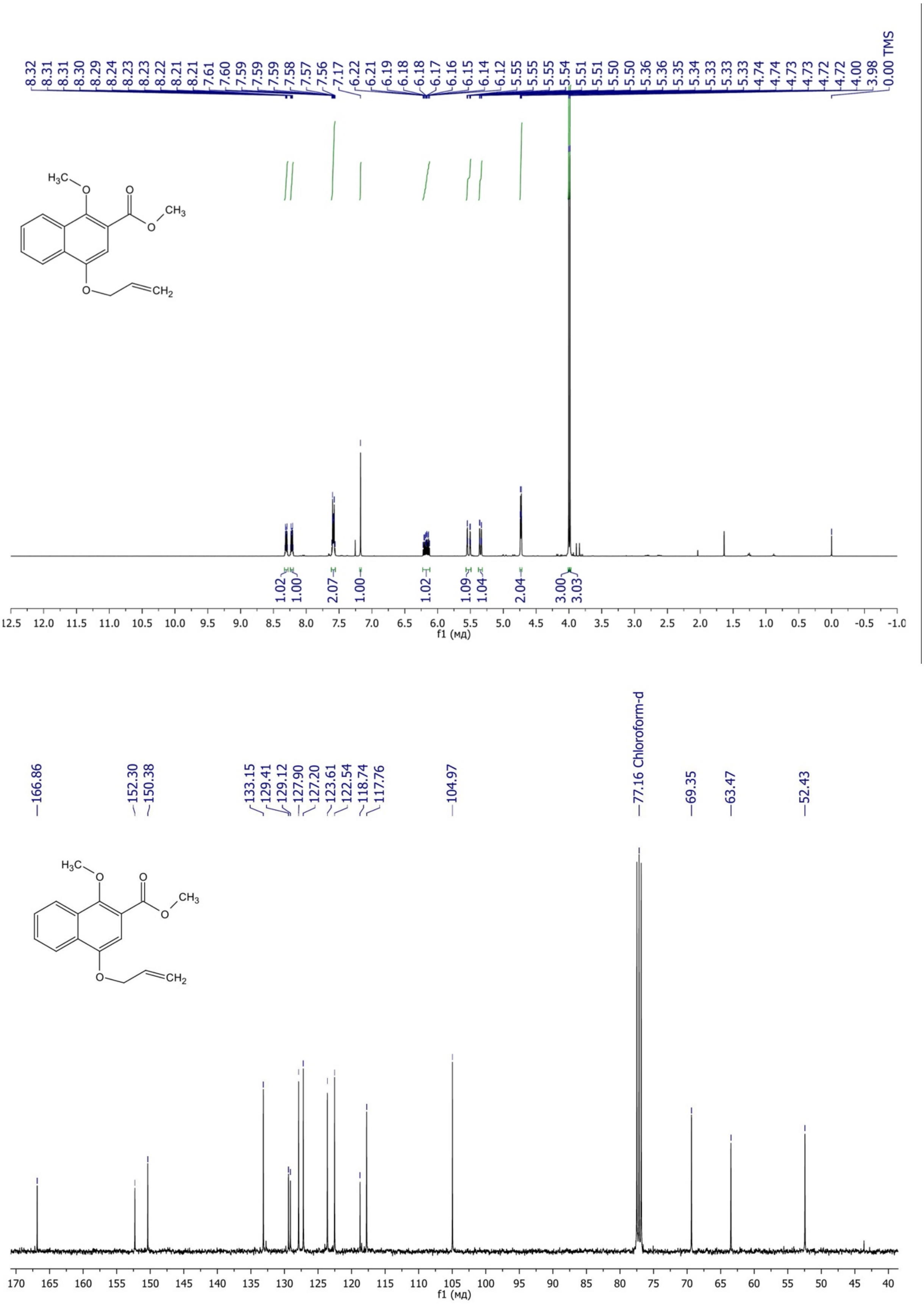

Supplementary Figure 53. ^1H, and ^{13}C NMR spectra of methyl 4-allyloxy-1-methoxy-2-naphthoate

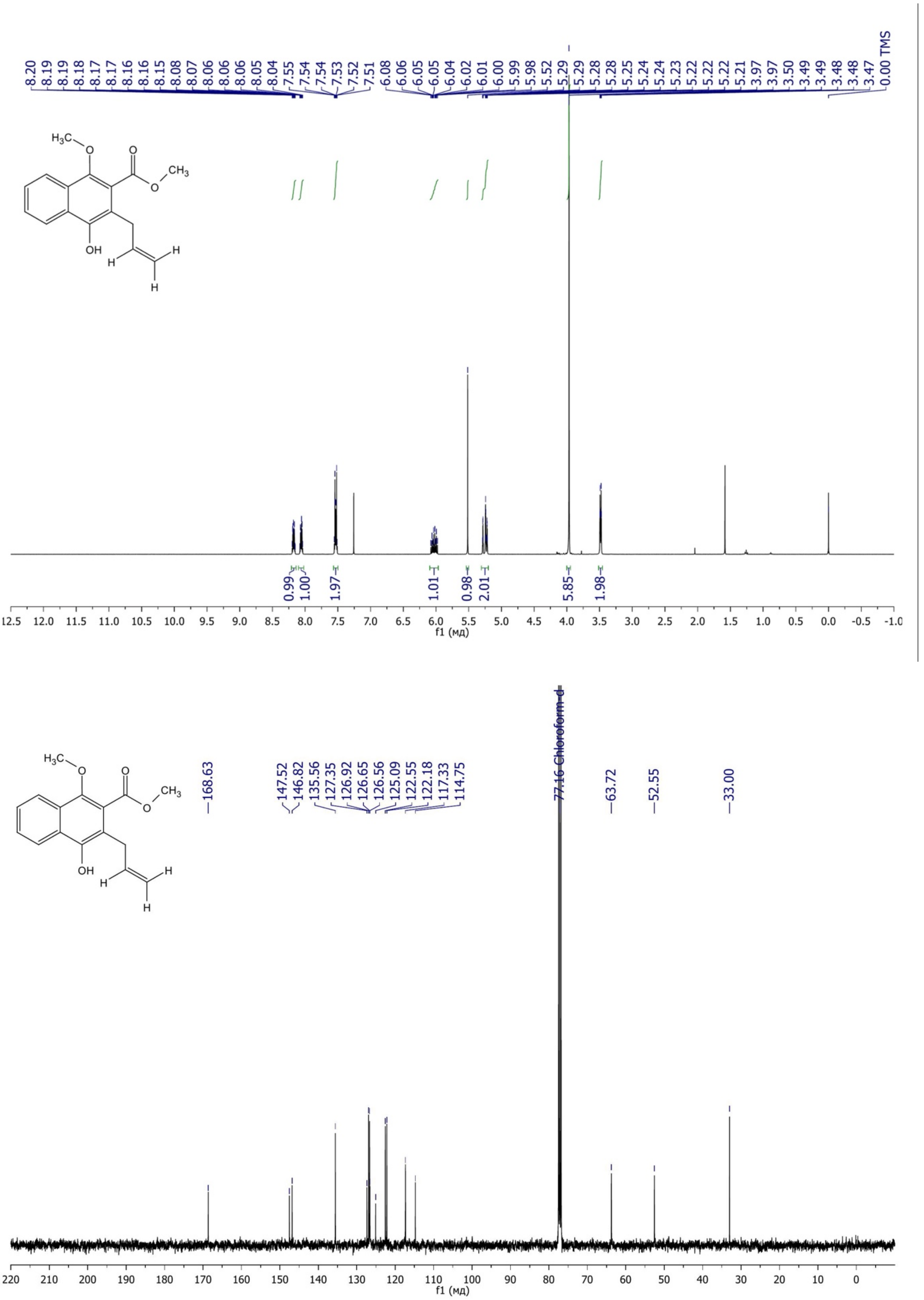

Supplementary Figure 54. ^{1}H, and ^{13}C NMR spectra of methyl 3-allyl-4-hydroxy-1-methoxy-2-naphthoate

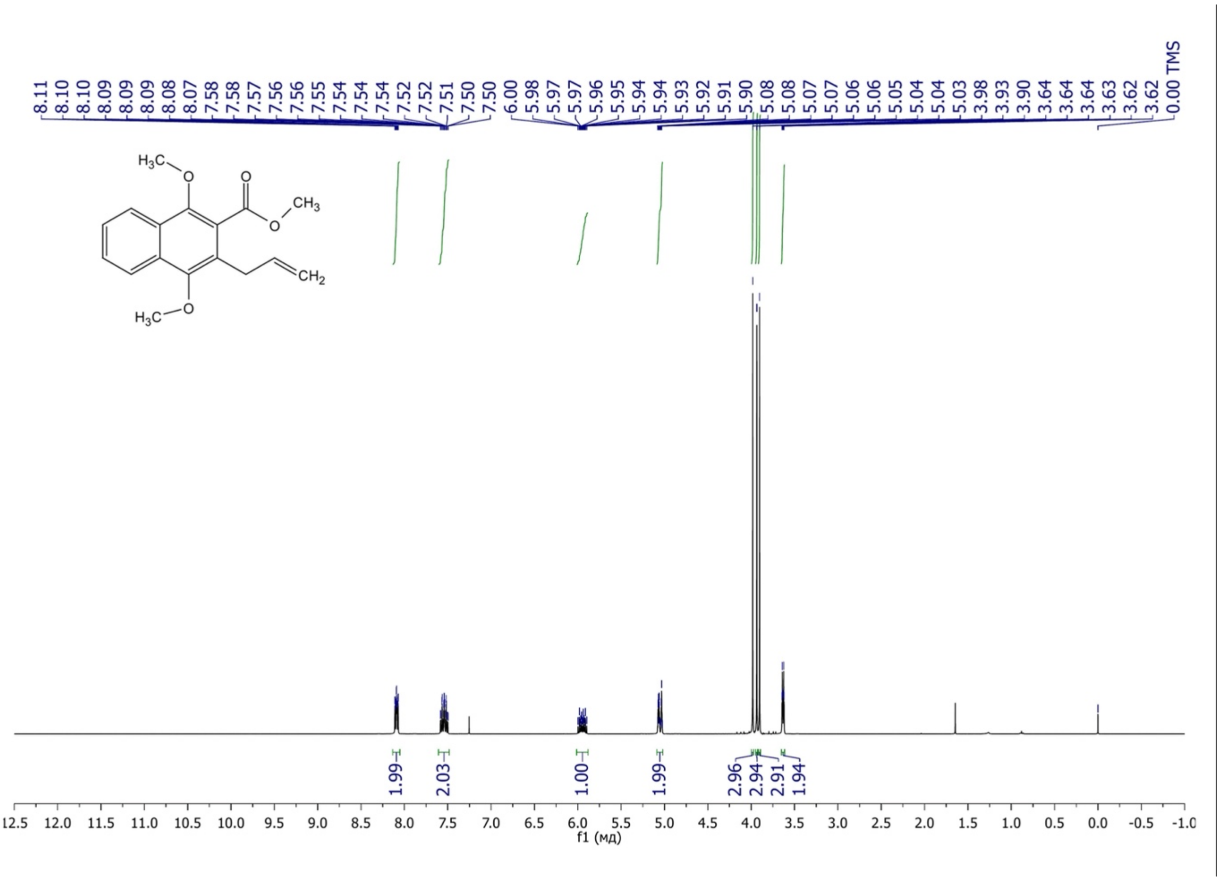

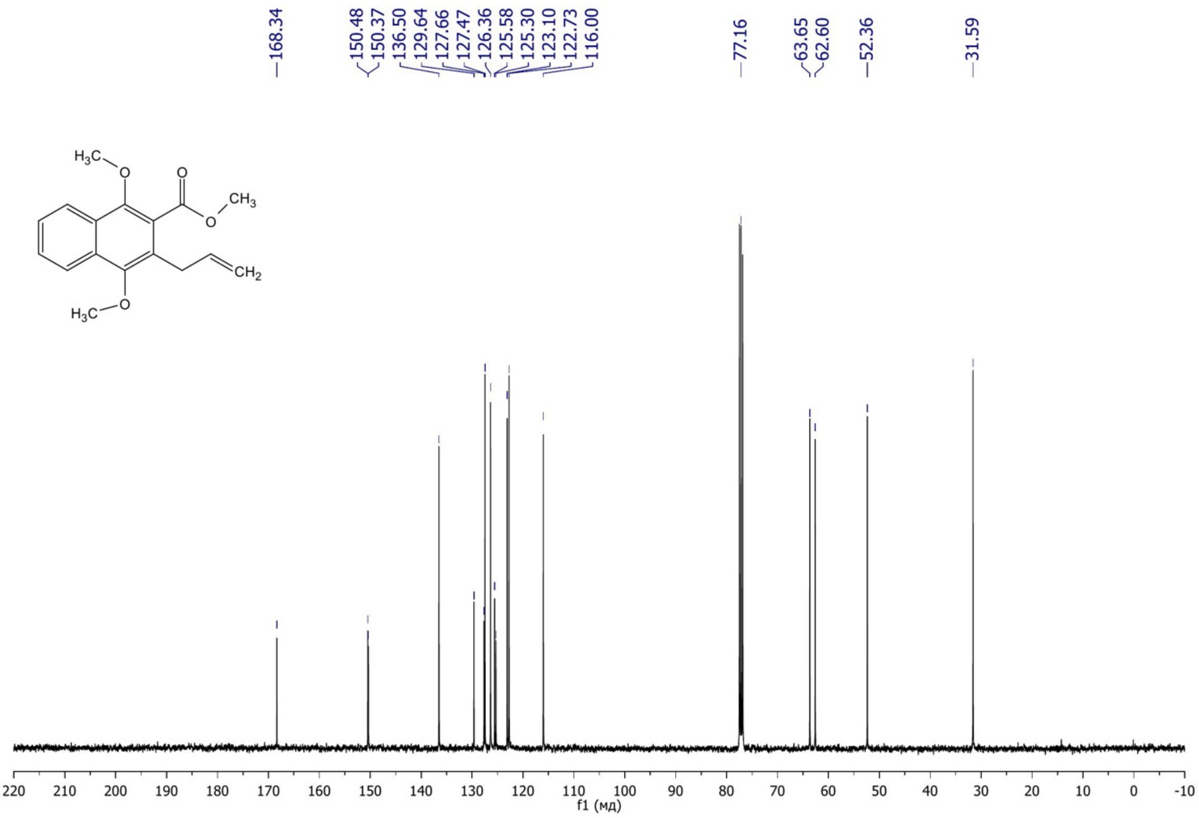

Supplementary Figure 55. ^{1}H, and ^{13}C NMR spectra of methyl 3-allyl-1,4-dimethoxy-2-naphtoate

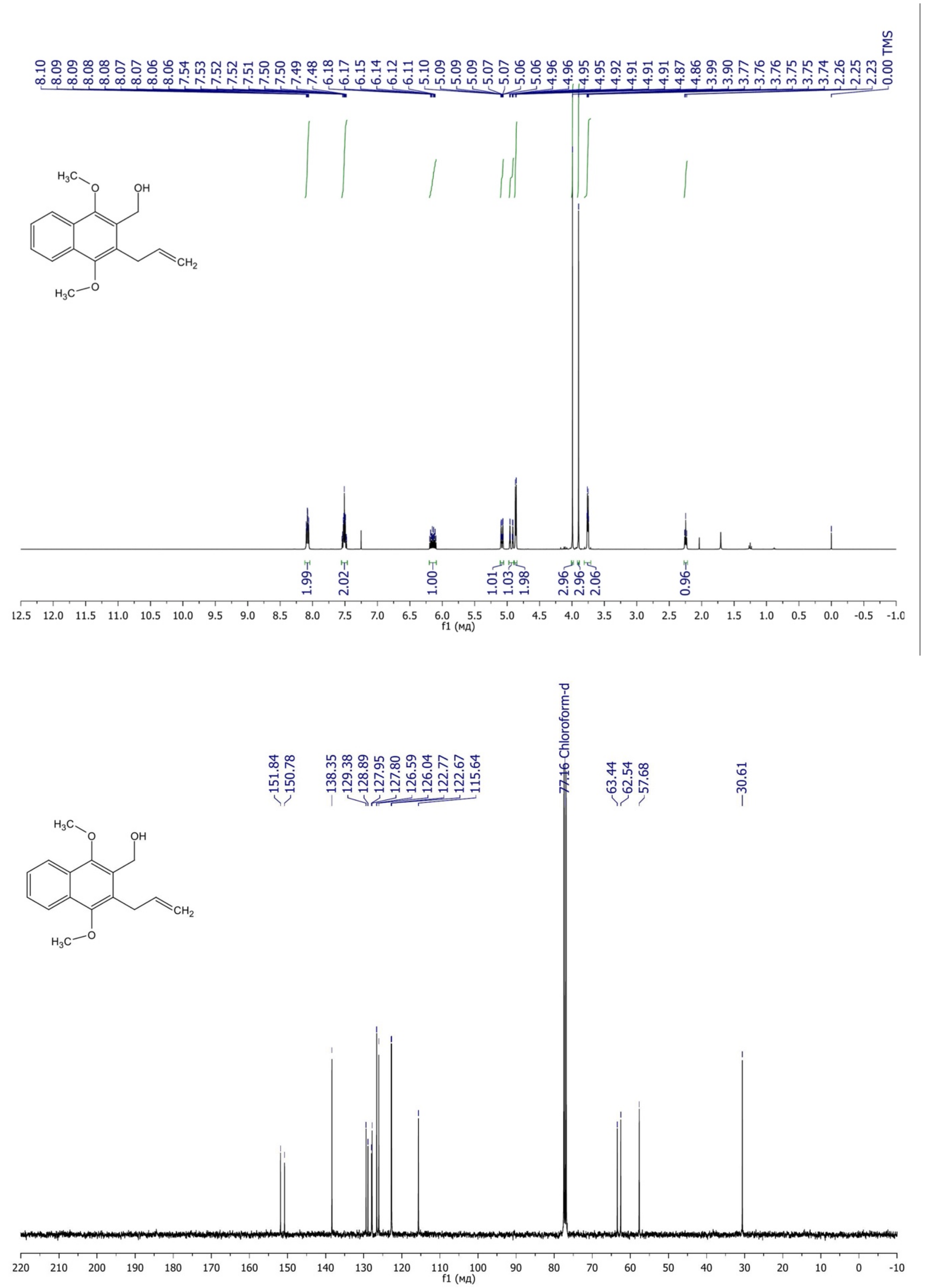

Supplementary Figure 56. ^{1}H, and ^{13}C NMR spectra of (3-allyl-1,4-dimethoxynaphthalen-2-yl)methanol

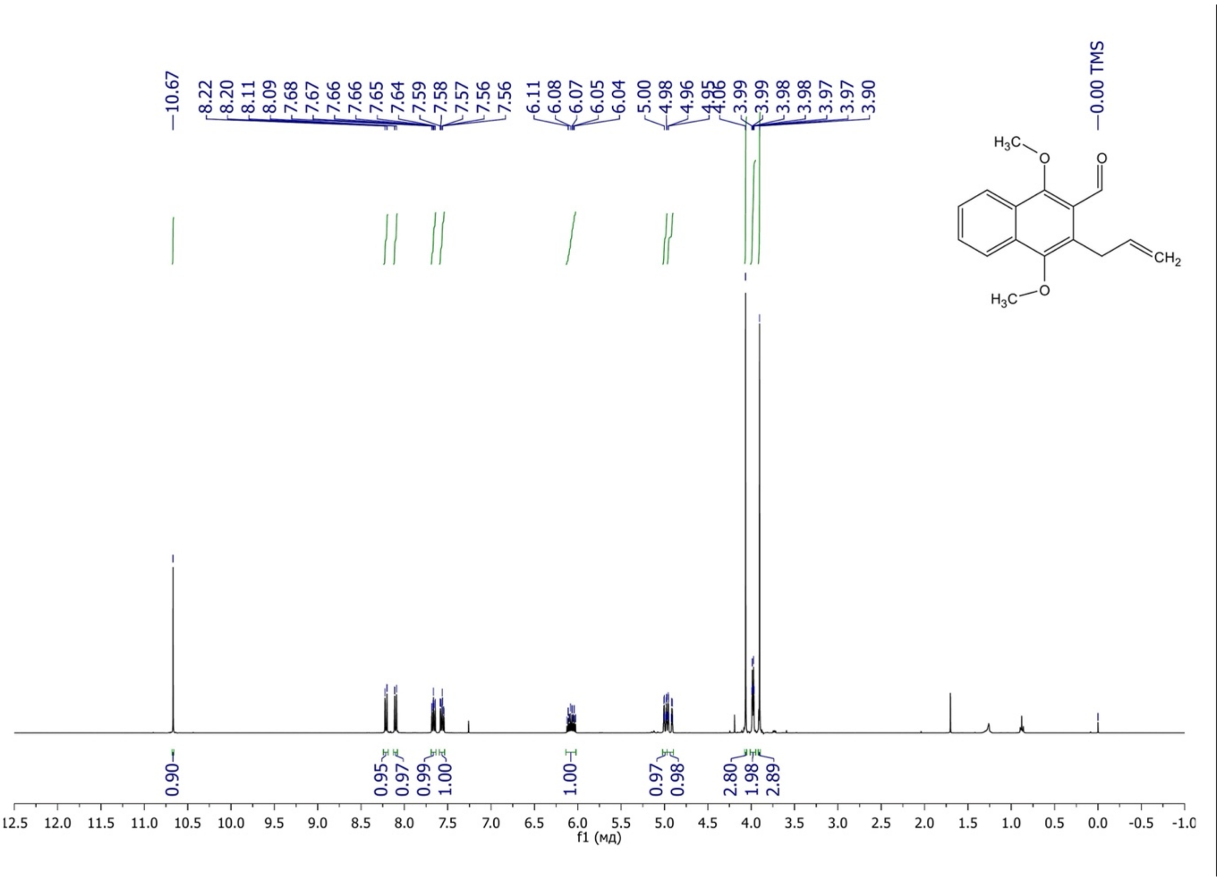

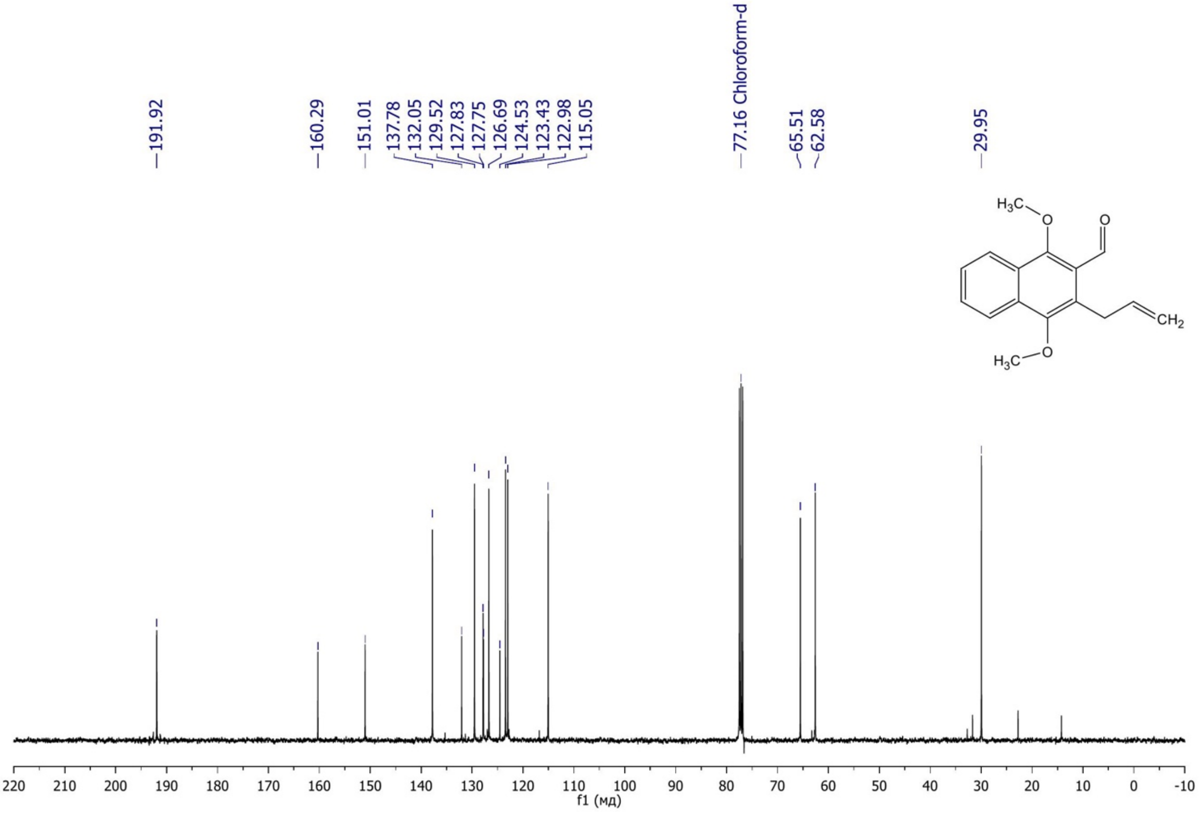

Supplementary Figure 57. ^{1}H, and ^{13}C NMR spectra of 3-allyl-1,4-dimethoxy-2-naphthaldehyde

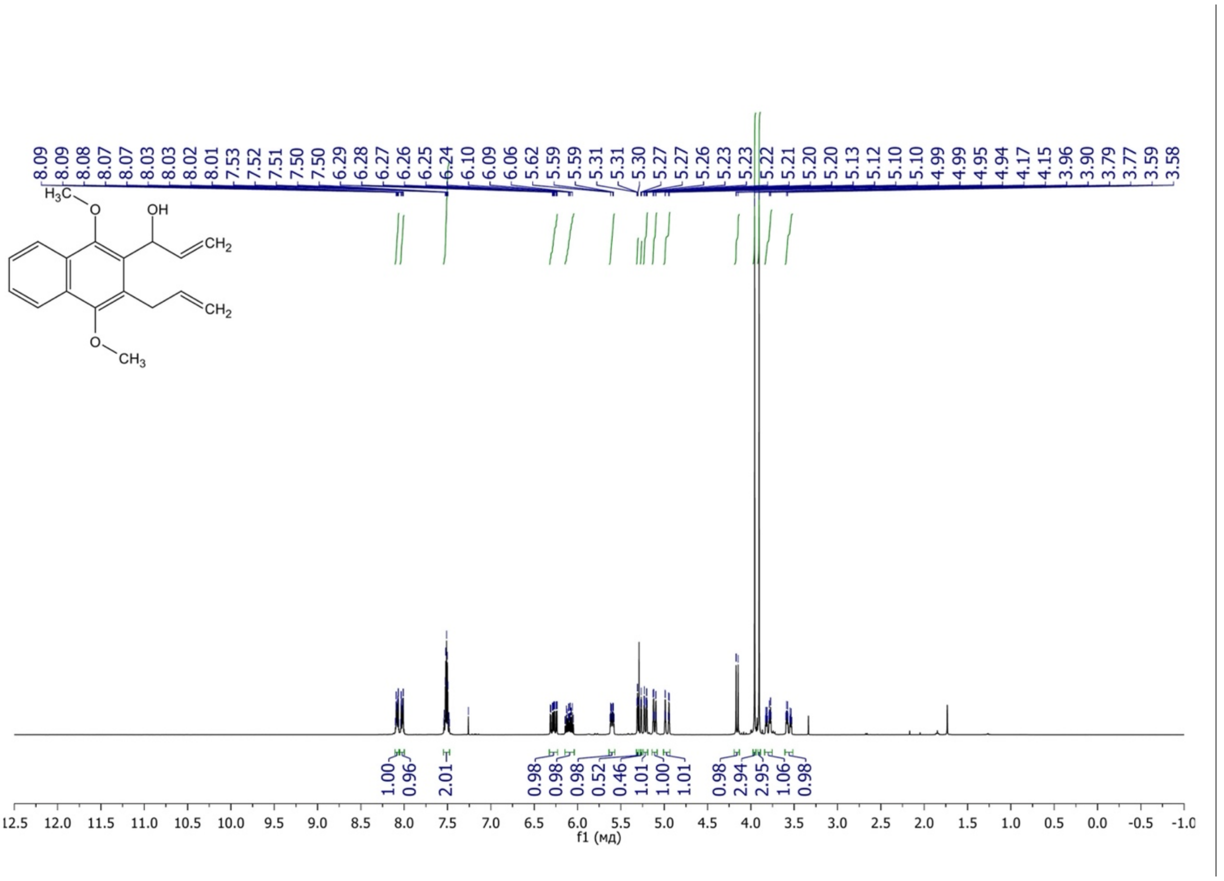

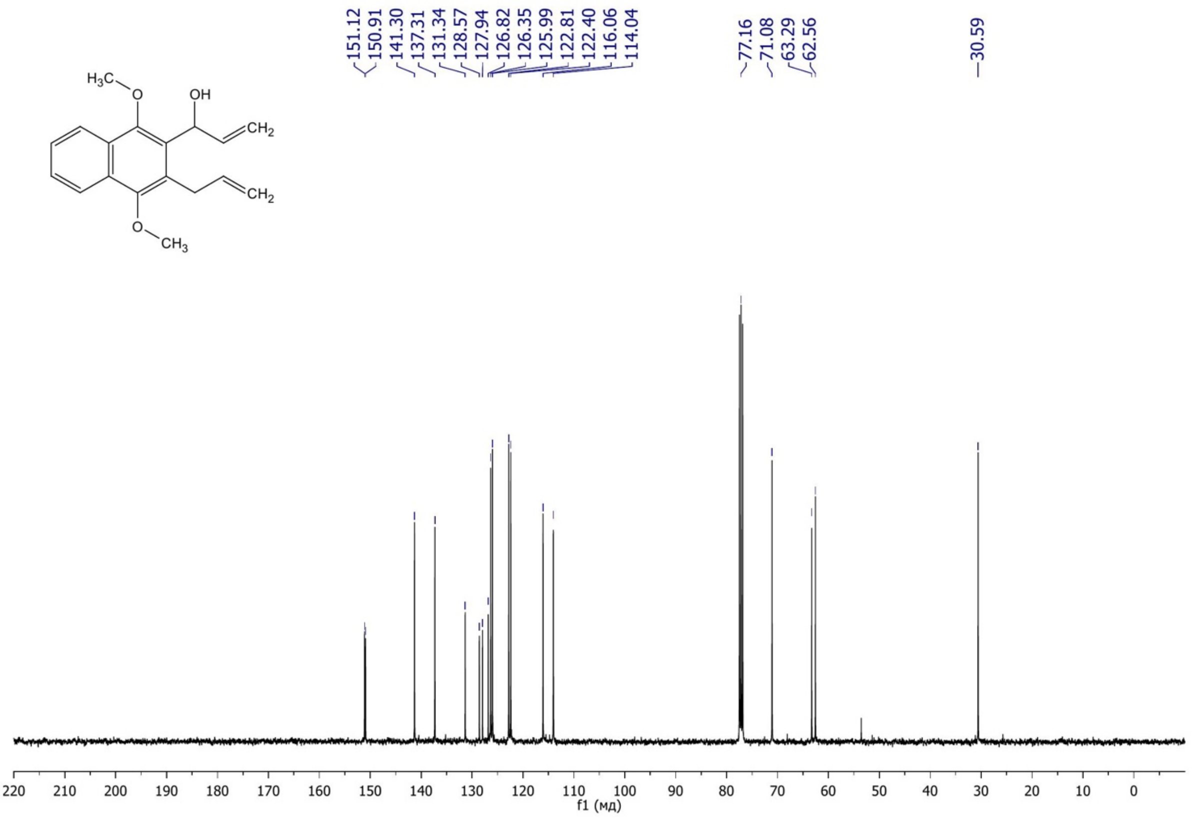

Supplementary Figure 58. ^{1}H and ^{13}C NMR spectra of 1-(3-allyl-1,4-dimethoxynaphthalen-2yl)prop-2-en-1-ol (**4**)

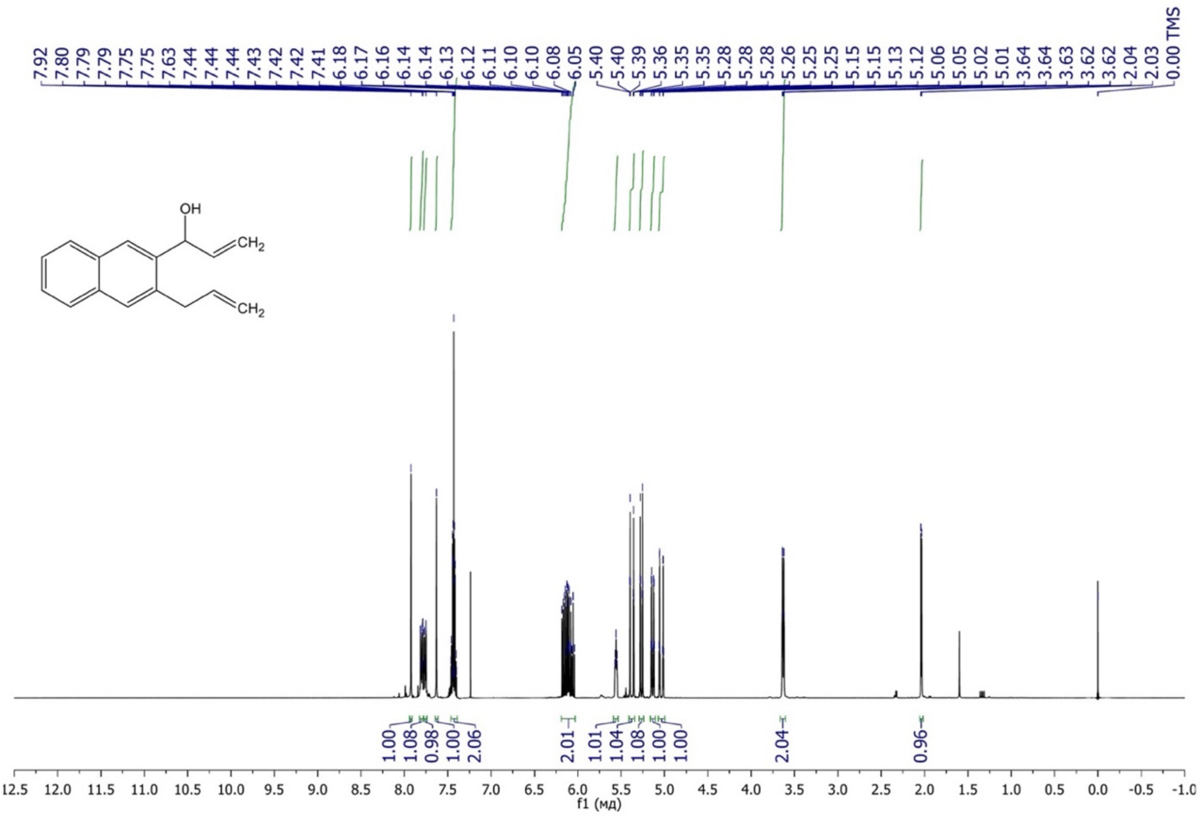

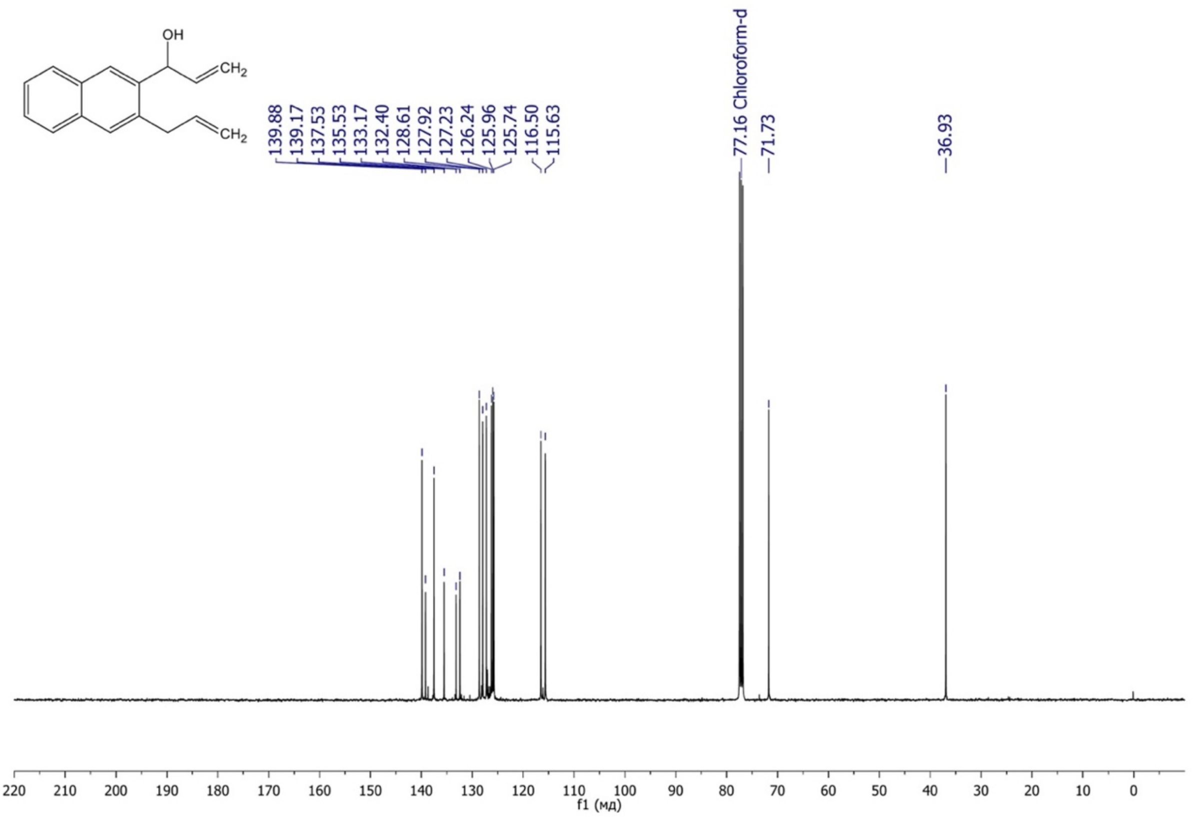

Supplementary Figure 59. ^{1}H and ^{13}C NMR spectra of 1-(3-allylnaphthalen-2-yl)prop-2-en-1-ol (**5**)

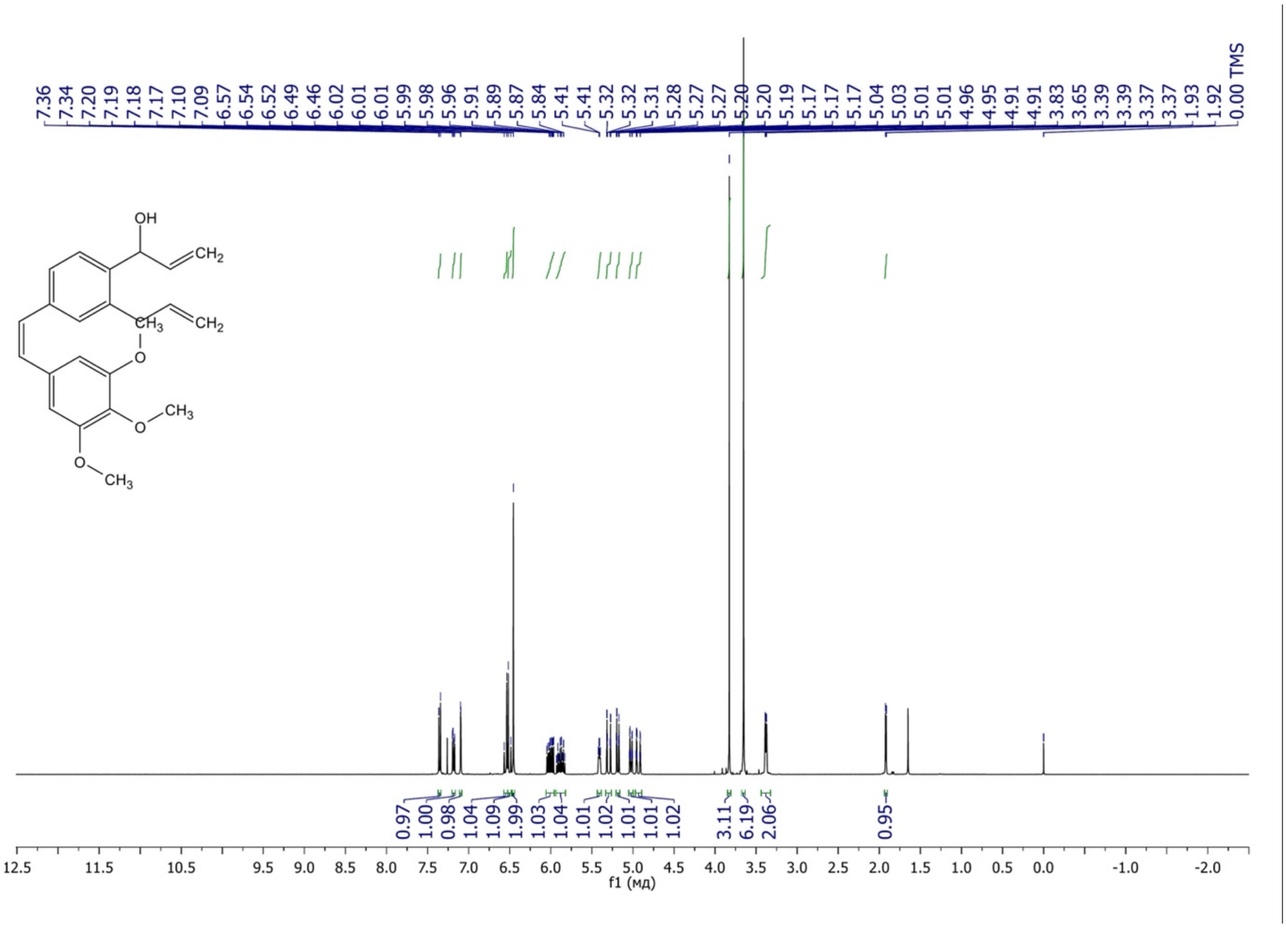

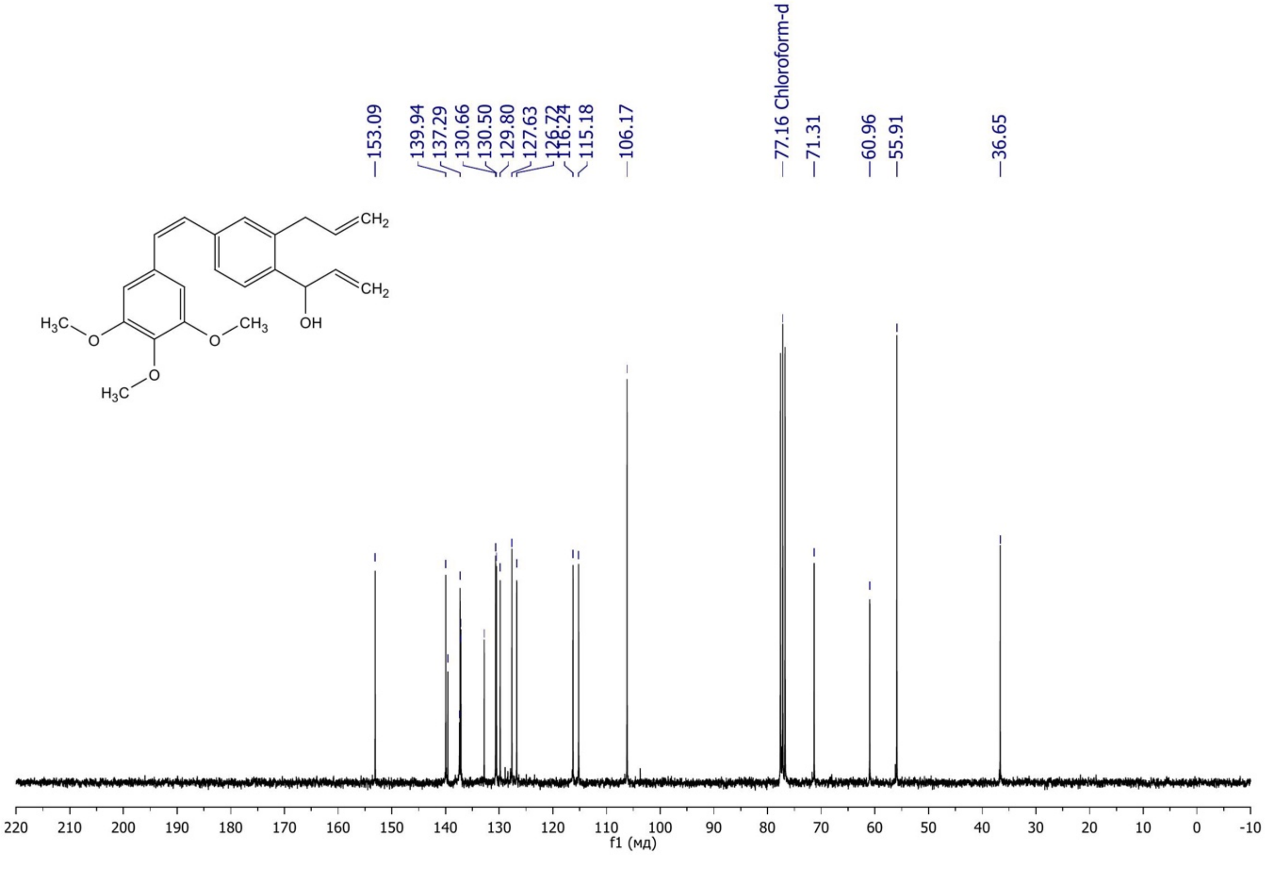

Supplementary Figure 60. ^{1}H and ^{13}C NMR spectra of
(*Z*)-1-(2-allyl-4-(3,4,5-trimethoxystyryl)phenyl)prop-2-en-1-ol (**19**)

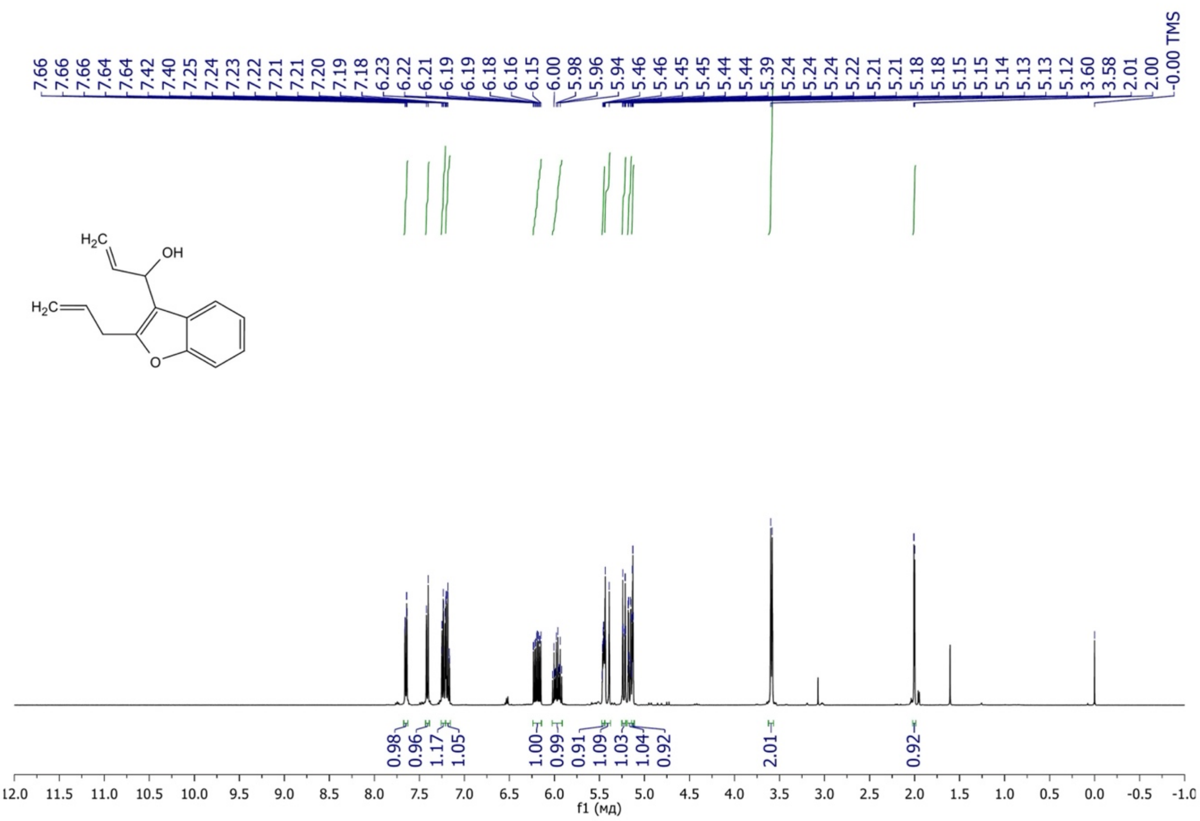

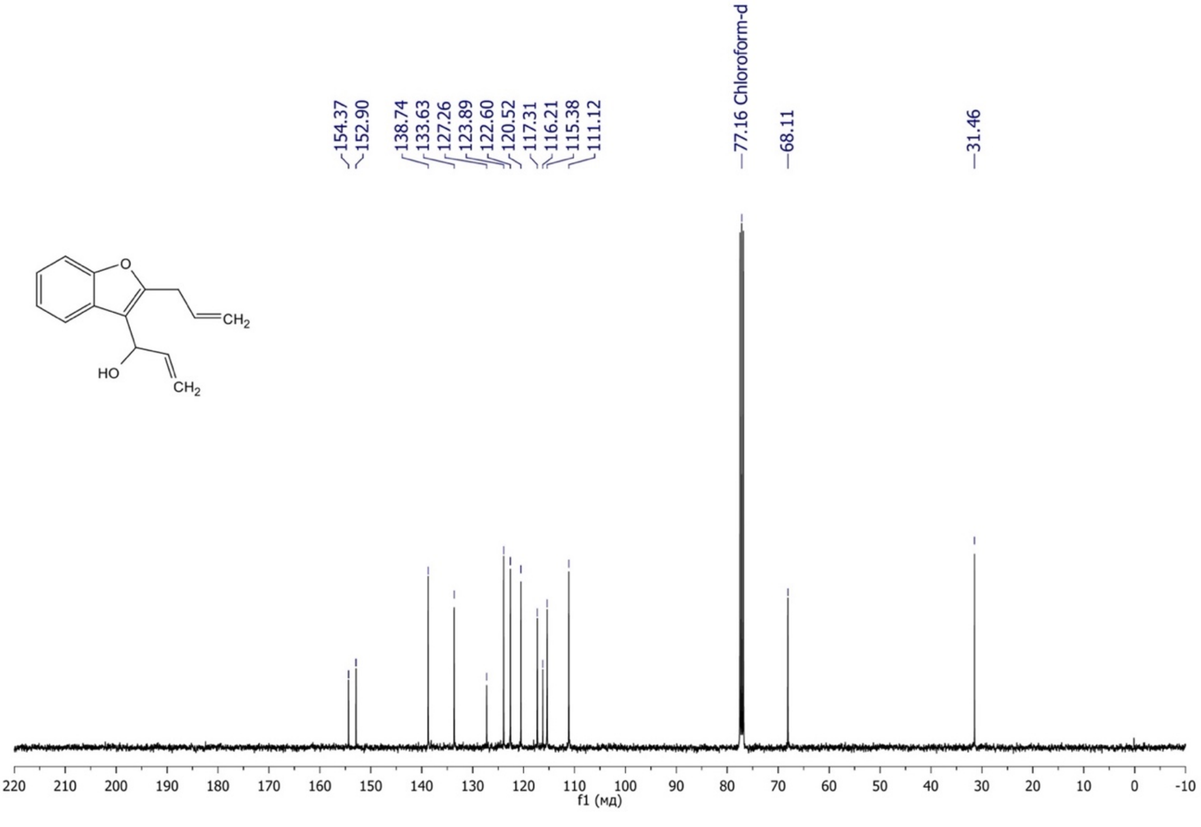

Supplementary Figure 61. ^{1}H and ^{13}C NMR spectra of 1-(2-allylbenzofuran-3-yl)prop-2-en-1-ol (**7**)

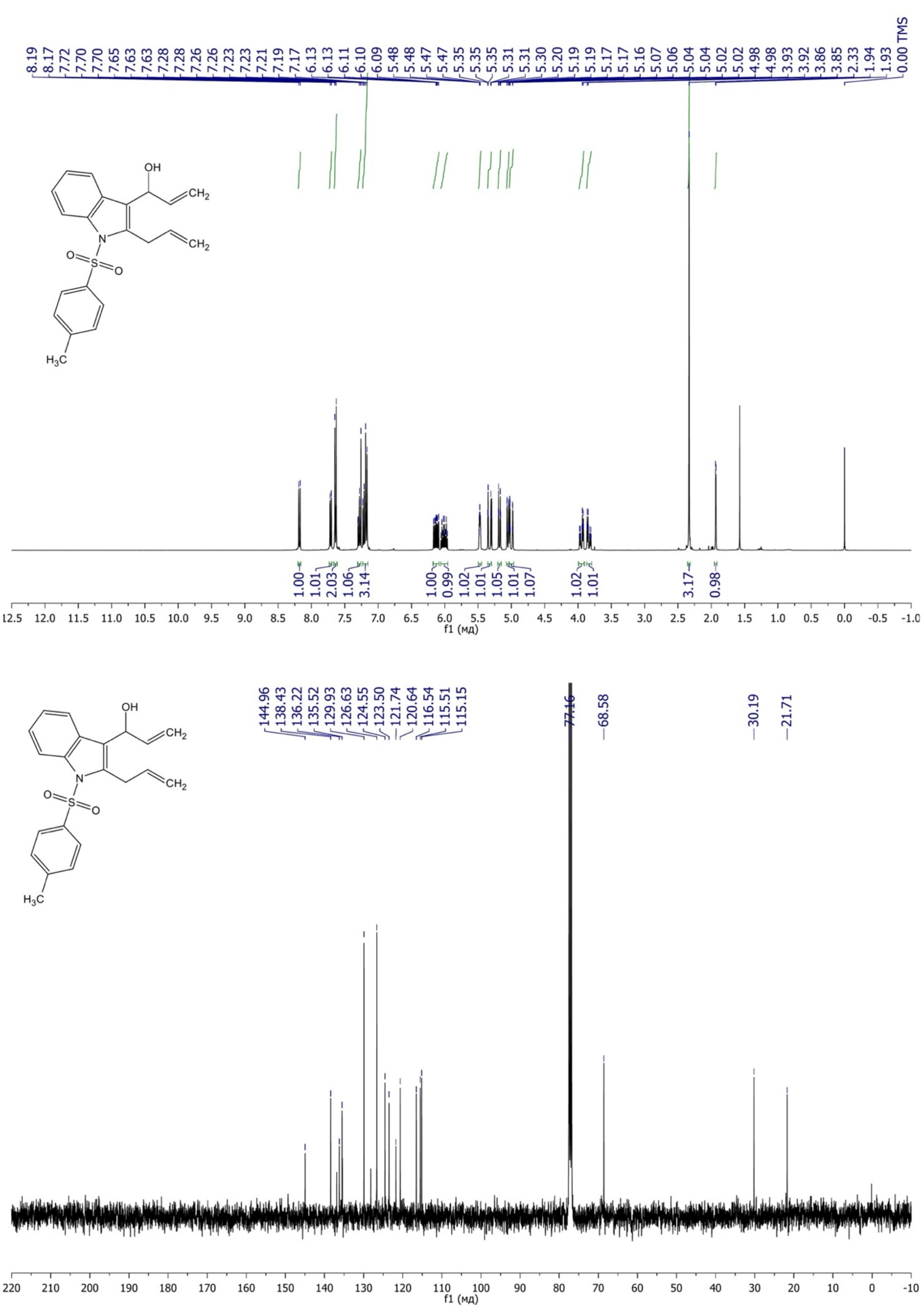

Supplementary Figure 62. ^{1}H and ^{13}C NMR spectra of 1-(2-allyl-1-tosyl-1H-indol-3-yl)prop-2-en-1-ol (**6**)

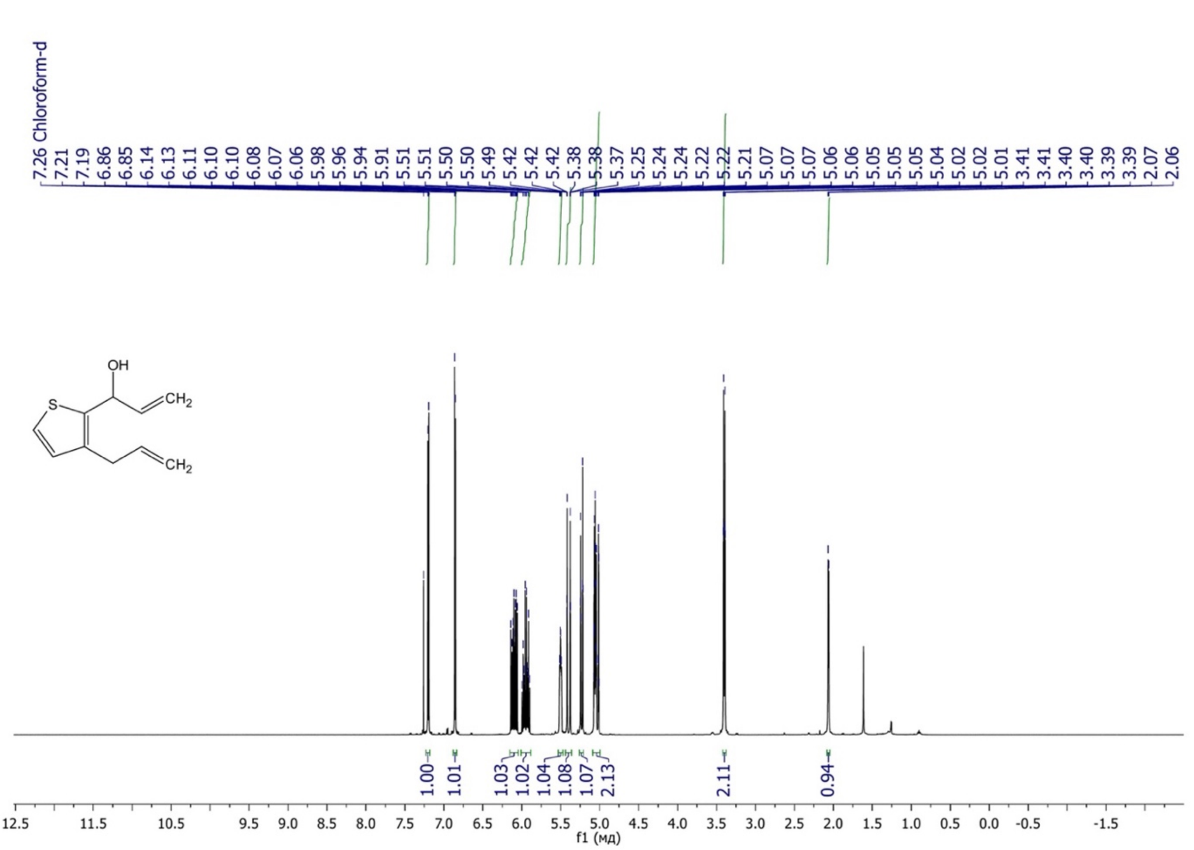

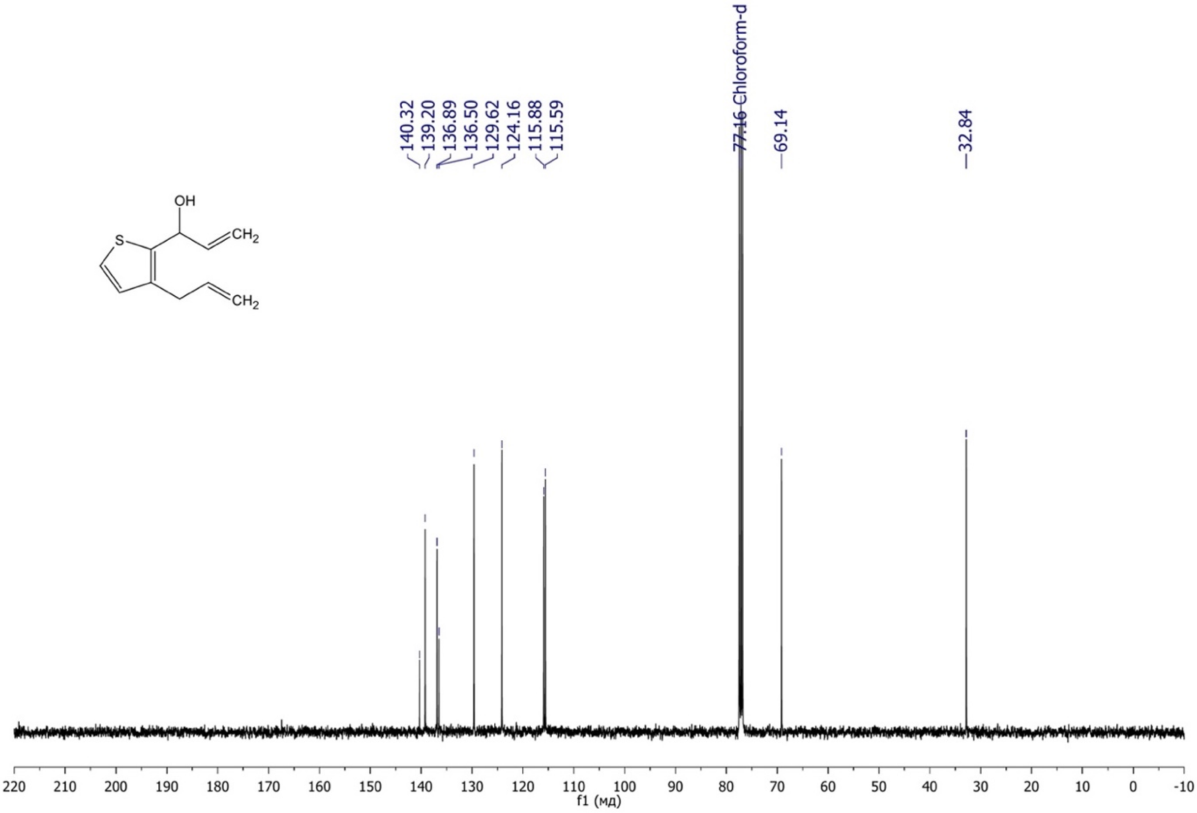

Supplementary Figure 63. ^{1}H and ^{13}C NMR spectra of 1-(3-allylthiophen-2-yl)prop-2-en-1-ol (**8**)

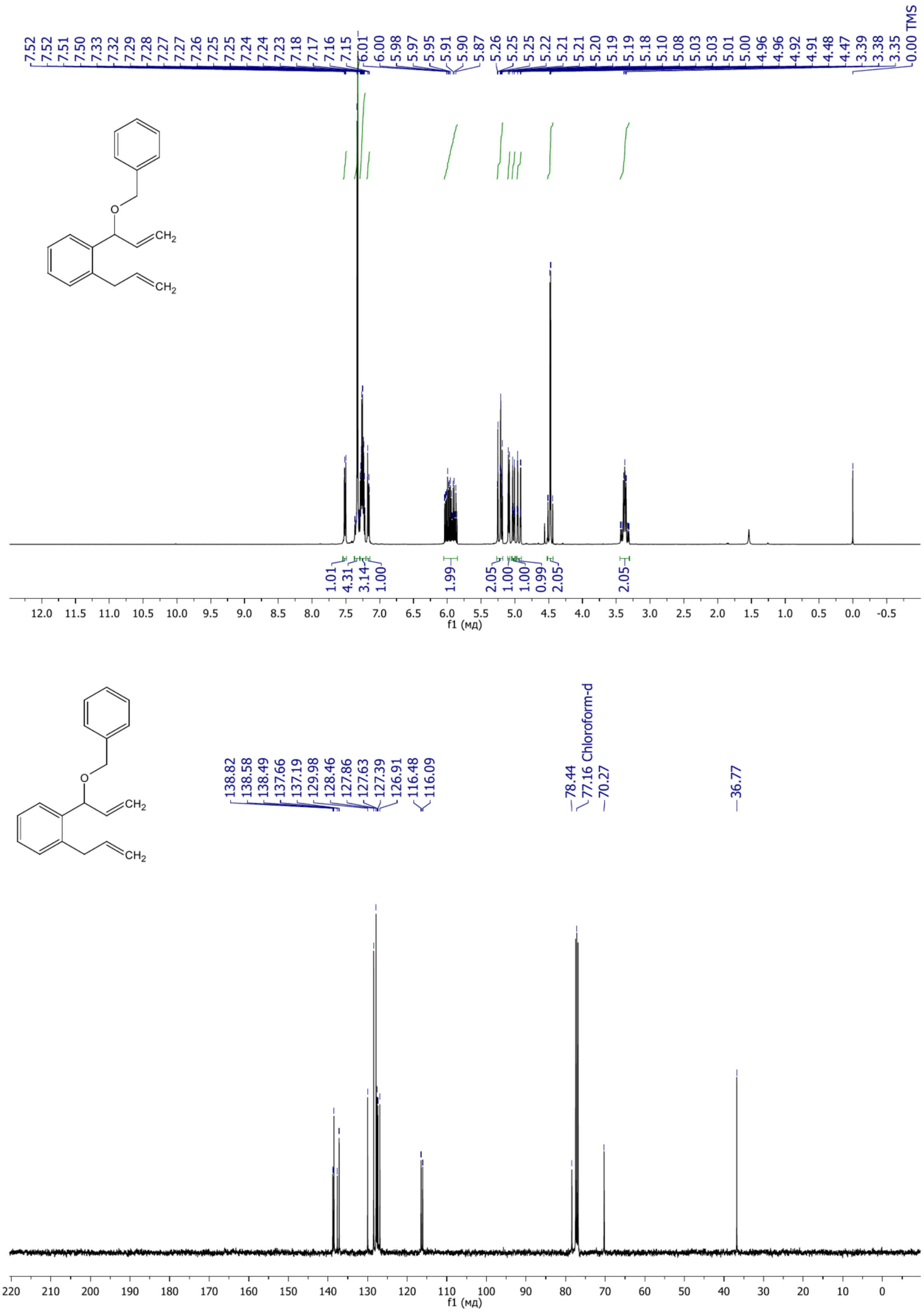

Supplementary Figure 64. ^{1}H and ^{13}C NMR spectra of 1-allyl-2-(1-(benzyloxy)allyl)benzene (**10**)

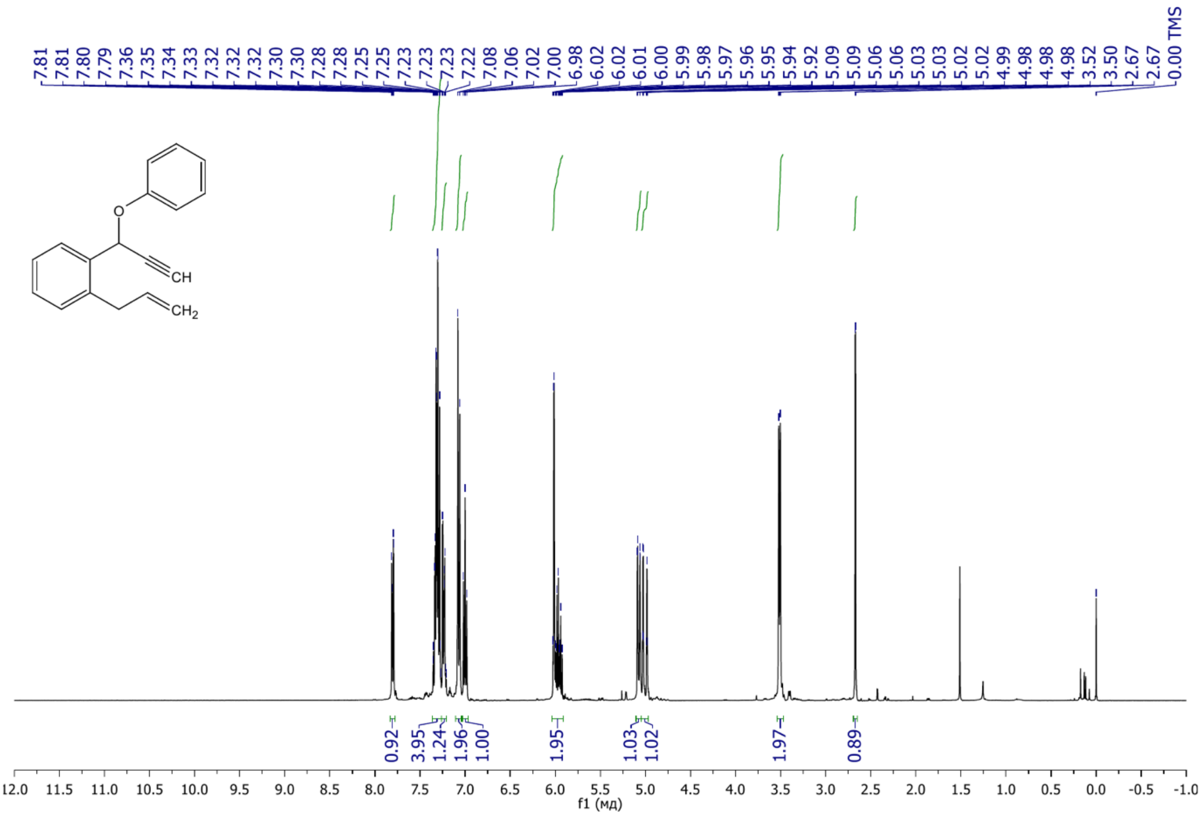

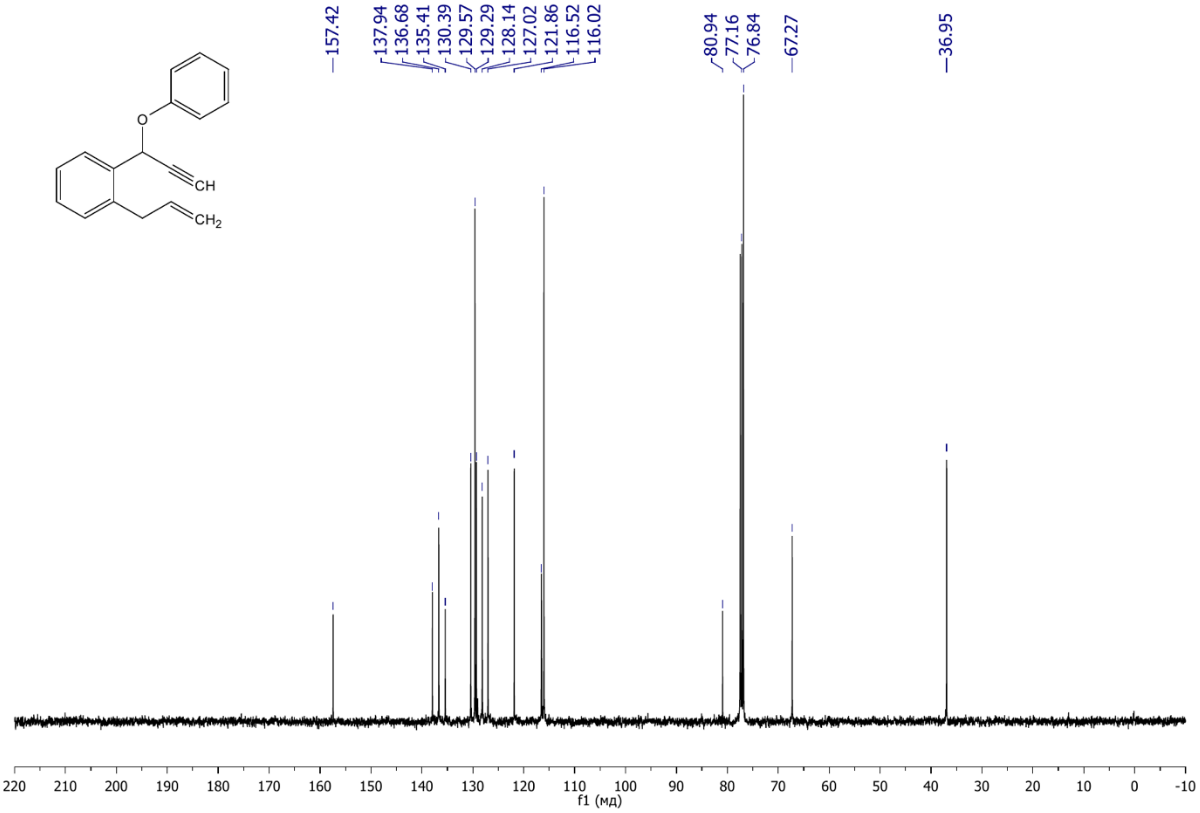

Supplementary Figure 65. ^{1}H and ^{13}C NMR spectra of 1-allyl-2-(1-phenoxyprop-2-yn-1yl)benzene

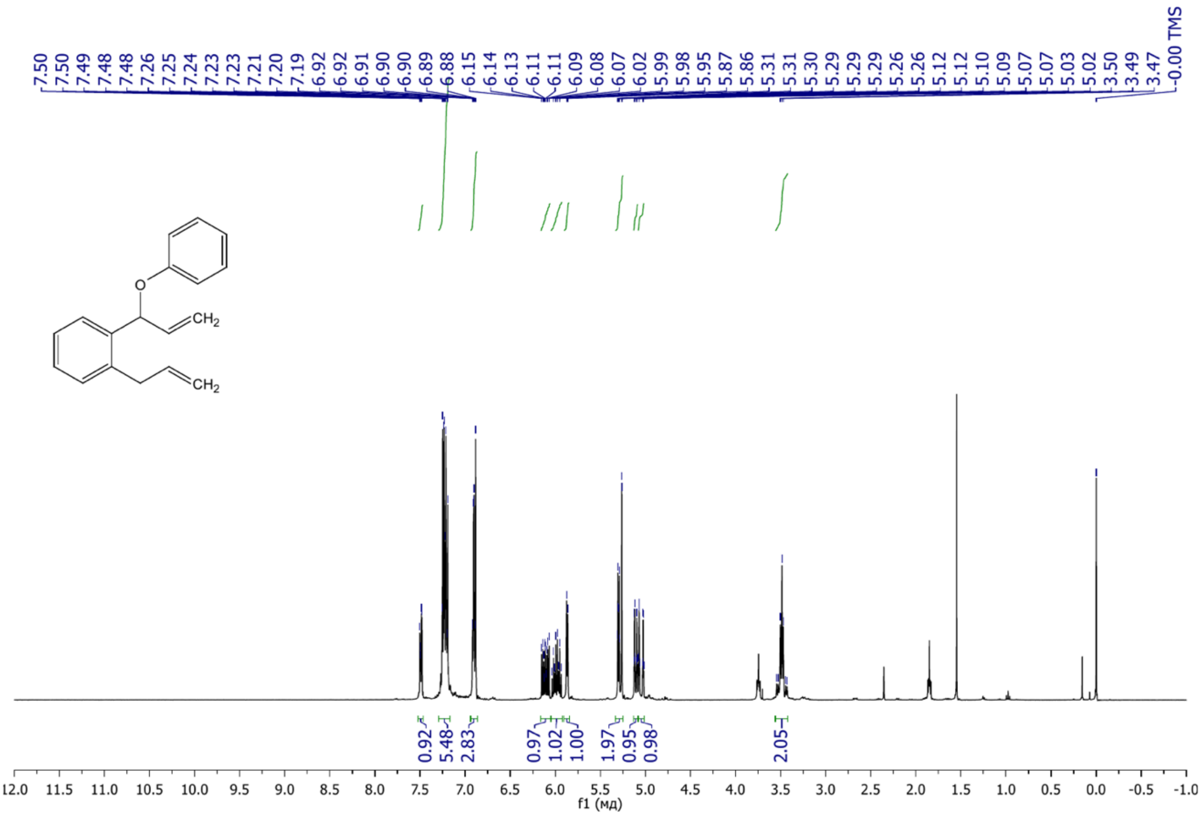

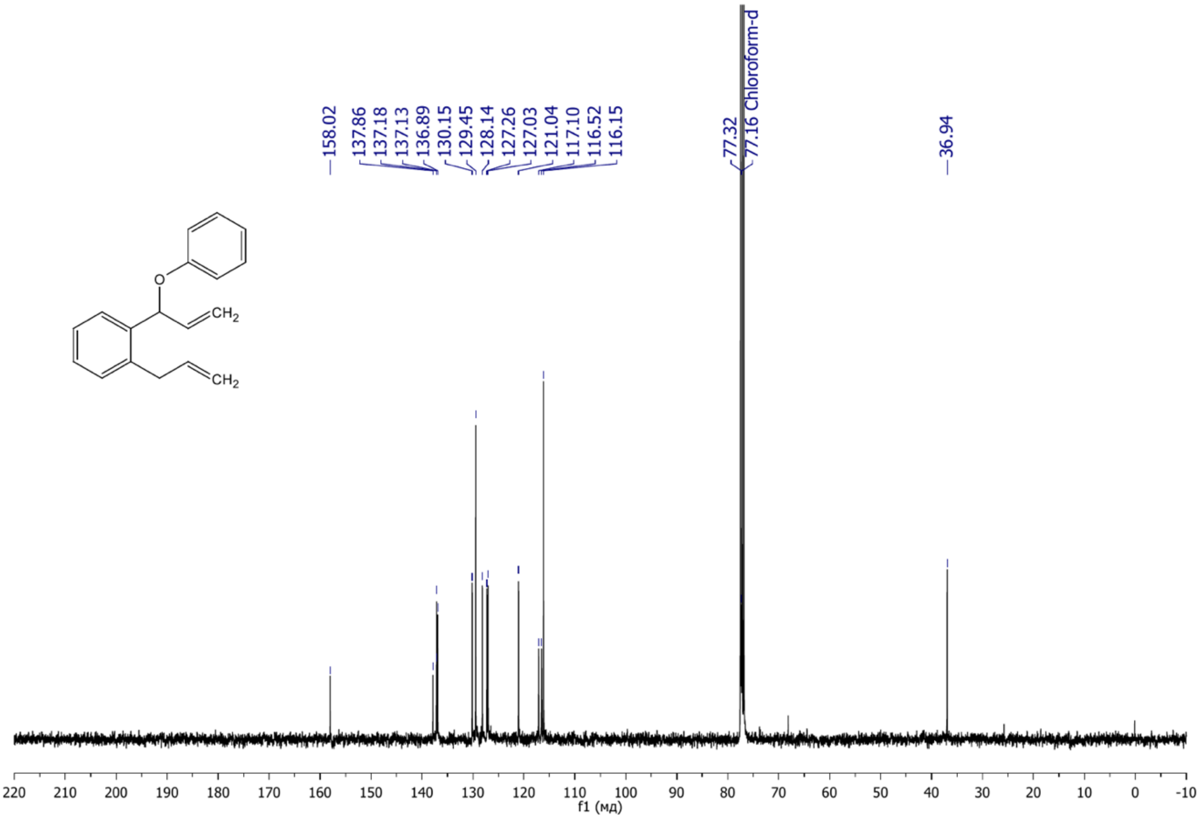

Supplementary Figure 66. ^{1}H and ^{13}C NMR spectra of 1-allyl-2-(1-phenoxyallyl)benzene (**11**)

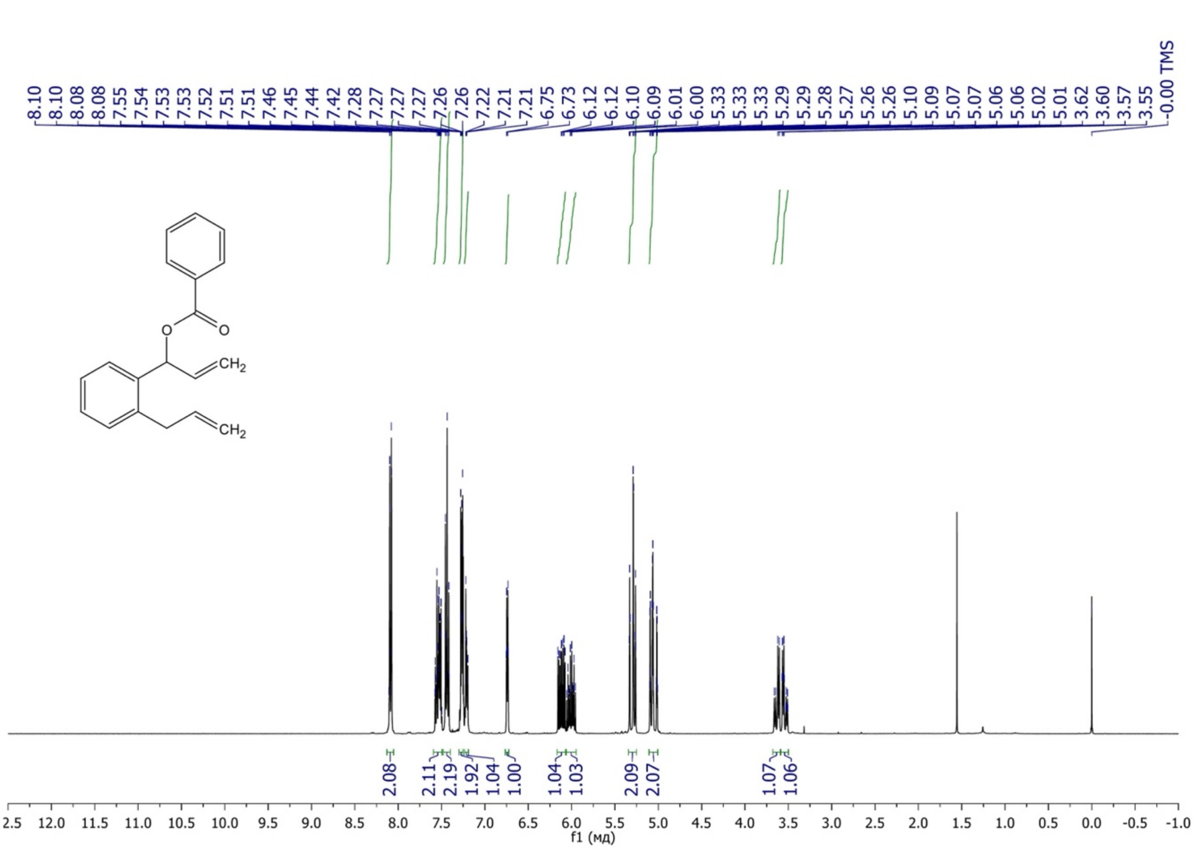

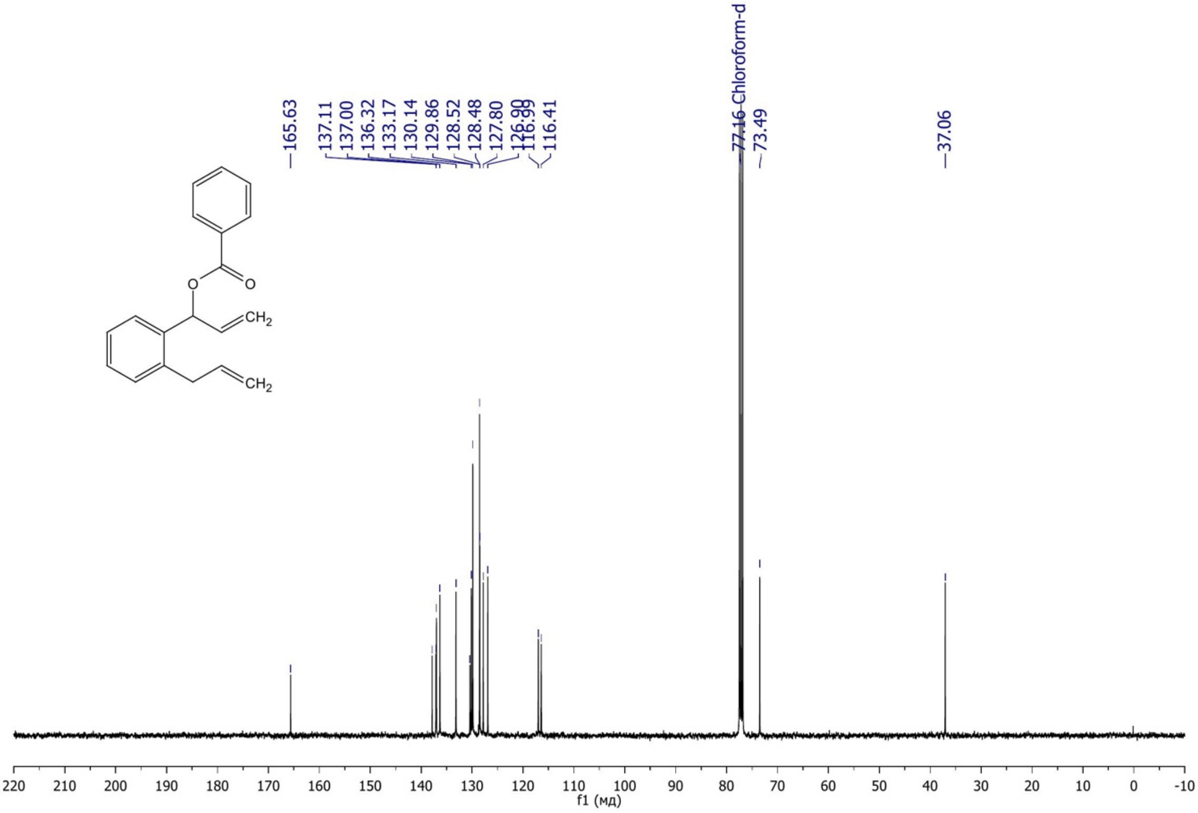

Supplementary Figure 67. ^{1}H and ^{13}C NMR spectra of 1-(2-allylphenyl)allyl benzoate (**12**)

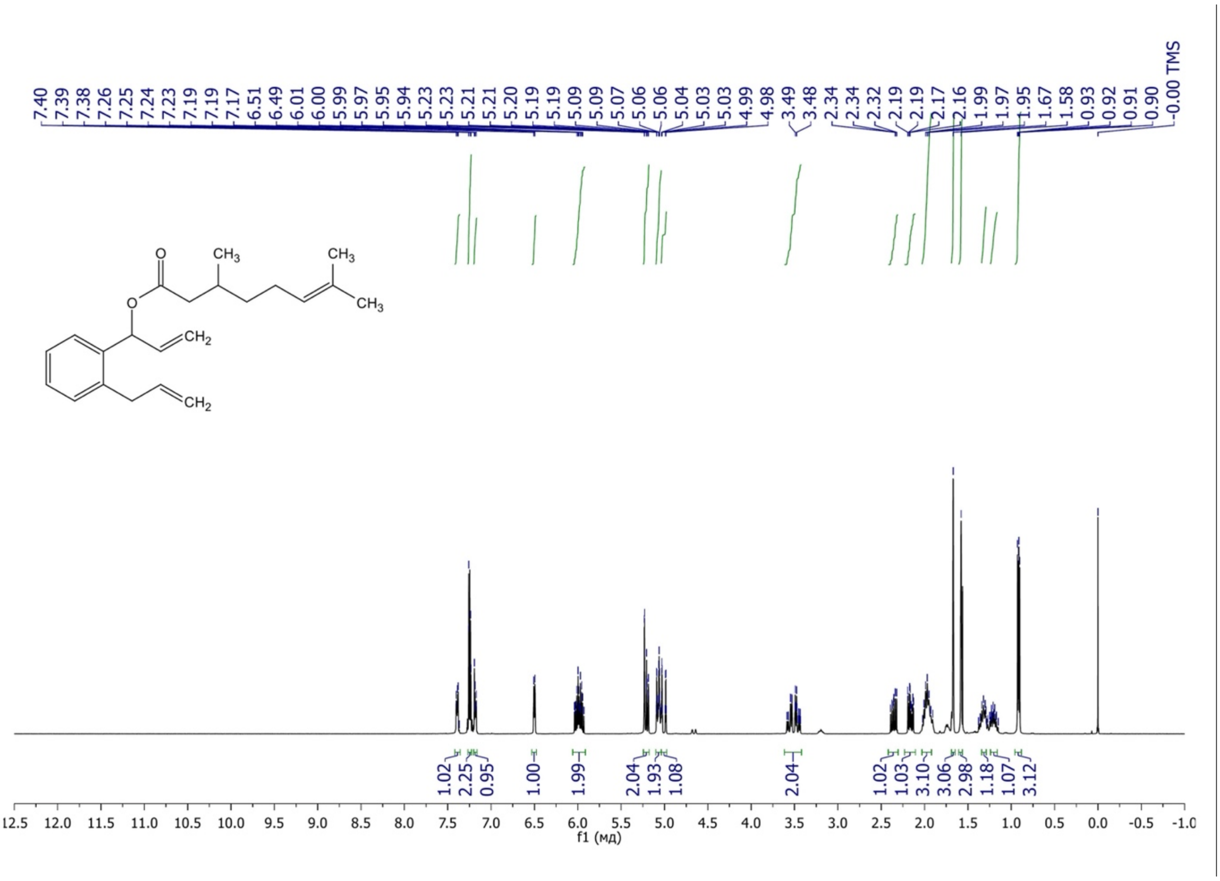

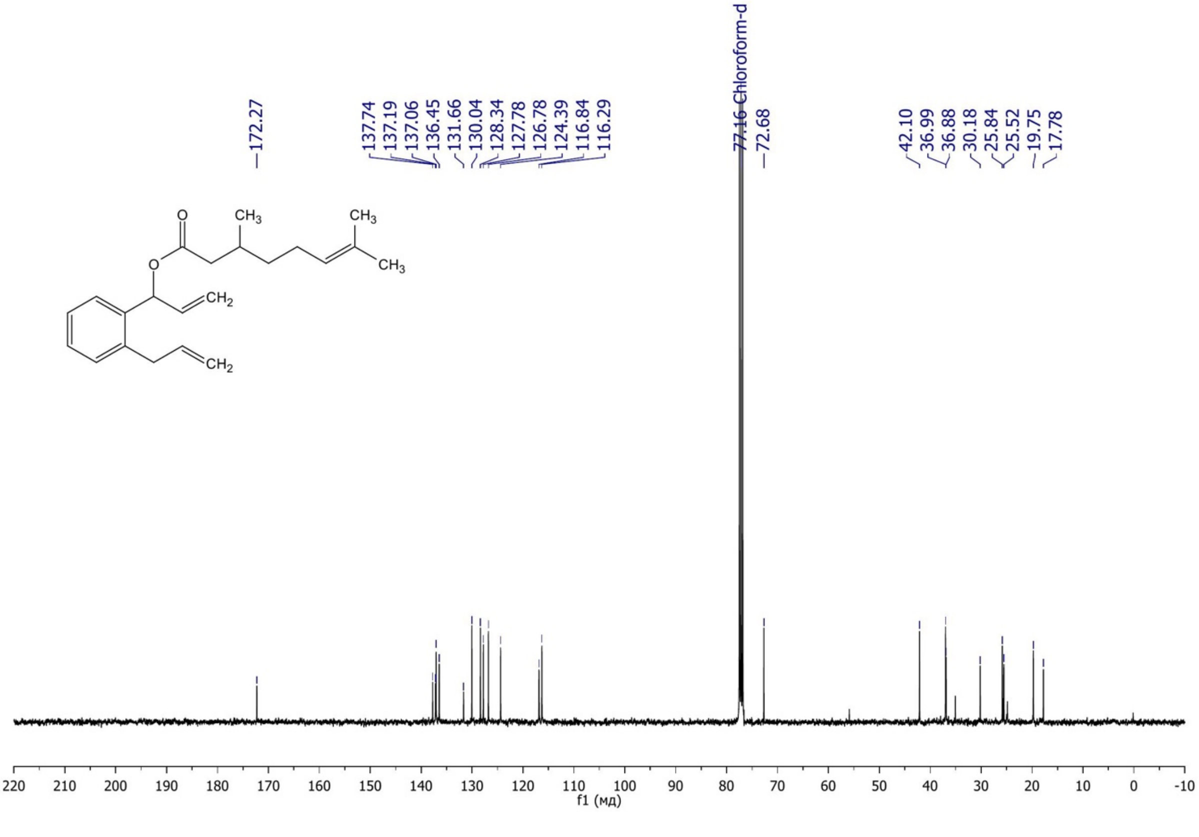

Supplementary Figure 68. ^{1}H and ^{13}C NMR spectra of 1-(2-allylphenyl)allyl 3,7-dimethyloct-6-enoate (**14**)

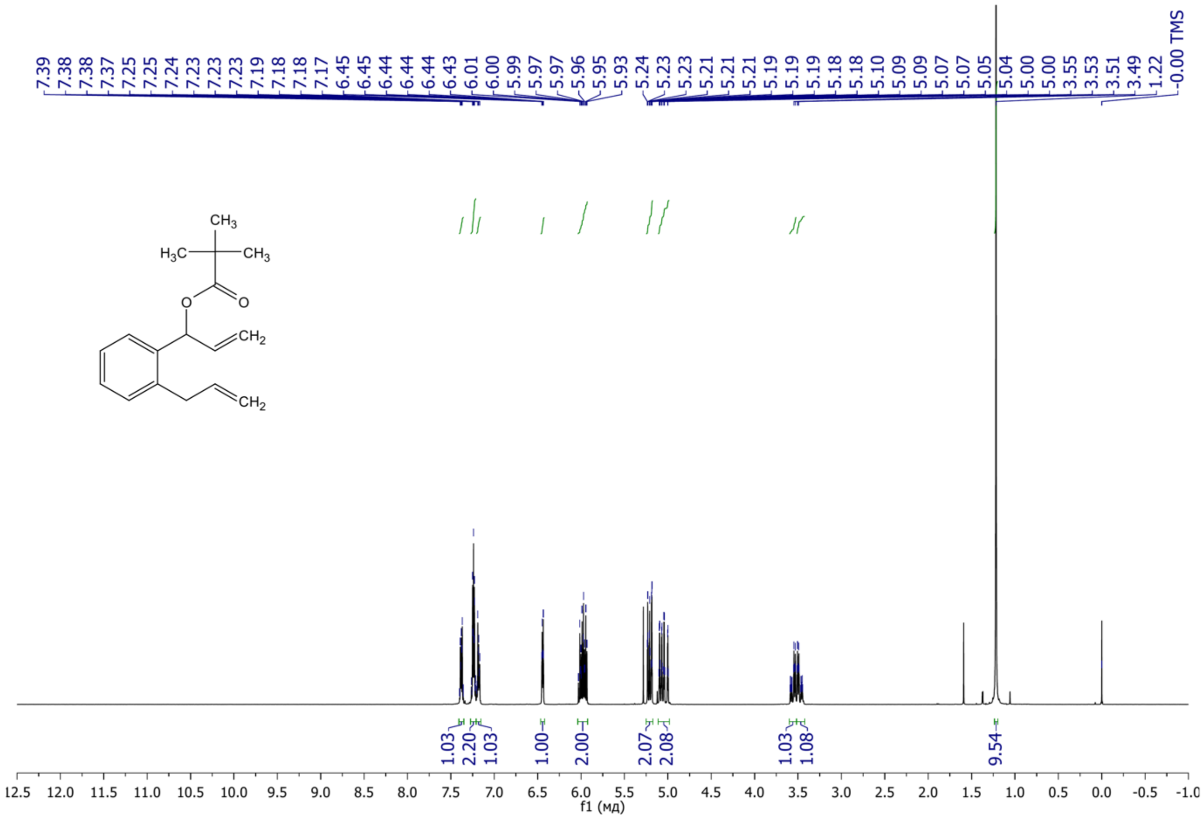

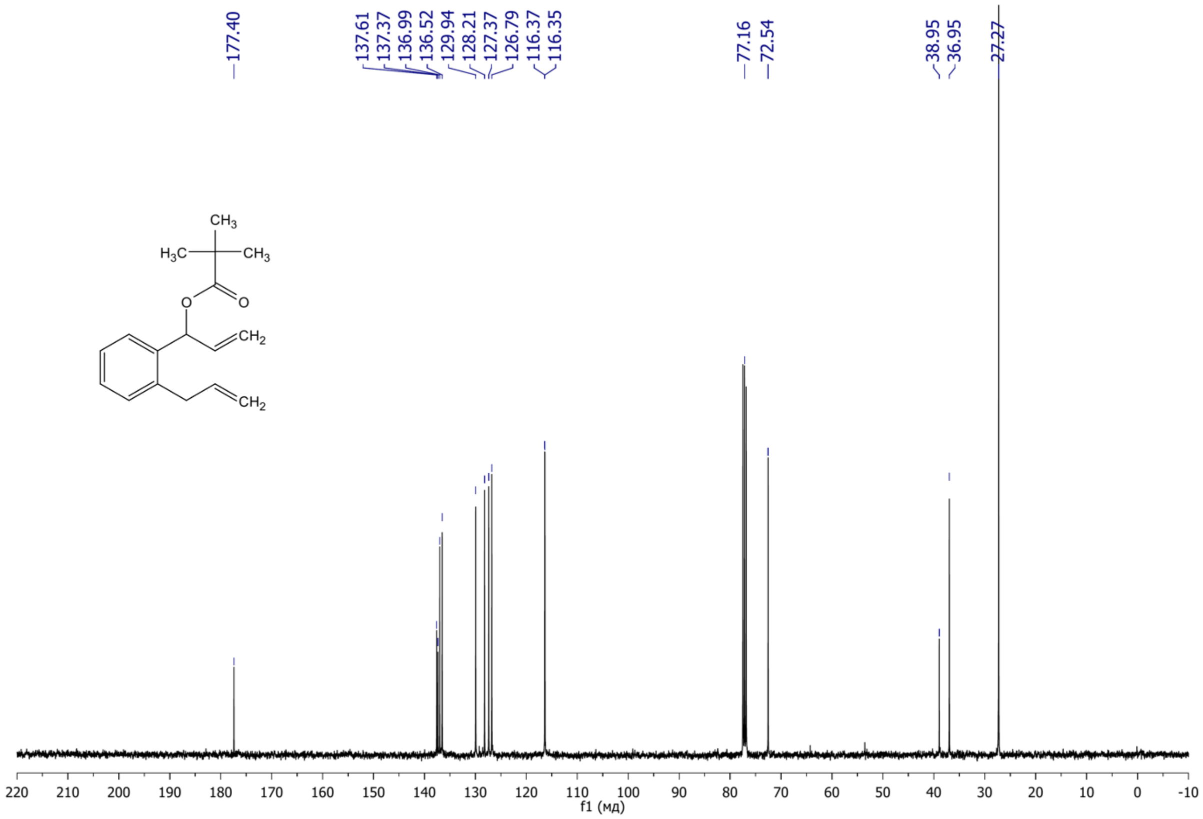

Supplementary Figure 69. ^{1}H and ^{13}C NMR spectra of 1 1-(2-allylphenyl)allyl pivalate (**15**)

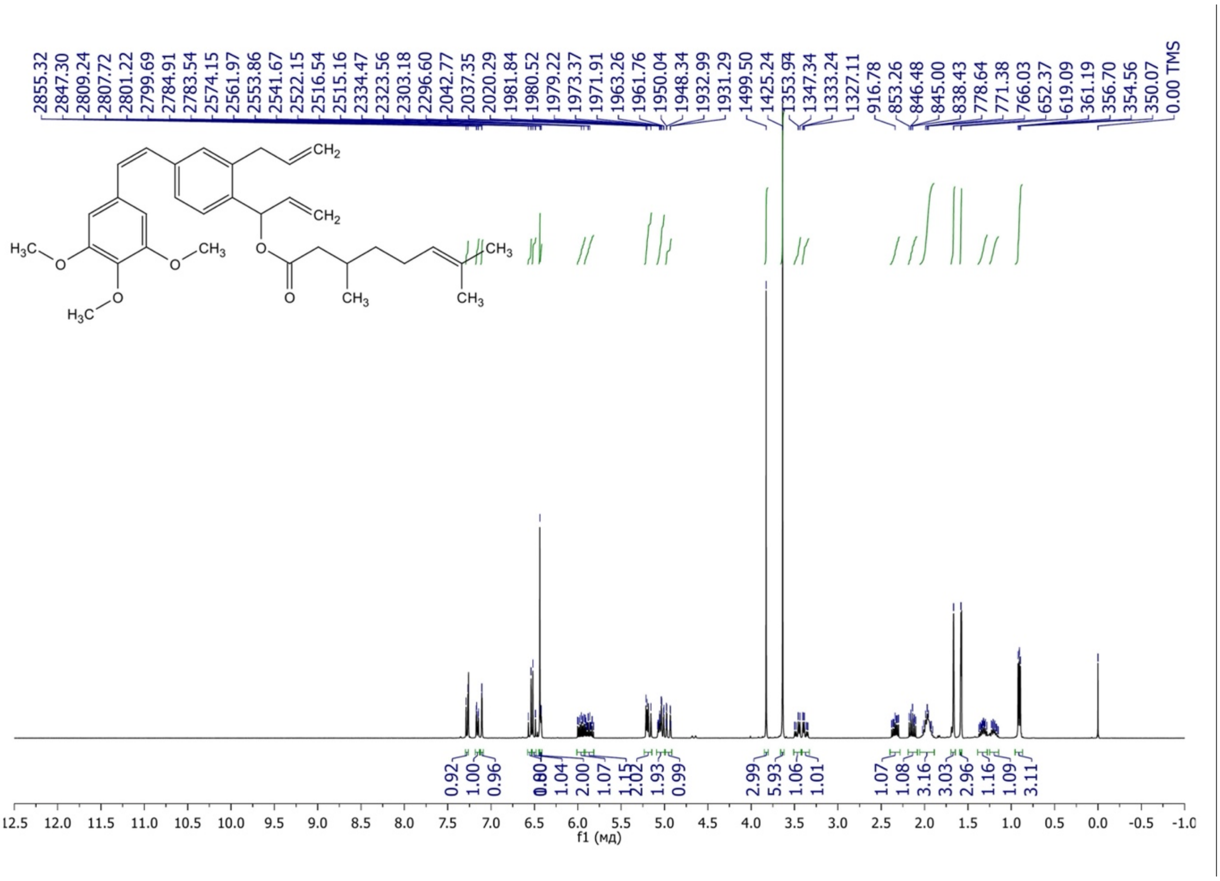

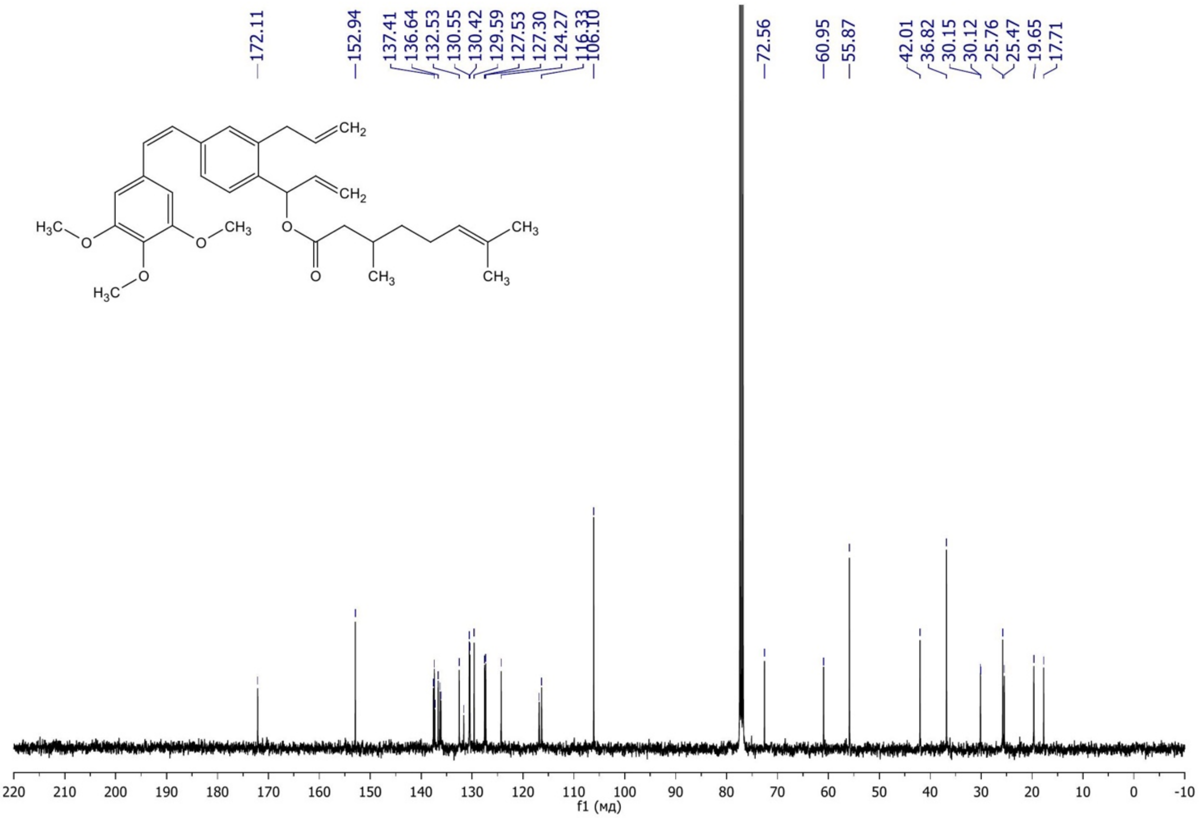

Supplementary Figure 70. ^{1}H and ^{13}C NMR spectra of (*Z*)-1-(2-allyl-4-(3,4,5-trimethoxystyryl)phenyl)allyl 3,7-dimethyloct-6-enoate (**16**)

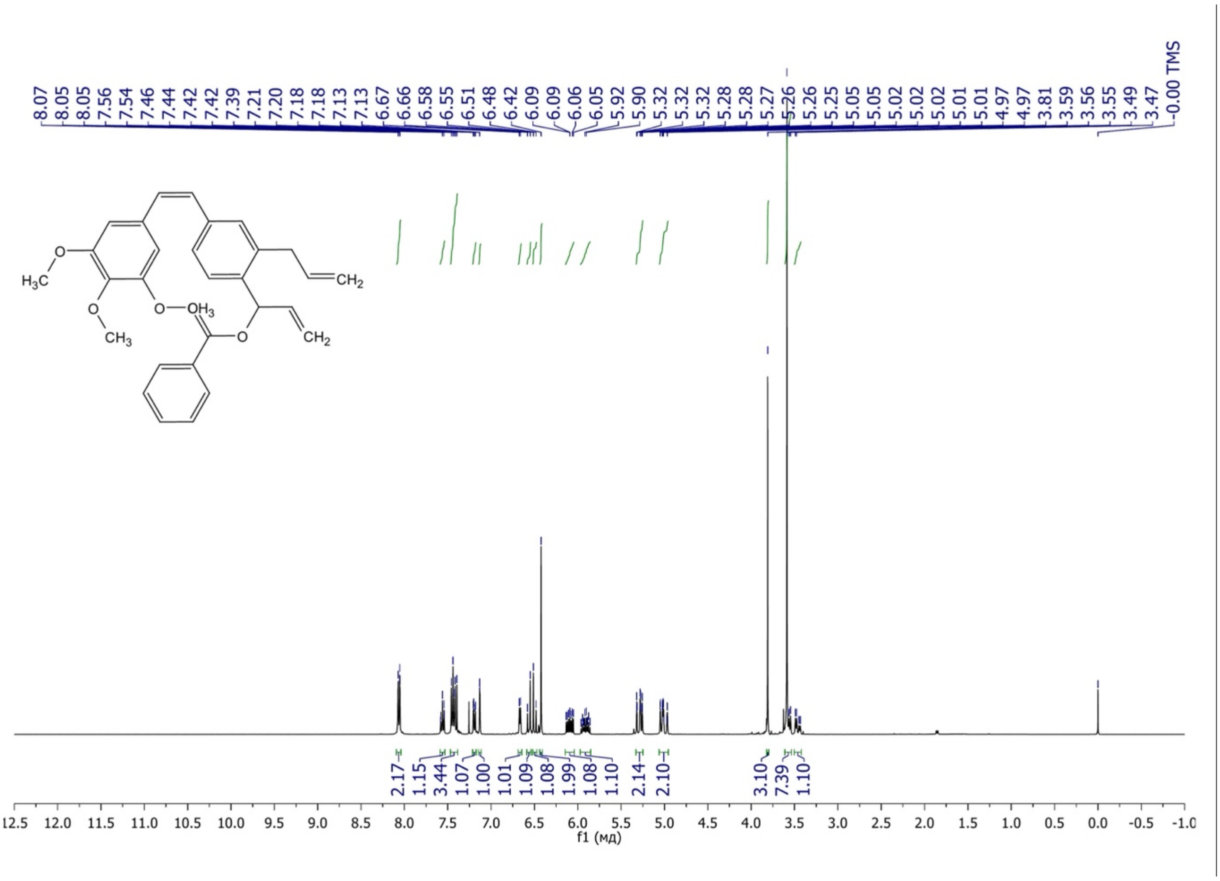

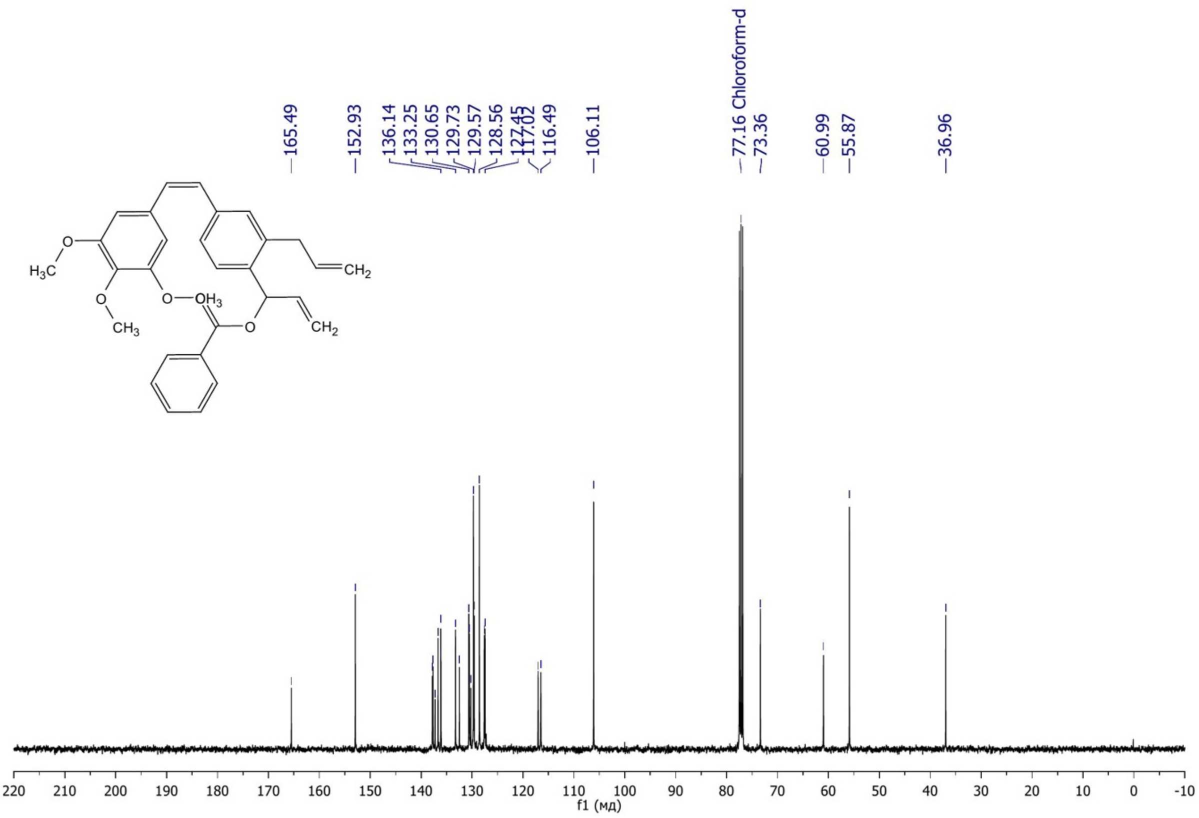

Supplementary Figure 71. ^{1}H and ^{13}C NMR spectra of
(*Z*)-1-(2-allyl-4-(3,4,5-trimethoxystyryl)phenyl)allyl benzoate (**18**)

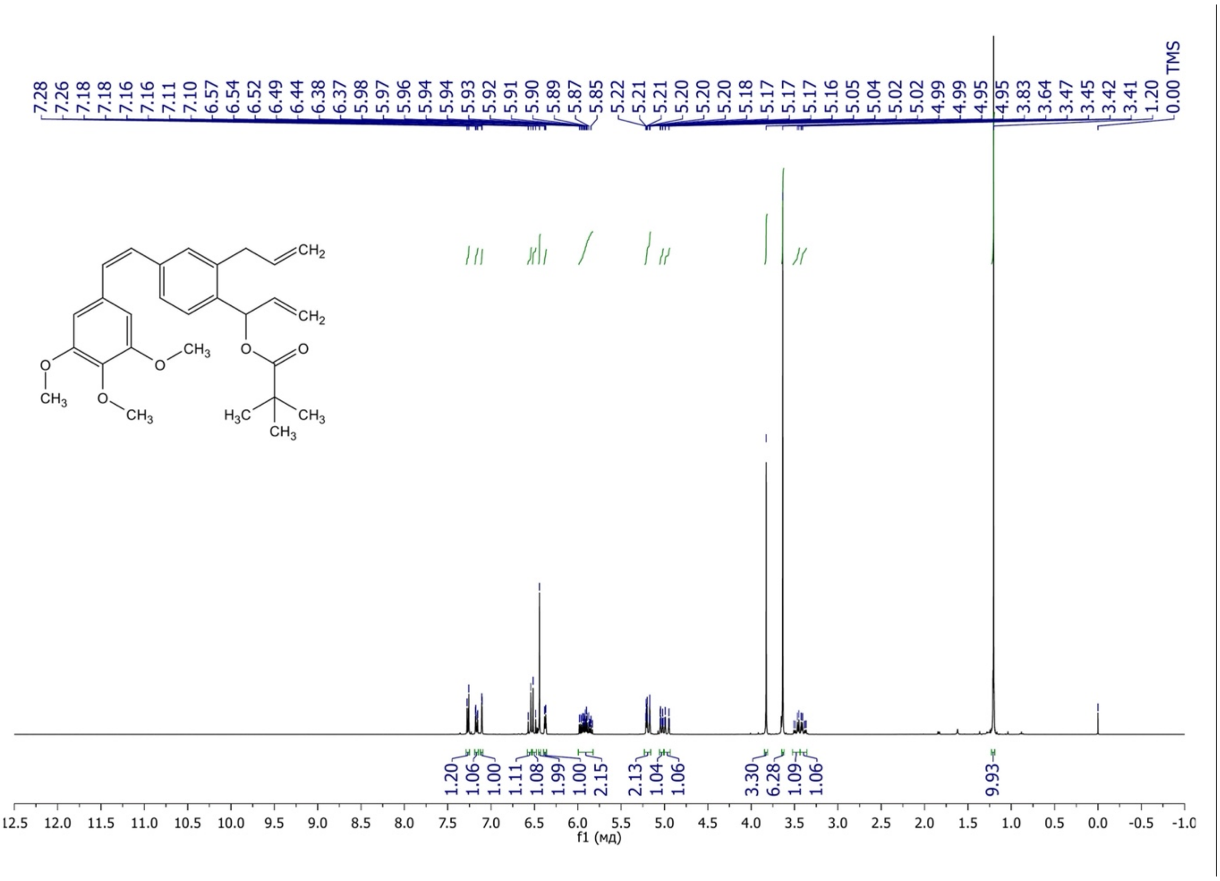

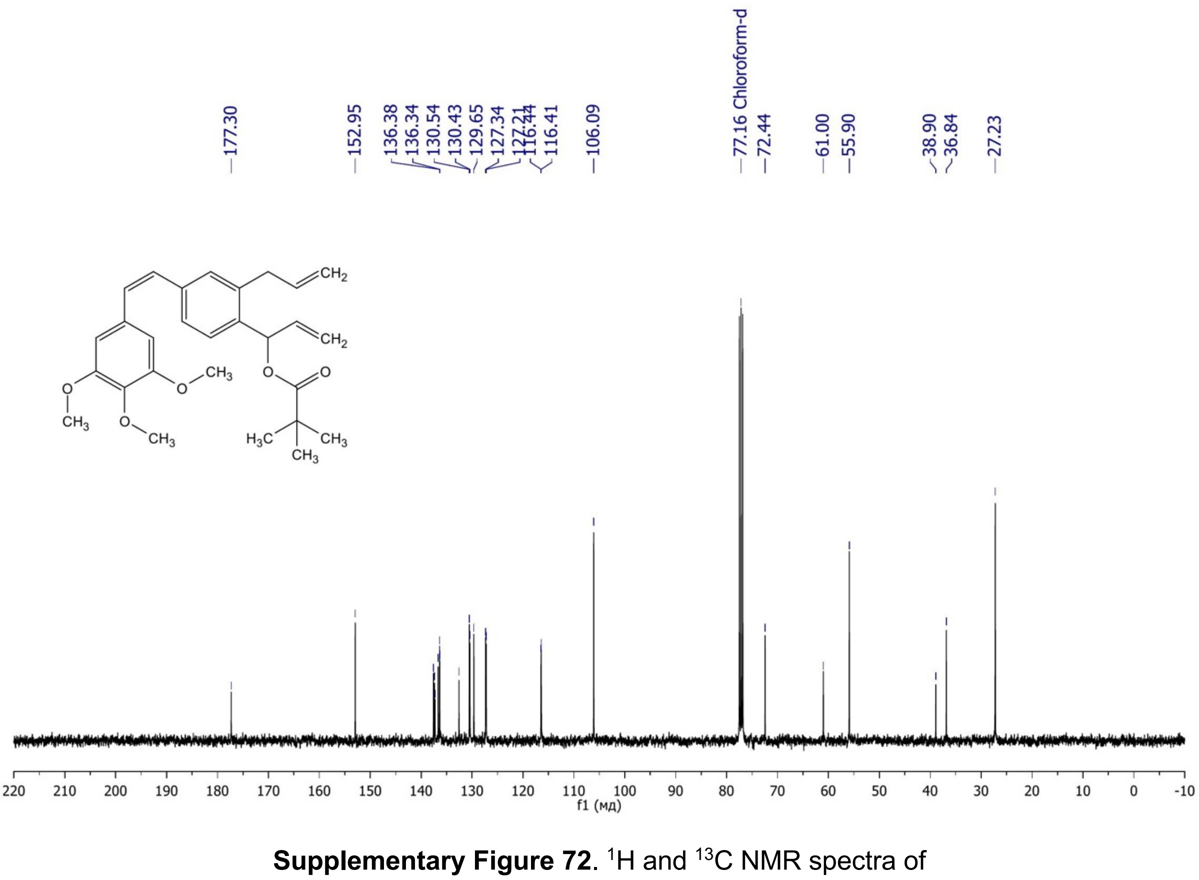

Supplementary Figure 72. ^{1}H and ^{13}C NMR spectra of (*Z*)-1-(2-allyl-4-(3,4,5-trimethoxystyryl)phenyl)allyl pivalate (**17**)

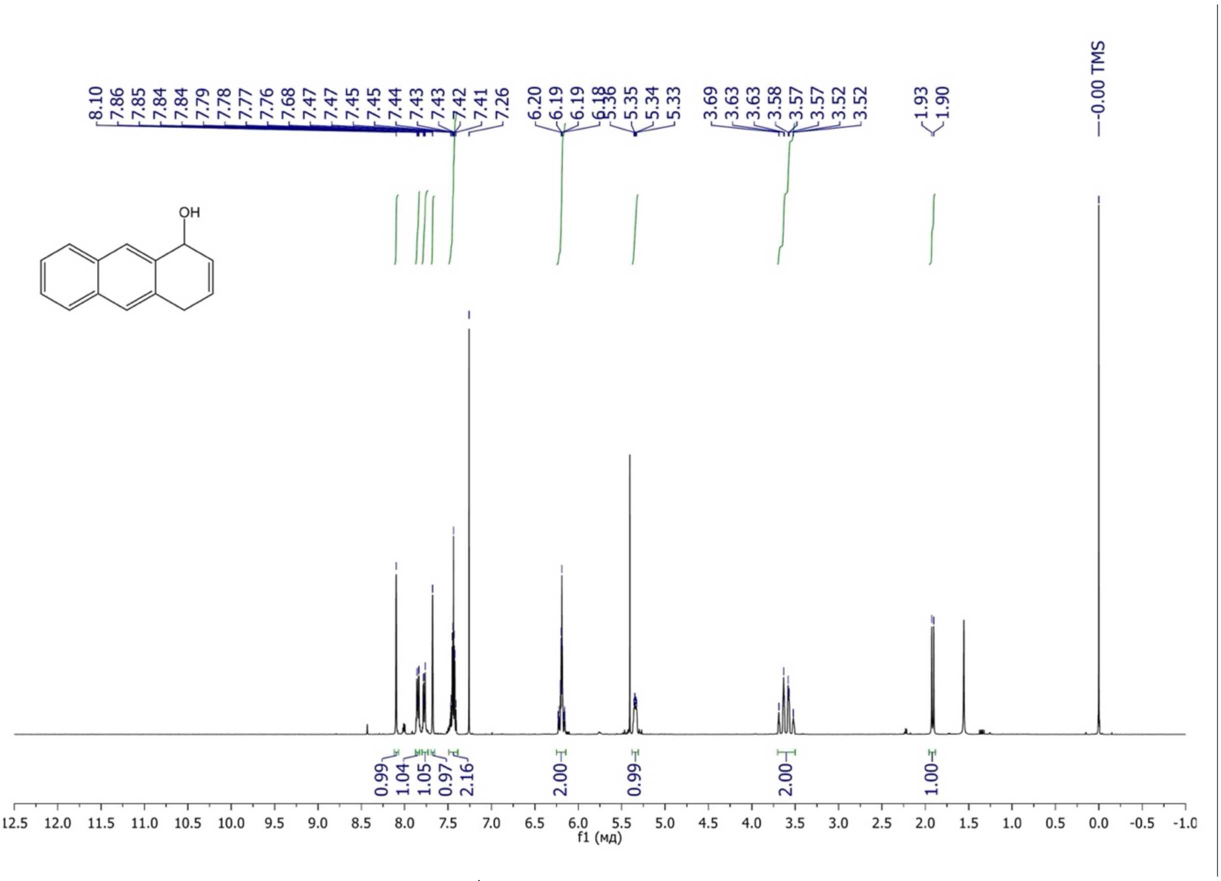

Supplementary Figure 73. ^{1}H NMR spectra of 1,4-dihydroanthracen-1-ol (**5a**)

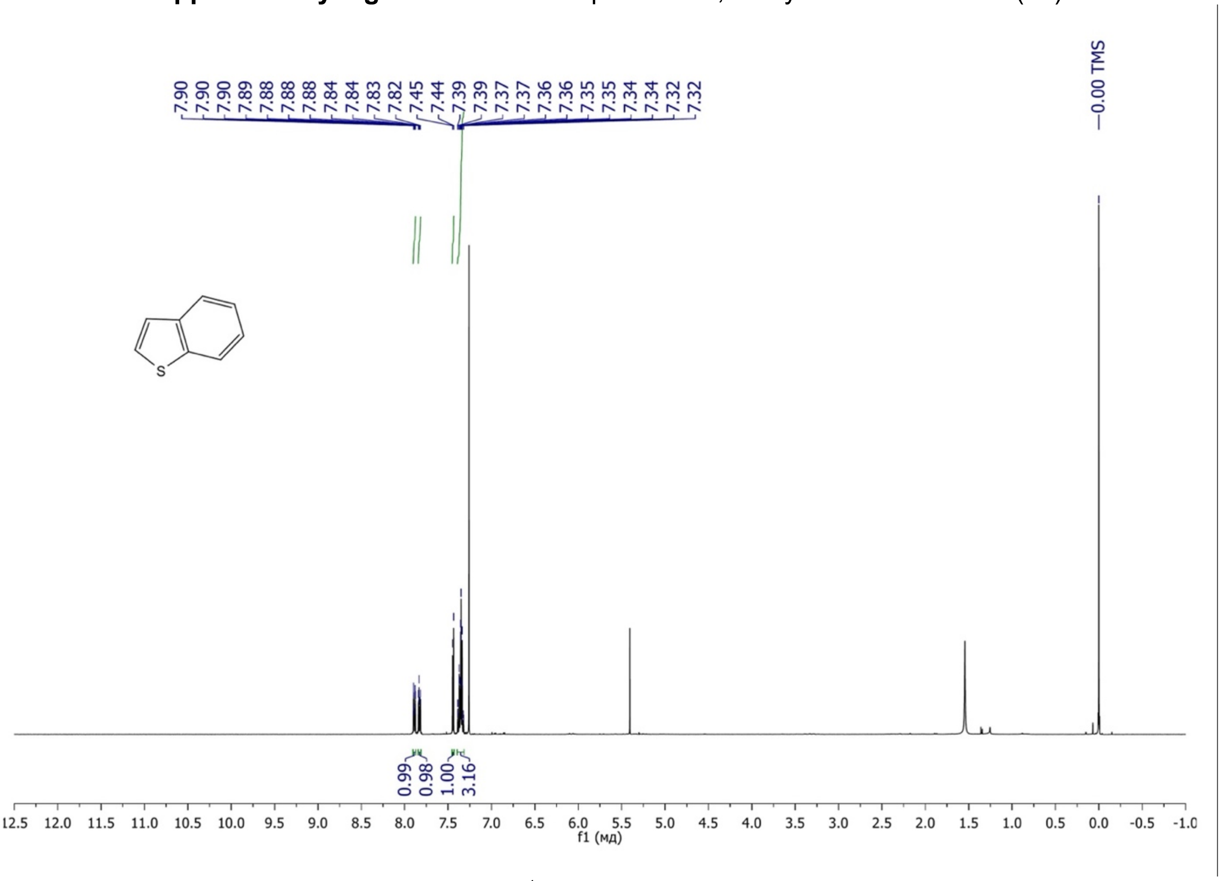

Supplementary Figure 74. ^{1}H NMR spectra of benzothiophene (**8b**).

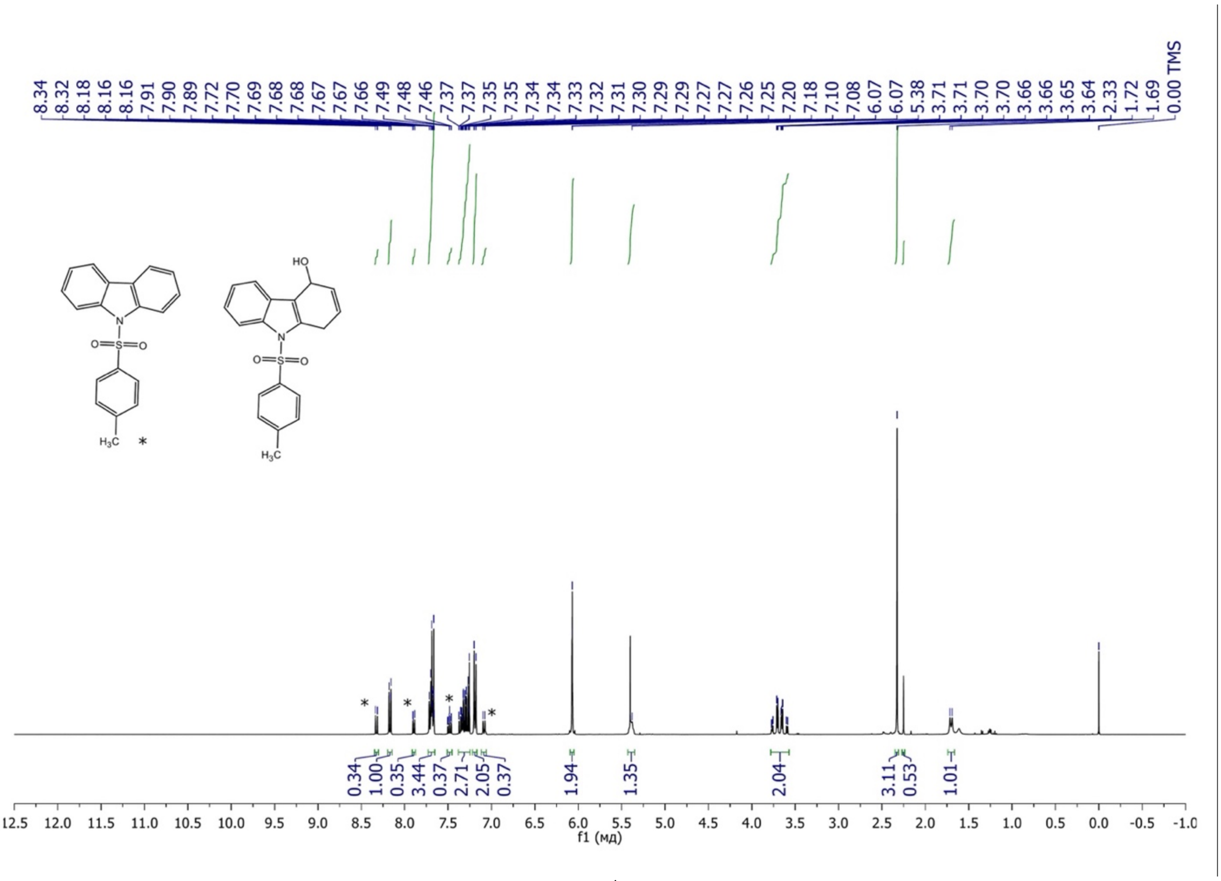

Supplementary Figure 75. ^{1}H NMR spectra mixture of 9-tosyl-4,9-dihydro-1*H*-carbazol-4-ol (**6a)** and carbazole (**6b)**

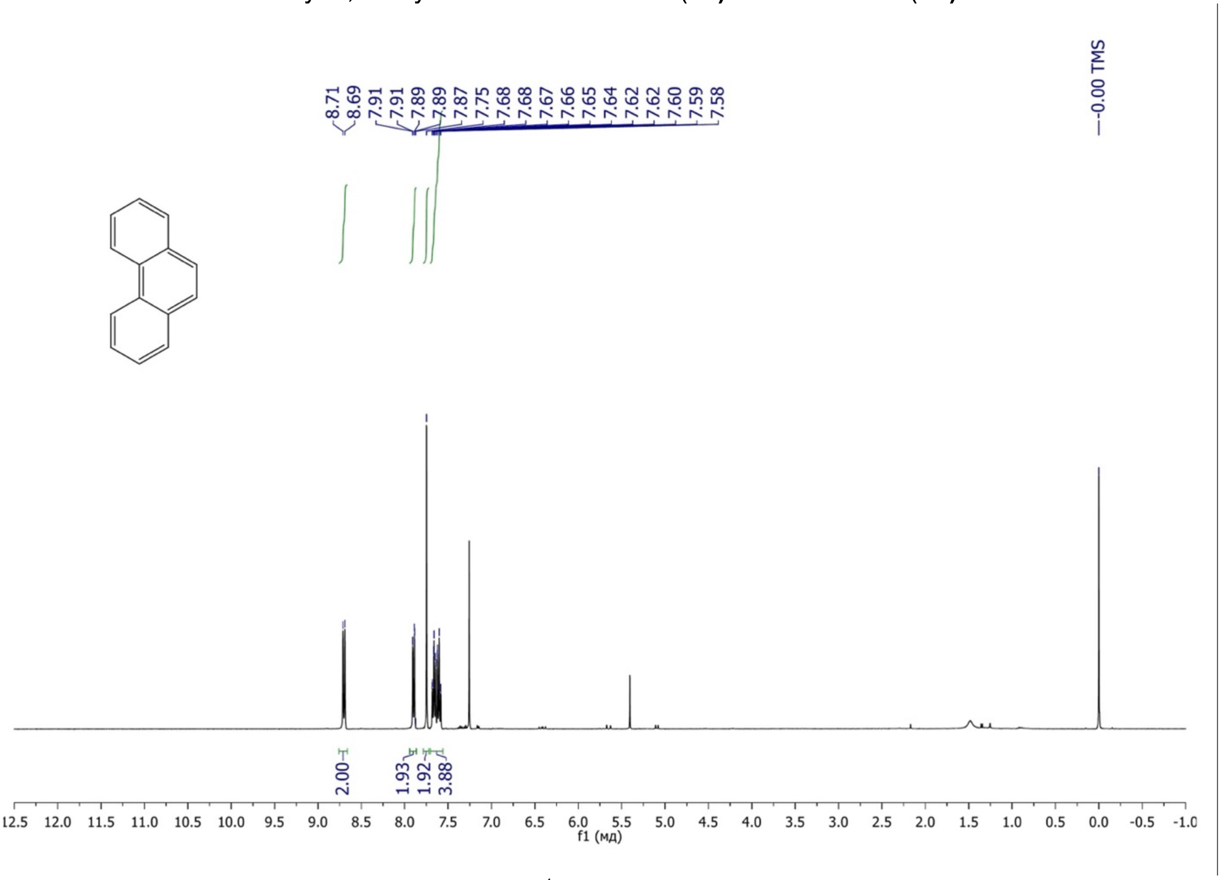

Supplementary Figure 76. ^{1}H NMR spectra of phenanthrene (**9a**)

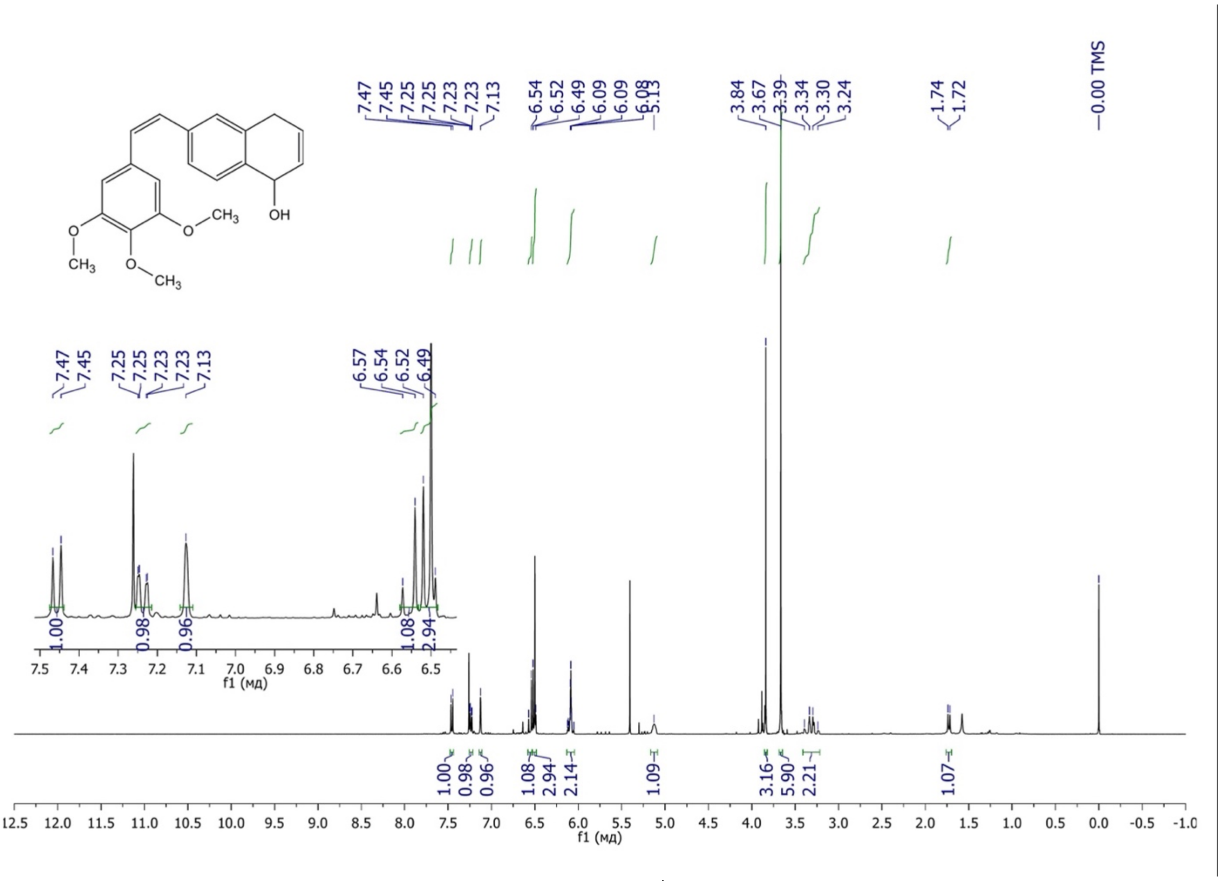

Supplementary Figure 77. ^{1}H NMR spectra of (*Z*)-6-(3,4,5-trimethoxystyryl)-1,4-dihydronaphthalen-1-ol (**20a**)

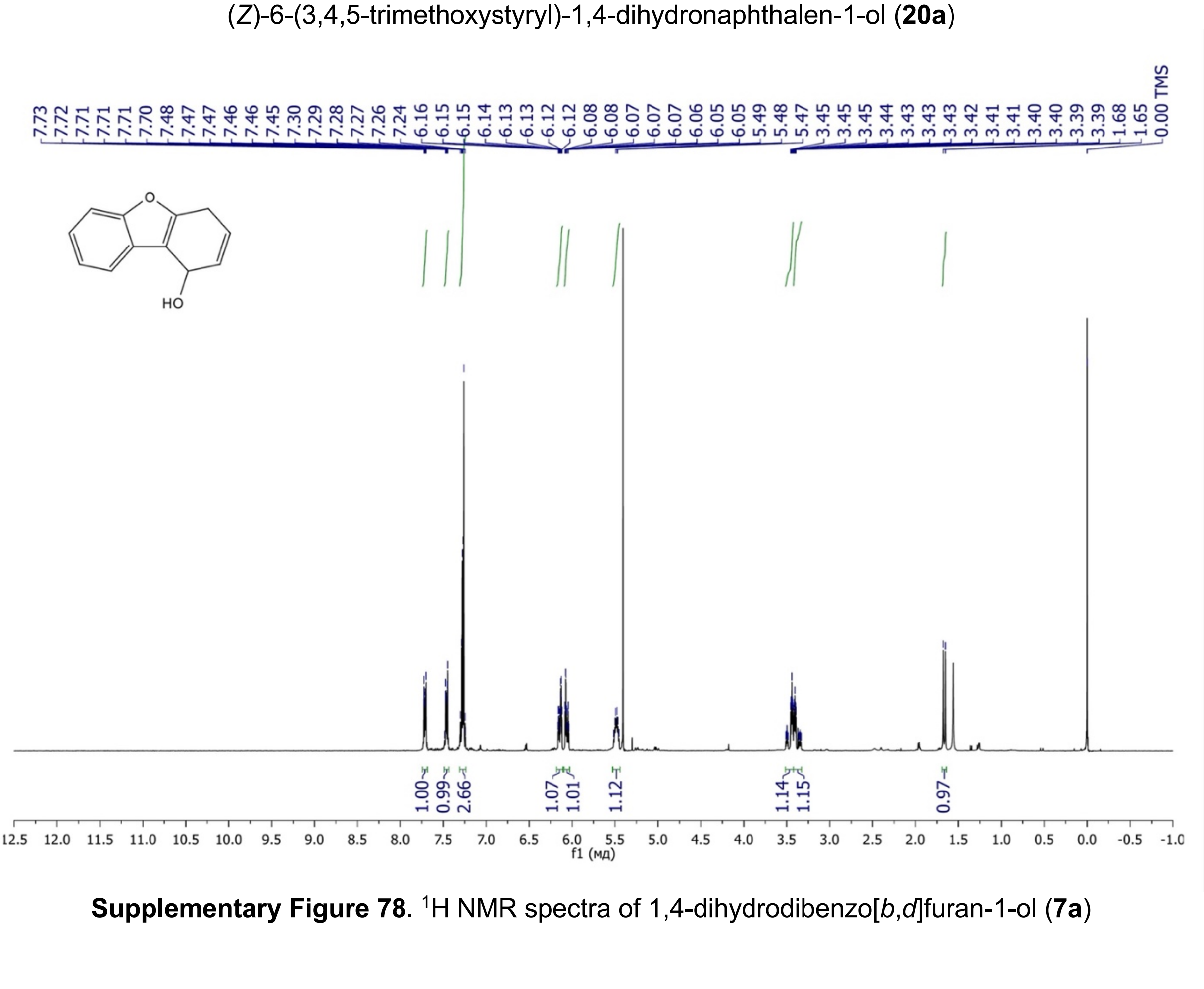

Supplementary Figure 78. ^{1}H NMR spectra of 1,4-dihydrodibenzo[*b*,*d*]furan-1-ol (**7a**)

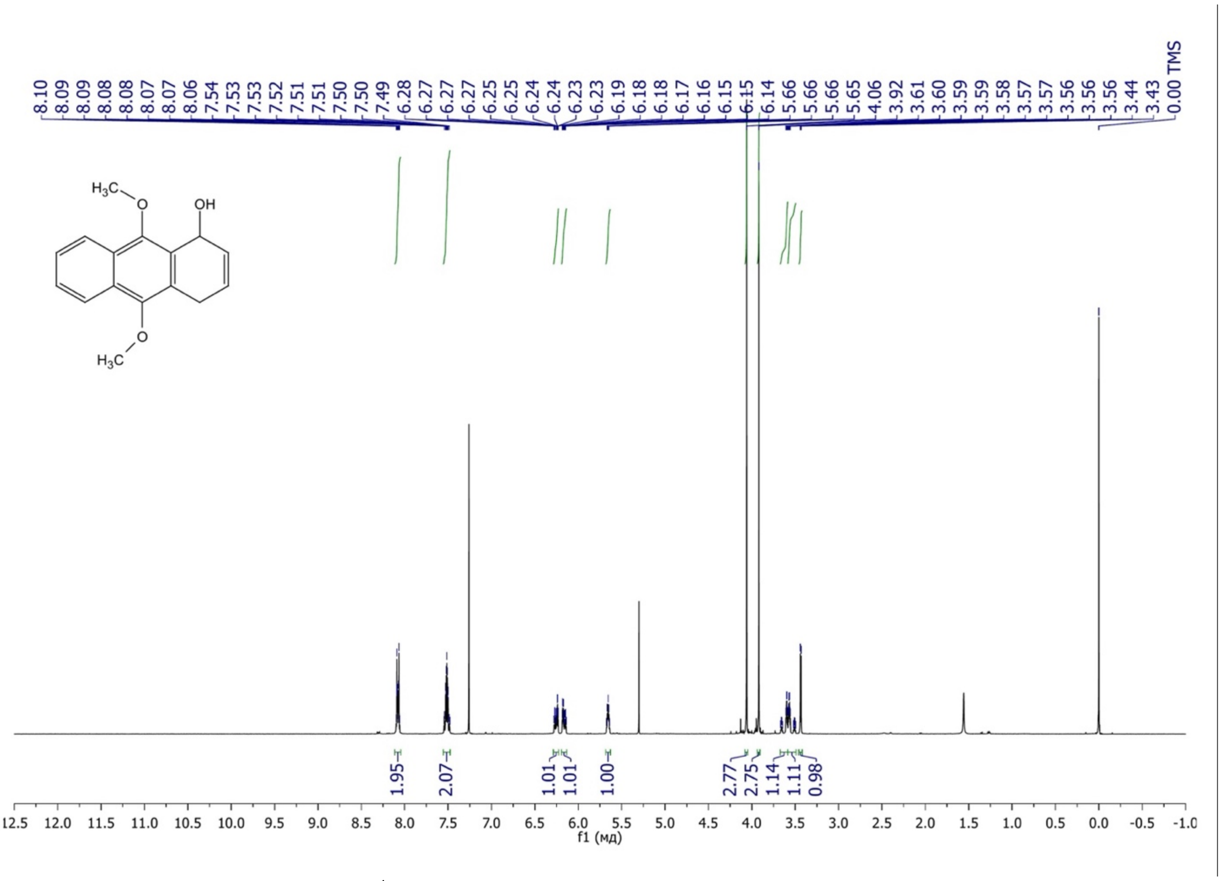

Supplementary Figure 79. ^{1}H NMR spectra of 9,10-dimethoxy-1,4-dihydroanthracen-1-ol (**4a**)

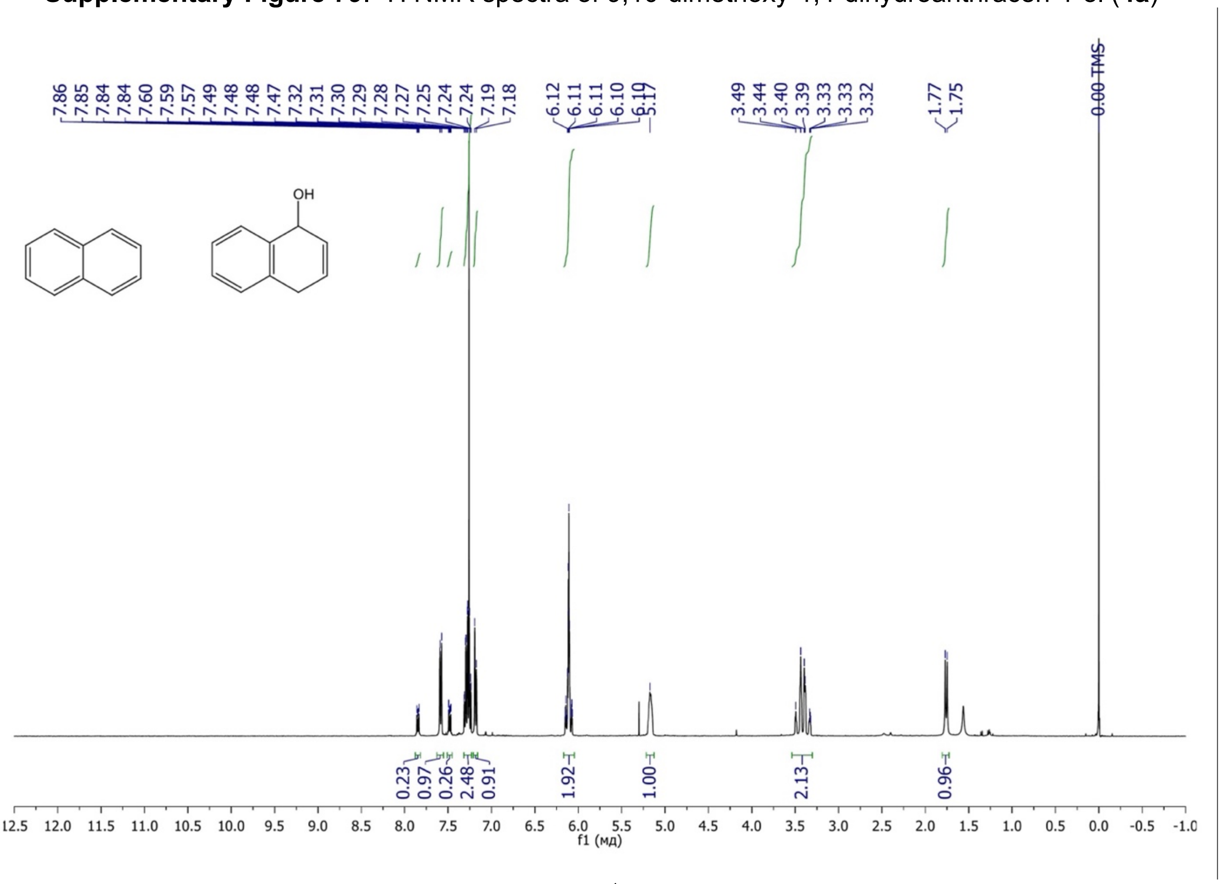

Supplementary Figure 80. ^{1}H NMR spectra mixture of
naphthalene (**13**) and 1,4-dihydronaphthalen-1-ol (**2a**)

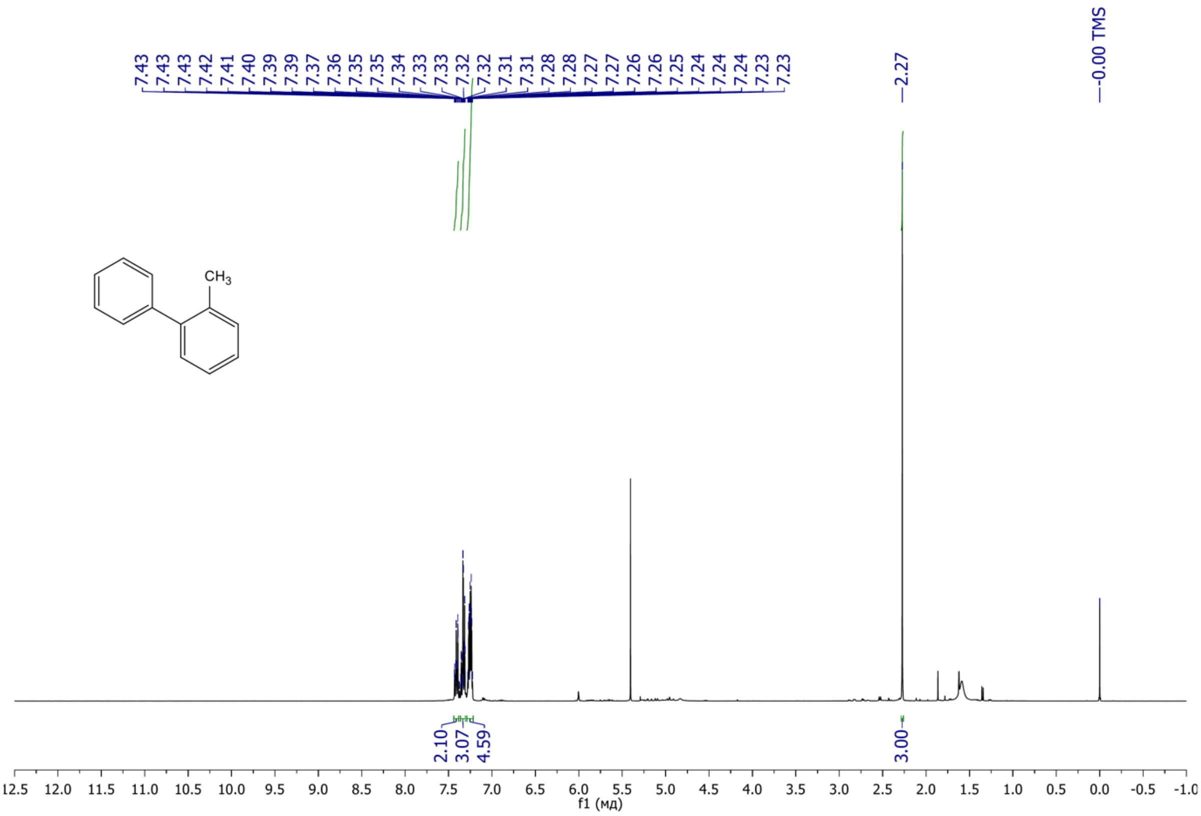

Supplementary Figure 81. ^{1}H NMR spectra of 2-methyl-1,1'-biphenyl (**3b**)

3. Supplementary References

1. Eda, S. *et al.* Biocompatibility and therapeutic potential of glycosylated albumin artificial metalloenzymes. *Nature Catalysis* **2**, 780–792 (2019).
2. Sabatino, V., Rebelein, J. G. & Ward, T. R. “Close-to-Release”: Spontaneous Bioorthogonal Uncaging Resulting from Ring-Closing Metathesis. *Journal of the American Chemical Society* **141**, 17048–17052 (2019).
3. Yoshida, K., Horiuchi, S., Iwadate, N., Kawagoe, F. & Imamoto, T. An Efficient Route to Benzene and Phenol Derivatives via Ring-Closing Olefin Metathesis. *Synlett* **2007**, 1561–1564 (2007).
4. Furmick, J. K. *et al.* Modeling, Synthesis and Biological Evaluation of Potential Retinoid X Receptor-Selective Agonists: Novel Halogenated Analogues of 4-[1-(3,5,5,8,8-Pentamethyl-5,6,7,8-tetrahydro-2-naphthyl)ethynyl]benzoic Acid (Bexarotene). *ChemMedChem* **7**, n/a-n/a (2012).
5. Nitti, A. *et al.* One-Pot Regiodirected Annulations for the Rapid Synthesis of Ï-Extended Oligomers. *Organic Letters* **22**, 3263–3267 (2020).
6. Wood, T. K., Piers, W. E., Keay, B. A. & Parvez, M. Synthesis and comparative characterization of 9-boraanthracene, 5-boranaphthacene, and 6-borapentacene stabilized by the H2IMes carbene. *Chemistry - A European Journal* **16**, 12199–12206 (2010).
7. Nomura, S., Endo-Umeda, K., Makishima, M., Hashimoto, Y. & Ishikawa, M. Development of Tetrachlorophthalimides as Liver X Receptor β (LXRβ)-Selective Agonists. *ChemMedChem* **11**, 2347–2360 (2016).
8. Bulman Page, P. C., Chan, Y., Noor Armylisas, A. H. & Alahmdi, M. Asymmetric epoxidation of chromenes mediated by iminium salts: Synthesis of mollugin and (3S,4R)-trans-3,4-dihydroxy-3,4-dihydromollugin. *Tetrahedron* **72**, 8406–8416 (2016).
9. Auzzas, L. *et al.* Non-natural macrocyclic inhibitors of histone deacetylases: Design, synthesis, and activity. *Journal of Medicinal Chemistry* **53**, 8387–8399 (2010).
10. Watson, I. D. G., Ritter, S. & Toste, F. D. Asymmetric Synthesis of Medium-Sized Rings by Intramolecular Au(I)-Catalyzed Cyclopropanation. *Journal of the American Chemical Society* **131**, 2056–2057 (2009).
11. Akai, S. *et al.* Highly regioselective nucleophilic carbon - Carbon bond formation on furans and thiophenes initiated by pummerer-type reaction. *Organic Letters* **6**, 3793–3796 (2004).
