# Supplementary material for: Synthetic prodrug design enables biocatalytic activation in mice to elicit tumor growth suppression
Source: Nat Commun. 2022 Jan 10;13:39. doi: 10.1038/s41467-021-27804-5 (PMC8748823; doi:10.1038/s41467-021-27804-5)
Supplement: Supplementary file 3 — Reporting Summary [file 41467_2021_27804_MOESM3_ESM.pdf]

nature portfolio

Corresponding author(s): Katsunori Tanaka

Last updated by author(s): Nov 14, 2021

Reporting Summary

Nature Portfolio wishes to improve the reproducibility of the work that we publish. This form provides structure for consistency and transparency in reporting. For further information on Nature Portfolio policies, see our Editorial Policies and the Editorial Policy Checklist.

Statistics

For all statistical analyses, confirm that the following items are present in the figure legend, table legend, main text, or Methods section.

n/a	Confirmed	
☐	☒	The exact sample size (n) for each experimental group/condition, given as a discrete number and unit of measurement
☐	☒	A statement on whether measurements were taken from distinct samples or whether the same sample was measured repeatedly
☒	☐	The statistical test(s) used AND whether they are one- or two-sided<br>*Only common tests should be described solely by name; describe more complex techniques in the Methods section.*
☒	☐	A description of all covariates tested
☒	☐	A description of any assumptions or corrections, such as tests of normality and adjustment for multiple comparisons
☒	☐	A full description of the statistical parameters including central tendency (e.g. means) or other basic estimates (e.g. regression coefficient) AND variation (e.g. standard deviation) or associated estimates of uncertainty (e.g. confidence intervals)
☐	☒	For null hypothesis testing, the test statistic (e.g. F, t, r) with confidence intervals, effect sizes, degrees of freedom and P value noted<br>*Give P values as exact values whenever suitable.*
☒	☐	For Bayesian analysis, information on the choice of priors and Markov chain Monte Carlo settings
☒	☐	For hierarchical and complex designs, identification of the appropriate level for tests and full reporting of outcomes
☒	☐	Estimates of effect sizes (e.g. Cohen's d, Pearson's r), indicating how they were calculated

Our web collection on statistics for biologists contains articles on many of the points above.

Software and code

Policy information about availability of computer code

Data collection	MALDI-TOF spectrometry data was collected on Shimadzu MALDI Solutions (Version 2.6.0). HPLC data was collected on LabSolutions Realtime Analysis version 5.81 SP1 software. MS data was collected on Bruker Daltonics Hystar version 3.2 SR4 software. NMR spectroscopy was collected using JNM-AL version 6.0 software. Molecular docking was calculated using Autodock 4.2 software version 1.1.2 software. Fluorescence data was collected using SoftMax Pro version 7.0.3 software. All software packages and code used are commercially or freely available.
Data analysis	MALDI-TOF spectrometry data was analyzed by Shimadzu MALDI Solutions (Version 2.6.0). HPLC data was analyzed on LabSolutions Postrun Analysis version 5.81 SP1 software. MS data was analyzed on Bruker Daltonics Compass DataAnalysis version 4.2 (Build 383.1). NMR spectroscopy was analyzed using JEOL Delta version 5.0.2 software. Molecular docking was graphically implemented using AutoDockTools version 1.5.6 software. Protein-ligand figure generation was performed using PyMOL version 0.99rc6 and Discovery Studio Visualizer version 17.2.0.16349 software. Fluorescence data was analyzed using SoftMax Pro version 7.0.3 software. All software packages and code used are commercially or freely available.

For manuscripts utilizing custom algorithms or software that are central to the research but not yet described in published literature, software must be made available to editors and reviewers. We strongly encourage code deposition in a community repository (e.g. GitHub). See the Nature Portfolio guidelines for submitting code & software for further information.

Data

Policy information about availability of data

All manuscripts must include a data availability statement. This statement should provide the following information, where applicable:

- Accession codes, unique identifiers, or web links for publicly available datasets
- A description of any restrictions on data availability
- For clinical datasets or third party data, please ensure that the statement adheres to our policy

To carry out molecular docking studies, the three-dimensional X-ray structure of tubulin (PDB ID: 5LYJ) was first taken from the Protein Data Bank. Experimental data that supports these results and other findings are available with the article, and can also be obtained from the corresponding author upon reasonable request. The source data underlying Figs. 2; 3b–d; 4b–d; 5a–c; 6b–c, e, and Supplementary Figs. 4, 33, 37, 39 and Supplementary Table 5 are provided as a Source Data file .

Field-specific reporting

Please select the one below that is the best fit for your research. If you are not sure, read the appropriate sections before making your selection.

☒ Life sciences ☐ Behavioural & social sciences ☐ Ecological, evolutionary & environmental sciences

For a reference copy of the document with all sections, see nature.com/documents/nr-reporting-summary-flat.pdf

Life sciences study design

All studies must disclose on these points even when the disclosure is negative.

Sample size	In both in cellulo and in vivo experiments, we have followed widely accepted standards of scientific practice. We used at least 3 biological replicates or 5 animals per group to calculate the means and standard deviations, as well as to perform statistical analysis.
Data exclusions	No data were excluded from the analysis, unless an obvious error/spillage/fault was encountered during the experiment.
Replication	All experiments were replicated at least three times (three independent cell or animals). Exact number of samples and animals are shown in figures legend.
Randomization	For animal experiment tumour bearing mice were randomly devided for four groups.
Blinding	There was no blinding. For data collection, we have followed standard laboratory procedures of randomization. All samples for comparison were collected and analyzed under the same conditions.

Reporting for specific materials, systems and methods

We require information from authors about some types of materials, experimental systems and methods used in many studies. Here, indicate whether each material, system or method listed is relevant to your study. If you are not sure if a list item applies to your research, read the appropriate section before selecting a response.

Materials & experimental systems

n/a	Involved in the study	
☒	☐	Antibodies
☐	☒	Eukaryotic cell lines
☒	☐	Palaeontology and archaeology
☐	☒	Animals and other organisms
☒	☐	Human research participants
☒	☐	Clinical data
☒	☐	Dual use research of concern

Methods

n/a	Involved in the study	
☒	☐	ChIP-seq
☒	☐	Flow cytometry
☒	☐	MRI-based neuroimaging

Eukaryotic cell lines

Policy information about cell lines

Cell line source(s)	HeLa S3, A549, PC-3, MCF-7 cell lines were obtained from RIKEN Cell Bank.
Authentication	All obtained from RIKEN Cell Bank, where Quality Control includes STR analyses.
Mycoplasma contamination	All obtained from RIKEN Cell Bank, where Quality Control includes tests for Mycoplasma infection and they were negative for

Mycoplasma contamination	mycoplasma contamination.
Commonly misidentified lines (See ICLAC register)	All obtained from RIKEN Cell Bank, where Quality Control includes tests for misidentification via Short Tandem Repeat polymorphism analysis and no commonly misidentified cell lines were used.

Animals and other organisms

Policy information about studies involving animals; ARRIVE guidelines recommended for reporting animal research

Laboratory animals	6 week-old female nude mice BALB/cAJcl-nu/nu were housed in a facility with controlled temperature, salinity, aeration, and a standard 12 h light/12 h dark cycle.
Wild animals	Study did not involve wild animals.
Field-collected samples	Study did not involve samples collected from the field.
Ethics oversight	All animal experiments were carried out with approval by RIKEN's Animal Ethics Committee.

Note that full information on the approval of the study protocol must also be provided in the manuscript.
